# Fluorination of White Phosphorus with Fluoride Salts and Quinone Oxidants

**DOI:** 10.1021/jacs.6c04361

**Published:** 2026-07-23

**Authors:** Benjamin Falge, Maximilian Philipp, Tobias Villing, Ruth M. Gschwind, Rafael E. Rodríguez-Lugo, Robert Wolf

**Affiliations:** † Institute of Inorganic Chemistry, 9147 University of Regensburg, Regensburg 93040, Germany; ‡ Institute of Organic Chemistry, 9147 University of Regensburg, Regensburg 93040, Germany

## Abstract

We report the synthesis of hexafluorophosphate (PF_6_^–^) salts directly from white phosphorus (P_4_) and simple fluorides MF (M = Li, Na, K, Cs, NMe_4_) in a single step. The utilization of *p*-quinones, such as 2,3-dichloro-4,5-dicyano-1,4-benzoquinone (DDQ) offers a selective fluorination pathway, which affords MPF_6_ salts in up to 95% yield in acetonitrile (MeCN). NMR investigations of the DDQ-mediated P_4_ fluorination reaction revealed the formation of PF_3_ and PF_5_·MeCN as key reaction intermediates. Donor–acceptor interactions between DDQ and fluoride are critical, effectively increasing the fluoride concentration and enabling efficient fluorination to PF_6_^–^. Tetraethylene glycol (TEG) selectively stops the reaction at the adduct K­[PF_5_·TEG]. In contrast, noncoordinating dichloromethane (DCM) favors PF_3_ formation by suppressing EDA interactions through low fluoride solubility, possibly via the tris­(hydroquinonyl)­phosphite P­(DDQH)_3_, underscoring the critical role of fluoride solubility. This work demonstrates that quinone–fluoride interactions can be exploited for oxidative fluorination chemistry, establishing a new conceptual framework for P_4_ fluorination.

## Graphical abstract

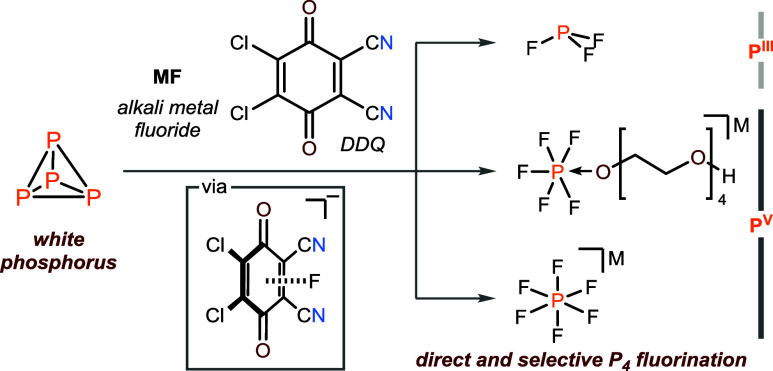

## Introduction

Fluorine imparts unique physicochemical properties due to its high electronegativity, small size, and strong element-fluorine bonds. As a result, the fluorination of organic and inorganic compounds has gained widespread importance across diverse areas of chemistry, including pharmaceuticals^1–3^ agrochemicals,^4,5^ functional materials,^6,7^ and energy-related applications.^8^

Elemental fluorine (F_2_) is the most powerful fluorinating agent, converting a wide range of substrates into highly fluorinated products. However, its practical use is severely limited by its hazardous nature, extreme reactivity, and poor selectivity, often leading to unselective or overoxidized products. Consequently, fluorine surrogates have been developed to enable safer and more controlled fluorination protocols.^9–13^ An increasingly attractive alternative strategy involves using fluoride salts in combination with external oxidants, thereby enabling the in situ generation of electrophilic fluorinating species from simple, readily available fluoride sources.^13–15^ This approach benefits from the low cost and ease of handling of inorganic fluorides such as KF or CsF, while avoiding the direct use of hazardous F_2_. Notable examples include oxidative fluorination systems based on fluoride salts and hypervalent iodine oxidants as well as transition-metal-mediated systems in which Mn, Ni, or Ag complexes are oxidized to higher-valent fluorinating species.^16–20^ In addition, electrochemistry has emerged as a powerful method for achieving fluorination under controlled conditions without the need for stoichiometric chemical oxidants.^21–23^

The fluorination of elemental phosphorus has garnered significant attention due to the crucial role of lithium hexafluorophosphate (LiPF_6_) as a component of lithium-ion batteries.^24–31^ Industrially, hexafluorophosphates are produced via the oxidation of white phosphorus (P_4_) to PCl_5_ using Cl_2_ gas, followed by treatment with anhydrous hydrofluoric acid to generate phosphorus pentafluoride (PF_5_). Reaction of PF_5_ with metal fluorides yields the desired hexafluorophosphate salts (MPF_6_).^32–37^ Recent reports have described alternative strategies, such as the reaction of PCl_5_ or P_4_O_10_ with fluorspar (CaF_2_) at 350 °C and 1.0 MPa, followed by a reaction with LiF,^38,39^ and the direct oxidation of red phosphorus (P_red_) in an F_2_ atmosphere in the presence of LiF.^40–42^ Complementing these reports, Cummins and coworkers achieved the synthesis of [nBu_4_N]­PF_6_ through the fluorination of P­(SiCl_3_)^2–^, prepared from trimetaphosphate (P_3_O_9_^3–^). This method requires XeF_2_ as an expensive fluorinating reagent.^43,44^

Several studies have described the fluorination of elemental phosphorus and of P­(III) and P­(V) compounds using a combination of oxidants and fluoride salts (Scheme 1a).^45–51^ Notably, Riesel has reported that Appel-type reactions of P_4_ and P­(OR)_3_ (R = alkyl, aryl) with CCl_4_ and NEt_3_·n­(HF) (n = 1–3) afford mixtures of partially fluorinated phosphorus species, including HPF_5_^–^. However, the formation of PF_6_^–^ was not reported.^45–48^ Additionally, Yang and coworkers have demonstrated the fluorination of P­(V) compounds using catalytic CuBr_2_ and DDQ as the terminal oxidant.^49^ This reaction was proposed to proceed via a copper-promoted insertion of DDQ into the phosphonate P–H bond, followed by fluoride substitution. Using a different strategy, Grützmacher, Pitts, and Togni have achieved the deoxyfluorination of P­(V) oxides to generate difluorophosphoranes with oxalyl chloride and KF.^50^

**Scheme 1:**
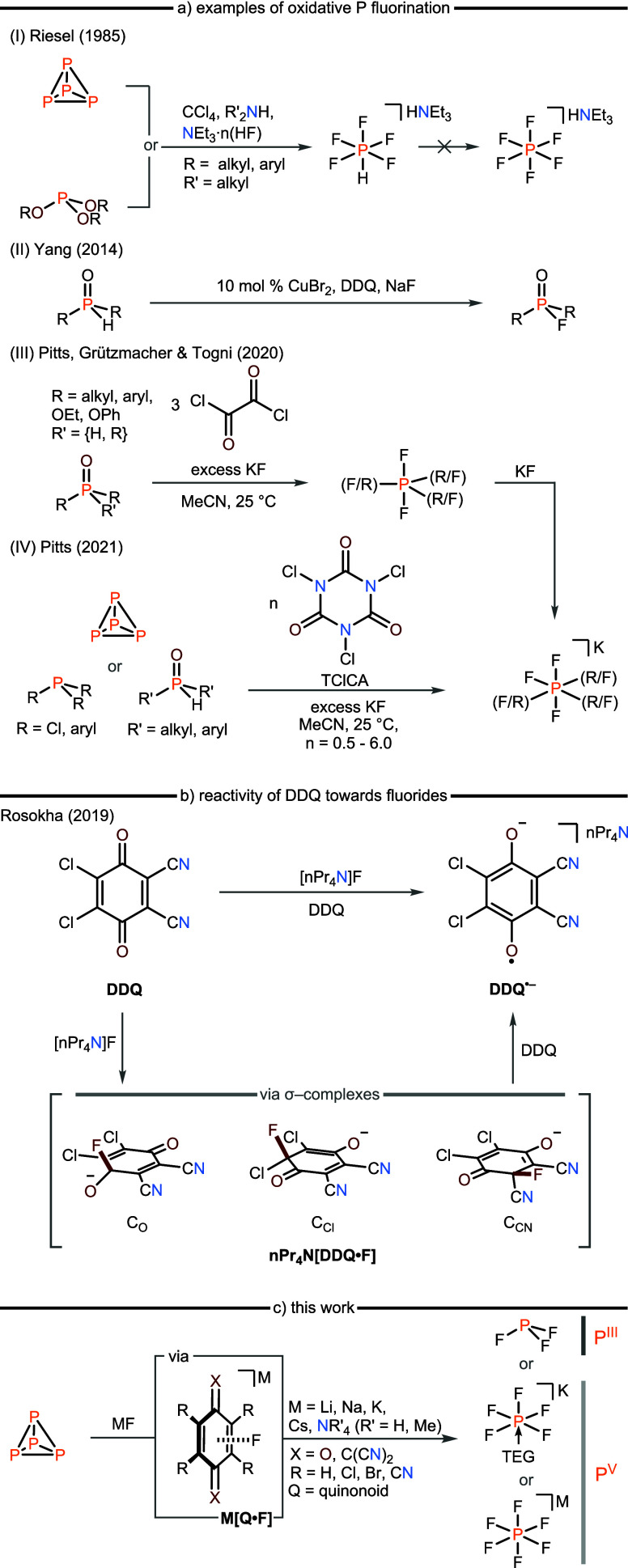
a) Examples of Oxidative Fluorination of Phosphorus Compounds (ref. 45–51). b) Reactivity of DDQ toward [nPr_4_N]­F, Forming DDQ^·–^, among Other Unidentified Products. c) Quinone-Mediated Oxidative Fluorination of P_4_ Explored in This Work (TEG = Tetraethylene Glycol)

Notably, a subsequent report describes the oxidative fluorination of P_4_, P_red_ and P­(III) compounds using trichloroisocyanuric acid (TClCA) and KF, resulting in the preparation of KPF_6_ from P_4_ in 62% yield.^51^ Similar to classical methods, TClCA acts as a chlorine source to generate chlorinated intermediates.^52^

Despite many significant advances in P_4_ functionalization,^53–65^ the direct, one-step synthesis of hexafluorophosphates or other fluorinated phosphorus compounds from P_4_ (or P_red_) remains challenging. Rosokha and coworkers reported that DDQ and other *p*-benzoquinones form EDA complexes with fluoride. In the case of DDQ, F^–^ anions induce the formation of the *p*-benzoquinone radical anion DDQ^·–^ via nucleophilic σ-adduct formation, followed by oxidation by a second DDQ equivalent (Scheme 1b).^66^ Building on these findings, we hypothesized that combining fluoride with electron-deficient quinoids could enable P_4_ fluorination. We now report a one-step method to synthesize hexafluorophosphates from P_4_, inorganic fluorides, and *p*-quinonoid oxidants, which serve as both oxidants and fluoride-ion shuttles (Scheme 1c). Our studies have revealed divergent reaction pathways that provide access to intermediates such as PF_3_ and Lewis-base-stabilized PF_5_ adducts. The DDQ-mediated fluorination reaction has been analyzed in detail using NMR and UV–Vis spectroscopic studies, confirming that the interaction between DDQ and fluoride ions is central to the observed reactivity and directs reaction selectivity.^66^

## Results and Discussion

### Reaction Discovery and Optimization

In initial experiments, we monitored the reaction of P_4_ (0.25 equiv.) with DDQ (5.0 equiv.) in the presence of KF (7.5 equiv.) in dimethylformamide (DMF) after 18 h at 65 °C by quantitative ^31^P­{^1^H} NMR spectroscopy (Table 1, SI Section S1.3). The complete conversion of P_4_ was observed, accompanied by the formation of KPF_6_ as the main product, in 63% yield (δ ^31^P −143.8 ppm, δ ^19^F −73.2 ppm, ^1^ *J*_PF_ = 707.0 Hz, Table S2).^67^ A solvent screening showed that polar aprotic solvents like tetrahydrofuran (THF) and acetone give KPF_6_ in low yield, although high conversions of P_4_ are observed (Table 1). No additional species were detected by ^31^P or ^19^F NMR spectroscopy. The fate of the remaining phosphorus is presently unclear. Acetonitrile (MeCN), the most polar solvent tested, produced the highest yield of KPF_6_ (95%, see Table 1, entries 1–4 and Table S2; 62% isolated yield). Ethanol led to side-product formation due to solvolysis of the fluorinated products, resulting in the formation of OP­(OEt_3_) (δ ^31^P −0.5 ppm, ^3^ *J*_PH_ = 8.0 Hz; Table S2 and Figure S2), among other nonfluorinated products.^68–70^ Using dichloromethane (DCM), the reaction selectively afforded PF_3_ (δ ^31^P 104.6 ppm, ^1^ *J*_PF_ = 1400.9 Hz; Table S2 and Figure S3).^67^ This is attributed to a different pathway operating in DCM (vide infra). Control experiments demonstrated that DDQ was essential for achieving P_4_ fluorination (Table 1, entry 5). Irradiation with blue LED light (456 nm, 7 W) did not affect PF_6_^–^ formation (compare entries 6 and 7, Table S9).^71–74^

**Table 1:**
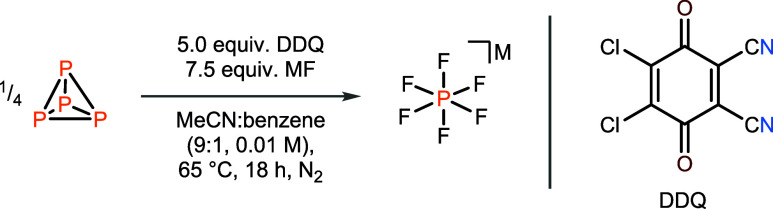
Reaction Optimization and Control Reactions

entry	deviation from standard conditions^a^	Yield^b^
1	None: KF, MeCN, P_4_ (0.01 M), 65 °C, 18 h, N_2_	95
2	DMF	63
3	Acetone	27
4	THF	22
5^c^	no DDQ	n.d.
6	25 °C	41
7	25 °C, blue LED, 456 nm, 7W	42
8	LiF	35
9	CsF	47
10	CaF_2_	n.d.
11	NMe_4_F	92
12	reaction performed in air	14
13^d^	P_red_ instead of P_4_ (N_2_/air)	11/22

a Standard conditions: KF, MeCN, P_4_ (0.01 M), 65 °C, 18 h, N_2_ atmosphere. All reactions were performed using 0.01 mmol P_4_ (100 μL, 0.1 M in benzene). Reactions on a 0.1 mmol scale using solid P_4_ yielded results similar to those under standard conditions (KPF_6_: 85%, spectroscopic yield, 62% isolated yield, see SI, Sections 3.3, Figures S39–S46).

b Yield of MPF_6_ and conversion of P_4_ were determined by quantitative ^31^P­{^1^H} NMR spectroscopy (Figure S1). Conversion of P_4_ in all cases >99% unless noted otherwise (Table S2).

c No conversion of P_4_.

d Reaction time: 5 d.

LiF and CsF yielded moderate amounts of the corresponding MPF_6_ salts (35–47%; Table 1, entries 8 and 9). The yield of LiPF_6_ did not improve upon the addition of crypt-222 (3.75 equiv.) and instead led to a less selective reaction (see Table S5 and Figures S28 and S29). Using CaF_2_, the complete conversion of P_4_ was observed, but no fluorinated products were detected (Table 1, entry 10). This is probably due to the poor solubility of CaF_2_ in MeCN.^75,76^ Notably, NMe_4_F proved to be an excellent fluoride source, affording (NMe_4_)­PF_6_ in >90% yield (Table 1, entry 11). Similarly, NEt_3_·3­(HF), NH_4_F, and KBF_4_ gave the corresponding MPF_6_ salts in yields ranging from 27% to 73% (Table S5).^77^ The reaction is sensitive to oxygen and moisture. When the reaction was carried out in air, KPF_6_ was obtained in low yield (14%), alongside KPO_2_F_2_ (Table 1, entry 12, δ ^31^P −17.9 ppm, t, δ ^19^F −82.9 ppm, d, ^1^ *J*_PF_ = 957.7 Hz; SI Section S3.1).^67,68^ P_red_ can also be fluorinated, affording KPF_6_ in low yields when the reaction is performed in air or under an N_2_ atmosphere (22% and 11% yield, respectively; Table 1, entry 13, Figures S36–S38). These results indicate that an inert atmosphere is mainly necessary to mitigate premature oxidation of P_4_ by air.

### Impact of Oxidizing Agents

To investigate the role of the oxidant in this transformation, we evaluated various electron acceptors in the fluorination of P_4_ to KPF_6_ (Figure 1a, Tables S7–S10).^78,80^ No general correlation of product yield with formal half-wave potentials (E_1/2_ vs Fc/Fc^+^ in MeCN) of the oxidants was found. For example, TCNQ (E_1/2_ = −0.30 V) and TCNE (E_1/2_ = −0.27 V)^78,81^ both enabled productive conversions, whereas TEMPO (E_1/2_ = 0.26 V)^82^ gave full conversion of P_4_ but did not result in any fluorination, despite having a significantly higher oxidation potential. This shows that reactivity is not solely governed by oxidation power. Inspection of the mediators that successfully produced KPF_6_ reveals that most of them contained a *p*-quinonoid moiety (Figure 1b). It is important to note, however, that electron-rich quinones failed to produce KPF_6_ (Tables S7–S9).^79,80,83^ KPF_6_ formation is seen only with quinoids that have an oxidation potential of at least −0.4 V vs Fc/Fc^+^. As a result, DDQ, the strongest commercially available quinonoid oxidant (E_1/2_ = 0.13 V), achieved the highest yield (95%).^80^

**Figure 1:**
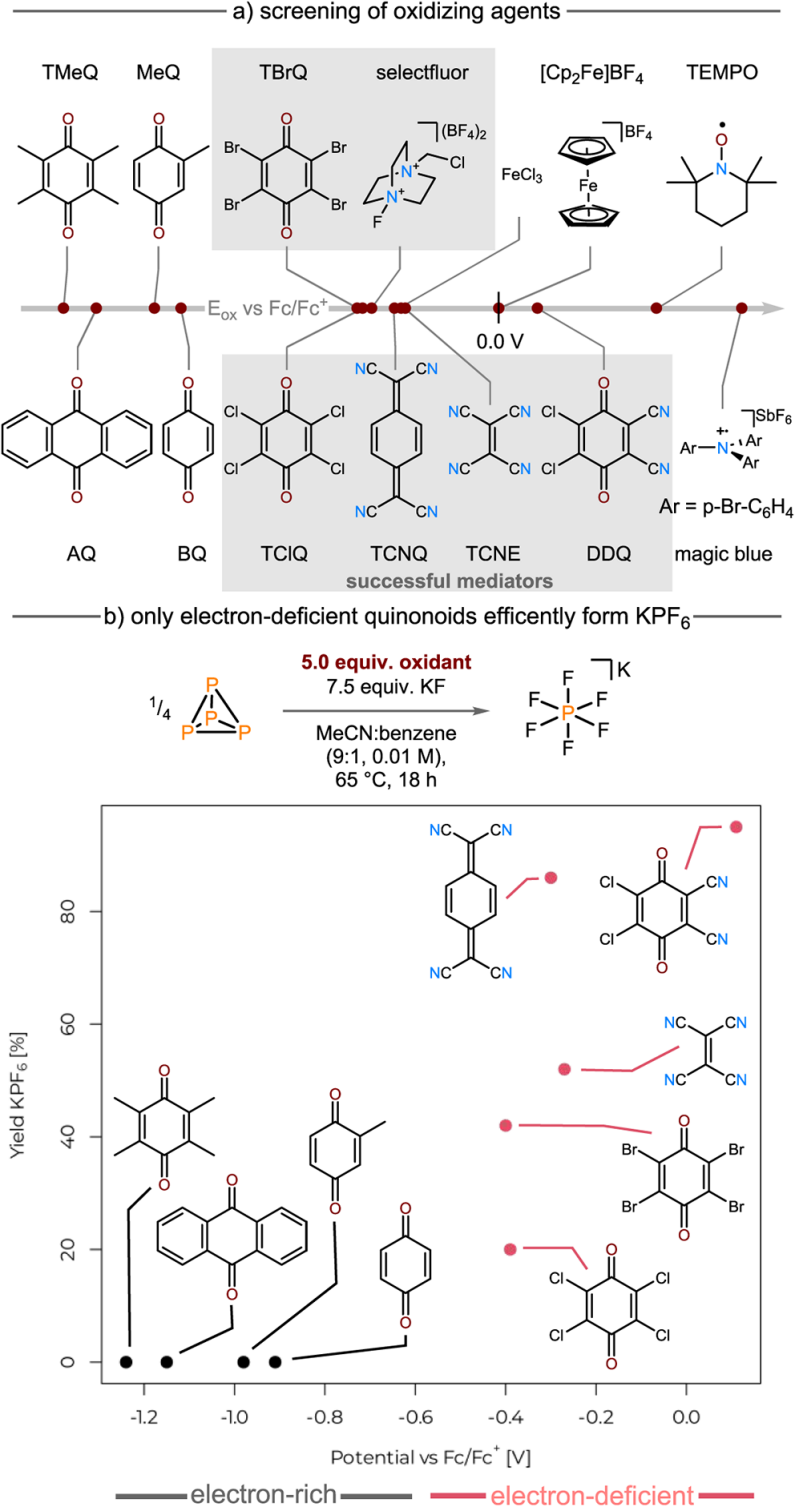
Impact of selected oxidants on P_4_ fluorination reaction. a) Structures and oxidation potential (E_1/2_ vs Fc/Fc^+^ in MeCN) of some selected oxidants used. Successful mediators highlighted in gray. b) Scatter plot showing the yield of KPF_6_ against oxidation potential for *p*-quinonoids in the P_4_ fluorination reaction.

### Studies on the Role of DDQ

Quinonoid compounds participate in electron donor-acceptor interactions with a range of donors, including halides and pseudohalides.^66,84–87^ Rosokha and coworkers previously proposed that the reduction of DDQ occurs via transient nPr_4_N­[DDQ·F] σ-complexes, formed by fluoride addition to electrophilic positions on the quinone ring (C_O_, C_Cl_, or C_CN_; Scheme 1b).^66,84,88^ These intermediates, possessing higher HOMO energies relative to DDQ, are susceptible to single-electron oxidation by a second equivalent of quinone, forming DDQ^·–^.^66,89,90^ To investigate whether similar complexes form under our reaction conditions, we added fluoride salts (MF) to solutions of DDQ in MeCN and monitored the solutions by UV–Vis absorption spectroscopy. The resulting immediate color change from orange to deep purple suggested transient formation of a fluoride-DDQ complex. Upon treatment of DDQ (Figure 2, black line, 0.91 mM, 1.0 equiv.) with substoichiometric NMe_4_F (0.018–0.045 mM, 0.02–0.05 equiv.) in MeCN, the absorption spectra (Figure 2, colored lines) revealed the characteristic absorptions of DDQ^·–^ (Figure 2, black dashed line), suggesting that DDQ is reduced under these conditions (via the pathway shown in Scheme 1b).^66^

**Figure 2:**
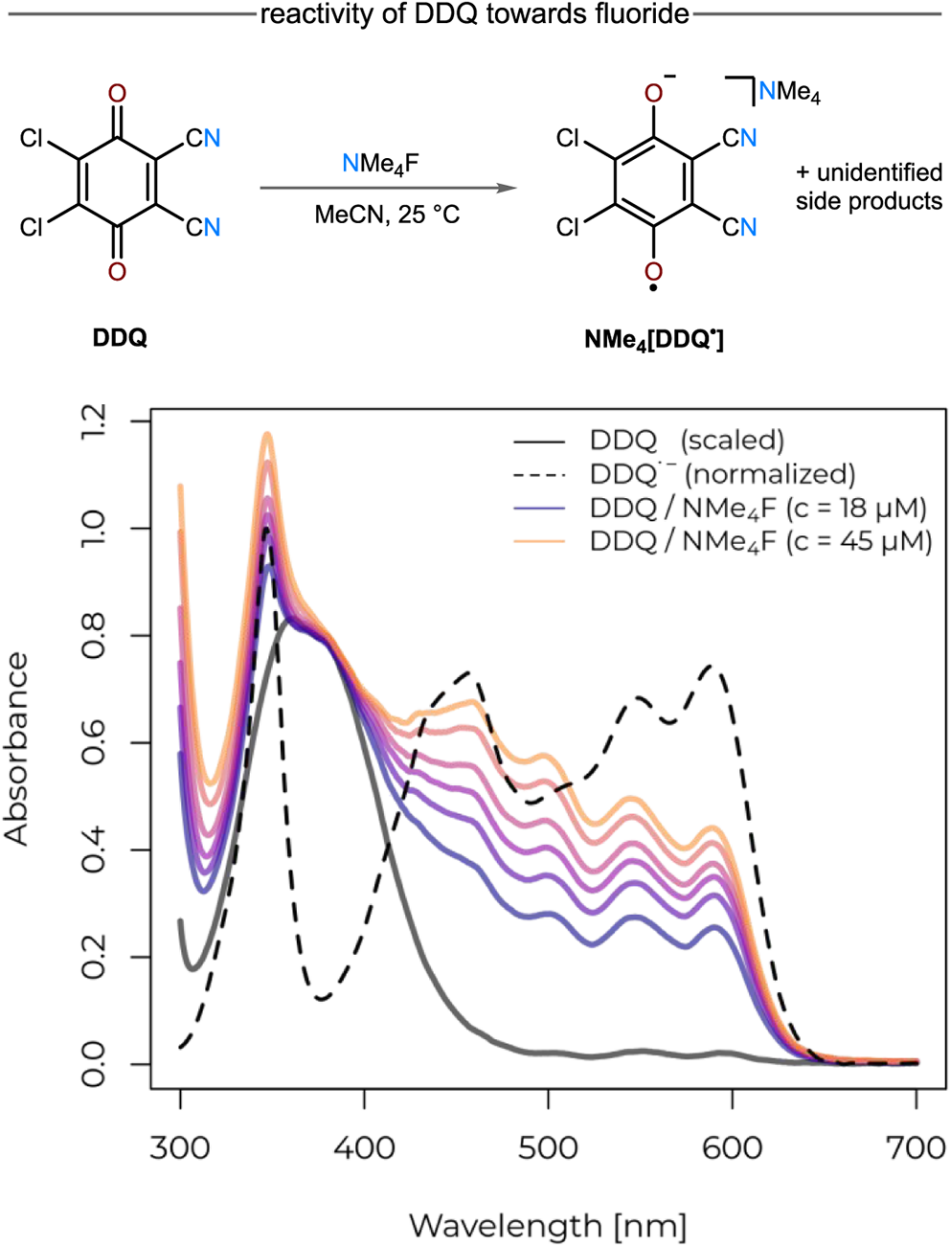
UV–Vis titration of DDQ (0.91 mM in MeCN) with NMe_4_F, showing the formation of DDQ^·–^. Normalized and scaled data were obtained from spectroelectrochemistry of DDQ (see SI, Section S3.6).

^19^F­{^1^H} NMR monitoring of the reaction between DDQ and KF at 65 °C gave an intractable mixture showing a large number of unidentifiable signals (Figure S67), which may possibly arise from the fluorination of DDQ. Since fluoride salts are generally poorly soluble in MeCN,^75^ we presume that the interaction between DDQ and fluoride plays a crucial role in increasing the fluoride concentration. This is in line with the observed reactivity trend among quinonoid oxidants, which all possess electron-deficient π-systems.^91^ In contrast, oxidants that failed to promote fluorination typically possess more electron-rich cores (e.g., *p*-benzoquinone, Figure 2) or do not readily act as fluoride acceptors (e.g., TEMPO). As a result, these reagents oxidize P_4_ without facilitating fluoride transfer, leading to unproductive reaction pathways.

UV–Vis monitoring of the reaction between DDQ and P_4_ in the absence of fluoride reveals no significant changes in the visible region, indicating that no EDA complex forms in this case (SI Section S3.8). In contrast to the rapidly developing DDQ^·–^ signal upon fluoride addition (Figure 2), no accumulation of DDQ^·–^ was observed. Instead, very weak bands assigned to DDQ^·–^ disappeared completely within 3 days (Figures S13–S16). It is important to note that the reaction between DDQ and fluoride occurs very fast after mixing both reagents. Although largely dependent on fluoride solubility (vide infra), it is significantly faster than the reaction of DDQ or DDQ^·–^ with P_4_.

To further gain insight into the role of DDQ, we monitored the reaction of P_4_ and DDQ in the absence of fluoride by ^31^P­{^1^H} NMR spectroscopy in MeCN-*d*_3_/C_6_D_6_ (9:1, v:v, see Scheme 2 and Figure S61). Heating at 65 °C for 18 h resulted in 58% conversion of P_4_ and a new resonance at δ ^31^P 136.1 ppm, assigned to the tris­(hydroquinonyl)­phosphite, P­(DDQH)_3_, which was formed in 5% yield, as determined by ^31^P­{^1^H} NMR integration. The assignment is based on reported resonances of related phosphites (e.g., P­(OPh)_3_: δ ^31^P 126 ppm).^58^ We propose that these hydroquinonyl units are protonated, based on control experiments which showed that DDQH_2_ (1.0 equiv.), upon deprotonation with NaH followed by addition of PCl_3_ (0.33 equiv.), gave the same major ^31^P­{^1^H} NMR signal (δ 136.1 ppm, Figure S58). The protons presumably originate from traces of moisture and not from the solvents MeCN-*d*_3_ or C_6_D_6_, as no signals for deuterated OH groups were detected by ^2^H NMR spectroscopy (Figures S59 and S60). Subsequent addition of KF (7.5 equiv.) to the same reaction mixture afforded KPF_6_ in 82% yield, with full consumption of P_4_ and P­(DDQH)_3_ (Figures S62 and S63). These observations demonstrate that P­(DDQH)_3_ is only a minor intermediate and suggest that other NMR-silent phosphorus species^92^ are present that are also chemically active in the fluorination reaction (Scheme 2).

**Scheme 2:**
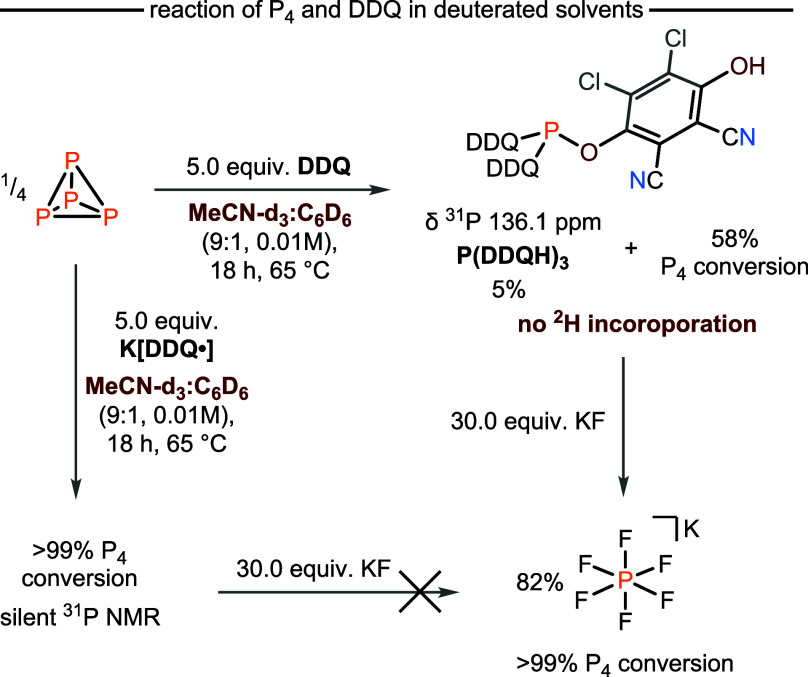
Reactivity of DDQ and K­[DDQ^·^] toward P_4_ and Further Reactivity with KF in Deuterated Solvents

Treatment of K­[DDQ^·^] (5.0 equiv.) with P_4_ (0.25 equiv.) in MeCN-*d*_3_ at 65 °C for 18 h resulted in the complete consumption of P_4_. No other signals were detected in the ^31^P­{^1^H} NMR spectrum of the reaction mixture (Figure S64). Performing the same reaction in the presence of KF resulted only in traces of fluorination products, indicating that K­[DDQ^·^] oxidizes P_4_, but the resulting phosphorus compounds do not participate in the fluorination reaction (Figures S65 and S66).

### In Situ Reaction Monitoring

To further understand the reaction pathways, we monitored the reaction of P_4_ with DDQ and KF in situ by UV–Vis and ^31^P­{^1^H} NMR spectroscopy. UV–Vis monitoring revealed that DDQ is converted into DDQ^·–^ (Figure 3), confirming that DDQ acts as a one-electron acceptor in this reaction. Absorption bands associated with DDQ^2–^ were not detected (Figures S8, S10 and S21, S22).^88^

**Figure 3:**
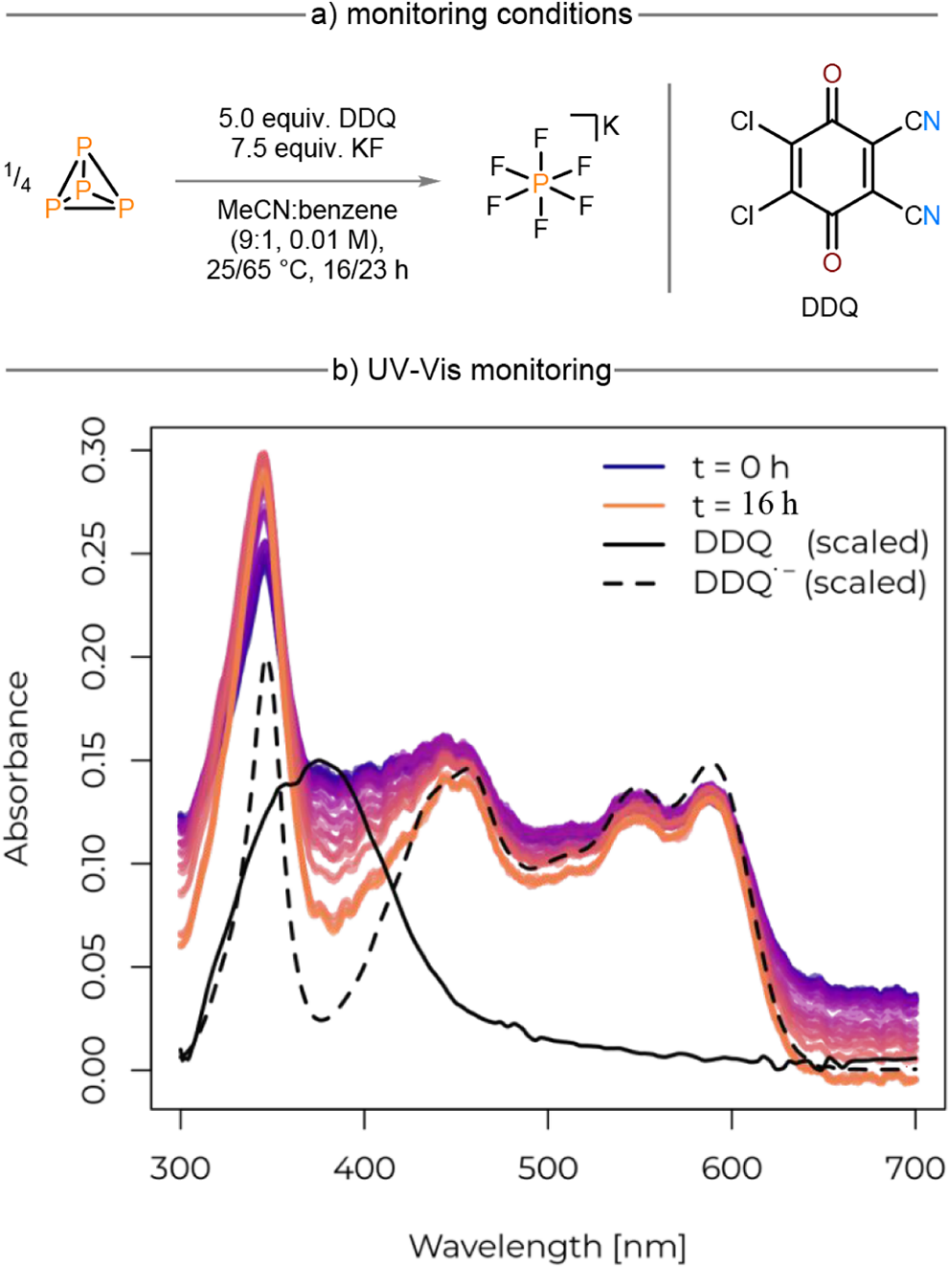
a) Conditions of the in situ reaction monitoring studies. b) UV–Vis reaction monitoring of a dilute reaction solution over 16 h at 25 °C, showing the consumption of DDQ and formation of DDQ^·–^. No DDQ^2–^ was detected. For details, see SI Section S3.6 and S3.18.

^31^P­{^1^H} and ^19^F­{^1^H} NMR reaction monitoring revealed the initial formation of PF_3_ (δ ^19^F −34.5 ppm, d, ^1^ *J*_PF_ = 1400.9 Hz, t = 1 h),^93,94^ which subsequently transforms into a mixture of P­(V) species (Figure 4 and SI Section S3.19). The ^19^F­{^1^H} NMR spectra showed two sets of resonances with distinct time-dependent integrals, demonstrating that they do not arise from a single compound but rather from at least two different Lewis base adducts of PF_5_ (Figure S24). The major species was assigned as PF_5_·MeCN, based on chemical shifts and ^1^ *J*_PF_/^2^ *J*_FF_ coupling constants of the four equatorial (eq.) fluorine atoms (δ ^19^F −64.9 ppm, ^1^ *J*_PF(eq.)_ = 755.6 Hz, ^2^ *J*_FF_ = 51.1 Hz) consistent with literature data.^95–100^ The minor signals are doublet of doublets. These resonances are presumably attributed to DDQ adducts of PF_5_ (PF_5_·DDQ, δ ^19^F −65.7 ppm, ^1^ *J*_PF(eq.)_ = 751.8 Hz, ^2^ *J*_FF_ = 49.7 Hz, and −65.5 ppm, ^1^ *J*_PF(eq.)_ = 752.4 Hz, ^2^ *J*_FF_ = 50.4 Hz). The observation of two sets of resonances may arise from different coordination modes or oxidation states of the quinone framework (DDQ or DDQ^2–^). KPF_6_ is slowly generated from these intermediates as the final product (δ ^19^F −73.2 ppm, ^1^ *J*_PF_ = 707.0).^101^

**Figure 4:**
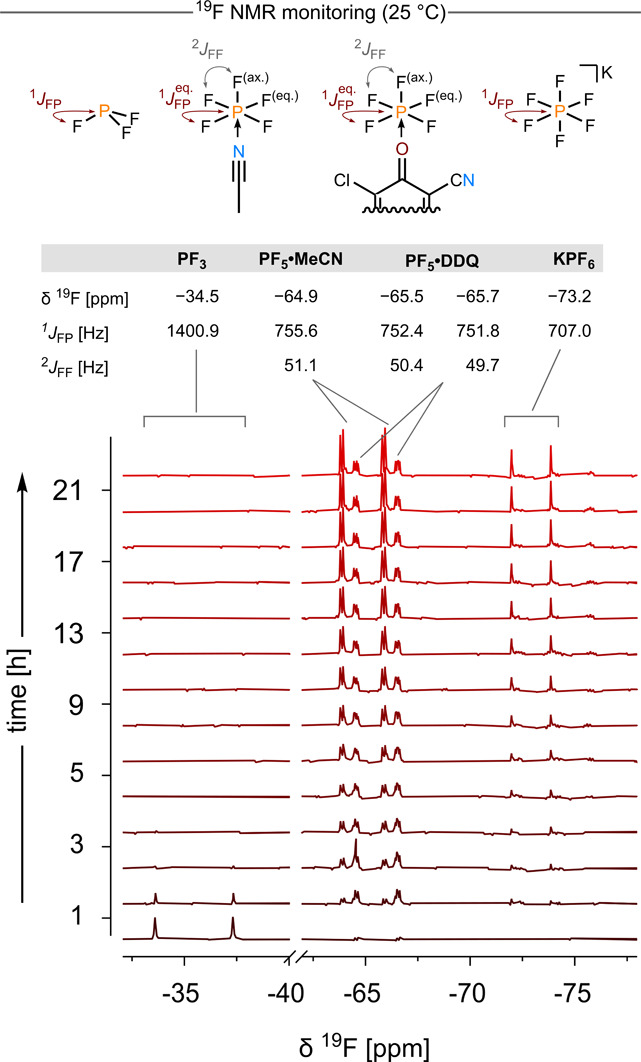
Section of the ^19^F­{^1^H} NMR reaction monitoring over 23 h at 25 °C (for conditions, see Figure 3a) showing the gradual formation of PF_3_, PF_5_·MeCN and KPF_6_).^96–98^ For details, see SI Section S3.19.

### Influence of Fluoride Solubility

Next, we assessed how solvents such as tetraethylene glycol (TEG) accelerate the reaction by enhancing the solubility of KF.^102–104^ ^31^P and ^19^F NMR spectroscopy of a reaction of P_4_, DDQ, and KF in MeCN/TEG (9:1 v:v) at 25 °C unexpectedly revealed the formation of a PF_5_ adduct K­[PF_5_·TEG] in 27% yield, containing a deprotonated TEG molecule coordinated to phosphorus (Figure 5a). The formation of K­[PF_5_·TEG] is evidenced by a doublet of pentets in the ^31^P­{^1^H} NMR spectrum (δ ^31^P −140.6 ppm, ^1^ *J*_PF(eq.)_ = 717.7 Hz, ^1^ *J*_PF(ax.)_ = 689.4 Hz) and two ^19^F­{^1^H} NMR resonances observed in a 4:1 integral ratio (Figure 5b, SI Section S3.5). The equatorial fluorine atoms give rise to a doublet of doublets (δ ^19^F^(eq.)^ −70.1 ppm, ^1^ *J*_PF(eq.)_ = 717.7 Hz, ^2^ *J*_FF_ = 41.5 Hz, 4F), while the axial (ax.) fluorine atom gives a doublet of pentets (δ ^19^F^(ax.)^ −66.5 ppm, ^1^ *J*_PF(ax.)_ = 689.4 Hz, ^2^ *J*_FF_ = 41.4 Hz, 1F). These resonances and coupling constants are similar to literature data for PF_5_OR^–^ complexes (e.g., R = Et: δ ^31^P = −141.8 ppm, ^1^ *J*_PF(eq.)_ = 722 Hz, ^1^ *J*_PF(ax.)_ = 675 Hz).^45–47^ In addition, the hydropentafluorophosphate anion (HPF_5_^–^) was detected (δ ^19^F^(eq.)^ −56.5 ppm, ^1^ *J*_PF_ = 818.1 Hz, ^2^ *J*_HF_ = 126.9 Hz, ^2^ *J*_FF_ = 42.4 Hz, 4F; δ ^19^F^(ax.)^ −67.5 ppm, ^1^ *J*_PF_ = 731.1 Hz, ^2^ *J*_FF_ = 42.4 Hz, 1F) as a side product (11%) alongside trace impurities of KPO_2_F_2_ and other phosphates (<6%, see SI Section 3.5 for details).^45,105,106^

Integration of the quantitative ^31^P­{^1^H} NMR spectra shows that the sum of the product integrals is significantly smaller than 100%, indicating the presence of additional NMR-silent products^92^ (Figure S47). This might be attributed to polymeric and insoluble phosphorus-containing byproducts. In DCM/TEG (9:1) at 25 °C, K­[PF_5_·TEG] was obtained in 75% spectroscopic yield without the formation of K­[HPF_5_] (Figures S49 and S50).

^19^F and ^1^H DOSY NMR experiments (see SI Section 3.21) gave additional evidence for the formation of K­[PF_5_·TEG]. The relative hydrodynamic volume of K­[PF_5_·TEG] (1,550 ± 44 Å^3^) corresponds to the sum of the volumes of KPF_6_ (237.9 ± 10 Å^3^) and TEG (1,335 ± 15 Å^3^). Electrospray ionization mass spectrometry (ESI-MS) of the reaction mixture (MeCN/TEG, 9:1, Table S15, entry 1) revealed a signal corresponding to K­[PF_5_·TEG] at *m*/*z* = 319.0619 (calcd: 319.0733, Figures S73 and S74).

The use of TEG as an additive affords a higher yield of fluorinated phosphorus products after 18 h (K­[PF_5_·TEG]: 75%, 25 °C) than in its absence (KPF_6_: 41%, 25 °C), demonstrating an increased reaction rate, likely due to a higher fluoride concentration. The formation of K­[PF_5_·TEG] appears to be a kinetic phenomenon, as increasing the reaction temperature to 65 °C leads to the formation of PF_6_^–^ (Table S15, Figures S51 and S52). Presumably, TEG’s chelation of fluoride impedes the final fluorination step (Figure 5a).

The product distribution is completely different when the reaction is performed in hot DCM (65 °C). In this case, PF_3_ (δ ^31^P 104.6 ppm, ^1^ *J*_PF_ = 1400.9 Hz, Figure S4) is formed in 13% yield (65 °C, Table S3) with P­(DDQH)_3_ (δ ^31^P 136.5 ppm, Figure S3) being a minor side product.^92^ The UV–Vis absorption spectra of DDQ and KF in DCM showed no change in absorbance, indicating that there is no reaction between DDQ and KF (Figure 6). This is likely due to the poor solubility of fluoride in DCM.^75^ In contrast, analogous experiments performed using MeCN revealed the characteristic formation of DDQ^·–^ (presumably via M­[DDQ·F], Figure 6 and Scheme 1b).^66,88^ These results indicate that P_4_ fluorination proceeds via distinct pathways in DCM and MeCN. In DCM, fluorination of P_4_ to PF_3_ proceeds slowly, presumably involving the phosphite P­(DDQH)_3_. Fluorination via similar DDQ-containing phosphonates has been described by Yang et al. (Scheme 1a).^49^ In MeCN, DDQ-fluoride complexes of the type K­[DDQ·F] are generated, which fluorinate P_4_ to KPF_6_.

This hypothesis is supported by investigations of the reaction between DDQ and KF using solvent mixtures of DCM and MeCN with TEG (DCM/TEG or MeCN/TEG 9:1 v:v, Figure 6c). Notably, the formation of DDQ^·–^ is observed by UV–Vis absorption spectroscopy in these mixtures. In contrast, in pure DCM, DDQ^·–^ is not detected by UV–Vis spectroscopy. The formation of DDQ^·–^ coincides with the formation of K­[PF_5_·TEG] in DCM/TEG and MeCN/TEG when P_4_ is present under similar conditions (Figure 5 and SI, Section S3.5).

To test whether DDQ mainly acts by solubilizing fluoride via σ-complex formation, we employed the strong oxidant Magic Blue in the presence of crypt-222 (7.5 equiv.) as fluoride-solubilizing agent. These conditions afforded KPF_6_ in 30% NMR yield (along with K­[HPF_5_], see Section 2.6, SI). Note that Magic Blue does not afford KPF_6_ in the absence of cryptand (Table S10). Further investigations are needed to elucidate the mechanism of the fluorine-transfer step.

**Figure 5:**
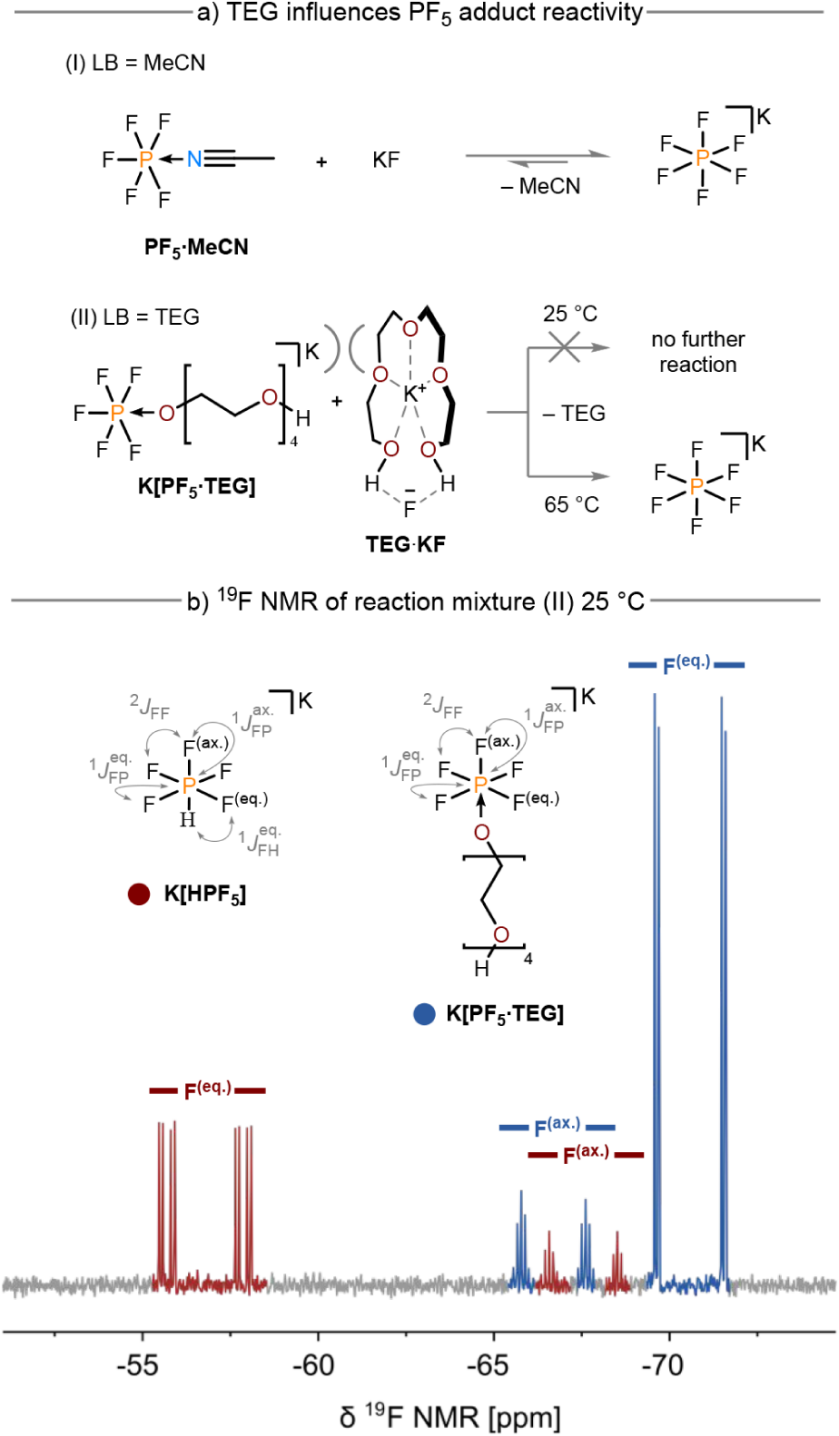
a) Rationalization for the preferential formation of the K­[PF_5_·TEG] adduct when TEG is present in the reaction mixture. b) Section of the ^19^F­{^1^H} NMR spectrum of the reaction mixture of P_4_, DDQ, and KF in MeCN/TEG (9:1) at 25 °C (Figures S47 and S48). ^19^F NMR chemical shifts and coupling constants for species observed under MeCN/TEG (9:1) conditions at 25 °C: K­[PF_5_·TEG]: δ −70.1 ppm (F^(eq.)^, dd, ^1^ *J*_PF_ = 717.7 Hz, ^2^ *J*_FF_ = 41.5 Hz, 4F); δ −66.5 ppm (F^(ax.)^, dp, ^1^ *J*_PF_ = 689.4 Hz, ^2^ *J*_FF_ = 41.4 Hz, 1F), K­[HPF_5_]: δ −56.5 ppm (F^(eq.)^, ddd, ^1^ *J*_PF_ = 818.1 Hz, ^2^ *J*_HF_ = 126.9 Hz, ^2^ *J*_FF_ = 42.4 Hz, 4F); δ −67.5 ppm (F^(ax.)^, dp ^1^ *J*_PF_ = 731.1 Hz, ^2^ *J*_FF_ = 42.4 Hz, 1F). For further details, see SI, Section 3.5.

### Summary of Reaction Steps

As shown in Scheme 3, the underlying reaction pathways can be divided into two distinct “fluoride-first” and “phosphorus-first” manifolds, depending on whether the reaction is performed in coordinating or noncoordinating solvent. In the coordinating solvent MeCN, the DDQ/M­[DDQ·F] mixture oxidizes and fluorinates P_4_ to yield PF_3_ and PF_5_·MeCN, as observed in the NMR monitoring studies (Figure 5), which ultimately react with fluoride to form MPF_6_ salts (Scheme 3, pathway a). In the presence of TEG, the reaction at 25 °C stops at K­[PF_5_·TEG], whereas the reaction under the same conditions at 65 °C proceeds fully to KPF_6_, as coordination of TEG to KF hinders further reactivity kinetically (Figure 5a). In the absence of a suitable redox-active fluoride acceptor such as P_4_, M­[DDQ·F] decomposes unproductively, partially generating DDQ^·–^ (Scheme 3, pathway c) and other unidentified fluorinated quinone species (Figure S67).

Quinone-fluoride complexes are not formed to a significant extent in DCM (Figure 6b). In this case, DDQ reacts directly with P_4_ to form the phosphite-like intermediate P­(DDQH)_3_ in traces (5%) among other NMR-silent phosphorus species, which remain active in the fluorination (Scheme 2). Reaction with fluoride in DCM subsequently generates PF_3_ (Scheme 3, pathway b, SI Section 3.14). P­(DDQH)_3_ was not detected in ^31^P­{^1^H} NMR monitoring studies in MeCN (Figures 4 and S68–72), suggesting that it does not contribute significantly to PF_6_^–^ formation, even though species related to P­(DDQH)_3_ have been proposed as intermediates in the fluorination of phosphonates.^49^

## Conclusions

This work reports the synthesis of PF_6_^–^ salts directly from P_4_ using commercial electron-deficient quinonoid oxidants and simple fluoride sources under mild conditions. To our knowledge, this is the first direct oxidative fluorination of P_4_, avoiding the use of chlorinated intermediates derived from Cl_2_ gas or Cl_2_-surrogates. Systematic variation of reaction parameters allowed the targeted generation of PF_3_, K­[PF_5_·TEG], and MPF_6_ salts (M = Li, Na, K, Cs, NMe_4_). Studies of model reaction sequences identified transient donor–acceptor interactions (M­[DDQ·F]) as key to increasing the effective fluoride concentration and promoting oxidative fluorination.^66^ Solvents with low fluoride solubility, such as DCM, redirect the reaction to form P­(DDQH)_3_ in traces, among other P-species undetectable by NMR, which produce PF_3_ through nucleophilic substitution. Adding TEG to MeCN or DCM improves KF solubility, allowing the formation of K­[PF_5_·TEG] while preventing further fluorination at room temperature. Detailed NMR and UV–Vis monitoring studies reveal PF_3_ and PF_5_·MeCN as intermediates en route to PF_6_^–^ and show that DDQ simultaneously acts as a single-electron oxidant and fluoride-ion shuttle.

**Figure 6:**
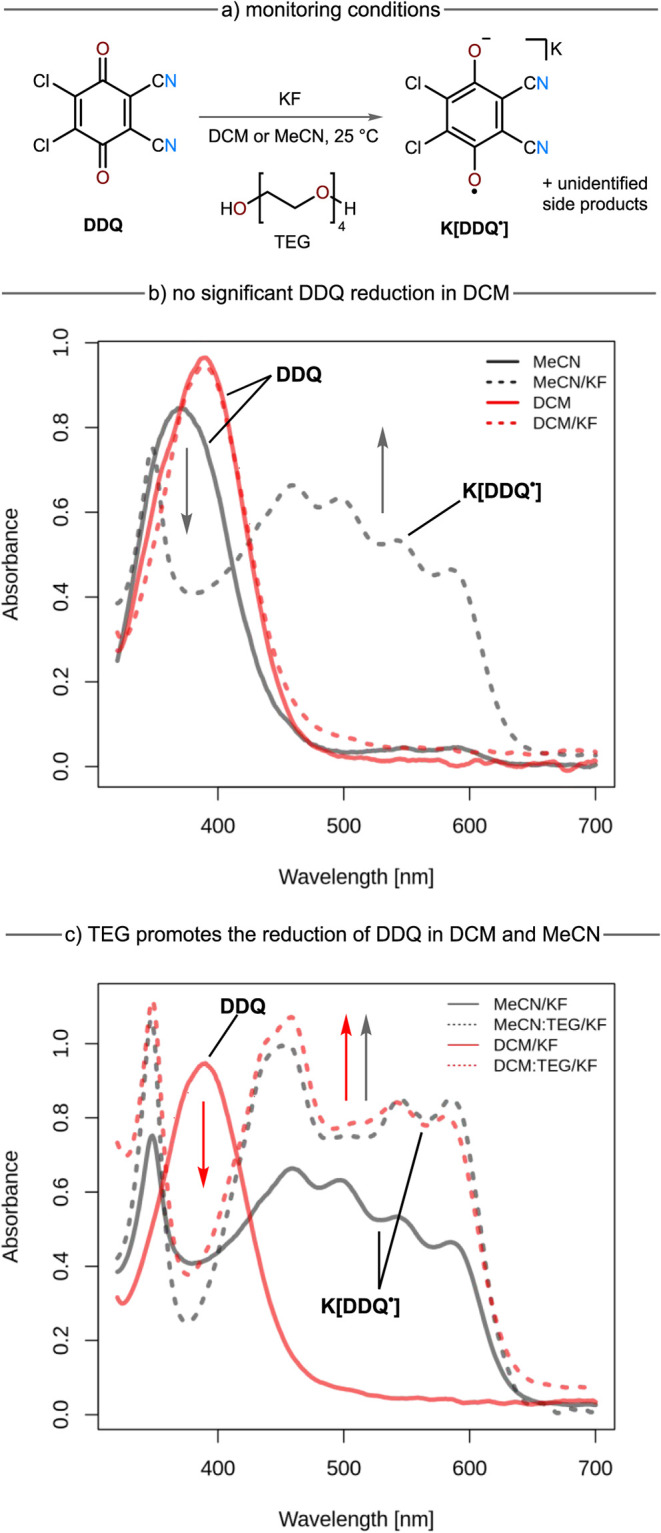
a) Schematic representation of the studied reaction. b) UV–Vis spectra of DDQ (bold lines) and DDQ with an excess of KF (dotted lines) in MeCN (black) and DCM (red), showing the generation of K­[DDQ^·^] in MeCN but not in DCM. c) UV–Vis spectra of DDQ in MeCN (black) and DCM (red) with an excess of KF added and in the presence of TEG, which enhances fluoride solubility and thus enables the reaction of DDQ and F^–^ in DCM. This highlights that fluoride solubility is a key factor in steering the reaction outcome. For details, see Section 3.18 in the SI.

**Scheme 3:**
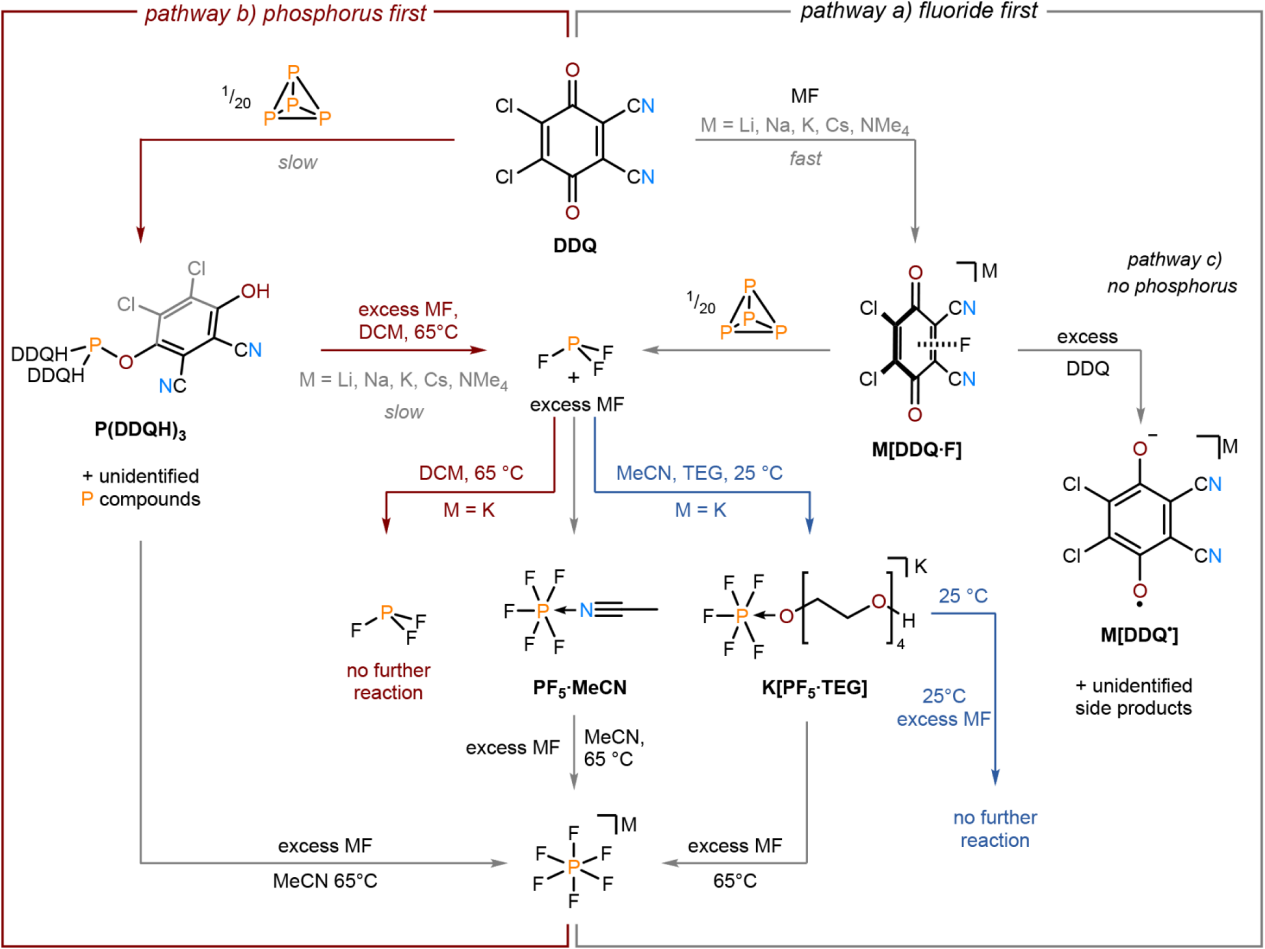
Proposed Reaction Steps Showing the Solvent-Dependent Reactivity. In Coordinating Solvents, DDQ Can Interact with MF and Efficiently Fluorinate P_4_ to KPF_6_ (Pathway a, Gray). When TEG Is Present in the Reaction Mixture, the Reaction Stops at the Penultimate Step (K­[PF_5_·TEG], Blue). In Noncoordinating Solvents, such as DCM, There Is No Significant Interaction between DDQ and MF, Thus Pathway b) (Red) Becomes Dominant and the Reaction Stops at PF_3_

Our results reveal a promising new avenue for converting P_4_ into valuable phosphorus fluorides via simple one-pot procedures. Additionally, we demonstrate the deliberate use of quinones for oxidative fluorination, broadening the synthetic scope of quinonoid oxidants in fluorine chemistry.

Future work in our laboratory will examine catalytic routes to PF_6_^–^ and PF_5_·LB species using the fluorination strategy outlined in this work, including the development of electrochemical methods. Furthermore, it seems likely that P_4_ activation by quinones can be used for the synthesis of other valuable phosphorus compounds via nucleophilic substitution. Additionally, transiently formed complexes such as K­[DDQ·F] could serve as versatile fluorination reagents in other synthetic contexts.

## Supplementary Material



## Notes

Methods, experimental procedures, reaction optimization, scale-up and product isolation, analytical data including NMR, UV–Vis and ESI-MS spectra DOSY NMR, and spectroelectrochemistry data (PDF)

## ◆

Istituto di Chimica dei Composti Organometallici, 50019 Sesto Fiorentino, Italy
