# Supplementary material for: Fluorination of White Phosphorus with Fluoride Salts and Quinone Oxidants
Source: J Am Chem Soc. 2026 Jul 23;148(30):31885–95. doi: 10.1021/jacs.6c04361 (PMC13450420; doi:10.1021/jacs.6c04361)
Supplement: Supplementary file 1 [file ja6c04361_si_001.pdf]

Supporting Information

Fluorination of White Phosphorus with Fluoride Salts and Quinone Oxidants

Benjamin Falge [a], Maximilian Philipp [b], Tobias Villing [a], Ruth M. Gschwind [b], Rafael E. Rodríguez-Lugo [a,†,*] and Robert Wolf [a,*]

Affiliations:

[a] Institute of Inorganic Chemistry, University of Regensburg, 93040 Regensburg, Germany.

[b] Institute of Organic Chemistry, University of Regensburg, 93040 Regensburg, Germany.

†Present Adress: Istituto di Chimica dei Composti Organometallici, 50019 Sesto Fiorentino, Italy,

*Corresponding authors: robert.wolf@ur.de, rafael.rodriguez@iccom.cnr.it

Contents

1. General Methods 4
   - 1.1. Reagents, Solvents and Other Materials 4
   - 1.2. Analytical Instrumentation 4
   - 1.3. Calibration of Quantitative $^{31}P\{^{1}H\}$ NMR Spectroscopy 7
2. Screening Experiments and Reaction Optimization 9
   - 2.1. General Procedure 9
   - 2.2. Solvent Screening 10
   - 2.3. Variation of Concentration and Temperature 13
   - 2.4. Screening of Fluoride Sources 14
   - 2.5. Quinone Screening 17
   - 2.6. Screening of Oxidizing Agents 19
3. Additional Synthetic Experiments and Spectroscopic Monitoring Studies 22
   - 3.1. Sensitivity to Water and Oxidation 22
   - 3.2. Reaction with Red Phosphorus 23
   - 3.3. Scale-up 24
   - 3.4. Isolation of KPF_6 24
   - 3.5. Effect of TEG as a Co-Solvent 26
   - 3.6. Spectroelectrochemistry of DDQ 28
   - 3.7. UV-Vis Studies on the Reactivity of DDQ Towards Fluoride 31
   - 3.8. UV-Vis Studies on the Reactivity of DDQ Towards P_4 33
   - 3.9. Synthesis of K[DDQ$^{\cdot}$] via Reduction of DDQ with KI 36
   - 3.10. Synthesis of $DDQH_2$ via Reduction of DDQ with $Na_2S_2O_4$ 37
   - 3.11. Synthesis of $P(DDQH)_3$ from PCl_3 and $DDQH_2$ 38
   - 3.12. Comments on the Proposed Structure of $P(DDQH)_3$ 39
   - 3.13. Studies on the Formation of $P(DDQH)_3$ 41
   - 3.14. Studies on Reactivity of P_4/DDQ Reaction Mixture with KF 42
   - 3.15. Reaction of K[DDQ$^{\cdot}$] with P_4 43
   - 3.16. Variation of the DDQ/P_4 Ratio 44
   - 3.17. UV-Vis Studies on the Solvent-Dependent Reactivity of DDQ 45
   - 3.18. In-situ UV-Vis Reaction Monitoring 48
   - 3.19. In situ NMR Reaction Monitoring Experiments 50
   - 3.20. Diffusion Ordered Spectroscopy (DOSY) Studies of K[PF_5·TEG] 52
4. NMR Spectra 58
   - 4.1. Screening of Fluoride Sources 58
   - 4.2. Screening of Oxidizing Agents 60
   - 4.3. Sensitivity to Water and Oxidation 64

4.4. Reaction with Red Phosphorus 66
4.5. Reaction Scale-Up 69
4.6. Isolation of KPF_6 72
4.7. Effect of TEG as a Co-Solvent 77
4.8. Synthesis of $DDQH_2$ 83
4.9. Synthesis of $P(DDQH)_3$ from PCl_3 and $DDQH_2$ 86
4.10. Studies on the Formation of $P(DDQH)_3$ 89
4.11. Studies on Reactivity of P_4/DDQ Reaction Mixture with KF 92
4.12. Reaction of $K[DDQ^{\cdot}]$ with P_4 94
4.13. Reaction of DDQ with KF 97
4.14. In situ NMR Reaction Monitoring Experiments 98
5. ESI-MS 103
6. References 105

1. General Methods

1.1. Reagents, Solvents and Other Materials

All reactions and manipulations were performed under an N_2 atmosphere (< 0.1 ppm O_2, H_2O) through use of an MBraun Unilab and Glovebox Systemtechnik glovebox. All glassware was oven-dried (180 °C) overnight prior to use or dried under vacuum using a heat gun three times. Benzene was dried over Na and stored over molecular sieves (3 Å). Tetraethylene glycol (TEG) was distilled successively over Ca and K and stored over molecular sieves (3 Å). Acetonitrile (MeCN), 1,2-dimethoxyethane (DME) and dimethylformamide (DMF) were distilled from CaH_2 and stored over molecular sieves (3 Å). Et_2O, tetrahydrofuran (THF), dichloromethane (DCM) and toluene were purified using an MBraun SPS-800 system and stored over molecular sieves (3 Å) at least overnight prior to use. Benzene-d_6 was distilled from potassium and stored over molecular sieves (3 Å). Acetonitrile-d_3, THF-d_8 and DCM-d_2 were stored over molecular sieves (3 Å). All other chemicals were purchased from major suppliers (Merck, TCI, VWR). Liquids (except $NEt_3{\cdot}3(HF)$) were purified by Kugelrohr distillation and freeze-pump-thaw degassed three times before use; P_4 and Ph_3PO (TPPO) were purified by sublimation; KF was dried under vacuum at 120 °C for three days; all other reagents were used as received.

Caution. *P_4 is highly toxic and pyrophoric and must be handled strictly under an inert atmosphere. PF_5 and related adducts are highly toxic and moisture-sensitive, hydrolyzing in air to release corrosive and toxic HF. Fluorophosphates and their esters are extremely toxic. DDQ can hydrolyze in the presence of water to release highly poisonous HCN. All manipulations must be conducted under inert atmosphere or in a well-ventilated fume hood while wearing appropriate protective laboratory clothing and safety equipment.*

1.2. Analytical Instrumentation

Product analysis was conducted by ^{1}H NMR (500.00 or 400.00 MHz), ^{2}H NMR (61.42 MHz), ^{13}C NMR (125.78 MHz), ^{11}B NMR (160.42 or 128.38 MHz), ^{19}F NMR (470.38 or 376.48 MHz) and ^{31}P NMR (202.40 or 161.97 MHz) spectroscopy (Bruker Avance neo 500 or Bruker Avance III 400). Chemical shifts (δ) are given in ppm. Multiplicity is described either as s = singlet, d = doublet, t = triplet, q = quartet, p. = pentet, sex. = sextet, sept. = septet, m = multiplet). ^{1}H NMR and ^{13}C NMR spectra were referenced against tetramethylsilane (TMS, δ 0.00 ppm) and were calibrated internally to the residual

solvent signals. ^{19}F NMR spectra are referenced against trichlorofluoromethane ($CFCl_3$, δ 0.0 ppm) and ^{31}P NMR spectra against H_3PO_4 (w = 85% in H_2O, δ 0.0 ppm). ^{2}H NMR spectra were recorded without reference-lock. Quantitative $^{31}P\{^{1}H\}$ NMR spectroscopy, as described in section S2, was used to determine product yield and substrate conversion unless noted otherwise.

Cyclic voltammetry was performed in a borosilicate glass single-compartment cell inside a nitrogen-filled glovebox using a CH Instruments CHI600E potentiostat. A platinum disc working electrode (1.6 mm diameter) was polished with 0.1 µm diamond paste, rinsed with acetone and water, then polished with 0.05 µm alumina paste, rinsed, and gently dried with a hot-air blower. The platinum wire counter electrode was rinsed with water and acetone before use and then annealed with a propane torch until no orange flame tail was observed, indicating complete combustion of organic residues. The silver wire pseudo-reference electrode was polished sequentially with 7000- and 10000-grit sandpaper, wiped with a lint-free tissue to remove residual solids, and rinsed with water and acetone. Residual solvent on the electrodes was removed under vacuum in the glovebox antechamber. The working and counter electrodes shared the same compartment, and the applied voltage was not corrected for IR drop. The supporting electrolyte, $[^{n}Bu_4N]PF_6$ ($TBAPF_6$), was dried under vacuum (10^{-3} mbar) at 110 °C for three days. All redox potentials are referenced to the half wave potential of the ferrocene/ferrocenium (Fc/Fc^+) redox couple. CVs were recorded at a scan rate of 100 mV/s.

UV-Vis spectra were recorded inside a nitrogen-filled glovebox on an Ocean Optics Flame Spectrometer with a DH-2000 UV-NIR halogen-deuterium lamp light source coupled with UV/SR-Vis high-OH, solarization-resistant optical fibers (50 µm core diameter, 0.22 numerical aperture, 25.4° full angle, 2 m length). Measurements were performed in Hellma QS quartz glass cuvettes (d = 10.0 mm).

Spectroelectrochemical measurements were performed using a Varian Cary 100 UV-Vis spectrophotometer coupled to a MetrOhm Autolab M204 potentiostat. Experiments were performed in MeCN (Merck, HPLC grade), degassed with Argon, containing 0.1 M $TBAPF_6$ as supporting electrolyte. A room temperature optically transparent thin-layer electrochemical (OTTLE) cell (Frantisek Hartl, University of Reading, UK) was employed, featuring a path length of approx. 0.02 cm, Pt minigrid working and counter electrodes (32 wires/cm^2), an Ag microwire pseudo-reference electrode, and optically

transparent CaF_2 windows. Prior to spectroelectrochemical measurements, CV (see above) was performed to determine the approximate positions of the redox processes. Subsequently, the potential window corresponding to the relevant electrode processes was stepped in 0.05 V increments, with a 10 s holding time at each step. The UV-Vis spectrum was recorded at the end of each step.

For photochemical experiments, a TAK120 AC (air-cooled) photoreactor from HK-Testsysteme GmbH (456 nm, 7W) with integrated temperature control and timer was used. The emission spectrum of the light source is depicted in previous work.[1]

Electrospray ionization mass spectrometry (ESI-MS) was performed on an Agilent 6540 UHD Q-TOF mass spectrometer, with the sample introduced offline, at the University of Regensburg.

Elemental analysis (C, H, N) was performed at the University of Regensburg.

Elemental analysis (C, H, N, K, P, F) was carried out by the Mikroanalytisches Laboratorium Kolbe, Oberhausen, Germany. Data was processed with JASPER v2.0 - JavaScript Percentage Elemental Results Calculator (University of York, https://www.yorku.ca/pgpotvin/public/Jasper/jasper2.htm).

Diffusion ordered NMR spectroscopy (DOSY) experiments were performed using a Bruker® Avance III-HD 600 MHz operating at 600.25 MHz for protons and 564.55 MHz for fluorine, equipped with a TBI 5 mm $^{1}H/^{19}F$-BB probe head with a BVT unit and with pulsed gradient units, capable of producing magnetic field pulsed gradients in the z-direction. The measurements were performed at 298 K. The temperature was certified by internal NMR calibration samples from Bruker®. NMR data were processed, evaluated, and plotted using TopSpin 3.2. Further analysis of the measurements was performed with Microsoft Excel and Spyder (Python 3.11).

1.3. Calibration of Quantitative $^{31}P\{^{1}H\}$ NMR Spectroscopy

NMR tubes were charged with varying amounts of P_4 stock solution (0.10 M in benzene) and KPF_6 stock solution (0.40 M in MeCN), as shown in Table S1. A fixed volume of the internal standard TPPO stock solution (100 μL, 0.10 M in MeCN) was added. Subsequently, $^{31}P\{^{1}H\}$ NMR spectra were recorded for all samples at 298 K, 1 atm, using 256 scans, a relaxation delay of 2.0 s, a receiver gain of 208.7 and a pulse width of 15.5 μs. All stock solutions were prepared in analytical flasks to minimize volume errors.

Figure S1 shows the normalized integrals against the mole fraction x_i of KPF_6 or P_4 in the different samples. The relationship between x_i and the integrals was fitted using the equation $f(x_i) = R_f \cdot x_i$, i.e., a linear fit constrained to pass through the origin (0,0), corresponding to the proportionality between integral and mole fraction (Pearson's $R^2 > 0.99$). The resulting response factors $R_f(KPF_6) = 1.97506$ and $R_f(P_4) = 1.70435$ were used for all subsequent calculations of yield and conversion unless noted otherwise. The integrals of P_4 (δ −522.3 ppm, s) and KPF_6 (δ −142.9 ppm, sept, $^{1}J_{PF} = 707.0$ Hz)[2] in the $^{31}P\{^{1}H\}$ NMR spectra were normalized to the TPPO signal (δ 27.4 ppm, s). For an example spectrum for KPF_6 quantification, see Figure S40.

Table S1. Samples prepared for $^{31}P\{^{1}H\}$ NMR calibration of P_4 and KPF_6 to the internal standard TPPO.

Entry	$x_f(P_4)$ [%]	$x_f(KPF_6)$ [%]	$V(P_4)$ [μL]	$V(KPF_6)$ [μL]
1	95	5	95.0	5.0
2	75	25	75.0	25.0
3	50	50	50.0	50.0
4	5	95	5.0	95.0

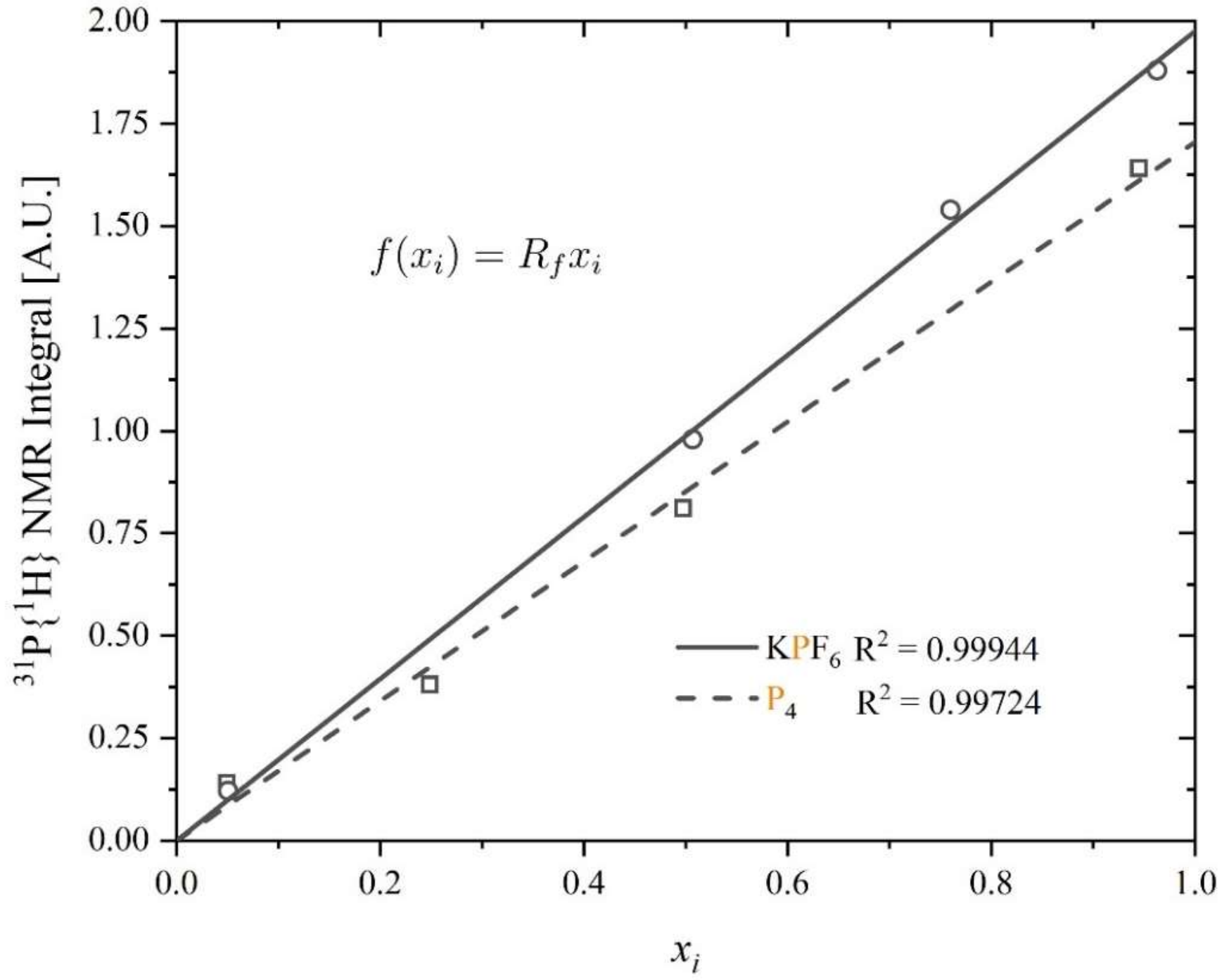

Figure S1. Calibration for quantitative $^{31}P\{^1H\}$ NMR spectroscopic analysis of KPF_6 and P_4. Normalized integrals of KPF_6 (bold line) and P_4 (dashed line) against their mole fraction x_i, showing a linear fit with the respective Pearson's R^2 correlation coefficient. Response factors (R_f) obtained for the analytes: R_f (KPF_6) = 1.97506 and $R_f(P_4)$ = 1.70435.

2. Screening Experiments and Reaction Optimization

2.1. General Procedure

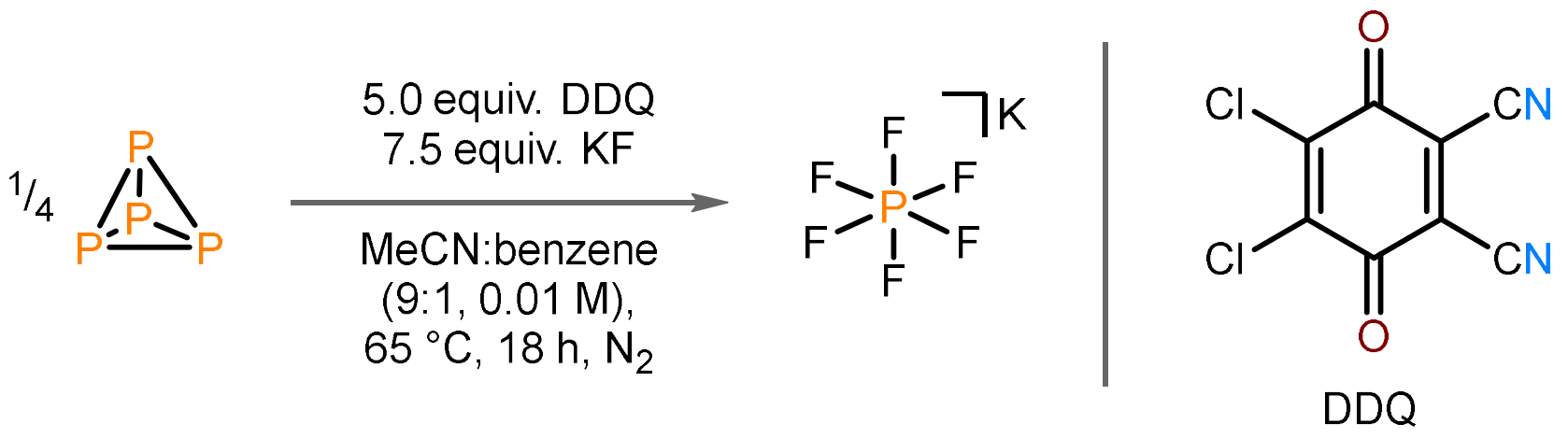

In a glovebox, a Schlenk-tube was charged with DDQ (45.5 mg, 200 µmol, 5.00 equiv.), KF (17.4 mg, 300 µmol, 7.50 equiv.), P_4 stock solution (0.1 M in benzene, 100 µL, 1.24 mg, 10 µmmol, 0.25 equiv.), TPPO stock solution (0.1 M in MeCN 100 µL, 10 µmol, 0.25 equiv.), and MeCN (0.8 mL). The reaction mixture was heated at 65 °C for 18 h. Conversion and yield were determined by quantitative $^{31}P\{^1H\}$ NMR spectroscopy, as described in section S1.3.

Note: Control experiments showed that adding the TPPO internal standard, either before or after the reaction, yielded identical results. Therefore, the TPPO stock solution was routinely added prior to heating.

2.2. Solvent Screening

Reactions were carried out according to the general procedure, with the solvent (1.0 mL) varied as listed in Table S2.

Table S2. Solvent screening experiments for the DDQ-mediated fluorination of P_4, including reaction conditions, conversion of P_4, yield of KPF_6, phosphorus molar balance, and the corresponding ^{19}F and ^{31}P NMR resonances and coupling constants of KPF_6. Optimal conditions are highlighted in grey.

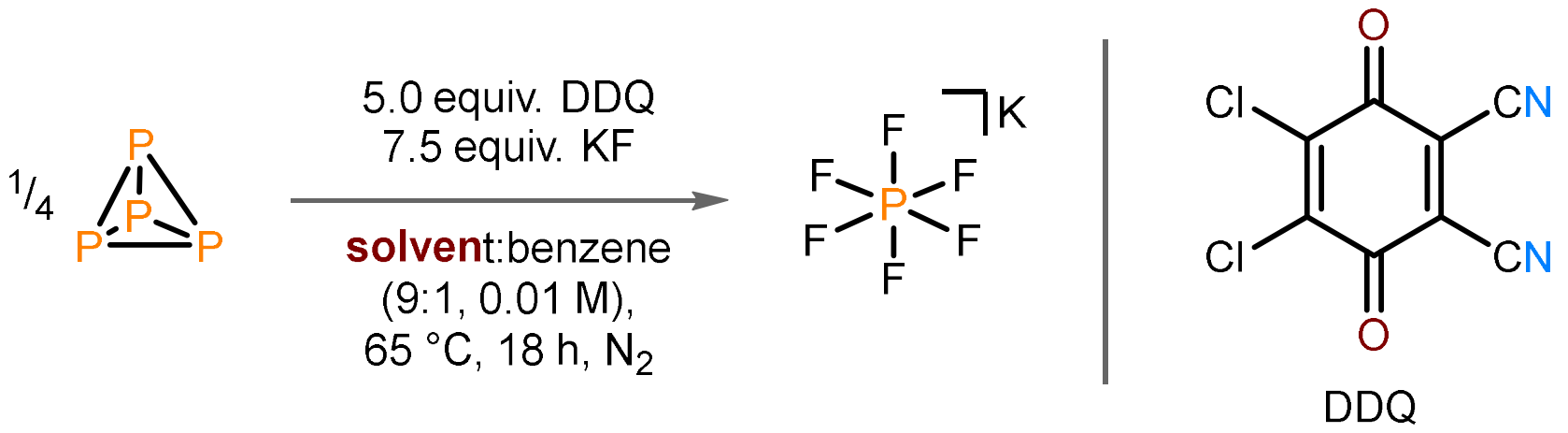

Entry	Solvent	Conversion P_4 [%][a]	Yield KPF_6 [%][a]	^{31}P δ KPF_6 [ppm][b]	^{19}F δ KPF_6 [ppm][c]	$\|^1J_{PF}\|$ (KPF_6) [Hz]
1	MeCN	>99	95	– 143.2	– 72.3	706.7
2[d]	EtOH	68	n.d.	n.d.	n.d.	n.d.
3	DMF	>99	63	– 143.5	– 72.2	710.1
4	acetone	>99	27	– 142.8	– 72.1	709.8
5	THF	>99	22	– 143.3	– 73.5	709.4
6[e]	$CHCl_3$	70	n.d.	n.d.	n.d.	n.d.
7[f]	DCM	97	n.d.	n.d.	n.d.	n.d.
8	Et_2O	92	n.d.	n.d.	n.d.	n.d.
9	toluene	84	n.d.	n.d.	n.d.	n.d.

[a] Yield of KPF_6 and conversion of P_4 was determined by quantitative $^{31}P\{^1H\}$ NMR spectroscopy using TPPO as internal standard (see section 1.3). [b] multiplicity: sept. [c] multiplicity: d. [d] $OP(OEt)_3$ was identified as the major product by ^{31}P NMR spectroscopy (δ – 0.5 ppm, sept. $^3J_{PH}$ = 8.0 Hz)[3]. [e] phosphoric acid/phosphate were identified as the major product by $^{31}P\{^1H\}$ NMR spectroscopy (δ – 0.4 ppm, s). [f] PF_3 was identified as the major product by $^{31}P\{^1H\}$ NMR spectroscopy (δ 104.6 ppm, q, $^1J_{PF}$ = 1400.9 Hz)[2,4,5].

Using EtOH as the solvent resulted in the formation of $OP(OEt)_3$, which was identified by a septet (δ – 0.5 ppm , $^3J_{PH}$ = 8.0 Hz, sept., Figure S2) in the proton-coupled ^{31}P NMR spectrum.[3,6] As a result, EtOH is unsuitable as a solvent for P_4 fluorination despite its higher fluoride solubility.

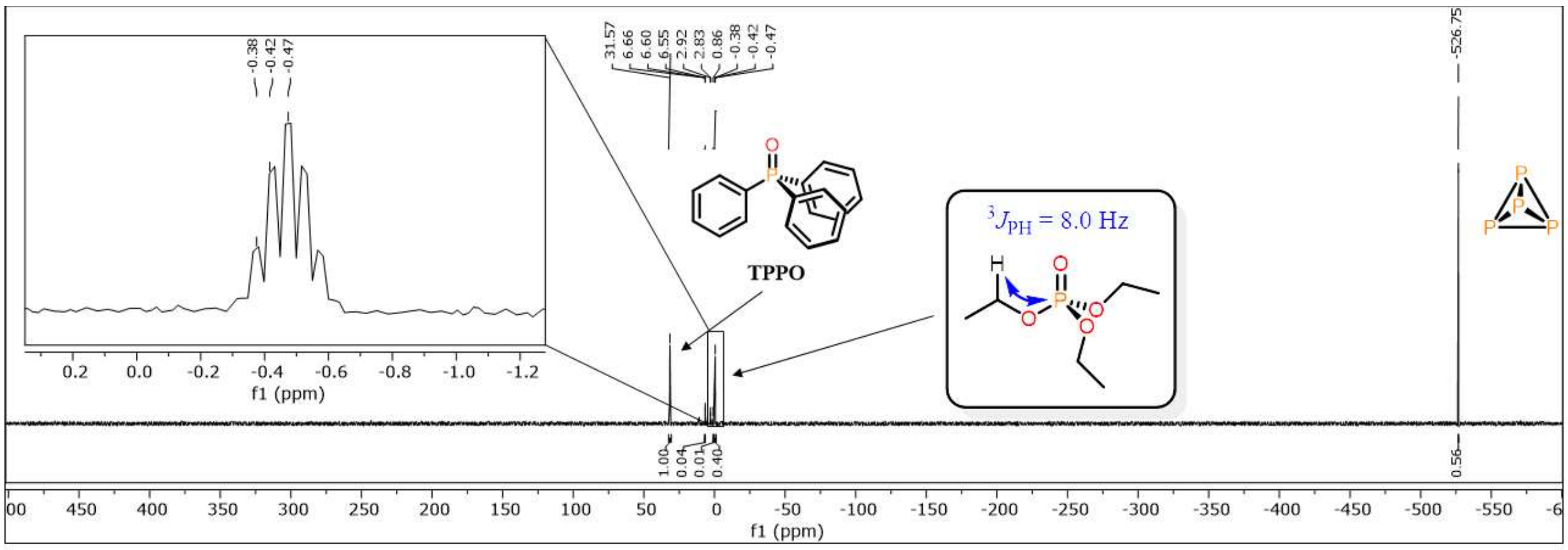

Figure S2. ^{31}P NMR (162.04 MHz, EtOH, C_6D_6) spectrum for the reaction in Table S2, entry 2, showing $OP(OEt)_3$ as the major product (δ –0.5 ppm, sept, $^3J_{PH}$ = 8.0 Hz). The inset highlights the septet splitting pattern arising from $^3J_{PH}$ coupling. Spectroscopic yield of $O{=}P(OEt)_3$: 20 %.

The reaction in DCM (Table S2. , entry 7) gave no KPF_6 but PF_3 (δ 104.6 ppm, q, $^1J_{PF}$ = 1400.9 Hz)[2,4,5] in 13% yield (89% conversion of P_4, Figure S3). An unidentified singlet signal at δ 136.5 ppm can presumably be assigned to the phosphite-like species $P(DDQH)_3$, due to a similar chemical shift observed in other phosphites, e.g. δ($P(OEt)_3$) 139.2, ppm and δ($P(OPh)_3$) 128.1 ppm.[3,7] For details on the proposed species $P(DDQH)_3$, see sections S3.11–S3.13.

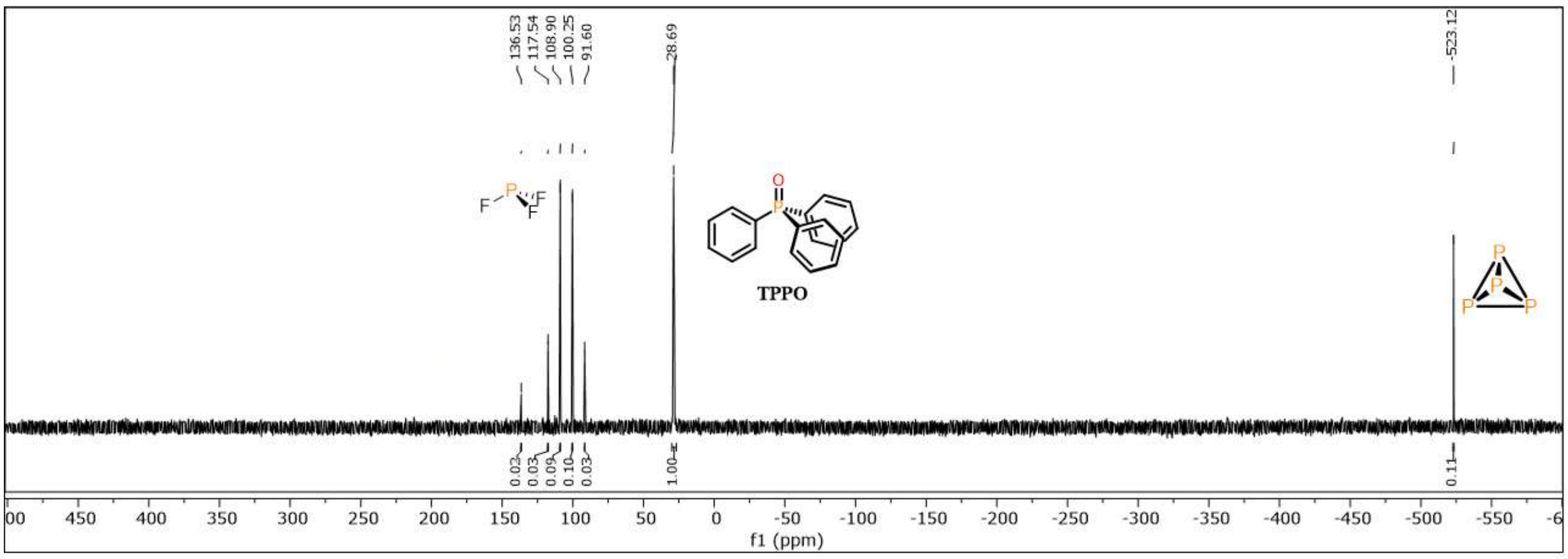

Figure S3. $^{31}P\{^1H\}$ NMR (162.04 MHz, DCM, C_6D_6) spectrum corresponding to the reaction presented in Table S2. , entry 7, showing PF_3 as the major product (δ 104.6 ppm, q, $^1J_{PF}$ = 1400.9 Hz)[2,4,5] and the proposed intermediate $P(DDQH)_3$ as minor signal (δ 136.5 ppm). Estimated yield of dissolved PF_3 gas: 13%.

The reaction in $CHCl_3$ (Table S2, entry 6) gave H_3PO_4 as the major product (δ ^{31}P – 0.4 ppm, s, 32 %), possibly due to hydrolysis. An unknown species containing a PF_2-fragment (δ ^{31}P 113.3 ppm, t, $^1J_{PF}$ = 1288.3Hz, 7 %) was observed as a minor product. The chlorine atom in the fluorinated side product most likely originates from fluoride-induced chloride substitution of chloroform.[8]

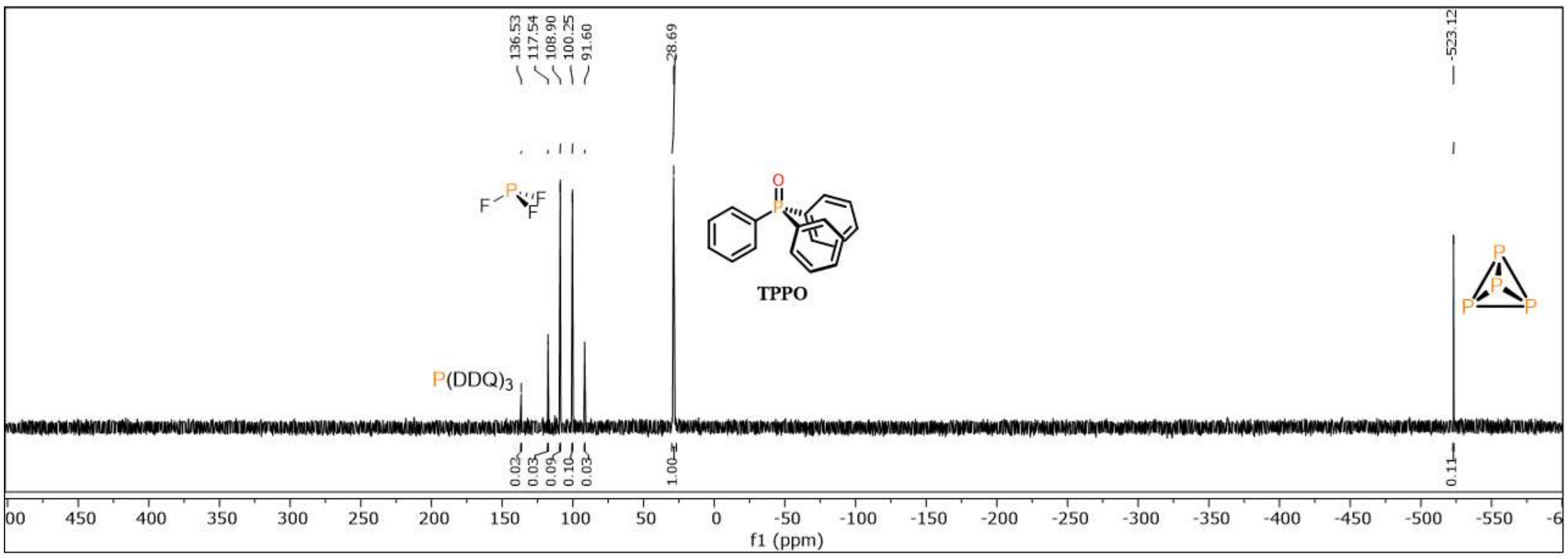

Figure S4. $^{31}P\{^1H\}$ NMR (162.04 MHz, $CHCl_3$, C_6D_6): Reaction in $CHCl_3$ (Table S2. , entry 6) showing phosphoric acid/phosphate as major product (δ – 0.4 ppm, s, 32 %) and a minor unknown product containing a PF_2-fragemnt (δ 113.3 ppm, t, $^1J_{PF}$ = 1288.3 Hz, 7 %).

2.3. Variation of Concentration and Temperature

Reactions were carried out according to the general procedure, with variation of concentration and temperature as listed in Tables S3 and S4.

Table S3. Variation of reaction concentration by changing the amount of MeCN, referenced to P_4 as the limiting reagent. Optimal conditions highlighted in grey.

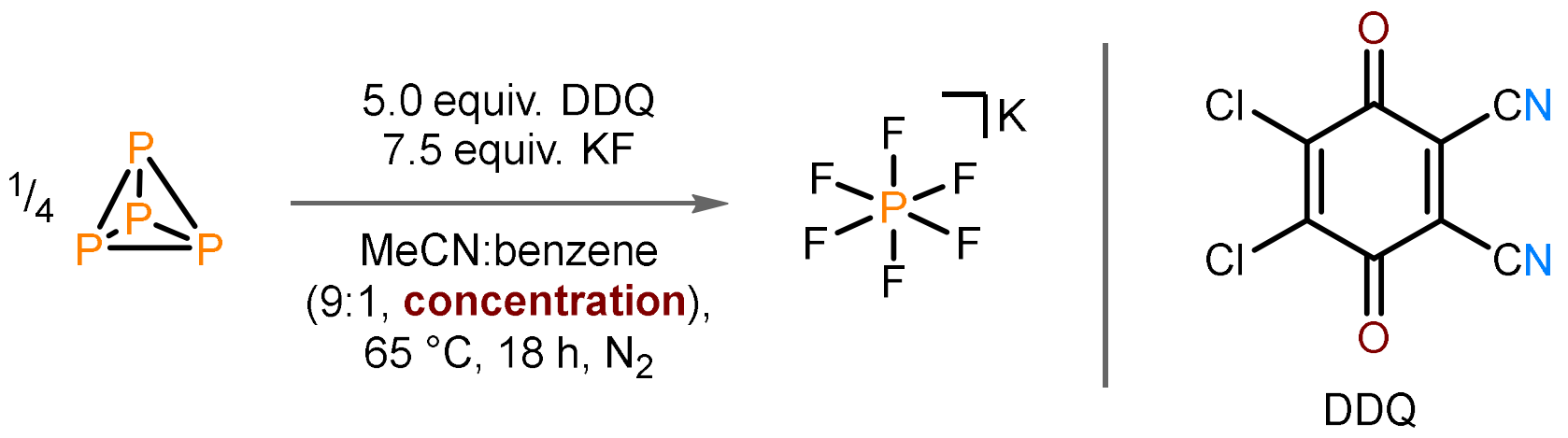

Entry	V(total) [mL]	c (P_4) [mM]	Yield KPF_6 [%]	Conversion P_4 [%]
1	0.6	17.0	70	>99
2	0.9	11.0	90	>99
3	1.0	10.0	95	>99
4[a]	2.2	4.5	25	50

[a] Solvent volume comprised of 1.1 ml MeCN, 1.0 ml THF and 0.1 mL benzene.

Table S4. Results of temperature screening for the DDQ-mediated fluorination of P_4 in MeCN. Average yield over two runs, with the corresponding standard deviations. Optimal conditions highlighted in grey.

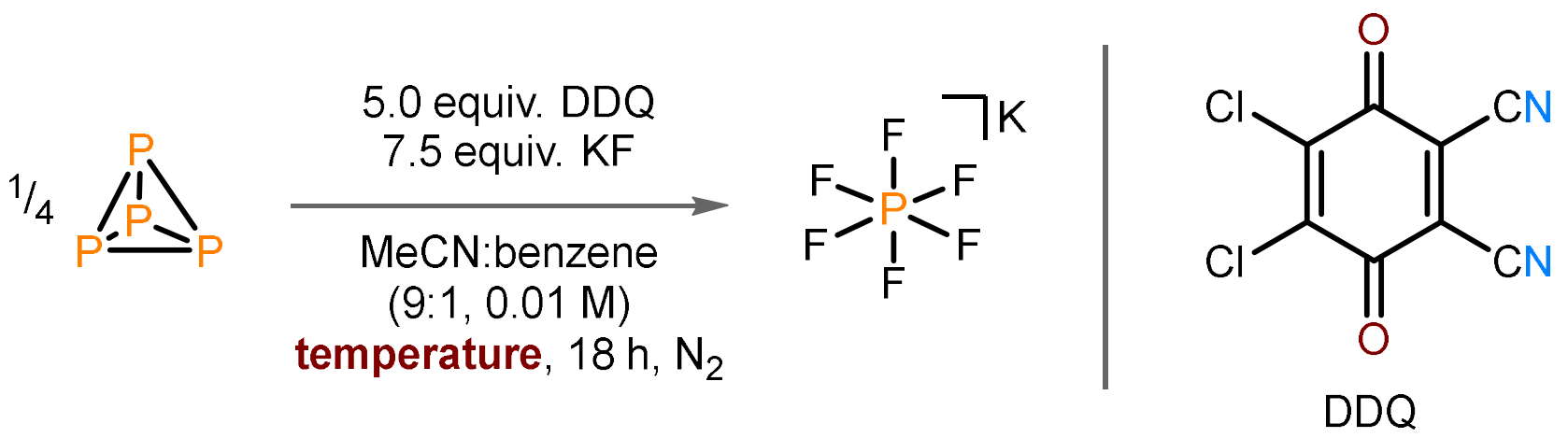

Entry	T [°C]	Yield KPF_6 [%]		Average yield KPF_6±sd [%]
		Run 1	Run 2	
1	25	41	42	41.5 ± 0.7
2	55	95	90	92.5 ± 3.5
3	65	96	95	95.5 ± 0.7
4	75	95	90	92.5 ± 3.5

2.4. Screening of Fluoride Sources

Reactions were carried out according to the general procedure, with the fluoride source (0.3 mmol, 7.5 equiv.) varied as listed in Table S5.

Table S5. Screening of the fluoride source with conversion of P_4 and yield of the corresponding hexafluorophosphate salt. Optimal conditions highlighted in grey.

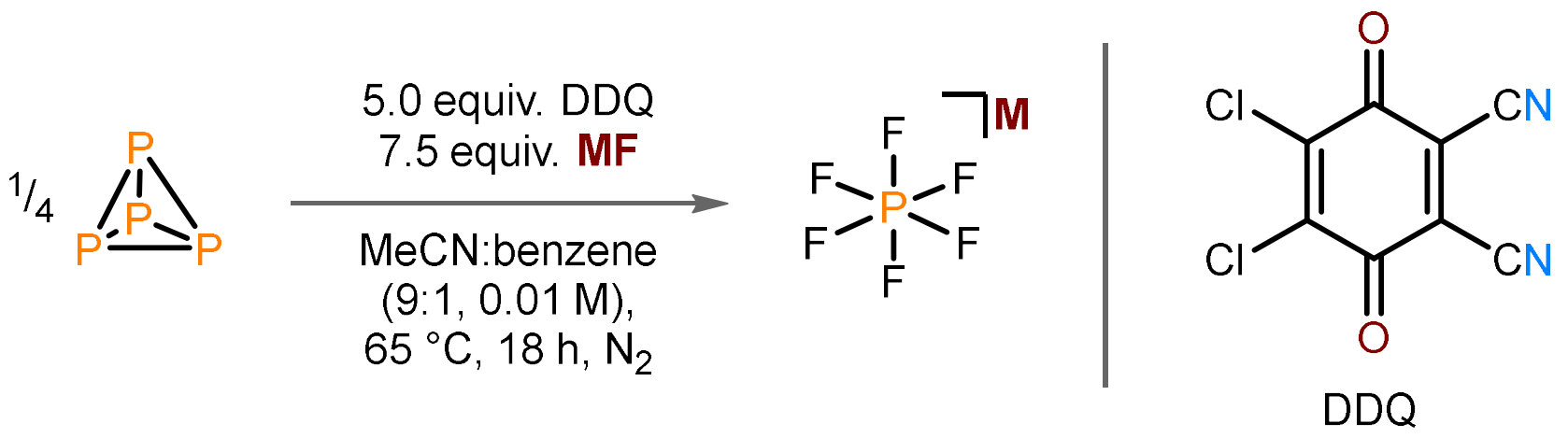

Entry	MF and conditions	n [mmol]/ m [mg] / V [µL]	Conversion P_4 [%]	Yield MPF_6 [%]
1	LiF	0.3/ 7.8	>99	35
2	NaF	0.3/ 12.6	>99	57
3	KF	0.3/ 17.4	>99	95
4	CsF	0.3/ 45.6	>99	47
5	CaF_2	0.3/ 23.4	>99	n.d.
6	NH_4F	0.3/ 11.1	>99	68
7	NMe_4F	0.3/ 27.9	>99	92
8	$NEt_3{\cdot}3(HF)$	0.3/ 48.4/ 48.9	>99	27
9	KHF_2	0.3/ 23.4	>99	n.d.
10	SF and KF	(SF) 0.3/ 106.3 ;(KF) 0.3/ 17.4	>99	50
11	SF, KF and no DDQ	(SF) 0.3/ 106.3; (KF)0.3/ 17.4	>99	92
12	$NaBF_4$	0.3/ 32.9	>99	73
13[a]	LiF, crypt-222	0.3/ 7.8; 0.15, 56.4	81	trace

[a] Reaction was performed in MeCN-d_3:C_6D_6 (9:1).

Hydrolysis under specific conditions during the DDQ-mediated fluorination of P_4 was evident from the formation of fluorophosphate species and fluorinated boron- or silicon-containing byproducts, as revealed by multinuclear NMR spectroscopy. In the ^{31}P NMR spectrum of the reaction using $NEt_3{\cdot}3(HF)$ as fluoride source (Table S6, entry 8), the species $PO_2F_2^-$ was observed as the major product (δ ^{31}P – 17.9 ppm, t, $^1J_{PF}$ = 957.7 Hz) in 57% yield (Figure S5). The corresponding ^{19}F{^{1}H} NMR spectrum displayed the expected resonance for $PO_2F_2^-$ (δ – 82.9 ppm, d, $^1J_{PF}$ = 957.7 Hz)[2,6], along with additional signals assignable to BF_4^- and SiF_6^{2-} (Figure S6). The BF_4^- anion appeared as an isotope mixture of $^{10}BF_4^-$ (δ – 151.9 ppm, s) and $^{11}BF_4^-$ (δ – 151.8 ppm, s), while SiF_6^{2-} resonated at δ – 136.8 ppm as a broad signal.[8,9]

Note: Ref. 6 employs a non-standard chemical shift convention. In that work, positive ^{19}F NMR values indicate upfield shifts; ^{19}F NMR resonances were referenced to $LiPF_6$ at 65.00 ppm, and ^{31}P NMR spectra were referenced to $LiPF_6$ at – 145.0 ppm. In our measurements, $LiPF_6$ was observed at δ ^{19}F – 72.2 ppm and δ ^{31}P – 142.9 ppm. Therefore, chemical shift values from Ref. 9 were adjusted by reversing their signs and then subtracting 7.2 ppm for ^{19}F and 2.1 ppm for ^{31}P, aligning them with the referencing standards outlined in the general information section S1.2

Note: Ref. 2 employs the same reference compounds for ^{19}F and ^{31}P NMR as this study. However, upfield shifts are represented by positive values. To facilitate comparison, the signs of the shifts reported in Ref. 10 were inverted.

Table S6. Fluoride source screening for the DDQ-mediated fluorination of P_4 in MeCN, including observed ^{31}P and ^{19}F NMR resonances and coupling constants, for MPF_6 and MPO_2F_2. Optimal conditions highlighted in grey.

Entry	F atom source MF	Yield MPF_6 [%]	Yield MPO_2F_2 [%]	MPF_6			MPO_2F_2		
				δ ^{31}P [ppm]	δ ^{19}F [ppm]	$\|^1J_{PF}\|$ [Hz]	δ ^{31}P [ppm]	δ ^{19}F [ppm]	$\|^1J_{PF}\|$ [Hz]
1[a]	LiF	35	7	– 143.2	– 72.1	707.0	– 16.5	– 83.9	929.1
2[a]	NaF	57	33	– 143.3	– 72.1	707.3	– 16.7	– 82.6	951.4
3	KF	95	n.d.	– 143.3	– 72.3	707.8	n.d.	n.d.	n.d.
4[a]	CsF	47	trace	– 143.2	– 71.7	707.2	n.d.	n.d.	n.d.
5	CaF_2	n.d.	n.d.	n.d.	n.d.	n.d.	n.d.	n.d.	n.d.
6[a]	NH_4F	68	4	– 143.5	– 72.5	707.5	– 16.3	– 82.9	944.7
7[a]	NMe_4F	92	6	– 143.4	– 72.2	707.4	– 14.6	– 81.5	949.0
8[a]	$NEt_3·3(HF)$	27	57	– 143.5	– 72.3	711.3	– 17.9	– 82.9	957.7
9[b]	KHF_2	n.d.	n.d.	n.d.	n.d.	n.d.	n.d.	n.d.	n.d.
10	SF, KF	54	12	– 143.9	– 72.4	708.1	– 16.4	– 83.0	955.5
11	SF, KF, no DDQ	92	n.d.	– 150.5	– 72.6	707.9	n.d.	n.d.	n.d.
12[b]	KBF_4	73	n.d.	– 144.1	– 73.1	706.7	n.d.	n.d.	n.d.
13[c]	LiF, crypt-222	traced	n.d.	– 144.1	– 71.5	706.7	n.d.	n.d.	n.d.

[a] Formation of BF_4^-, as $^{10}BF_4^-$ (δ – 151.8 ppm, s) and $^{11}BF_4^-$ (δ – 151.9 ppm, s) isotope mixture[9], observed by $^{19}F\{^1H\}$ NMR spectroscopy. [b] Formation of HBF_4, as $H^{10}BF_4$ (δ – 143.1 ppm, d, J_{FH} = 7.2 Hz) and $H^{11}BF_4$ (δ – 143.1 ppm, d, J_{FH} = 7.2 Hz) isotope mixture, observed by ^{19}F NMR spectroscopy. [c] Reaction was performed in MeCN-d_3:C_6D_6 (9:1) with addition of crypt-222 (0.15 mmol, 15.0 equiv.).

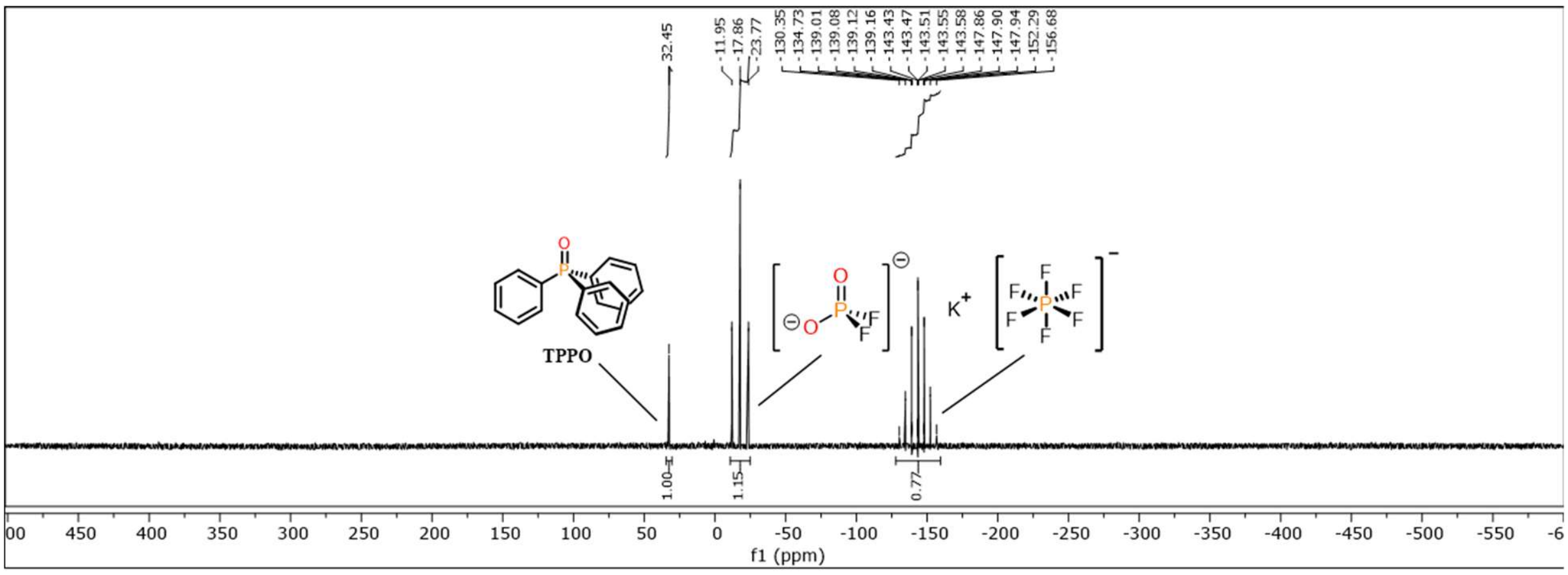

Figure S5. ^{31}P NMR (162.04 MHz, MeCN, C_6D_6) Reaction with $NEt_3{\cdot}3(HF)$ as fluorine source (Table S6, entry 8) showing $PO_2F_2^-$(δ – 17.9 ppm, t, $^1J_{PF}$ = 957.7 Hz) as the major product in 57% spectroscopic yield.

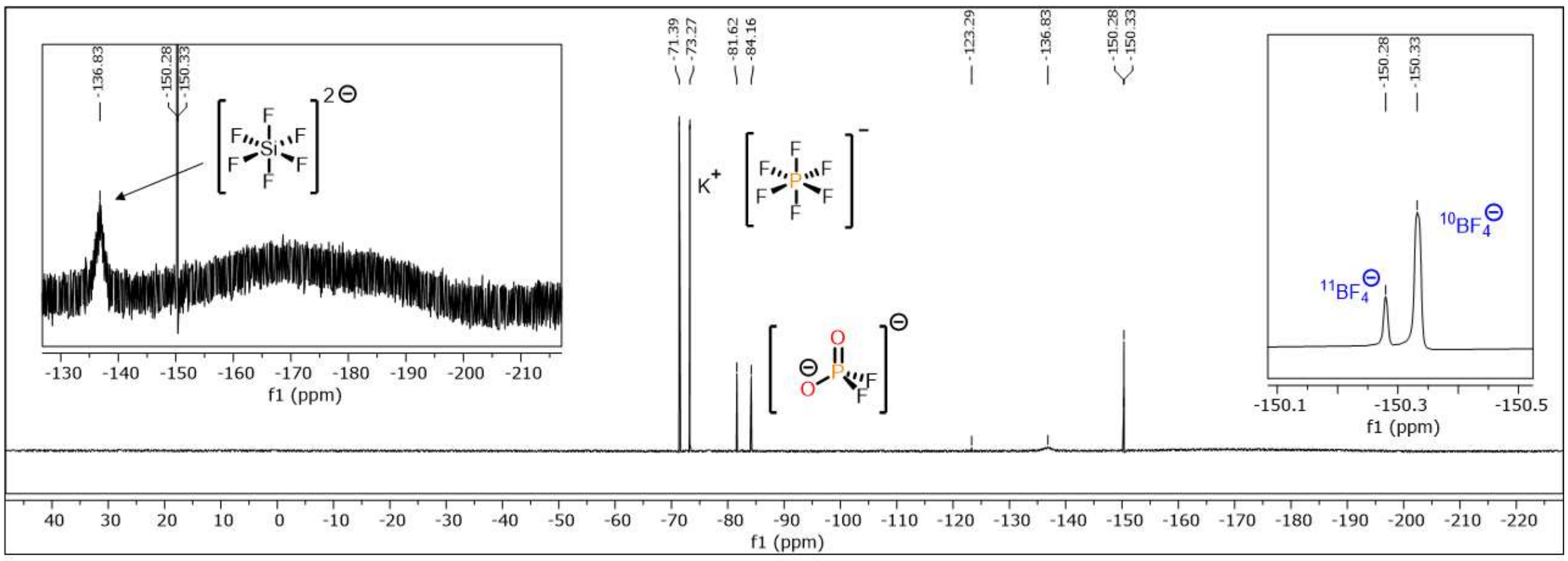

Figure S6. $^{19}F\{^1H\}$ NMR (367.62 MHz, MeCN, C_6D_6) Reaction with $NEt_3{\cdot}3(HF)$ as fluorine source (Table S6, entry 8) showing $PO_2F_2^-$(δ – 17.9 ppm, t, $^1J_{PF}$ = 957.7 Hz) as the major. Insert showing the formation of BF_4^-, as a $^{10}BF_4^-$ (δ ^{19}F – 151.9 ppm, s) and $^{11}BF_4^-$ (δ – 151.8 ppm, s) isotope mixture and SiF_6^{2-} (δ – 136.8 ppm, bs).

2.5. Quinone Screening

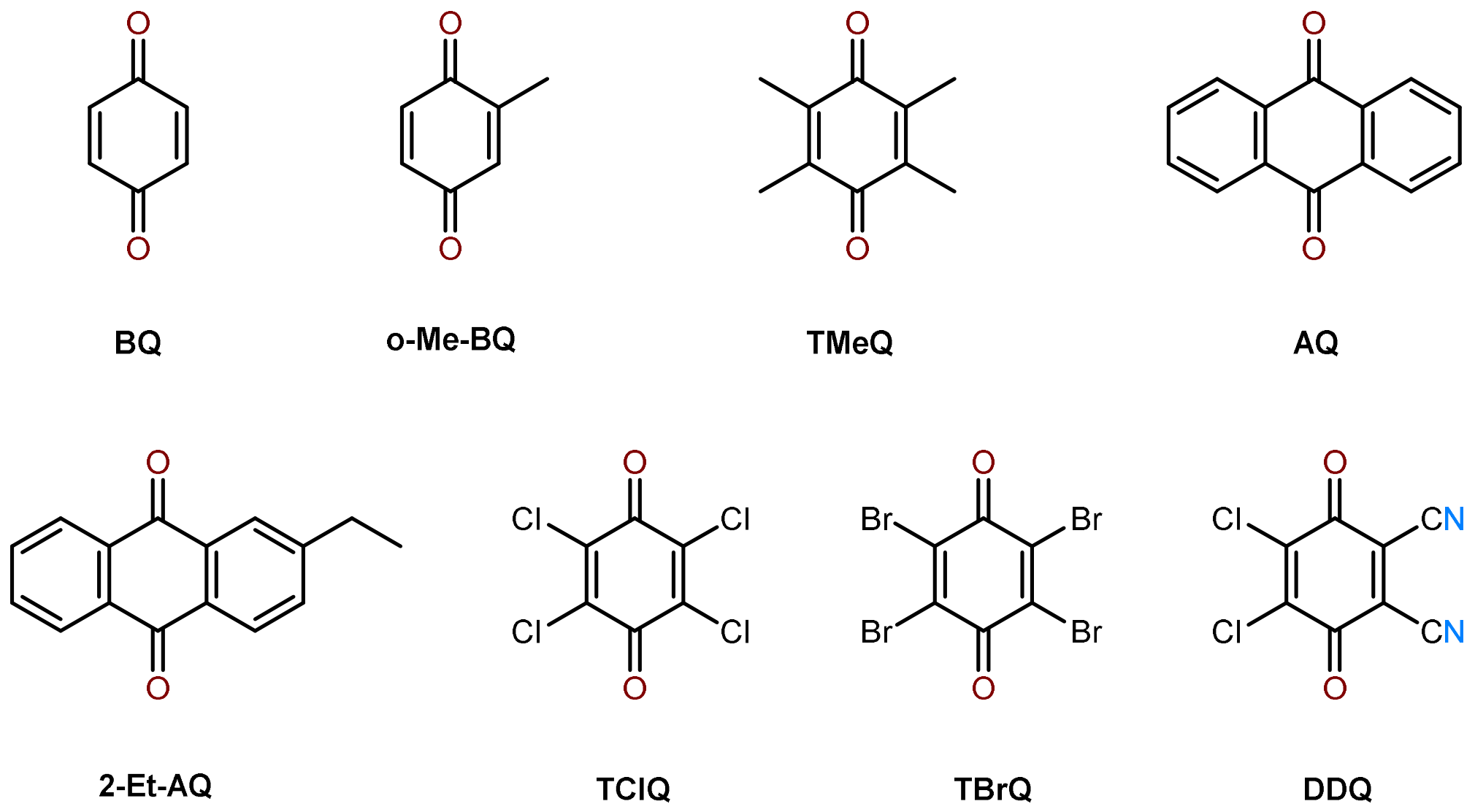

Chart S1. Structures of the *p*-quinone oxidants used in this work, including their respective abbreviations.

According to the general procedure, various *p*-quinones (0.2 mmol, 5.0 equiv.) were screened in the P_4 fluorination reaction using LiF (7.8 mg, 0.3 mmol, 7.5 equiv., Table S7) or KF (17.4 mg, 0.3 mmol, 7.5 equiv., Tables S7–S9) as fluoride source.

Table S7. *p*-Quinone screening with LiF as nucleophile under thermal conditions. Optimal conditions are highlighted in grey.

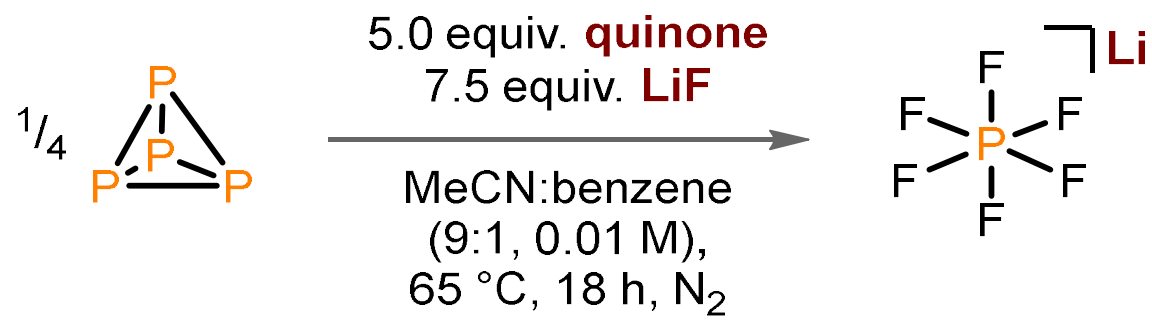

Entry	*p*-quinone	m [mg]	Yield KPF_6 [%]	Conversion P_4 [%]
1	DDQ	45.4	37	>99
2	TBrQ	84.7	n.d.	>99
3	TClQ	49.2	n.d.	>99
4	2-Et-AQ	47.3	n.d.	40
5	TMeQ	32.8	n.d.	2
6	o-Me-BQ	24.4	n.d.	11
7	BQ	21.6	n.d.	33
8	AQ	41.6	n.d.	30

Table S8. Screening of *p*-quinones with KF as nucleophile under thermal conditions. Optimal conditions are highlighted in grey.

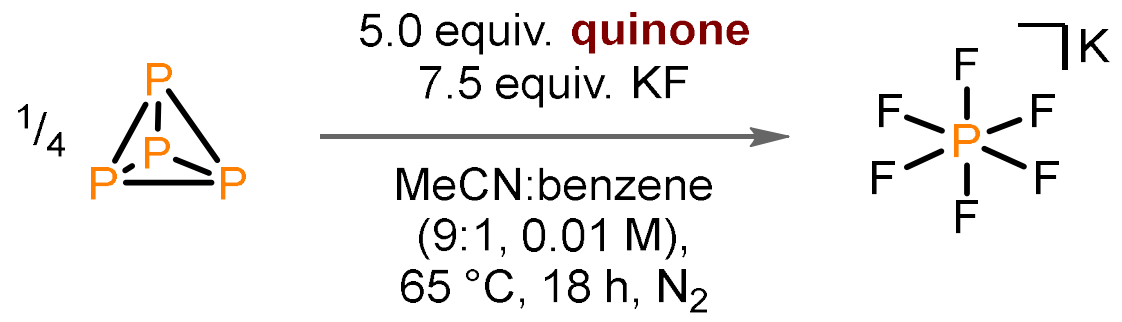

Entry	*p*-quinone	m [mg]	Yield KPF_6 [%]	Conversion P_4 [%]
1	DDQ	45.4	95	>99
2	TBrQ	84.7	43	>99
3	TClQ	49.2	20	>99
4	2-Et-AQ	47.3	n.d.	90
5	TMeQ	32.8	n.d.	15
6	o-Me-BQ	24.4	n.d.	n.d.
7	BQ	21.6	n.d.	>99
8	AQ	41.6	n.d.	24

Table S9. Screening of *p*-quinones with KF as nucleophile under photochemical conditions (see general information). Optimal conditions are highlighted in grey.

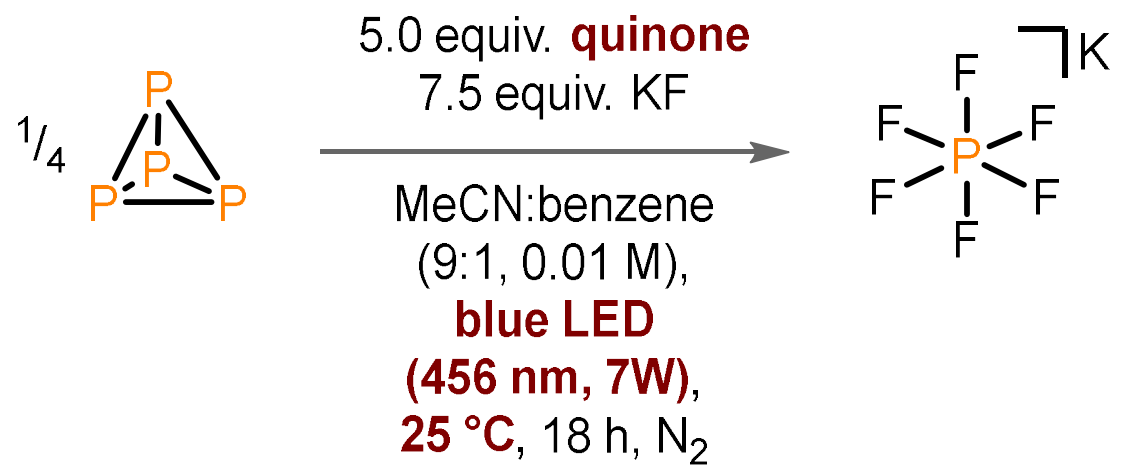

Entry	*p*-quinone	m [mg]	Yield KPF_6 [%]	Conversion P_4 [%]
1	DDQ	45.4	42	>99
2	TBrQ	84.7	12	>99
3	TClQ	49.2	15	78
4	2-Et-AQ	47.3	n.d.	26
5	TMeQ	32.8	n.d.	n.d.
6	o-Me-BQ	24.4	n.d.	72
7	BQ	21.6	n.d.	>99
8	AQ	41.6	n.d.	n.d.

2.6. Screening of Oxidizing Agents

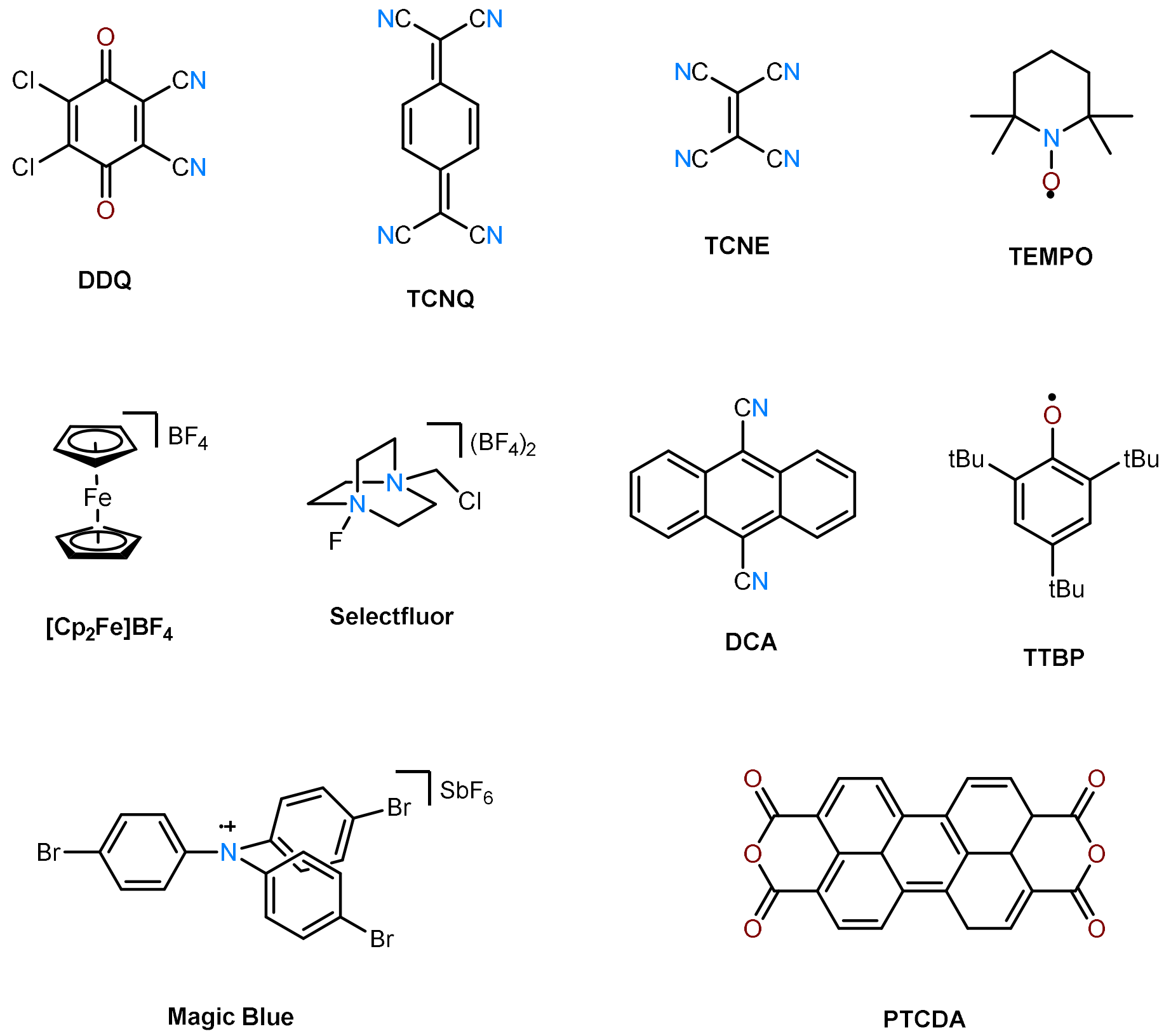

Chart S2. Structures of some oxidants used in this work, including their respective abbreviations .

According to the general procedure, with the oxidizing agent (0.2 mmol, 5.0 equiv.) varied as listed in Table S10.

Table S10. Screening of oxidizing agents. Best conditions highlighted in grey.

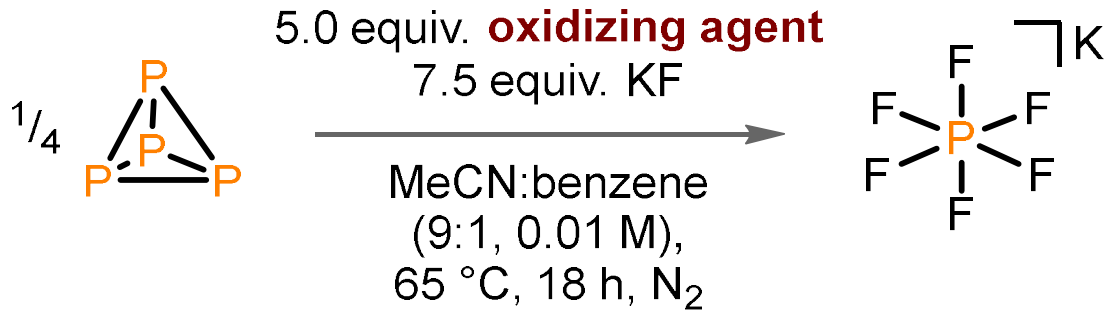

Entry	Oxidizing Agent	m [mg]	Yield KPF_6 [%]	Conversion P_4 [%]
1	DDQ	45.4	95	>99
2	TCNQ	40.8	86	>99
3	TCNE	25.6	52	80
4	TEMPO	31.3	n.d.	>99
5	$[Cp_2Fe]BF_4$	54.6	10	58
6	Selectfluor	106.3	92	>99
7	DCA	45.7	n.d.	0
8	Magic Blue	163.3	n.d.	0
9	PTCDA	78.5	n.d.	0
10	TBPR	52.3	n.d.	0
11	$FeCl_3$	32.4	n.d.	0

The oxidants ferrocenium tetrafluoroborate (10%) and Selectfluor (95%, Chart S2) successfully mediate P_4 fluorination despite not having a *p*-quinonoid structure. SF, a formal "F^+" source, gave excellent yields of KPF_6. However, the synthesis of SF requires F_2, limiting its practicality as a stoichiometric fluoride source in comparison with the simple fluorides used in this work.

According to the general procedure, oxidizing agent (0.2 mmol, 5.0 equiv.), KF (17.4 mg, 0.3 mmol, 7.5.0 equiv.) and crypt-222 (99.8 mg, 0.3 mmol, 7.5 equiv.) were used (Table S11).

Table S11. Screening of selected oxidizing agents with crypt-222 as chelating agent.

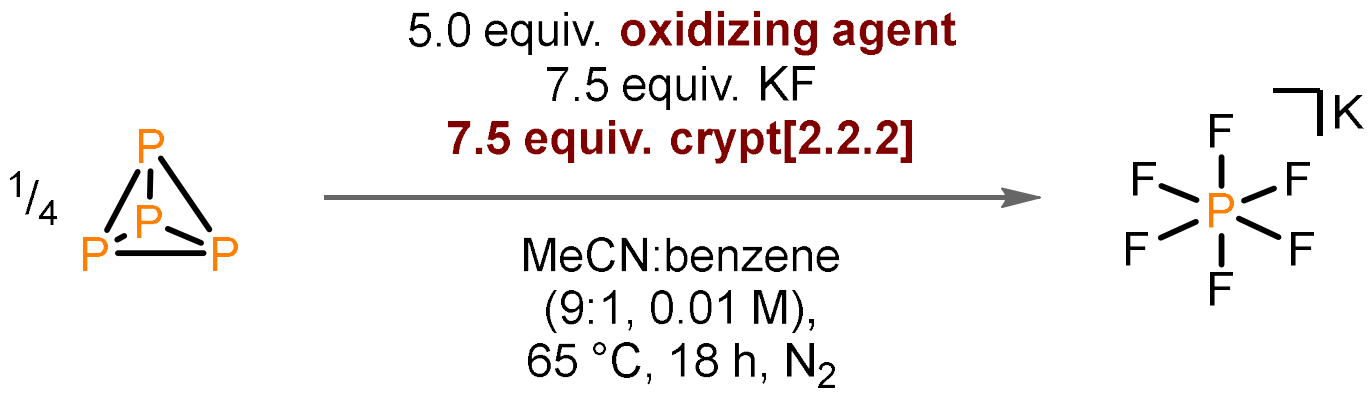

Entry	Oxidizing Agent	m [mg]	Yield KPF_6 [%]	Conversion P_4 [%]
1	Magic Blue	163.3	30	51
2	TEMPO	31.3	n.d.	>99

The reaction of P_4 with KF and Magic Blue in the presence of crypt-222 gave KPF_6 in 30% NMR yield at 51% P_4 conversion (Figure S30). This was accompanied by the formation of additional phosphorus-containing products such as K[HPF_5]. A high loading of crypt-222 (7.5 equiv.) was necessary for the formation of KPF_6 with Magic Blue as the oxidant. In contrast, complete consumption of P_4 (>99% conversion) but no detectable formation of KPF_6 was observed when TEMPO was employed as the oxidant (Figure S32). Instead, a complex mixture of products was obtained, including a proposed (TEMPO)PF_2 species observed in 17% yield by $^{31}P\{^1H\}$ NMR spectroscopy, alongside several unidentified resonances. It is important to note that both Magic Blue and TEMPO have more positive oxidation potentials than DDQ. Nevertheless, this does not improve selectivity or product yield of KPF_6 with respect to using DDQ as an oxidant, indicating that the outstanding properties of DDQ are not exclusively related to the oxidation potential.

3. Additional Synthetic Experiments and Spectroscopic Monitoring Studies

3.1. Sensitivity to Water and Oxidation

According to the general procedure, the reaction was performed in the presence of different contaminants, as listed in Table S12 and Table S13.

Table S12. Control reactions for the DDQ mediated fluorination of P_4 under different conditions.

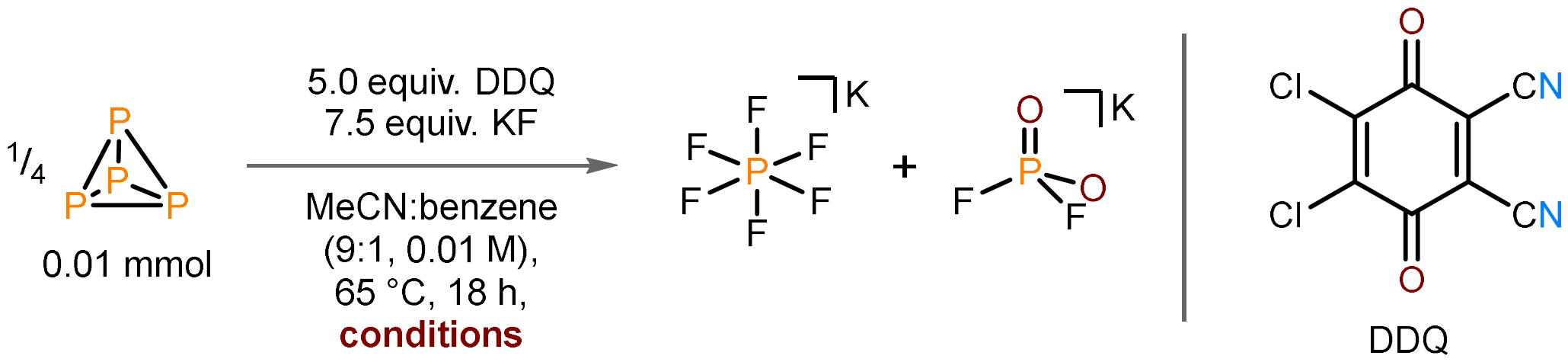

Entry	Conditions	Yield KPF_6 [%]	Yield KPO_2F_2 [%]
1	25°C	41	n.d.
2	reaction opened to air	14	23
3	no DDQ	n.d.	n.d.

The reaction performed on air gave a singlet at δ – 0.8 ppm in the $^{31}P\{^{1}H\}$ NMR spectrum as the major product (Table S12, entry 2) , which was assigned to phosphoric acid or inorganic phosphates (see Figure S34 and S35, for $^{31}P\{^{1}H\}$ and $^{19}F\{^{1}H\}$ NMR spectra with assignments).

Table S13. Screening reactions with varying water content.

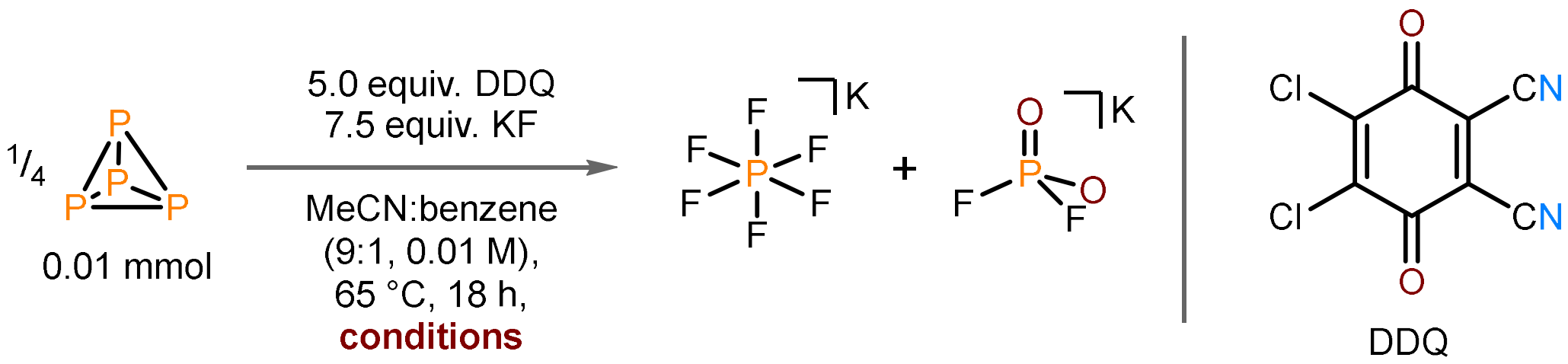

Entry	$V(H_2O)$ [µL]	$c(H_2O)$ [M]	Yield KPF_6 [%]	Yield KPO_2F_2 [%]	Conversion P_4 [%]
1	1	0.05	18	32	>99
2	2	0.09	17	38	>99
3	5	0.23	n.d.	49	>99
4	10	0.46	n.d.	30	>99
5	25	1.13	n.d.	36	>99
6	50	2.22	n.d.	33	>99
7	100	4.27	n.d.	20	>99

3.2. Reaction with Red Phosphorus

In a glovebox, a Schlenk flask was charged with DDQ (455.4 mg, 2.0 mmol, 20 equiv.), KF (174.2 mg, 3.0 mmol, 30 equiv.), red phosphorus (12.4 mg, 0.1 mmol, 1.00 equiv.) and DMF (10 mL). The reaction mixture was heated at 65 °C for 5 days under the conditions specified in Table S14. Conversion and yield were determined by $^{31}P\{^{1}H\}$ NMR spectroscopy, as described in section 1.3

Table S14. Reaction with red phosphorus as substrate under different conditions.

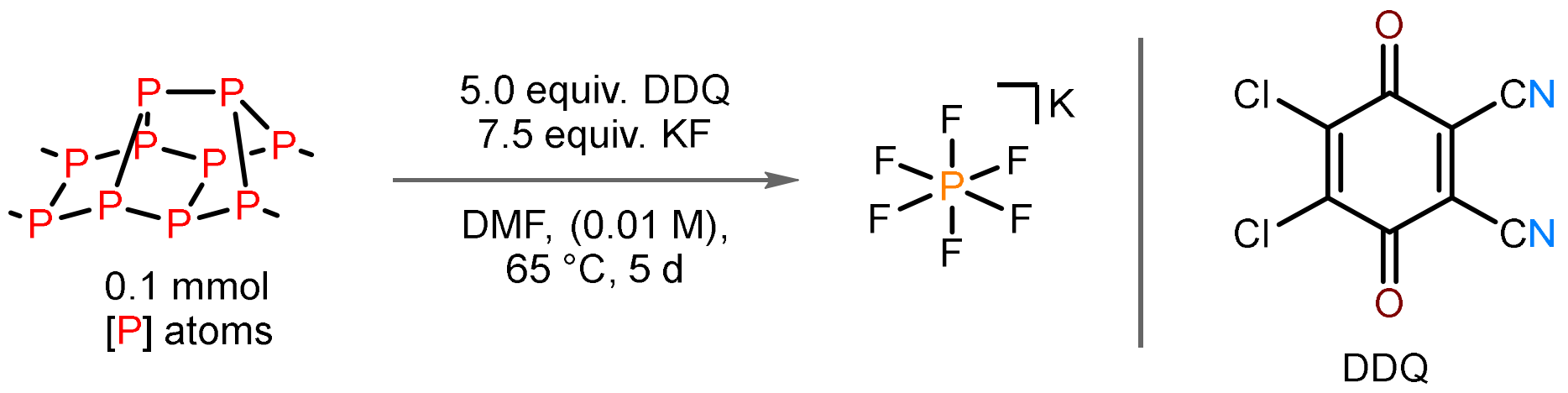

Entry	Conditions	Yield KPF_6 [%]	Yield KPO_2F_2 [%]
1	Under N_2 atmosphere	11	2
2	Under Air	22	5

Alongside KPF_6, several hydrolysis products, such as KPO_2F_2 and $K[HPF_5]$, were observed (Figure S36–S38).[2,6,10] For KPF_6 and $K[HPF_5]$, overlapping signals have been assigned with the corresponding coupling constants reported. Similar ^{31}P and ^{19}F NMR spectra were observed for the reaction under air (Table S14, entry 2).

3.3. Scale-up

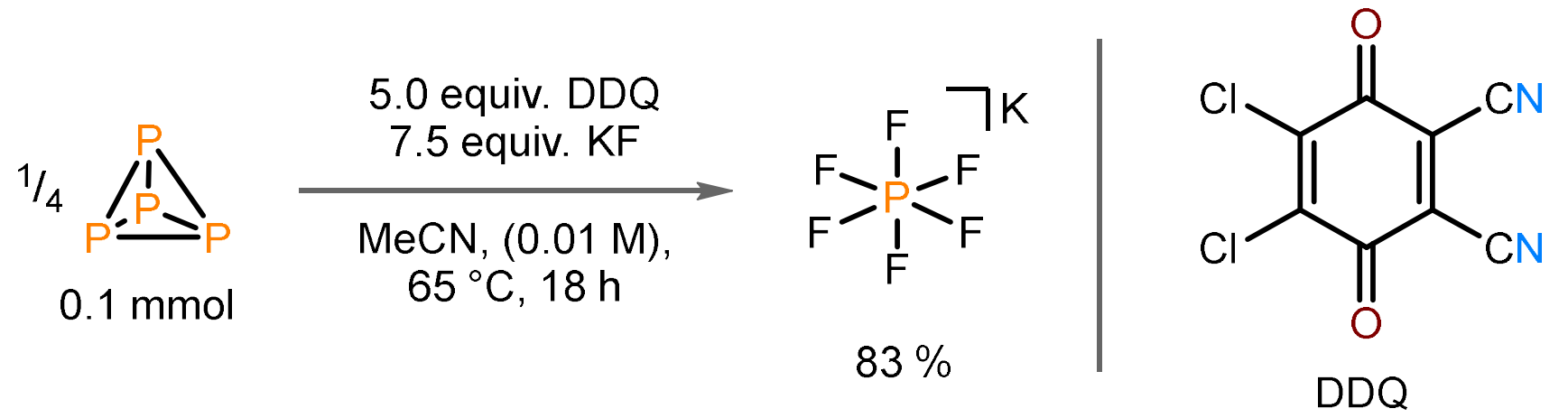

In a glovebox, a Schlenk flask was charged with DDQ (455.4 mg, 2.00 mmol, 20 equiv.), KF (174.2 mg, 3.00 mmol, 30 equiv.), solid P_4 (12.4 mg, 0.1 mmol, 1.00 equiv.) and MeCN (10 mL). The reaction mixture was heated at 65 °C for 20 h. An aliquot (500 µL + 100 µL TPPO, 0.1 M in MeCN) was analyzed by quantitative $^{31}P\{^1H\}$ NMR spectroscopy. The analysis indicated complete conversion of P_4 and a spectroscopic yield of KPF_6 of 83% (Figure S39 – Figure S41).

3.4. Isolation of KPF_6

The reaction was performed according to the scale-up procedure (see section 3.3). $^{31}P\{^1H\}$ NMR spectroscopy indicated complete consumption of P_4 and the formation of KPF_6 in >80% spectroscopic yield. The reaction mixture was allowed to cool to 25 °C and filtered by gravity through a 150 mm Whatman GF/A glass microfiber filter. The solid residue from the filtration was washed with MeCN until the filtrate ran colorless. The combined filtrates were evaporated to dryness under reduced pressure, and the residue was dissolved in H_2O (20 mL). Powdered activated charcoal (PAC, 1.5 g) was added, and the suspension was stirred vigorously for 1 h. Then Celite (0.5 g) was added, and stirring continued for an additional 15 min. The mixture was filtered by gravity through a 150 mm Whatman GF/A glass microfiber filter. The filter cake was washed thoroughly with H_2O. Evaporation of the combined filtrates under reduced pressure afforded an off-white solid. The solid was extracted with hot MeCN (50 °C, 4 × 10 mL). Subsequently, the combined extracts were filtered through a 0.2 µm PTFE syringe filter to remove residual PAC particles. The solvent was removed under vacuum and the residual solid was dried in vacuo at 65°C for 18h, to afford KPF_6 as a colorless powder in 62% (47 mg; yield corrected for impurities as described below; uncorrected yield: 64%) yield. For $^{31}P\{^1H\}$ and ^{19}F NMR spectra, see Figures S42–S45.

$^{31}P\{^1H\}$ NMR (202.48 MHz, 298 K, D_2O) δ – 144.6 (sept., $^1J_{PF}$ = 706.4 Hz) ppm.

^{19}F NMR (470.59 MHz, 298 K, D_2O) δ – 72.9 (d, $^1J_{PF}$ = 706.4 Hz) ppm.

Elemental Analysis found (calcd.) for $KPF_6 \cdot 0.2\ H_2O \cdot 0.05$ MeCN; C: 0.33 (0.63), H: 0.80 (0.29), N: 0.93 (0.37), F: 60.03 (60.08), P: 16.35 (16.33), K: 20.60 (20.61)

The amounts of residual water and MeCN in the sample were quantified by ^{1}H NMR spectroscopy (Figure S46) using 1,3,5-trimethoxybenzene (TMB) as internal standard. The integrals were normalized to the aryl protons of TMB to determine the absolute molar amounts of H_2O and MeCN according to:

$$n_{H_2O} = \frac{I_{H_2O}/2}{I_{TMB}/3} \cdot n_{TMB} \qquad n_{MeCN} = \frac{I_{H_2O}/3}{I_{TMB}/3} \cdot n_{TMB} \qquad \text{(eq. 1 \& 2)}$$

where I denotes the respective signal integrals and n_{TMB} the known amount of internal standard. The amount of KPF_6 is estimated from the total mass of the sample by subtracting the contributions of residual solvent:

$$n_{KPF_6} = \frac{m_{sample} - n_{H_2O}M_{H_2O} - n_{MeCN}M_{MeCN}}{M_{KPF_6}} \qquad \text{(eq. 3)}$$

With M_{H2O} = 18.02 g/mol, M_{MeCN} = 41.05 g/mol and M_{KPF6} = 184.06 g/mol. The stoichiometric coefficients x and y in $KPF_6 \cdot x H_2O \cdot y$ MeCN are obtained as

$$x = \frac{n_{\mathrm{H_2O}}}{n_{\mathrm{KPF_6}}} \qquad y = \frac{n_{\mathrm{MeCN}}}{n_{\mathrm{KPF_6}}} \qquad \text{(eq. 4 \& 5)}$$

With n_{TMB} = 0.0077 mmol (1.3 mg), m_{sample} = 4.9 mg, I_{H2O} = 2.82, I_{MeCN} = 1.09 the values amount to x = 0.18 and y = 0.046, thus confirming the values of x and y obtained independently by solving the elemental analysis for KPF_6, H_2O and MeCN by linear regression using JASPER v2.0 - JavaScript Percentage Elemental Results Calculator (University of York, https://www.yorku.ca/pgpotvin/public/Jasper/jasper2.htm).

Note: The reaction solvents (MeCN and especially benzene, if reaction is performed according to the general procedure using a P_4 stock solution (0.1 M in benzene) instead of solid P_4) need to be completely removed under vacuum to ensure that K[DDQ˙] is quantitatively absorbed by PAC. Residual organic solvents significantly reduce K[DDQ˙] uptake, resulting in a brown-colored filtrate after fine filtration (0.2 µm PTFE) or necessitating the use of very large amounts of PAC, which in turn severely hampers filtration. The addition of Celite to the PAC suspension is crucial for retaining ultrafine PAC particles that otherwise pass through glass-microfiber filters (Whatman GF/A, approx. 1 µm pore size). The amount of Celite

used in this procedure minimizes product loss while ensuring efficient removal of fine particulates. Upon filtration through a Celite pad (by gravity or vacuum), KPF_6 is partially retained in the Celite, resulting in a reduced isolated yield.

3.5. Effect of TEG as a Co-Solvent

According to the general procedure, the reaction was performed in the presence of TEG as co-solvent (10% v/v, 100 μL) in the solvents specified in Table S15.

Table S15. Reactions with TEG as additive under different conditions.

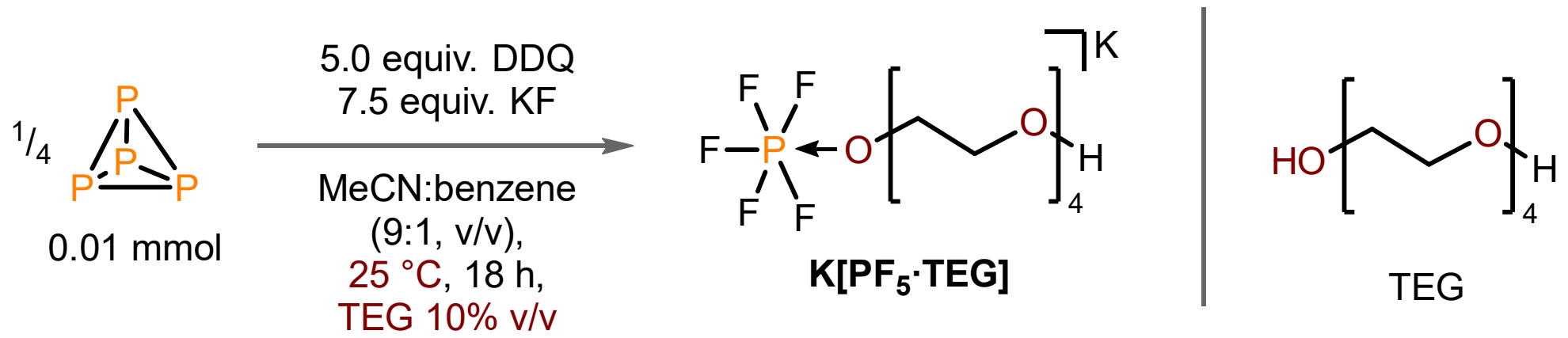

Entry	Conditions	Yield K[PF_5·TEG] [%]	Yield K[HPF_5] [%]	Conversion P_4 [%]	Yield KPF_6 [%]
1	MeCN	27	11	>99	n.d.
2	DCM	75	n.d.	75	n.d.

When the reaction is performed in the presence of TEG (10% v/v in MeCN), the $^{31}P\{^{1}H\}$ and ^{19}F NMR spectra (Figures S47 and S48;Table S15, entry 1) indicate the formation of K[PF_5·TEG] together with a significant amount of K[HPF_5][10], with nearly complete consumption of P_4 after 18 h at 25 °C. The presence of K[HPF_5] suggests partial hydrolysis under these conditions.[18] In contrast, when the reaction is carried out in DCM/TEG (9:1) (Figures S49 and S50; Table S15, entry 2), K[PF_5·TEG] is the predominant fluorinated phosphorus species, with no detectable K[HPF_5]. Minor additional resonances observed in both solvent systems are attributed to hydrolysis and solvolysis involving TEG. One of these side-products was tentatively assigned as a TEG ester of monofluorophosphoric acid $K_x[OP(O)_x(TEG)_{2-x}F]$ (x = 0, 1) (δ ^{31}P – 8.3 ppm, d, $^{1}J_{PF}$ = 972.9 Hz), based on coupling constants and chemical shifts of related compounds reported in the literature.[2,6]

Table S16. Reactions with TEG at 65 °C using varying amounts of glycol, showing formation of KPF_6. Full conversion of P_4 in all cases.

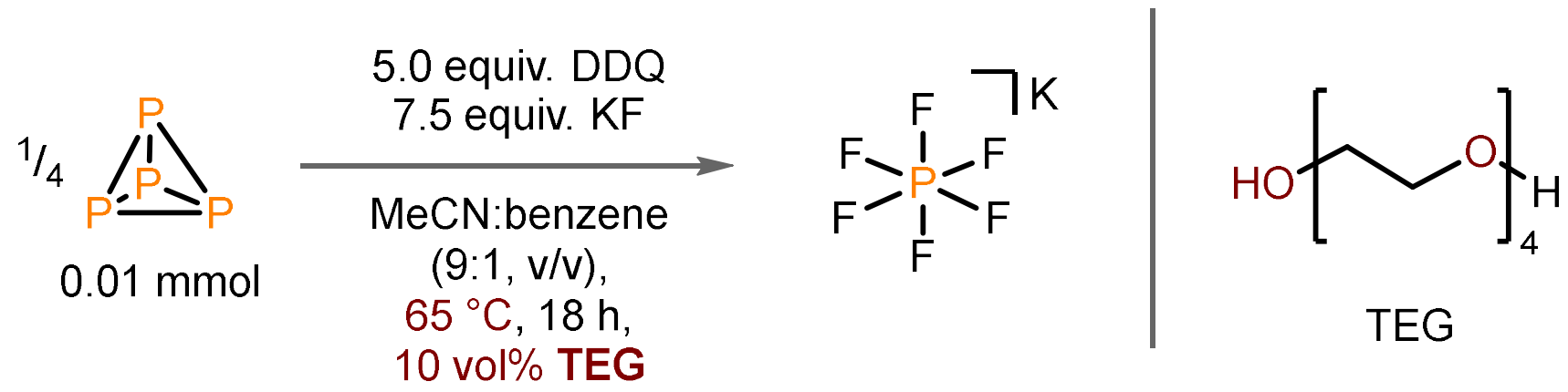

Entry	equiv. TEG	c(TEG) [mM]	V(TEG) [μL]	Yield Phosphates [%]	Yield KPF_6 [%]
1	0.10	0.004	0.54	0	91
2	0.25	0.010	1.40	4	89
3	0.50	0.020	2.80	11	74
4	1.00	0.040	5.60	9	89
5	2.00	0.080	11.2	42	55
6	16.00	0.640	22.4	72	23

Reactions performed at 65 °C resulted in the formation of PF_6^-, suggesting that the process is kinetically controlled (see Figure S51–Figure S52). Notably, K[PF_5·TEG] was not observed upon cooling down the reaction mixture, neither after extended storage at room temperature, as confirmed by NMR spectroscopy. In addition, reactions conducted at 65 °C with increasing amounts of TEG (Table S16) exhibited progressively greater formation of hydrolysis and solvolysis products as the TEG loading increased. Showing the delicate balance between solvent, temperature and TEG amount required for selective formation of K[PF_5·TEG].

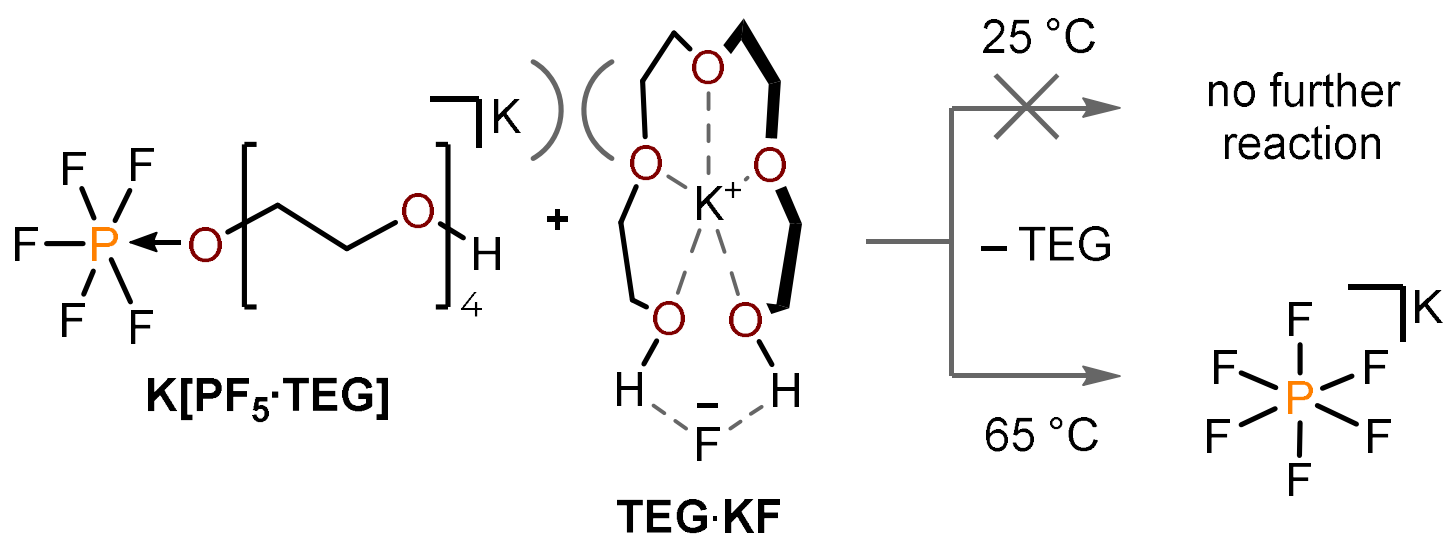

Scheme S3. Irreversible formation of KPF_6 at elevated temperature (65 °C). The selective conversion is attributed to a kinetically favored pathway.

3.6. Spectroelectrochemistry of DDQ

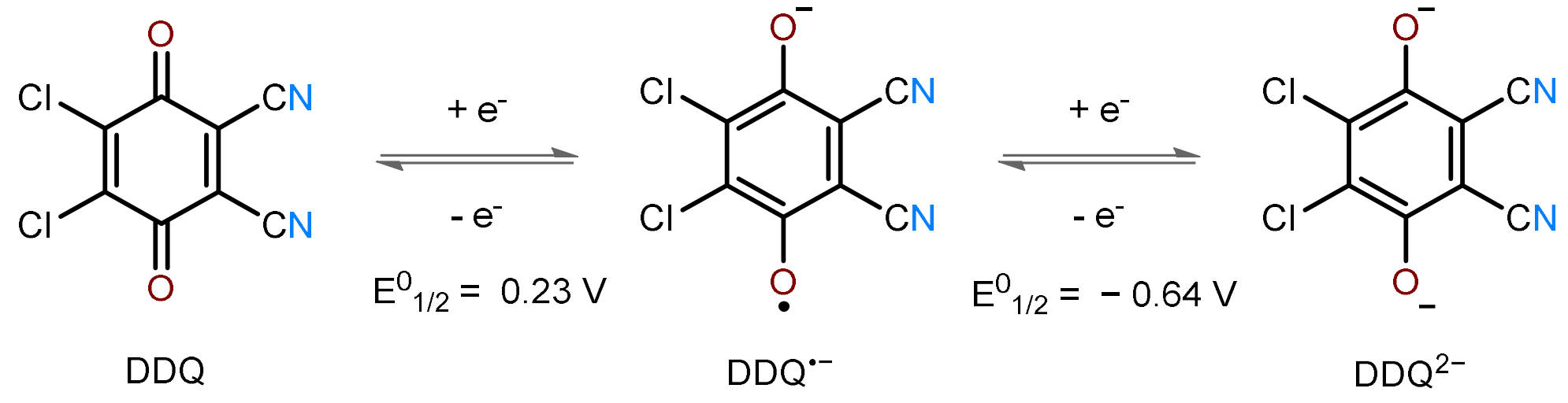

Scheme S4. Reversible single electron reductions of DDQ towards DDQ^{-} and DDQ^{2-} with corresponding half wave potentials (mV vs. Fc/Fc^{+})

DDQ (4.5 mg, 0.01 mmol) and $^{n}Bu_4NBF_4$ (TBABF) supporting electrolyte (32.9 mg, 0.1 mmol) were dissolved in MeCN (1.0 mL), transferred into an OTTLE cell, and subjected to electrolysis while being simultaneously analyzed by UV–Vis spectroscopy. Stepwise electrolysis was performed as shown in Figure S8, where each square represents an electrolysis step (ΔE = 50 mV) with a duration of t = 10 s, followed by the acquisition of a UV–Vis spectrum. The procedure was automated using the MetrOhm NOVA 2.1.6 software.

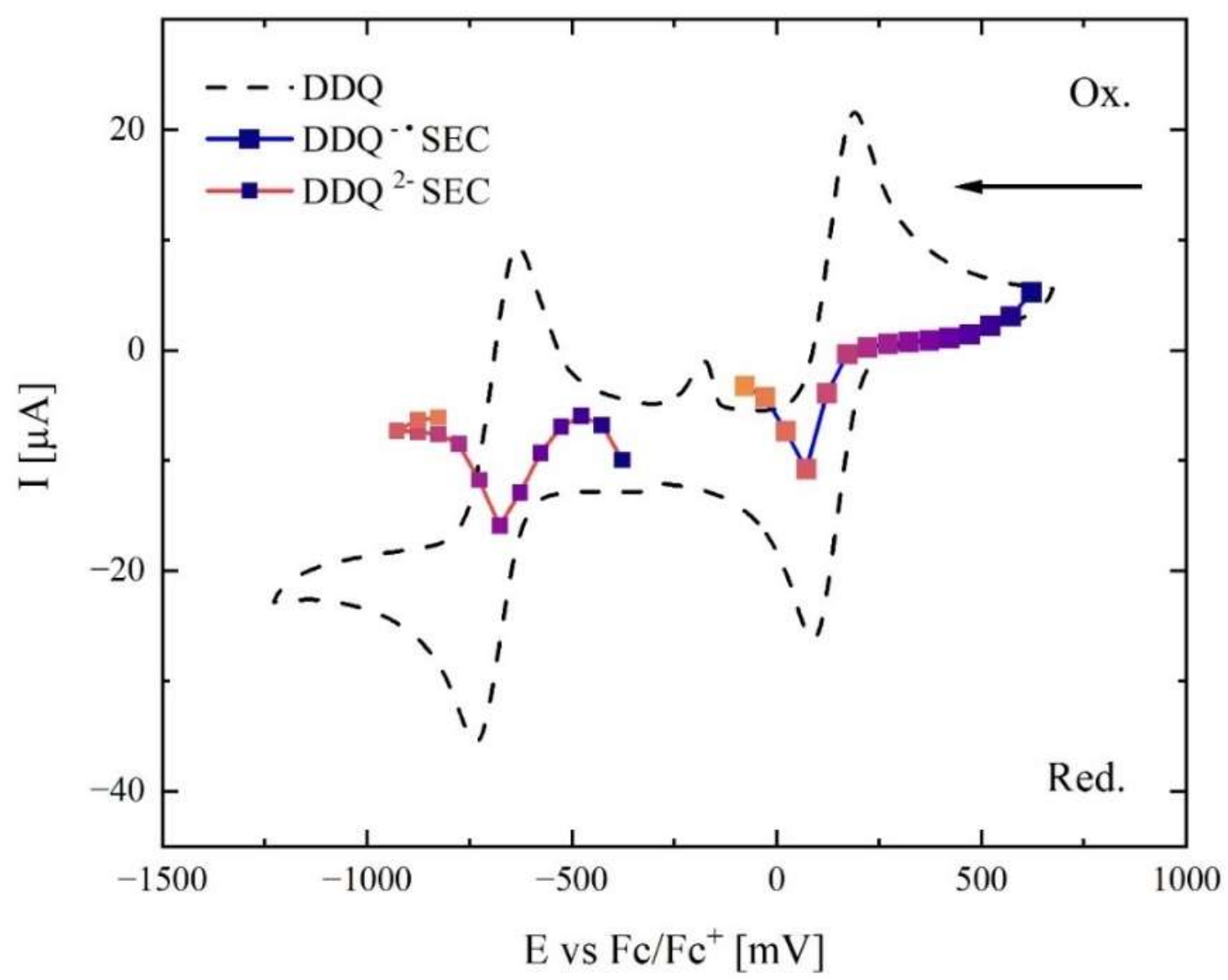

Figure S7. Cyclic Voltammogram of DDQ (black dashed line ---, scan rate 0.1 V/s). Applied potential (vs. Fc/Fc^{+}) plotted vs. measured current in the SEC experiment; blue line first reduction to DDQ^{-} ($E^{0}_{1/2}$ = 0.23 V); red line second reduction of DDQ^{2-} ($E^{0}_{1/2}$ = − 0.64 V). UV-Vis spectra were recorded at each square (step size and duration ΔE = 0.05 V, t = 10 s), color corresponds to spectral traces shown in FiguresFigure **S8** and Figure **S10**. Conditions: OTTLE cell, DDQ (0.01 M) in MeCN with $TBABF_4$ (0.1 M) as supporting electrolyte.

First Reduction of DDQ to DDQ$^{\bullet-}$

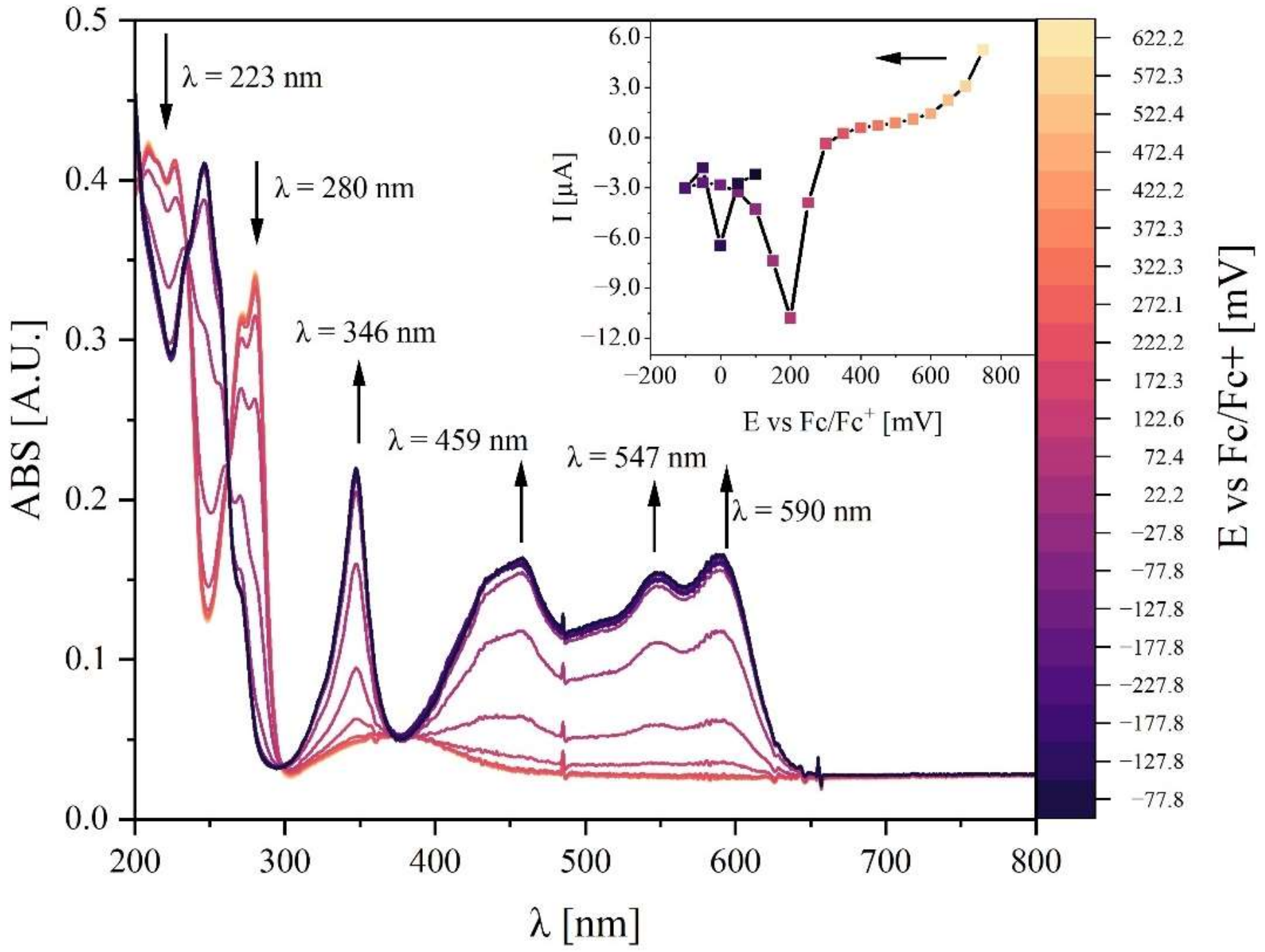

Figure S8. UV-Vis spectra obtained by following the gradual electrolytic reduction from DDQ to DDQ$^{\bullet-}$. Inset showing measured voltage and current during SEC experiment.

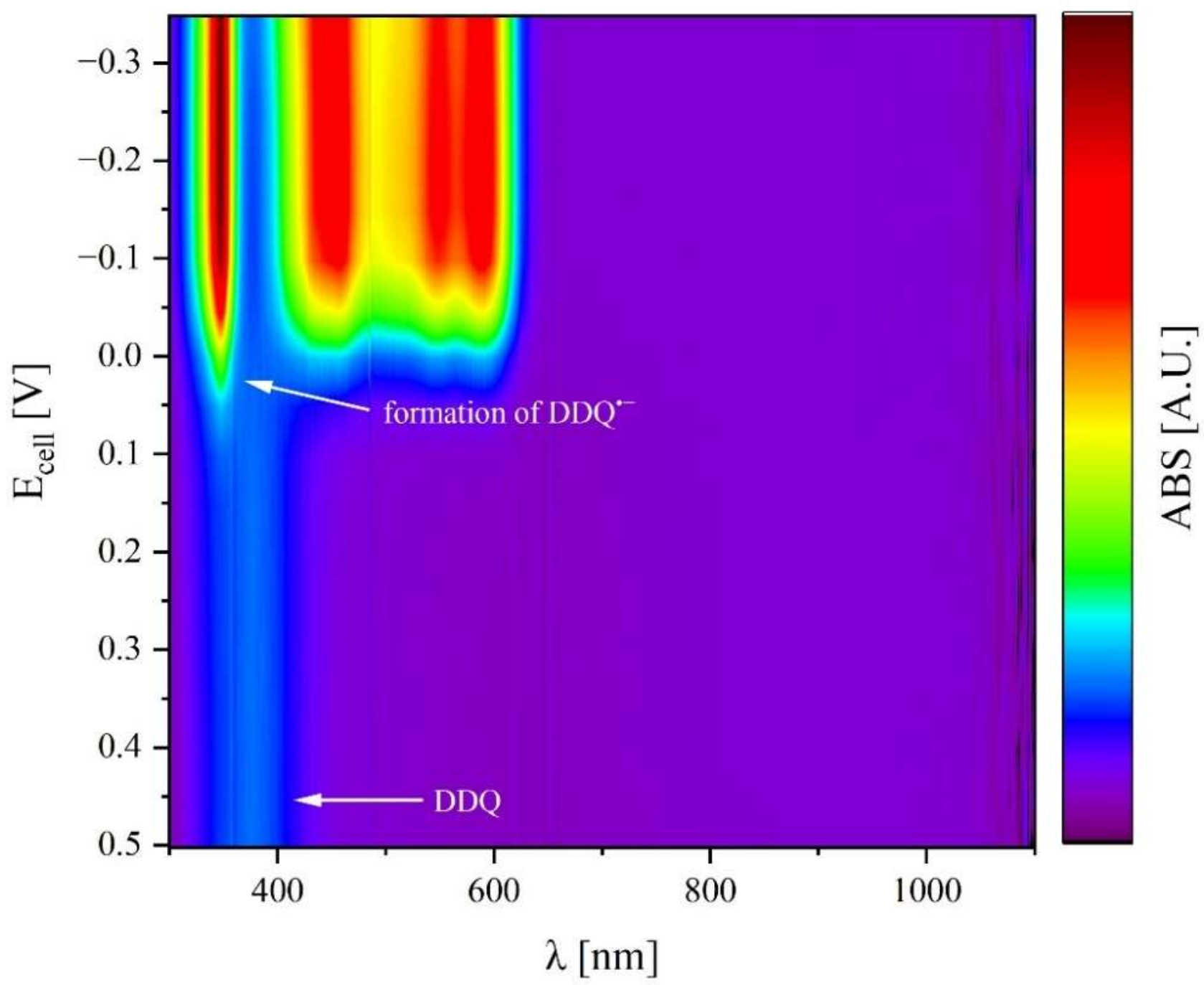

Figure S9. Spectroelectrochemistry of the reduction from DDQ to DDQ$^{\bullet-}$, displayed as a contour plot. (Dark red: 0.30 A.U., purple: 0.00 [A.U.].

Second Reduction of $DDQ^{\cdot-}$ to DDQ^{2-}

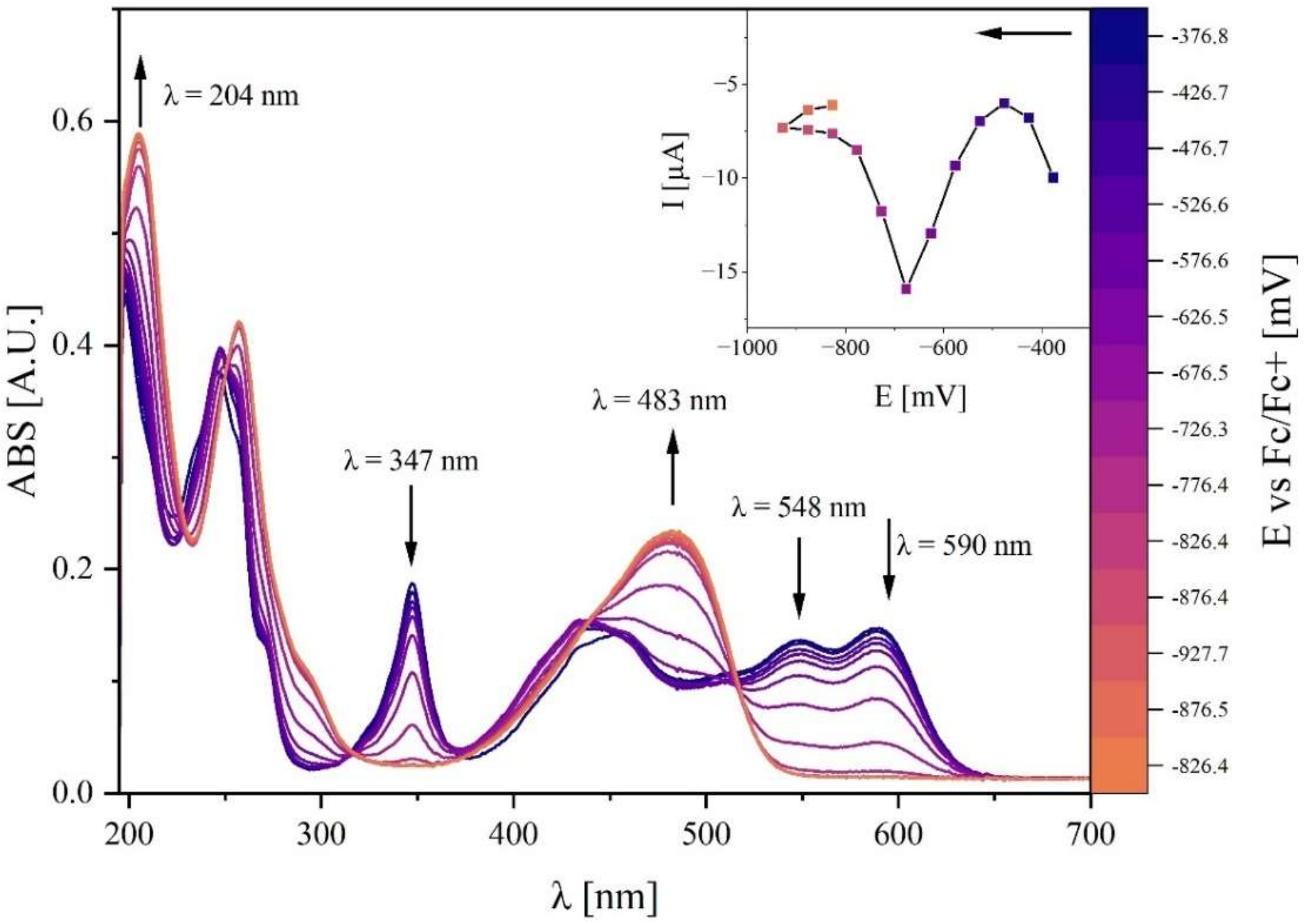

Figure S10. UV-Vis spectra obtained by following the gradual electrolytic reduction from $DDQ^{\cdot-}$ to DDQ^{2-}.

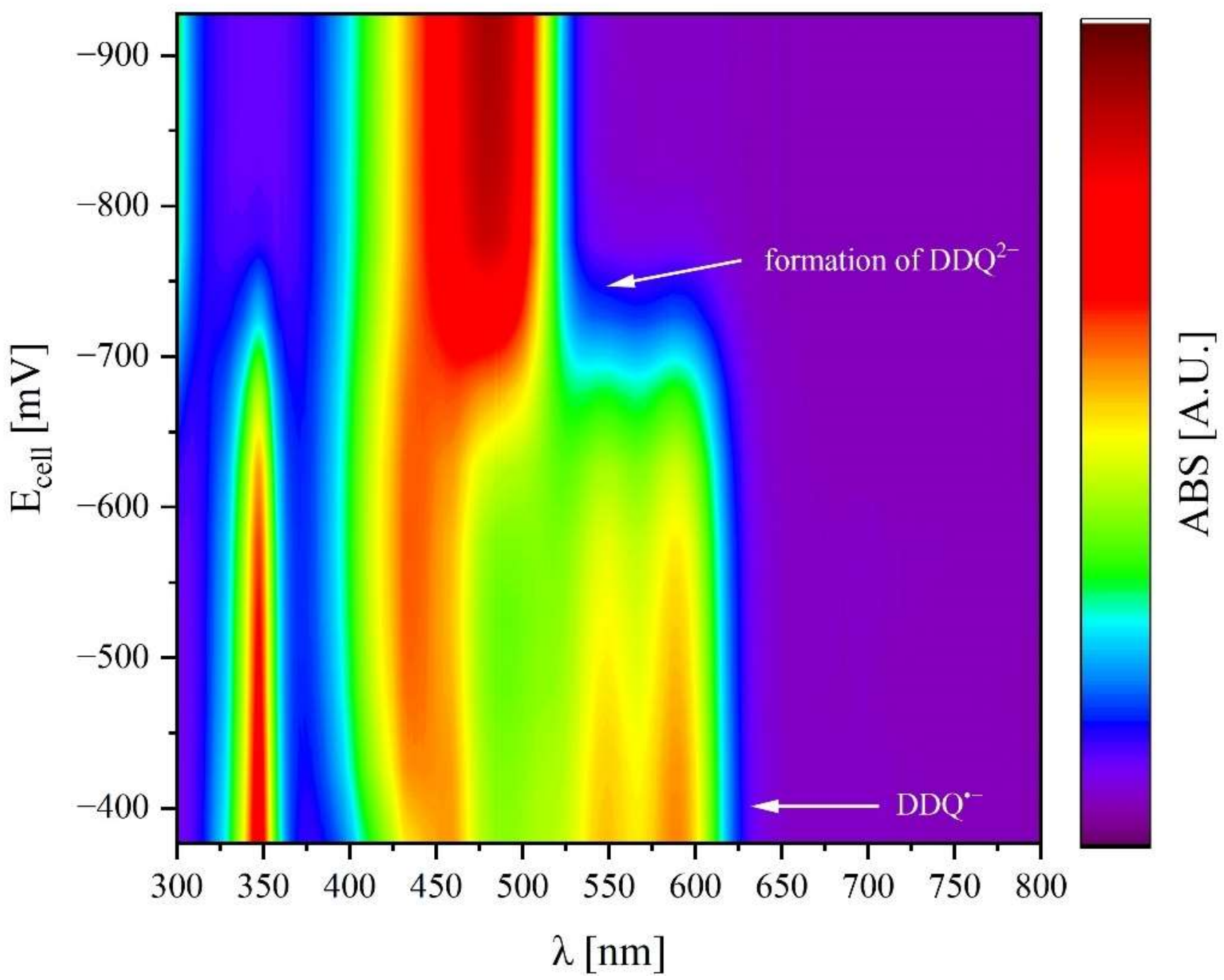

Figure S11. Spectroelectrochemistry of the reduction from $DDQ^{\cdot-}$ to DDQ^{2-}, displayed as a contour plot. (Dark red: 0.30, purple: 0.00 [A.U.].

3.7. UV-Vis Studies on the Reactivity of DDQ Towards Fluoride

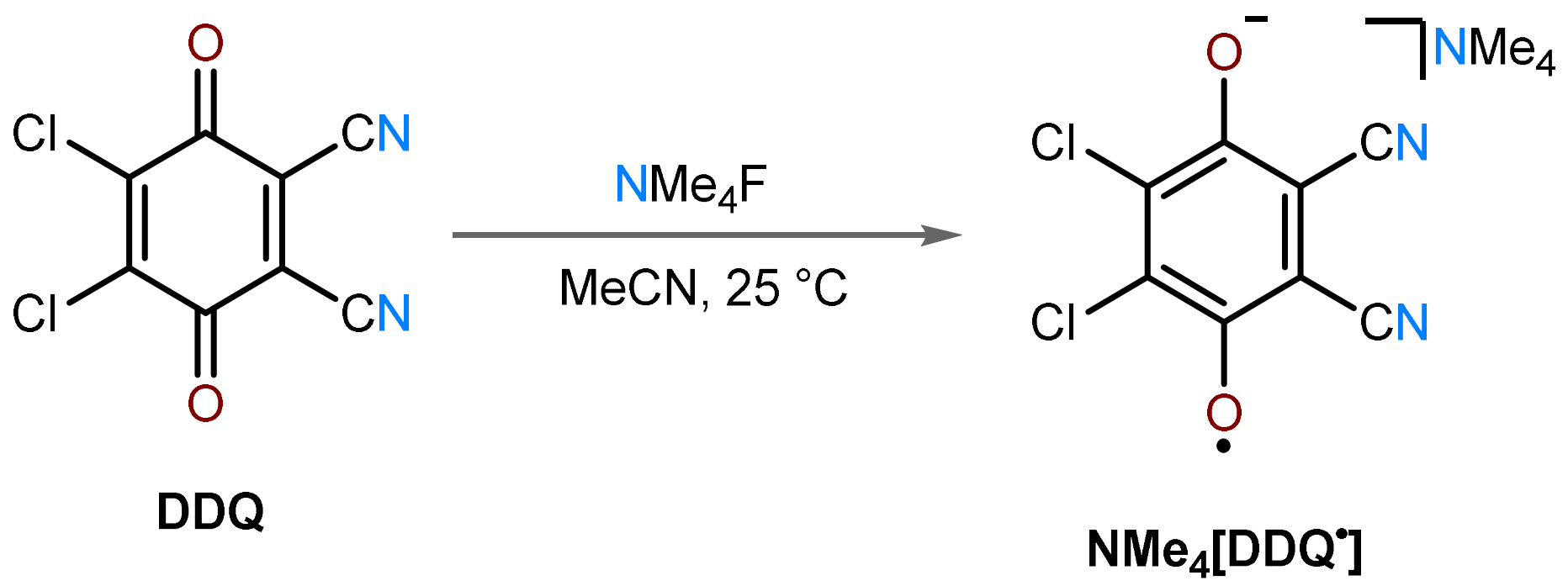

Scheme S5. Reaction conditions for UV–Vis studies of the reaction between DDQ and NMe_4F.

A 10 mM DDQ stock solution was prepared by dissolving DDQ (22.7 mg, 10.0 μmol) in MeCN (10.0 mL). Similarly, a 10 mM NMe_4F stock solution was prepared by dissolving NMe_4F (9.3 mg, 10.0 μmol) in MeCN (10.0 mL). Analytical flasks were used to minimize volumetric error. Samples of variable concentrations were obtained by combining the two solutions with MeCN (2.0 mL), as shown in Table S17.

Note: NMe_4F was used here as the poor solubility of KF did not allow the preparation of a stock solution of the desired concentration. Use of NMe_4F as a stoichiometric flouride source gave a high yield of NMe_4PF_6 (92%) which is comparable to the yield of KPF_6 obained using KF (95%, Table S5, entrys 3 and 7).

Table S17. Prepared UV-Vis samples for the investigation of the reaction between DDQ and NMe_4F.

Entry	V(NMe_4F stock) [μL]	V(DDQ) [μL]	V total [μL]	c(DDQ) [μM]	c(NMe_4F) [μM]	NMe_4F:DDQ [%]
1	-	200	2200	909.1	-	
2	35	200	2235	894.9	156.6	17.5
3	45	200	2245	890.9	200.4	22.5
4	55	200	2255	886.9	243.9	27.5
5	65	200	2265	883.0	287.0	32.5
6	80	200	2280	877.2	350.9	40.0
7	90	200	2290	873.4	393.0	45.0

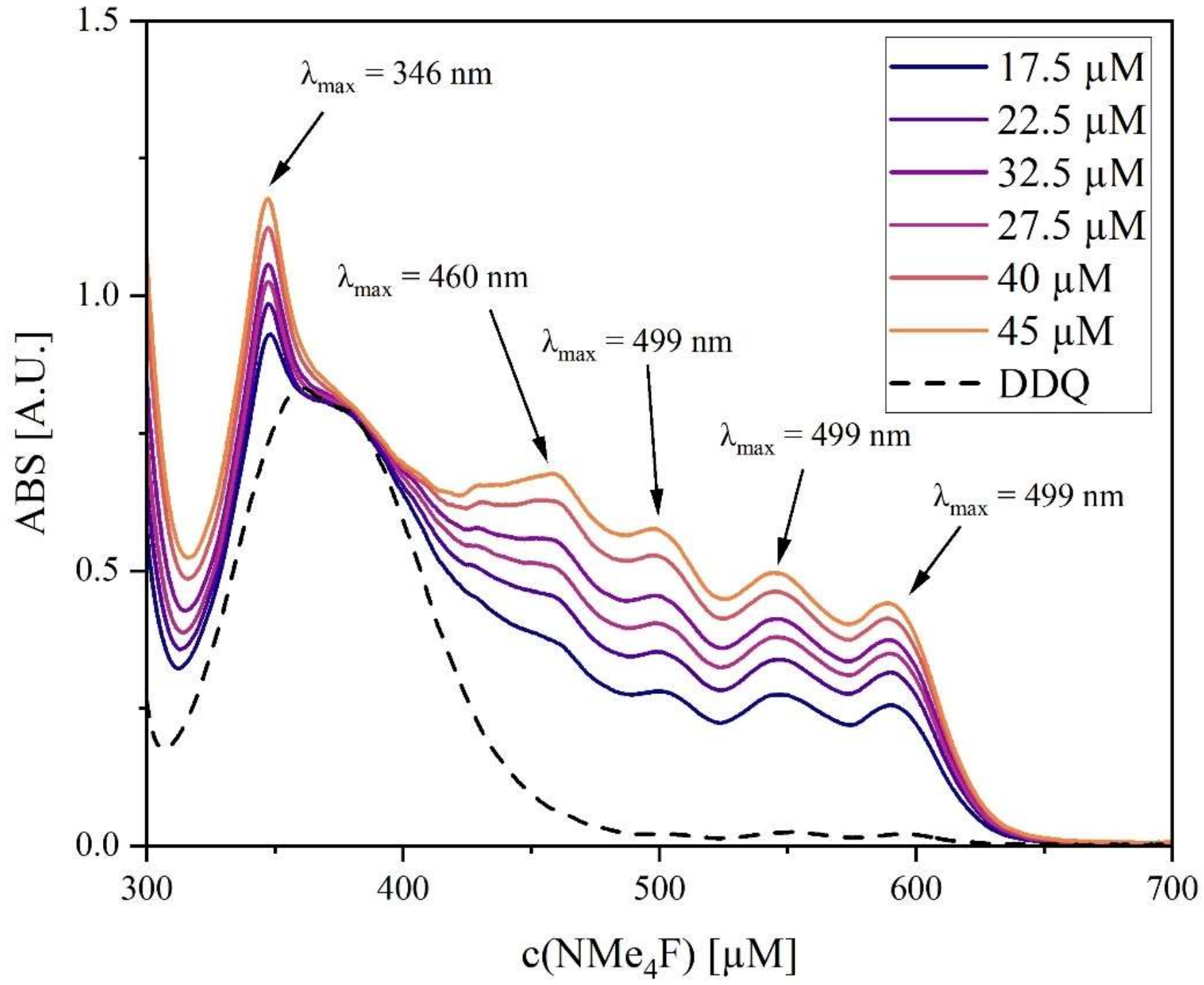

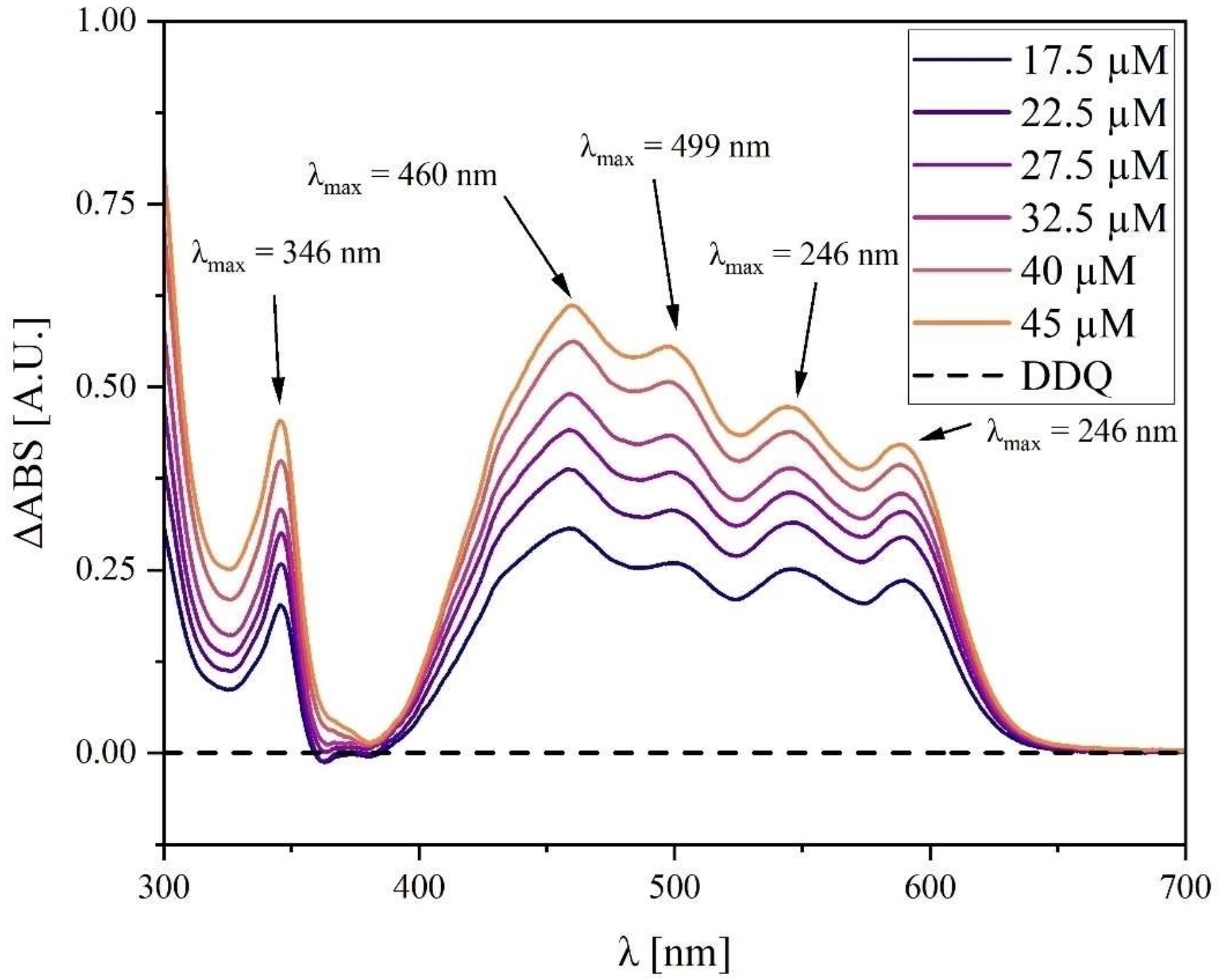

Figure S12. (top) UV-Vis absorption spectrum of DDQ with varying concentration of NMe_4F (from 0.0 to 45.0 μM) showing the formation of $DDQ^{\cdot-}$. (bottom) Difference absorption spectrum with absorbance of DDQ (Table S17, entry 1) set as reference.

3.8. UV-Vis Studies on the Reactivity of DDQ Towards P_4

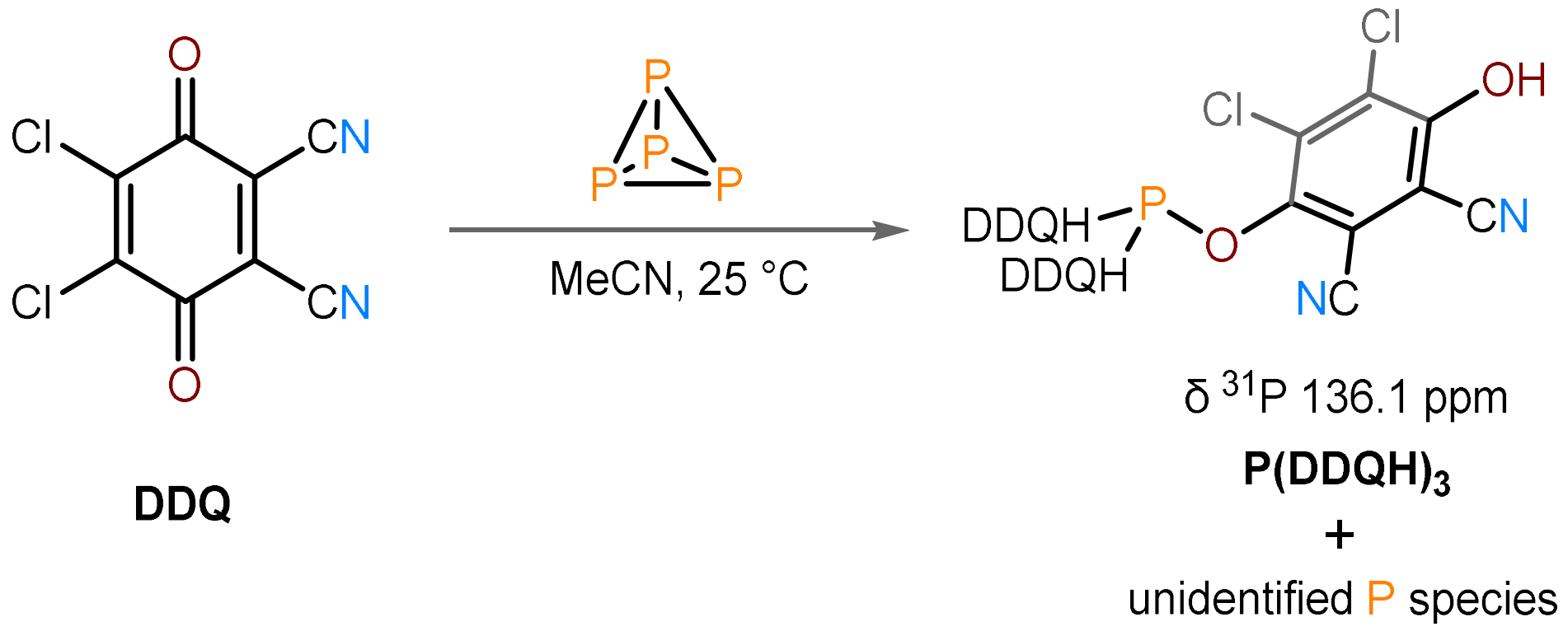

Scheme S6. Reaction conditions for UV–Vis studies of the reaction between DDQ and P_4, with formation of proposed phosphite $P(DDQH)_3$.

To a quartz cuvette filled with MeCN (2.0 mL), DDQ (50.0 μL, 0.01 M stock solution in MeCN) and P_4 (100.0 μL, 0.01 M stock solution in benzene) were added. The reaction was monitored by UV–Vis spectroscopy for four days.

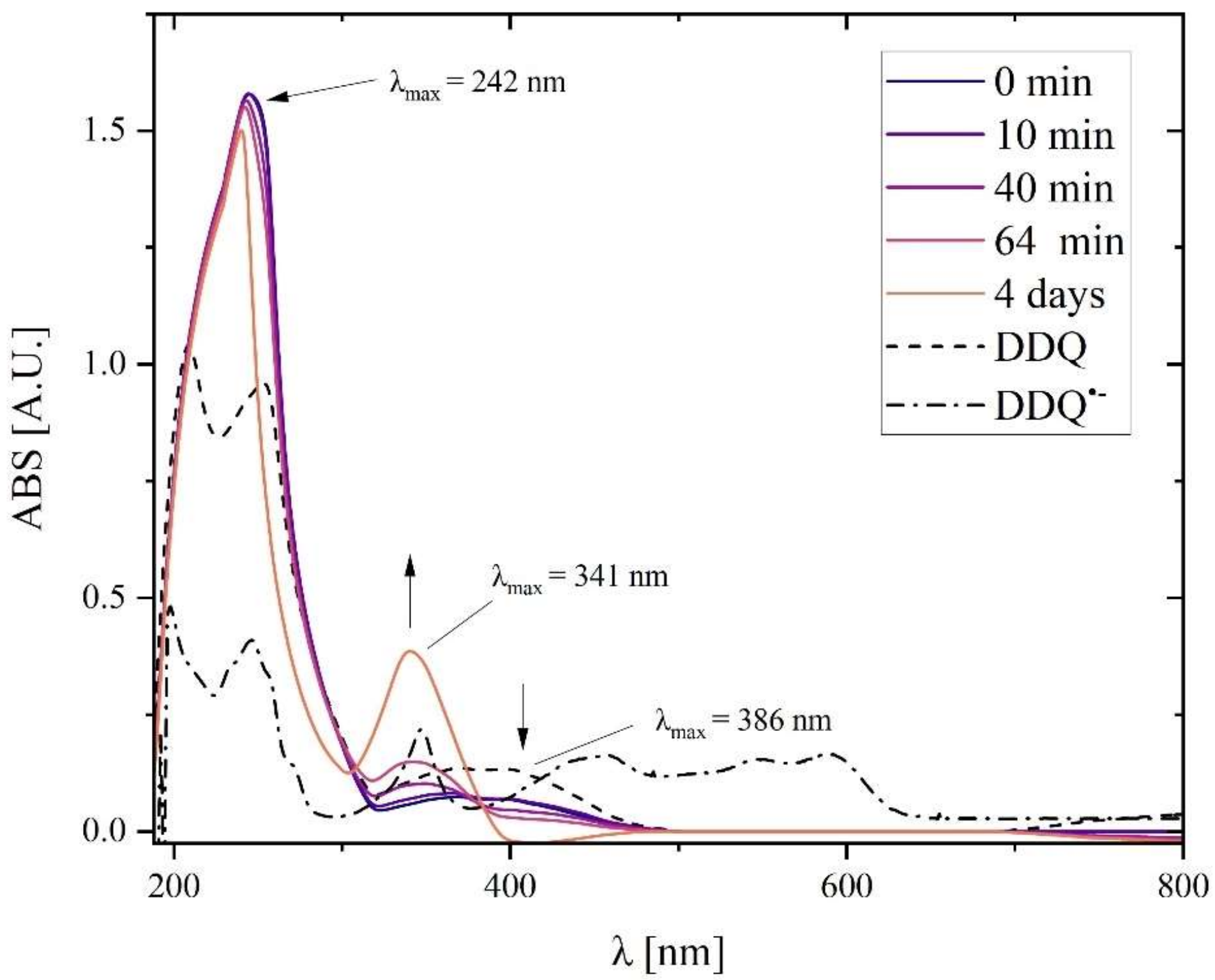

Figure S13 . Reaction of DDQ (0.23 mM) and P_4 (0.47 mM), absorption monitoring over four days.

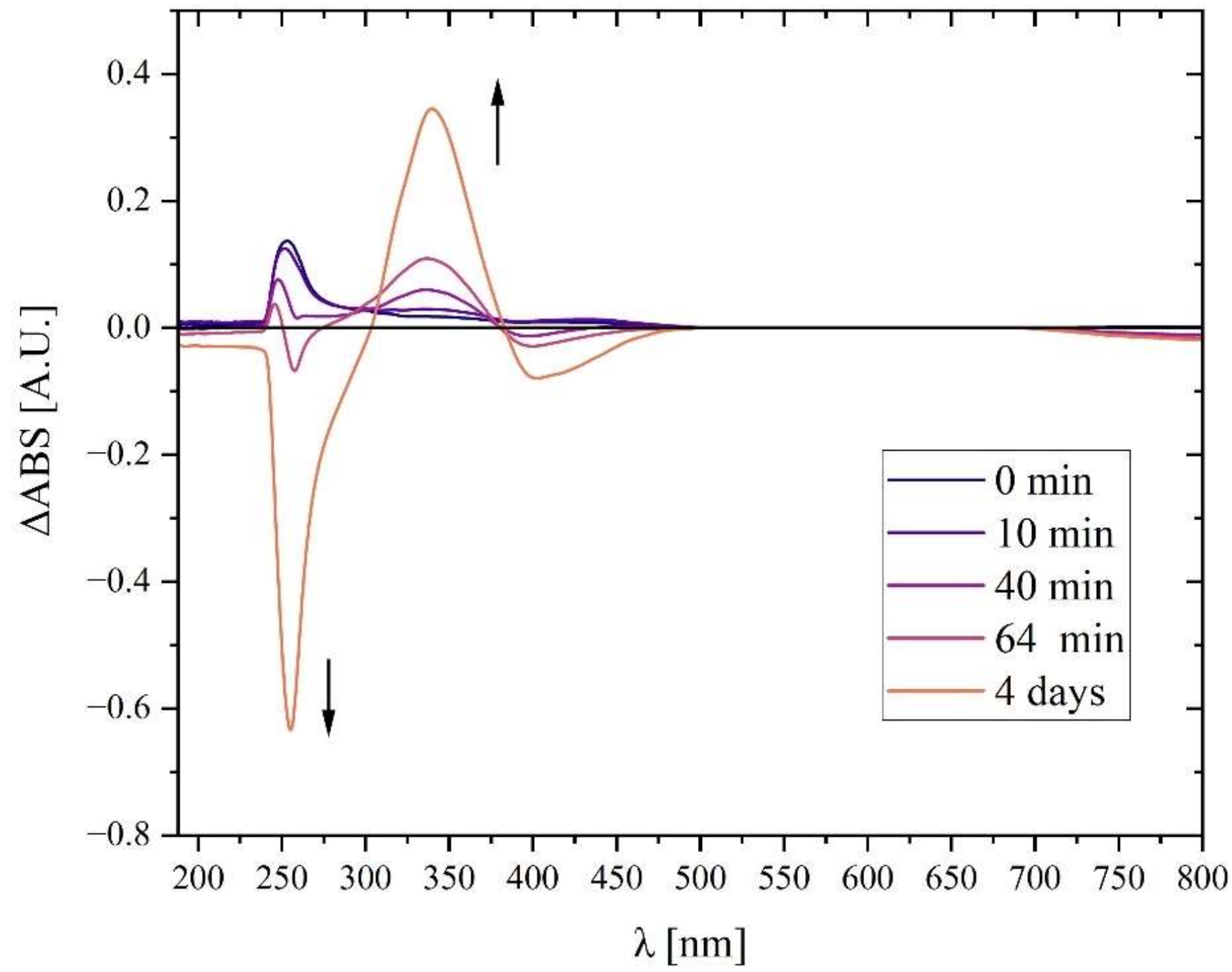

Figure S14. Reaction of DDQ (0.23 mM) and P_4 (0.47 mM), relative absorption ($A(t_0)$ = 0 min) monitoring over four days.

DDQ (22.7 mg, 10.0 µmol) was dissolved in MeCN (10.0 mL) to afford a 10 mM solution. To a quartz cuvette containing MeCN (2.0 mL), DDQ (1.0, 11.0, or 31.0 µL) and solid P_4 (2.4 mg) were added. The cuvette was closed and shaken vigourusly. After the remaining solid P_4 setteled the reaction was monitored per UV-Vis spectroscopy for a total of three days.

This experiment was performed to determine whether the UV–Vis profiles remain consistent in the absence of benzene and to examine the UV region that was obscured by the absorption of benzene in the preceding experiment.

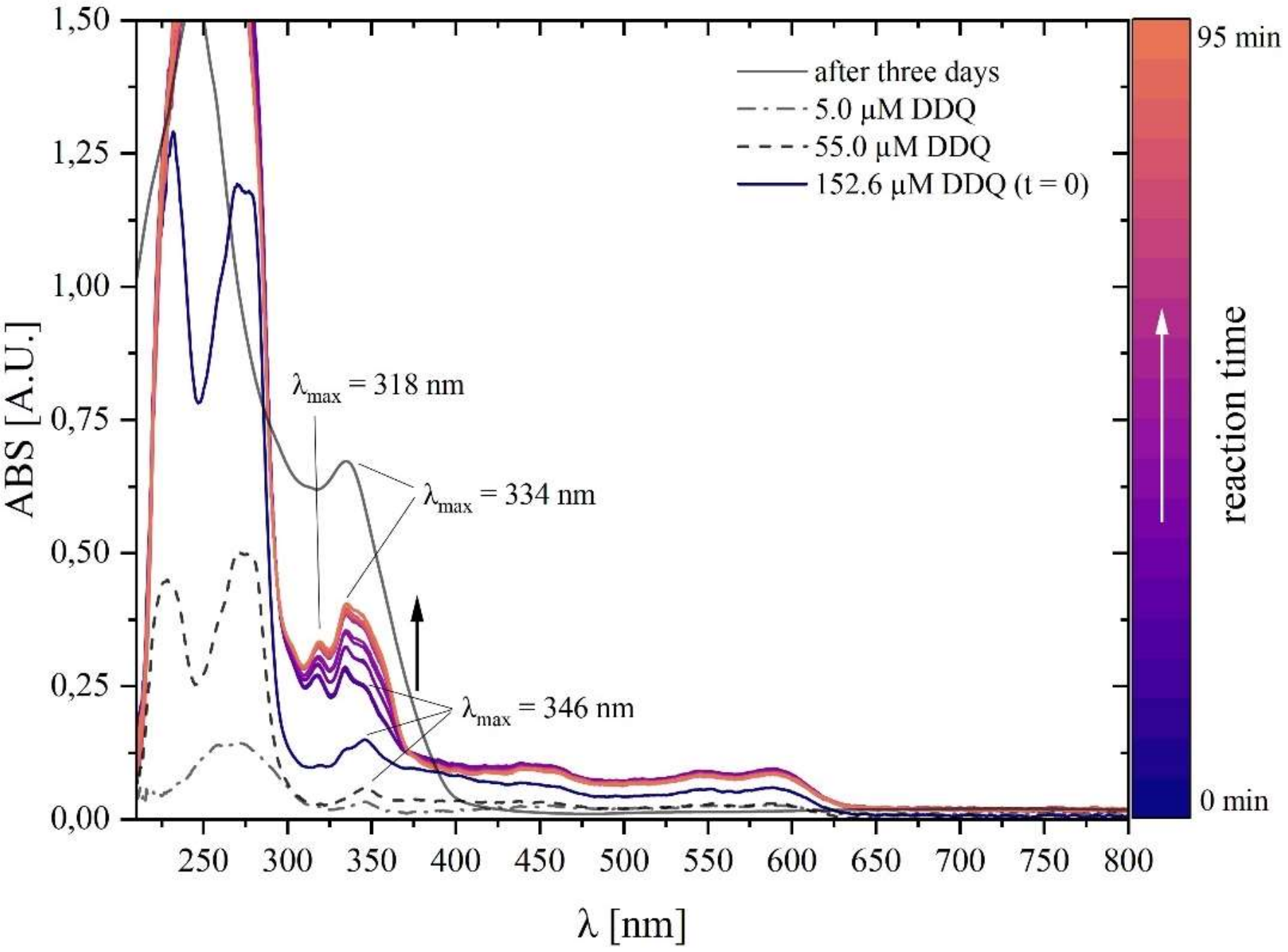

Figure S15. Reaction of DDQ (152.6 µM) and P_4 (solid, excess): Absorption monitoring over three days. Dashed lines: different concentrations of DDQ (5.0 µM, 55.0 µM) with excess P_4.

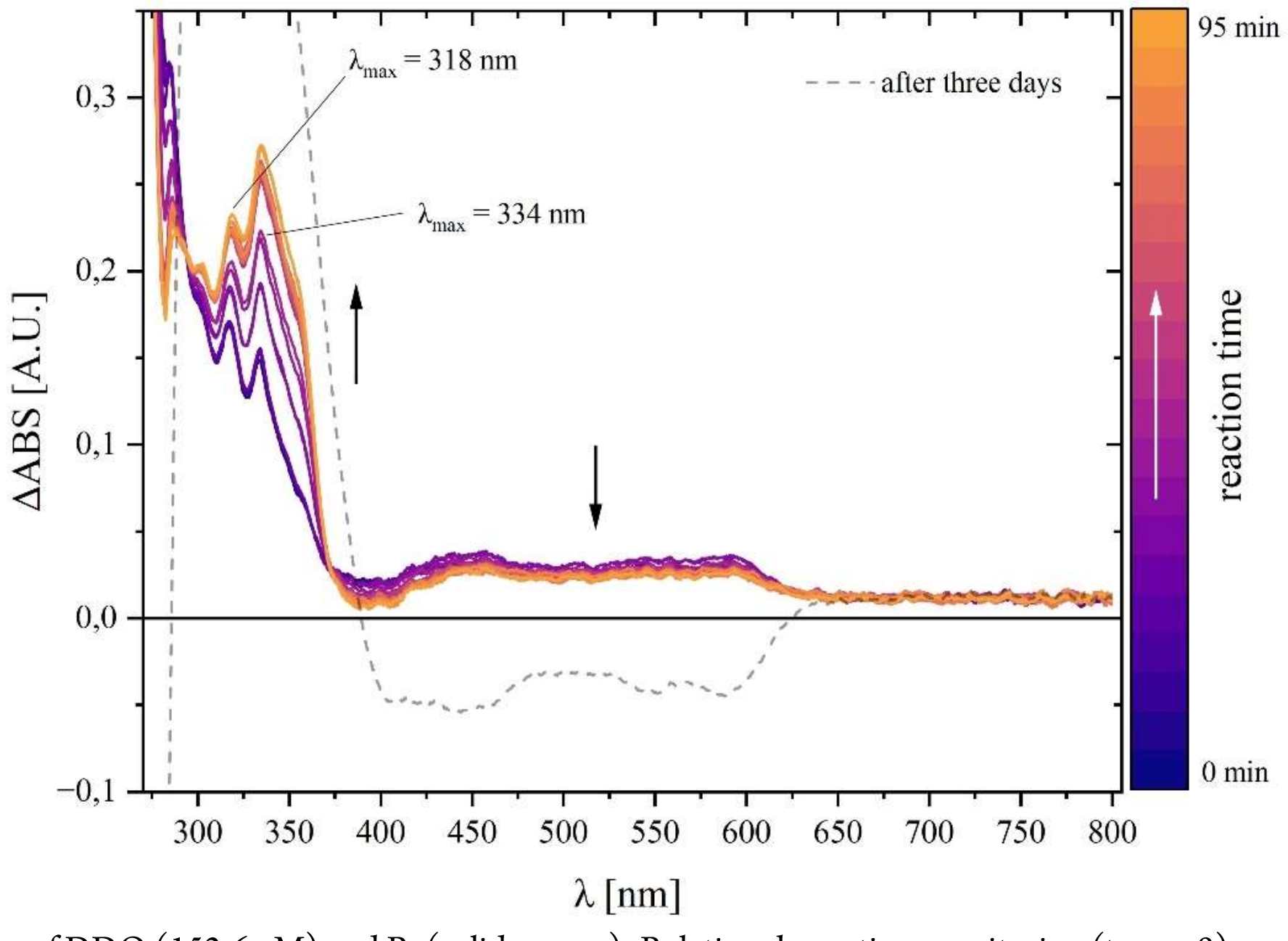

Figure S16. Reaction of DDQ (152.6 µM) and P_4 (solid, excess): Relative absorption monitoring ($t_{0\,min} = 0$) over three days.

3.9. Synthesis of K[DDQ·] via Reduction of DDQ with KI

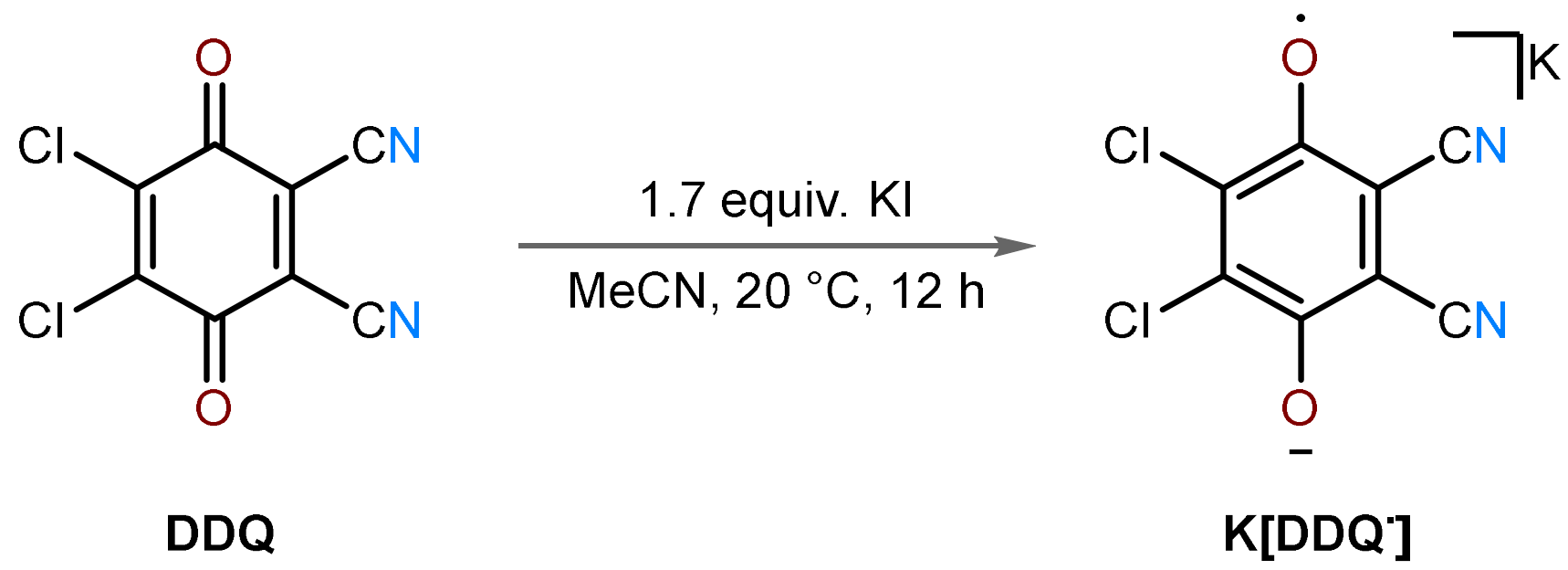

Scheme S7. Reaction conditions for the synthesis of K[DDQ·]

The procedure was adapted from Miller et al.[11]

DDQ (5.00 g, 22.03 mmol, 1.0 equiv.) and KI (6.22 g, 37.4 mmol, 1.7 equiv.) were dissolved in dry MeCN (100 mL) and stirred at 20 °C for 12 h. As the reaction mixture formed a thick slurry during the reaction, additional MeCN (50 mL) was added to maintain efficient stirring. The suspension was filtered and the residue washed with MeCN (3 × ca. 100 mL). Removal of the solvent in vacuo at 65 °C afforded a purple-brown solid, during which sublimation of elemental iodine was observed. The residue was further dried under dynamic vacuum at 65 °C for 4 h and subsequently washed with toluene until the filtrate ran clear. The remaining solid was redissolved in a minimal amount of MeCN. Storage of the solution at – 30 °C for 3 d afforded dark crystals. The supernatant was decanted, and the crystals were washed with precooled MeCN (3 × 50 mL, – 30 °C). K[DDQ·] was obtained in the form of dark green crystals in 19% yield (1.19 g).

Elemental Analysis found (calcd.) for $KC_8H_2O_2N_2Cl_2$: C 34.17 (36.11), H 0.61 (0.00), N 10.02 (10.53). The deviations in the elemental analysis values may arise from trace contamination by MeCN and iodine-containing byproducts such as I_2 or KI_3 formed during the reaction.[11]

Notes: Formation of elemental iodine during the reaction and drying process was confirmed qualitatively by rapid discoloration upon treatment with aqueous sodium thiosulfate solution. Care must be taken to keep both the crystal suspension and washing solvent below – 30 °C during workup, as significant amounts of K[DDQ·] dissolve at higher temperatures, resulting in substantial product loss.

3.10. Synthesis of $DDQH_2$ via Reduction of DDQ with $Na_2S_2O_4$

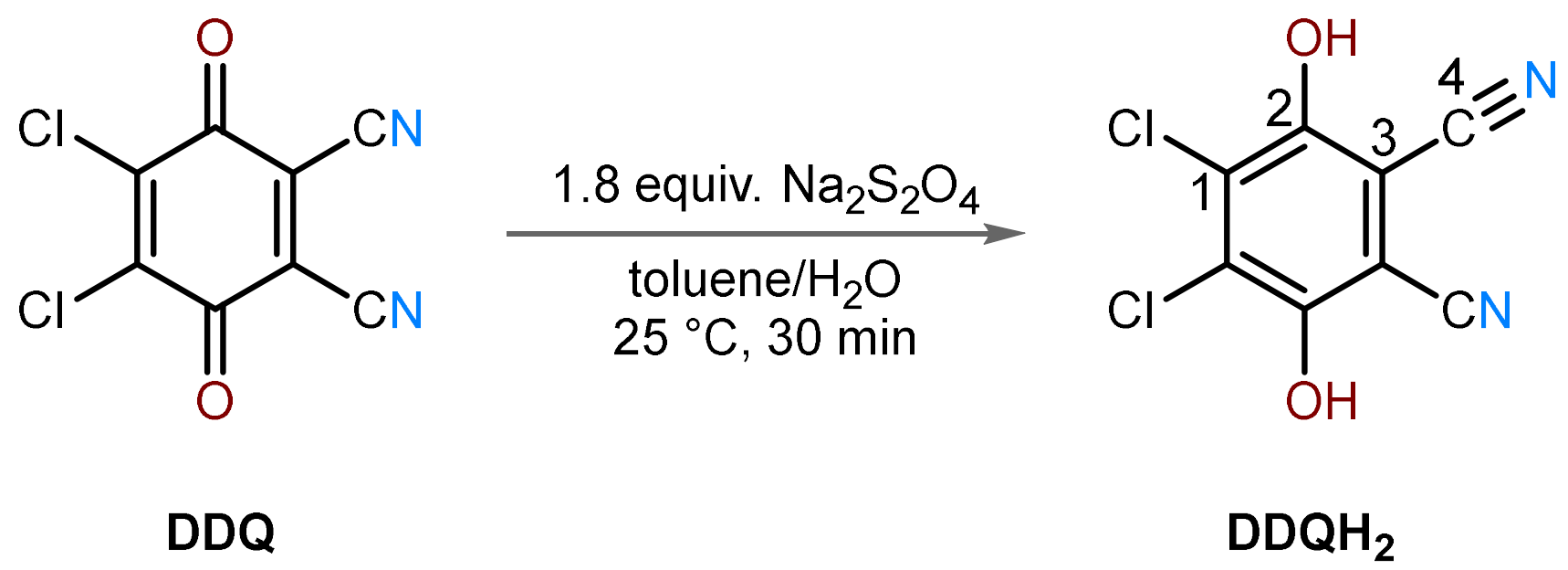

Scheme S8. Reaction conditions for the synthesis of $DDQH_2$. Carbon atoms are numbered for the NMR signals assignment.

The procedure was adapted from al-Raqa et al.[12]

At room temperature, a solution of sodium dithionite (3.375 g, 0.021 mol, 1.8 equiv.) in water (42.5 mL) was added slowly to a solution of DDQ (2.5 g, 0.012 mol, 1.0 equiv.) in toluene (50 mL). The mixture was filtered, and the remaining solid was washed with small portions of cold water (2 x 10 mL), followed by toluene (20 mL) and hexane (3 x 20 mL). The collected filtrate was evaporated to dryness. The obtained residue was further dried under vacuum at 65 °C overnight, giving $DDQH_2$ as an off-white solid in 71% yield (1.786 g). For ^{1}H and $^{13}C\{^{1}H\}$ NMR spectra with signal assignments, see Figure S53 Figure S55

^{1}H NMR (500.18 MHz, 298 K, THF-d_8) δ 10.26 (bs, OH) ppm.

$^{13}C\{^{1}H\}$ NMR (125.78 MHz, 298 K, THF-d_8) δ 101.99 (s, C(3)-CN), 112.27 (s, C(4)N), 127.91 (s, C(1)-Cl,), 150.58 (s, C(2)-OH) ppm.

Elemental Analysis found (calcd.) for $C_8H_2O_2N_2Cl_2$: C 4.83 (41.96), H 1.00 (0.88), N 12.14 (12.23).

3.11. Synthesis of P(DDQH)$_3$ from PCl$_3$ and DDQH$_2$

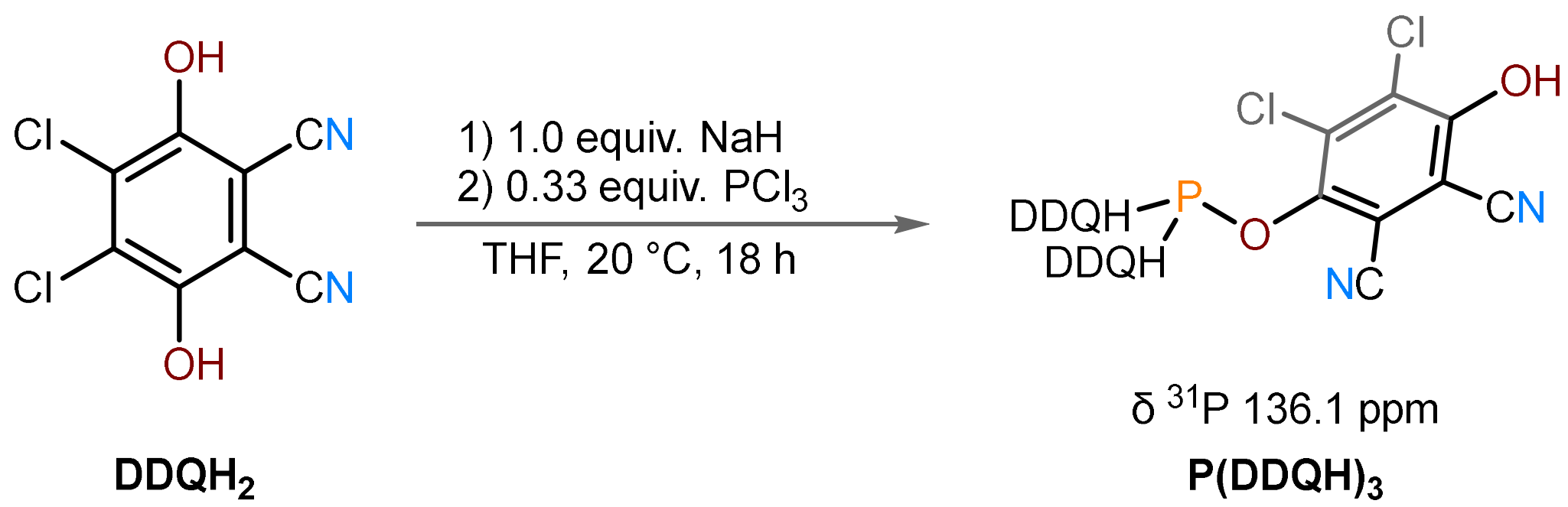

Scheme S9. Reaction conditions for the attempted synthesis of P(DDQH)$_3$

In a Schlenk flask, DDQH$_2$ (see section S3.10; 500 mg, 2.18 mmol, 1.0 equiv.) was dissolved dry THF (10 mL) the mixture was stirred ensuring complete dissolution. NaH (104.8 mg, 4.37 mmol, 1.0 equiv.) was added portion-wise. The mixture was stirred until gas evolution stopped and a bright orange precipitate formed (presumably Na[DDQH])[11]. PCl$_3$ (1.45 mL, 0.5 M in THF 0.73 mmol, 0.33 equiv.) was added dropwise. The mixture was stirred at 25 °C for 18 h. After this the suspension was filtered by canula and washed with THF (3 x 10 mL). The filtrate was concentrated and dried in vacuo. A concentrated solution of the crude mixture in THF-*d*$_8$ was prepared for NMR analysis (see section S4.9).

The reaction showed two new signals in the $^{31}P\{^1H\}$ NMR spectrum, assigned to P(DDQH)$_3$ (136.1 ppm, s) and one of the corresponding chlorophosphites (C$_2$PDDQH or ClP(DDQH)$_2$, 171.7 ppm) as a minor side product.[13,14] The ^1H NMR spectrum showed two broad resonances, which were assigned to the respective OH resonances of DDQH$_2$ (10.28 ppm; see Figure S53) and P(DDQH)$_3$. The $^{13}C\{^1H\}$ NMR spectrum showed the expected eight distinct carbon resonances expected for P(DDQH)$_3$, in addition to the signals associated with DDQH$_2$ (see Figure S55). These results indicate the formation of P(DDQH)$_3$; however, the presence of large amounts of DDQH$_2$ has thus far prevented isolation of the product.

3.12. Comments on the Proposed Structure of P(DDQH)$_3$

To gain insight into the nature of this phosphite-like species—specifically whether it is closed-shell and/or protonated—we attempted its synthesis via an independent route (see section S3.11). DDQ was first reduced using $Na_2S_2O_4$ in a toluene/water mixture to form the corresponding hydroquinone $DDQH_2$. The isolated product was then reacted with PCl_3 (0.33 equiv.) after deprotonation with NaH (1.0 equiv.) in THF for 18 h at 20 °C. The resulting mixture displayed the same ^{31}P NMR signal at δ 136.1 ppm as observed in the reaction of DDQ with P_4 in MeCN, as well as in the reaction of DDQ, P_4, and KF in DCM (Figures S58, S61 and S3) These observations support the conclusion that the same phosphorus-containing species is formed in all three reactions. The species was assigned as an aryl phosphite based on its ^{31}P{^{1}H} NMR chemical shift, as discussed in section S3.13 and in the main manuscript.

In principle, each reduced quinone unit can exist in one of three electronic states, namely as a negatively charged species, a neutral radical, or a protonated hydroquinone fragment. For a phosphite containing three such units, the total number of distinct compositions depends on the possible distributions of these states, irrespective of their positional arrangement. This corresponds to a combinatorial problem of distributing n indistinguishable units among k distinguishable states, which is given by the expression $N = \binom{n+k-1}{k-1}$. In the present case, $n = 3$ reduced quinone units and $k = 3$ possible states are considered, yielding $N = \binom{3+3-1}{3-1} = \binom{5}{2} = 10$. Thus, ten distinct compositions of phosphites with three reduced quinone units are possible in principle, encompassing all mixed combinations of negatively charged, radical, and protonated states (see Scheme S10)

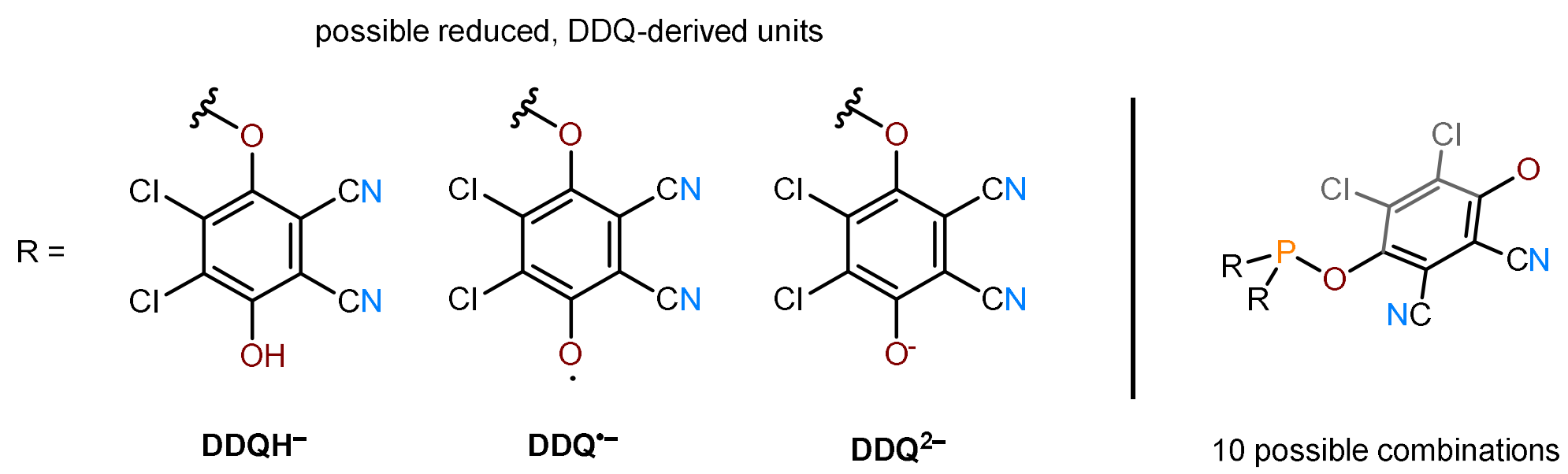

Scheme S10. Schematic representation of the possible reduced quinone units (anionic, radical, and protonated hydroquinone) and the conceivable phosphites formed from these building blocks.

However, the observation of a sharp $^{31}P\{^{1}H\}$ NMR resonance (δ 136.1 ppm, s, Figure S61) renders open-shell species unlikely. The remaining question is therefore whether the species is anionic (formal charge −1 to −3) or protonated (formal charge 0 to −2). As isolation of the phosphite has not yet been achieved, we pursued its synthesis via a traditional pathway by deprotonating $DDQH_2$ with NaH and reacting the mixture with PCl_3. A new major $^{31}P\{^{1}H\}$ NMR resonance was observed (δ 136.1 ppm, s, Figure S58) and the ^{1}H NMR spectrum showed a broad resonance at δ 11.11 ppm which we assigned to the OH protons of the phosphite (Figure S56). This provides evidence that the species is best described as a protonated tris(hydroquinonyl) phosphite, $P(DDQH)_3$ (Scheme S11). The protons in the reaction between P_4 and DDQ presumably originate from traces of moisture in the reaction mixture or reagents (see section S3.13).

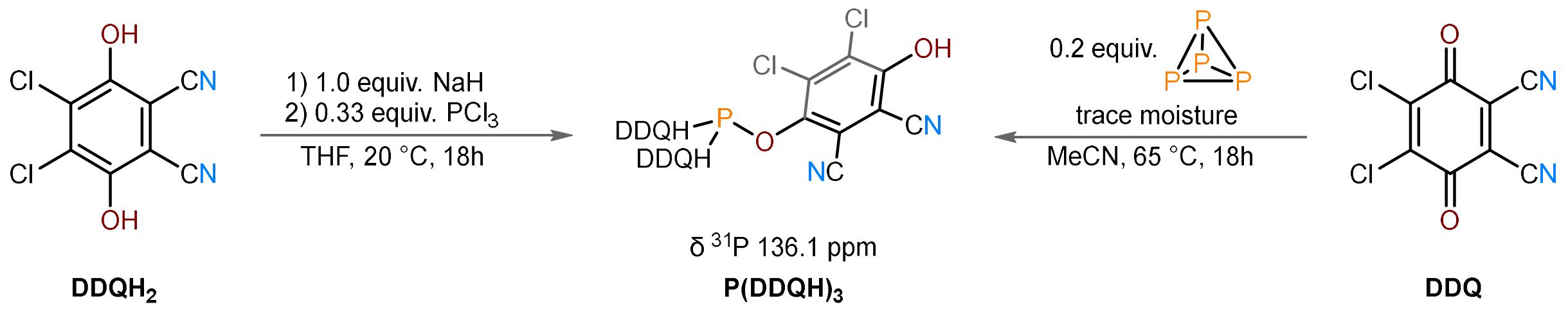

Scheme S11. Convergent formation of $P(DDQH)_3$: direct reaction of P_4 with DDQ and a base-mediated reaction of $DDQH_2$ with PCl_3 both lead to the same major phosphite species, $P(DDQH)_3$, as identified by $^{31}P\{^{1}H\}$ NMR spectroscopy (δ 136.1 ppm, s).

Based on our results, the formation of alternative species containing negatively charged or radical quinone units cannot be excluded. However, such species would likely not be detectable by ^{31}P NMR spectroscopy owing to their open-shell character and/or poor solubility. Accordingly, the observed $^{31}P\{^{1}H\}$ NMR signal is attributed to $P(DDQH)_3$.

3.13. Studies on the Formation of P(DDQH) 3

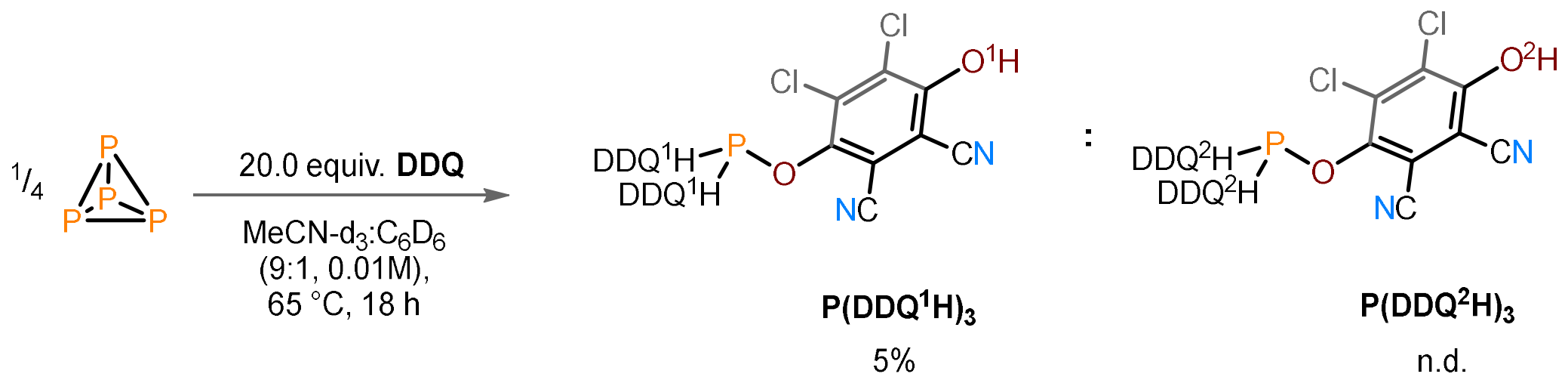

Scheme S12. Reaction of P_4 with DDQ in deuterated solvents to identify the proton source in $P(DDQH)_3$ formation using, showing no deuterium incorporation by ^2H NMR spectroscopy.

According to general procedure, P_4 and DDQ in MeCN-d_3:C_6D_6 (9:1, 1.0 mL) were heated for 18 h at 65 °C. The mixture was then filtered and analyzed by NMR.

To investigate the origin of the protons incorporated into $P(DDQH)_3$, reactions were conducted in deuterated solvents. DDQ and P_4 were heated in a MeCN-d_3:C_6D_6 (9:1) mixture at 65 °C for 18 h in the presence of TPPO as an internal standard. $^{31}P\{^1H\}$ NMR shows a new resonance at δ 136.1 ppm (s), which we assign to a phosphite-like adduct, $P(DDQH)_3$. This assignment is supported by literature data for related phosphite species, which exhibit comparable chemical shifts (e.g., $P(OEt)_3$: δ 139.2 ppm; $P(OPh)_3$: δ 128.1 ppm)[3,7]. Similar signals observed in the solvent-screening experiments (DCM, Table S2, entry 7) were likewise attributed to this phosphite-like species. $P(DDQH)_3$ was formed in 5% yield alongside 58% conversion of P_4 (Figure S61, by $^{31}P\{^1H\}$ NMR integration against TPPO). ^1H NMR analysis showed two broad resonances (Figure S59), assigned to O–H protons of the phosphite species (δ 9.34 ppm) and $DDQH_2$ (δ 8.53 ppm; confirmed by dissolving $DDQH_2$ (section S3.10) in MeCN-d_3 which also gives a broad signal at 8.34 ppm, Figure S54) Importantly, ^2H NMR analysis did not show any corresponding O–D resonance, indicating that no detectable deuterium incorporation had occurred during the reaction (Figure S60). The observed spectroscopic data are consistent with independently synthesized $P(DDQH)_3$, obtained by deprotonating $DDQH_2$ (1.0 equiv.) in situ with NaH (1.0 equiv.) and reacting this mixture with PCl_3 (0.33 equiv.; see section S3.11). Taken together, these experiments demonstrate that the proton incorporated into $P(DDQH)_3$ does not seem to originate from the solvent. Instead, we believe that traces of water in the reaction mixture represent the most plausible proton source, despite the use of thoroughly dried solvents, KF, and P_4. Moisture is most likely associated with DDQ. Traces of water do not have a noticeable effect on the fluorination of P_4.

3.14. Studies on Reactivity of P 4/DDQ Reaction Mixture with KF

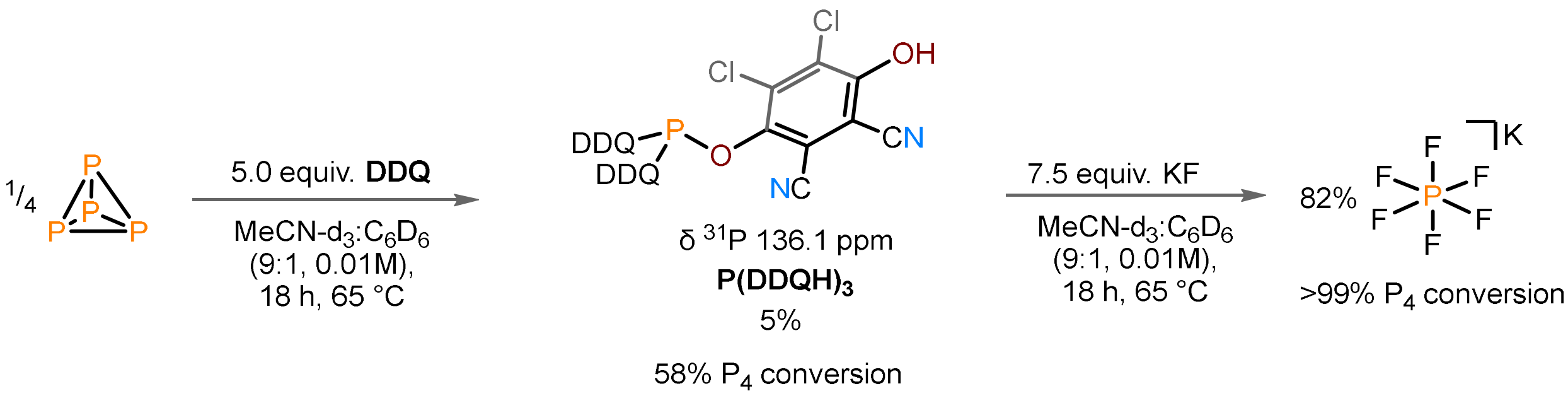

KF (17.4 mg, 0.3 mmol, 7.5 equiv.) was added to the crude reaction mixture from section S3.13 and the mixture was heated at 65 °C for 18 h. The mixture was then filtered and analyzed by NMR.

This experiment was conducted to probe the fate of the missing phosphorus intensity in the reaction between P_4 and DDQ (section S3.13) as determined by quantitative $^{31}P\{^1H\}$ NMR. While $P(DDQH)_3$ was only observed in 5% yield, 58% conversion of P_4 occurred, leaving 53% of the phosphorus intensity unaccounted for. Addition of KF (7.5 equiv.) to the crude reaction mixture (section S3.13) followed by heating at 65 °C for 18 h afforded KPF_6 in 85% yield alongside some minor unidentified fluorination products, as determined by $^{31}P\{^1H\}$ and $^{19}F\{^1H\}$ NMR analysis (Figures S62 and S63). These results indicate that the remaining phosphorus-containing species are largely NMR-silent but remain chemically active intermediates in the fluorination reaction, with $P(DDQH)_3$ representing only the fraction observable by $^{31}P\{^1H\}$ NMR (see section S3.12 for comments on possible intermediates).

3.15. Reaction of K[DDQ˙] with P_4

According to the general procedure, K[DDQ˙] (0.2 mmol, 5.0 equiv.) was treated with P_4 under conditions specified in Table S18.

Table S18. Reaction of K[DDQ˙] with P_4

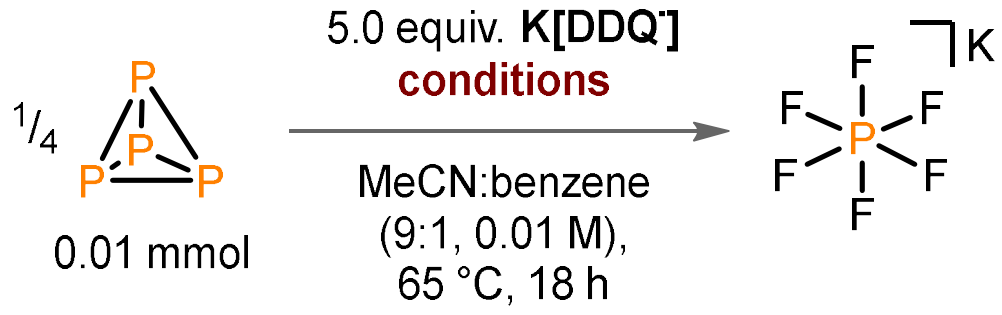

Entry	conditions	Conversion P_4 [%]
1	-	>99
2	7.5 equiv. KF	48

Treatment of K[DDQ˙] (5.0 equiv.) with P_4 (0.25 equiv.) in MeCN/benzene at 65 °C for 18 h resulted in an NMR silent reaction mixture. No signals were detected in this mixture by $^{31}P\{^1H\}$ NMR spectroscopy. Performing the same reaction in the presence of KF resulted in only 48% conversion of P_4, but only traces of unidentified fluorinated phosphorus compounds were observed (Figure S64–Figure S66). These results show that K[DDQ˙] is capable of oxidizing P_4, but is largely inactive in the fluorination reaction.

3.16. Variation of the DDQ/P_4 Ratio

According to the general procedure, Y. Joung NMR tubes were charged with different volumes of the DDQ stock solution (0.213 M in MeCN, see Table S19). MeCN was then added to bring the total volume to 1.2 mL in each sample.

Table S19. Screening of different P_4:DDQ stoichiometries in MeCN by varying the amount of DDQ

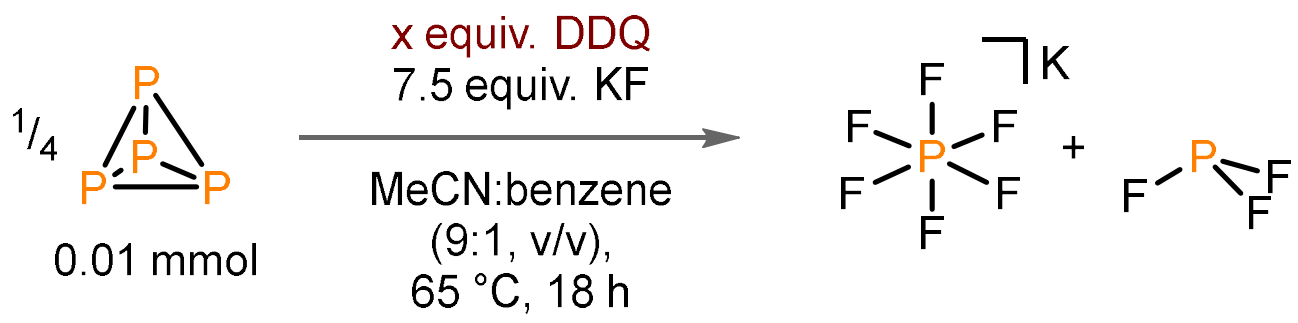

Entry	equiv. P_4 vs. equiv. DDQ	V(DDQ) [µL]	c(DDQ) [mM]	Yield KPF_6 [%]	Yield PF_3 [%][a]	Conversion P_4 [%]
1	1:2	92.2	16.7	n.d.	n.d.	41
2	1:4	184.3	33.3	n.d.	trace	55
3	1:6	276.5	50.0	n.d.	8	71
4	1:8	368.7	66.7	trace	7	88
5	1:10	460.8	83.3	trace	trace	98
6	1:12	553.0	100.0	trace	trace	>99
7	1:14	645.2	116.7	64	n.d.	>99
8	1:16	737.3	133.3	84	n.d.	>99
9	1:20	921.7	166.7	95	n.d.	>99

3.17. UV-Vis Studies on the Solvent-Dependent Reactivity of DDQ

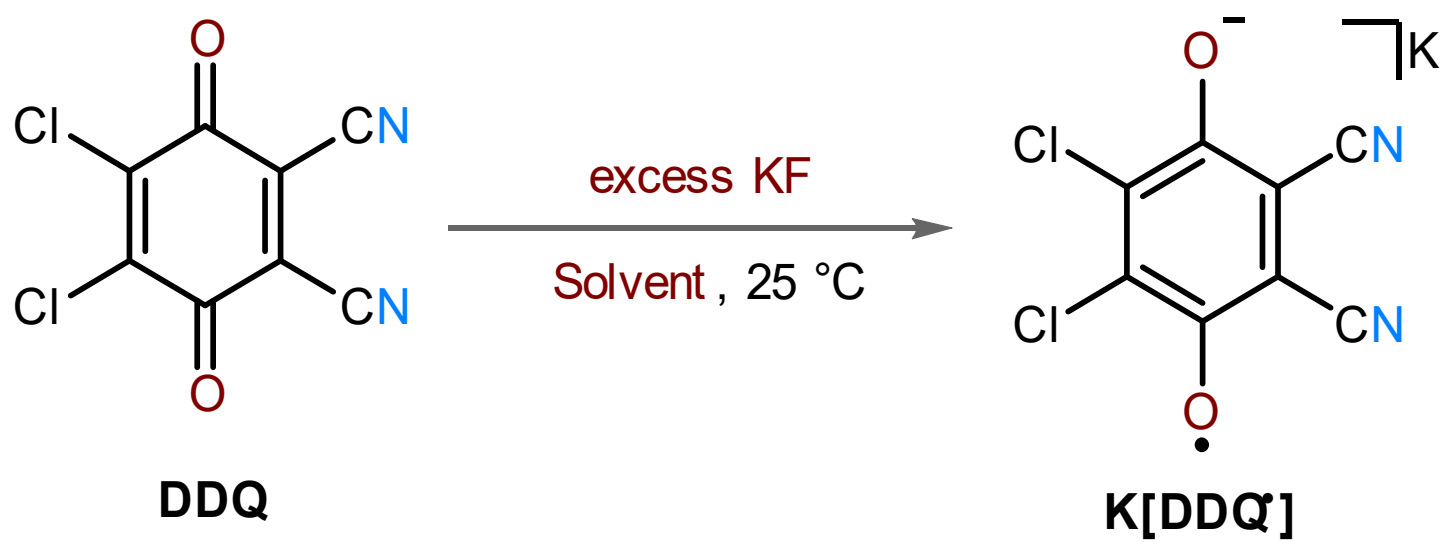

Scheme S13. Reaction conditions for UV–Vis studies of the reaction between DDQ and KF in different solvent mixtures.

Solutions of DDQ (10 mM) were prepared in MeCN and DCM (see previous UV-Vis experiments, section S3.6 – S3.8). Aliquots of these solutions were titrated into a quartz cuvette containing 2 mL of the respective solvent until a suitable absorbance was achieved. The solvents used were MeCN, DCM, or a mixture of the chosen solvent and TEG (10% v/v). To investigate the effect of fluoride, an excess of anhydrous KF (10 mg, 0.17 mmol) was added to the cuvette, which was then capped, vigorously shaken, and allowed to react for 5 min before recording the spectra. The formation of the DDQ^{-} was confirmed by comparison with SEC data (section S3.6).

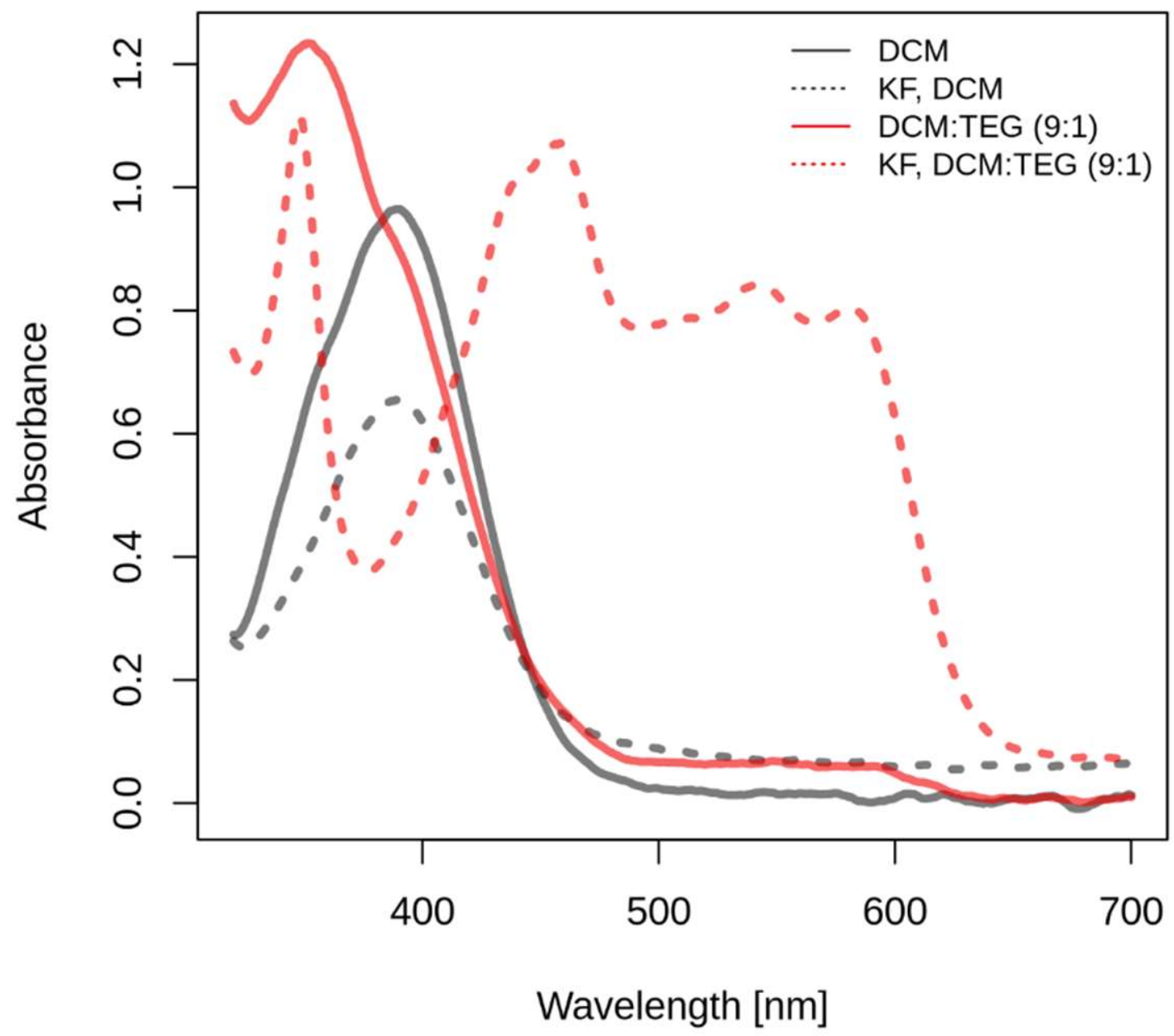

Figure S17. UV–Vis spectra of DDQ in DCM and in a DCM/TEG mixture (9:1). No fluoride interactions are observed in pure DCM, whereas pronounced interactions appear upon addition of TEG, highlighting the critical role of fluoride solubility. In line with this observation, the fluorination of P_4 in the DCM/TEG mixture proceeds selectively to K[PF_5TEG] (Table S15), whereas in DCM it halts at PF_3 (Figure S3).

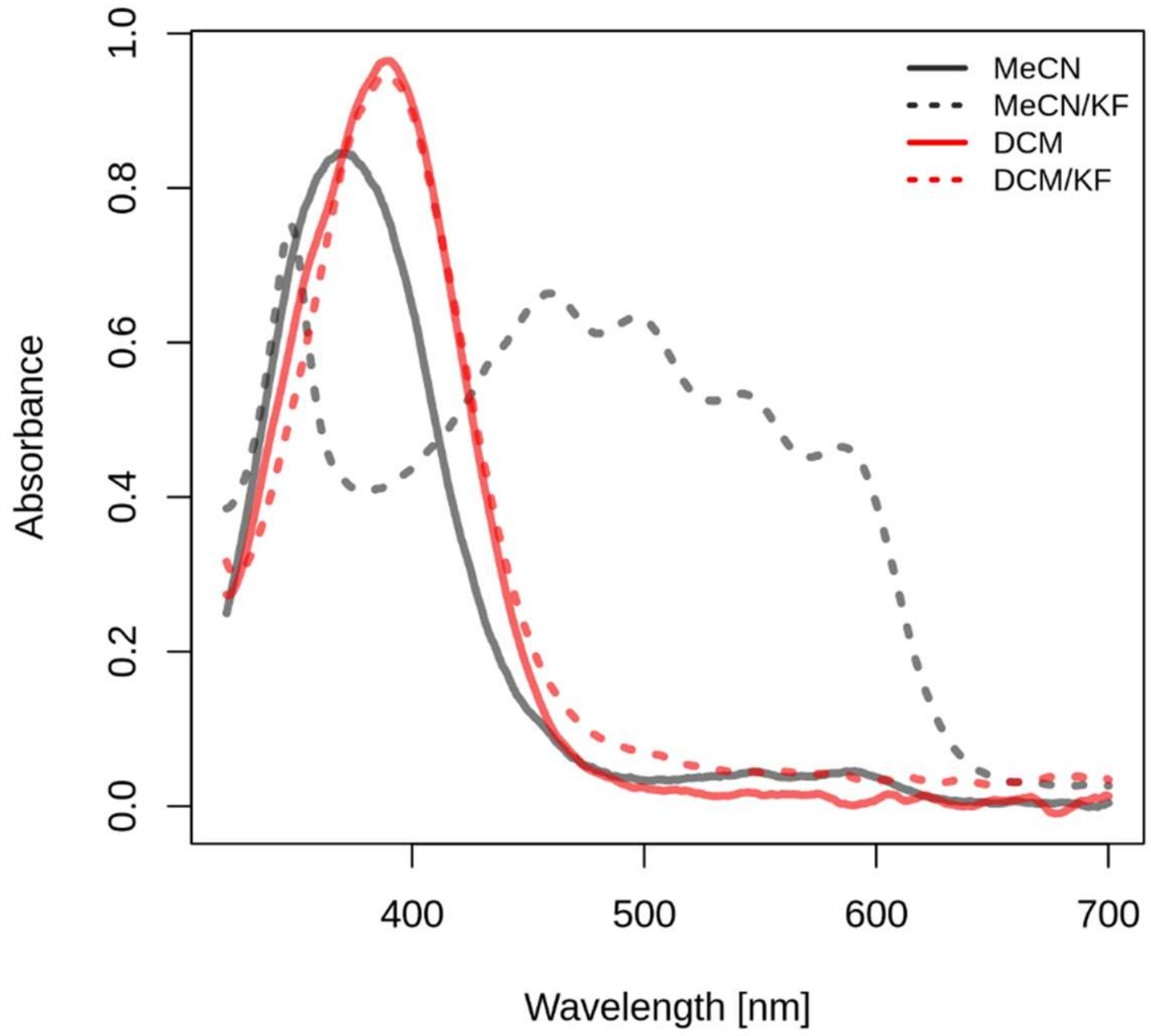

Figure S18. UV–Vis spectra of DDQ in MeCN and DCM. Upon addition of KF, clear fluoride interactions are observed in MeCN, whereas no spectral changes occur in DCM due to the absence of such interactions.

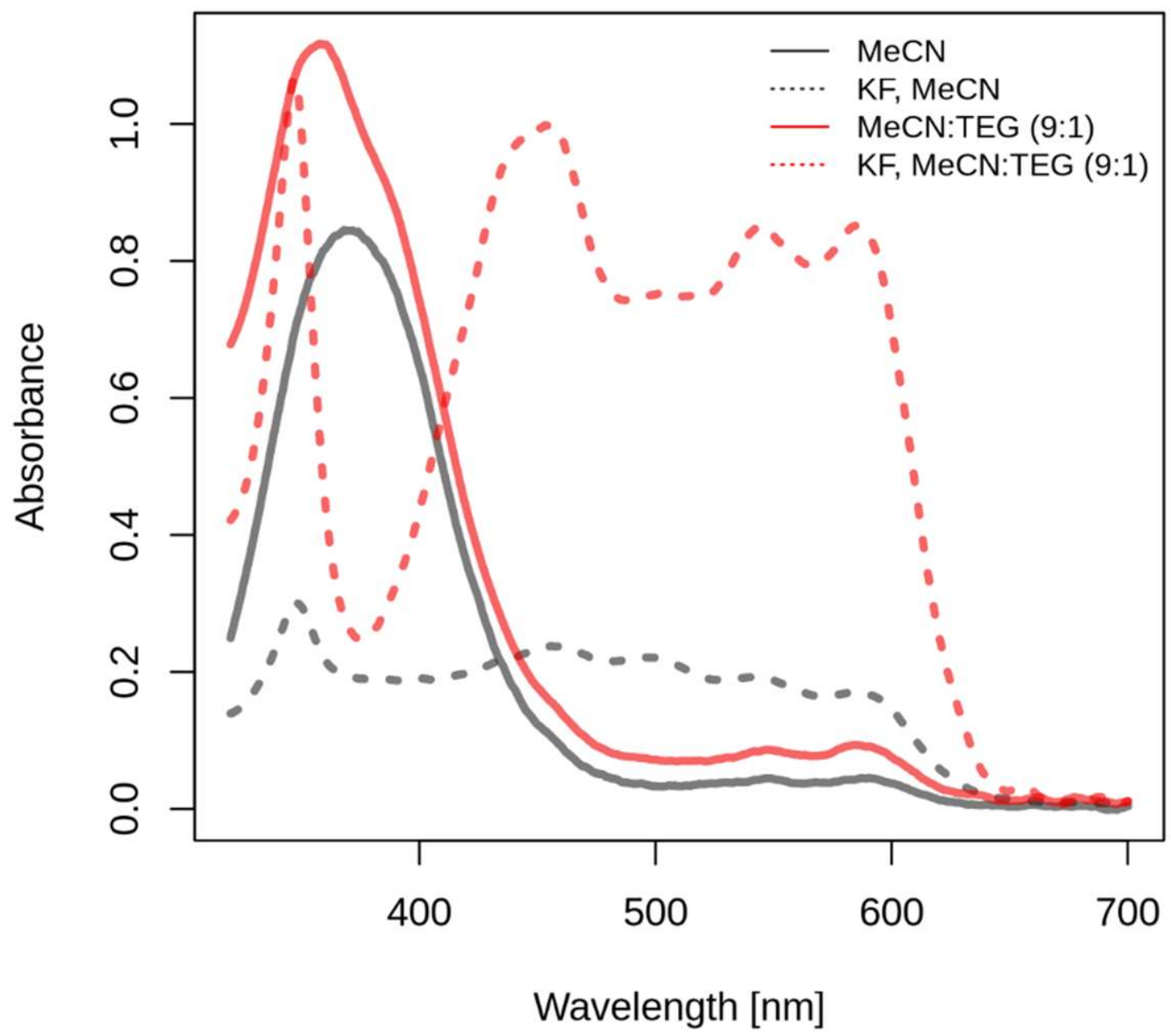

Figure S19. UV–Vis spectra of DDQ in MeCN and in a MeCN/TEG (9:1) mixture. Fluoride interactions are observed in both solvents, although the concentration of the resulting $DDQ^{\cdot-}$ is significantly higher in MeCN/TEG, reflecting more facile interactions due to enhanced fluoride solubility.

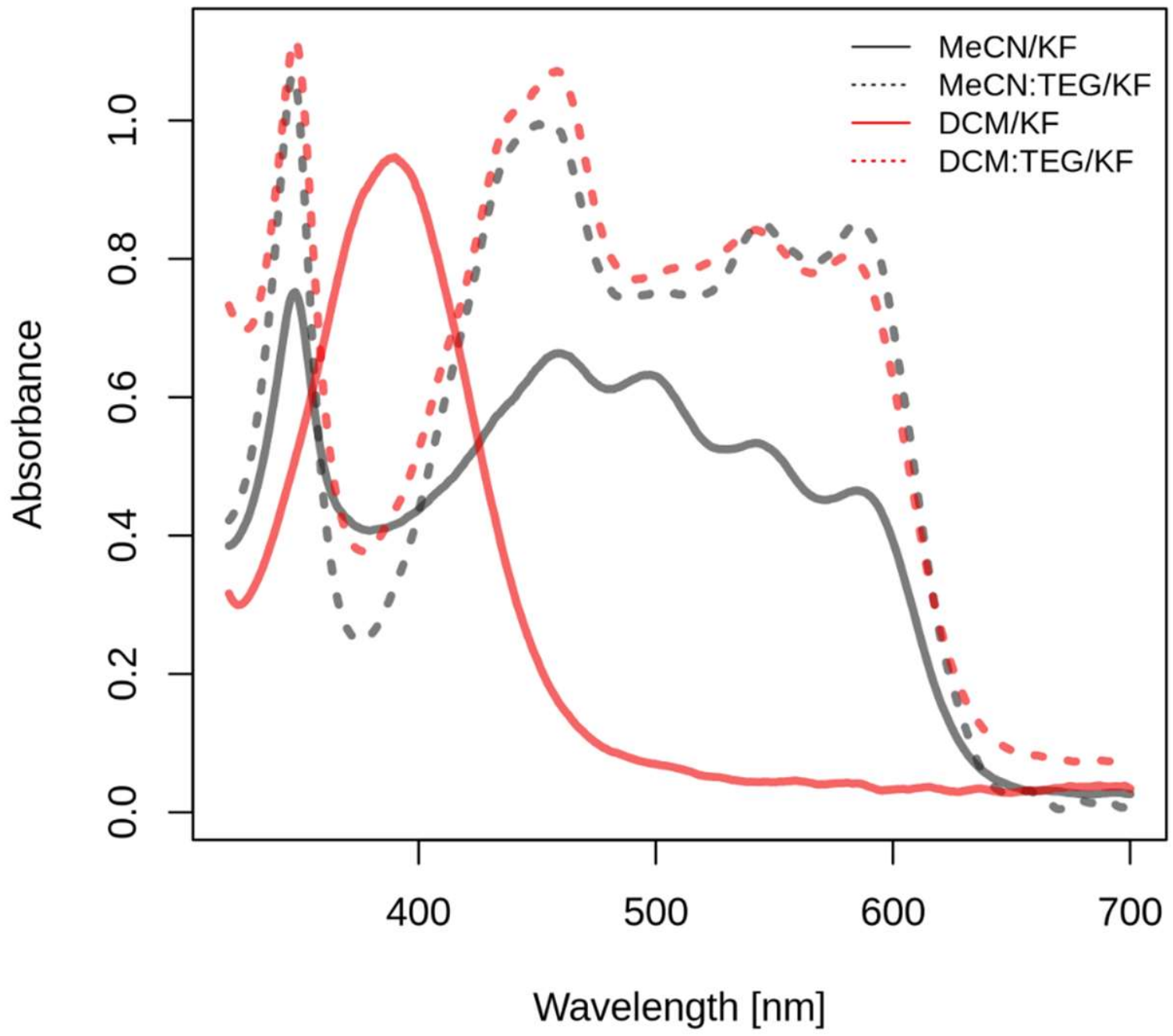

Figure S20. UV–Vis spectra of DDQ with excess fluoride added in all previously discussed solvent systems, demonstrating that fluoride solubility governs fluoride interactions. Only in DCM, where fluoride solubility is low, are no interactions observed.

3.18. In-situ UV-Vis Reaction Monitoring

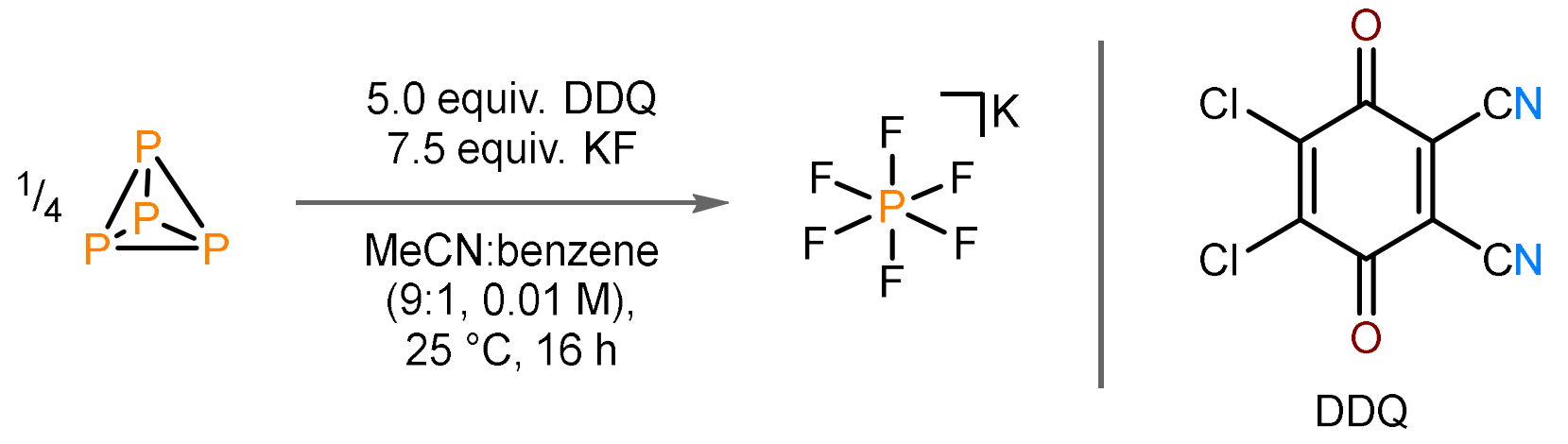

Scheme S14. Reaction conditions for in-situ UV–Vis monitoring of the DDQ-mediated fluorination of P_4.

The reaction mixture was prepared according to the general procedure, except that no internal standard was used. The mixture was diluted 200-fold with a MeCN/benzene (9:1) solution and filtered into a quartz-glass thin-layer cuvette (d = 1.0 mm). The reaction was monitored by UV–Vis spectroscopy over 16 h.

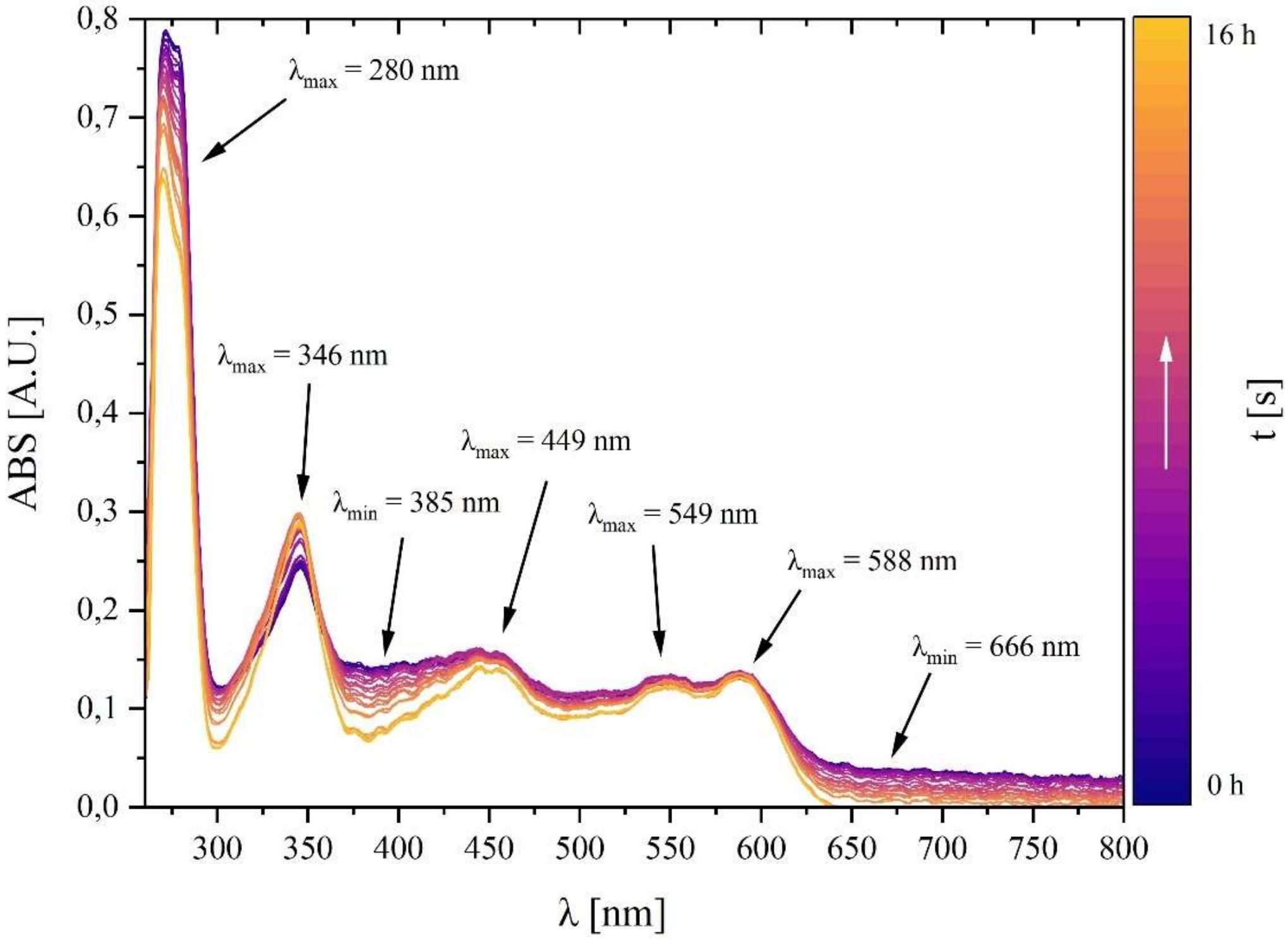

Figure S21. UV–Vis reaction monitoring of the DDQ-mediated fluorination of P_4 over 16 h.

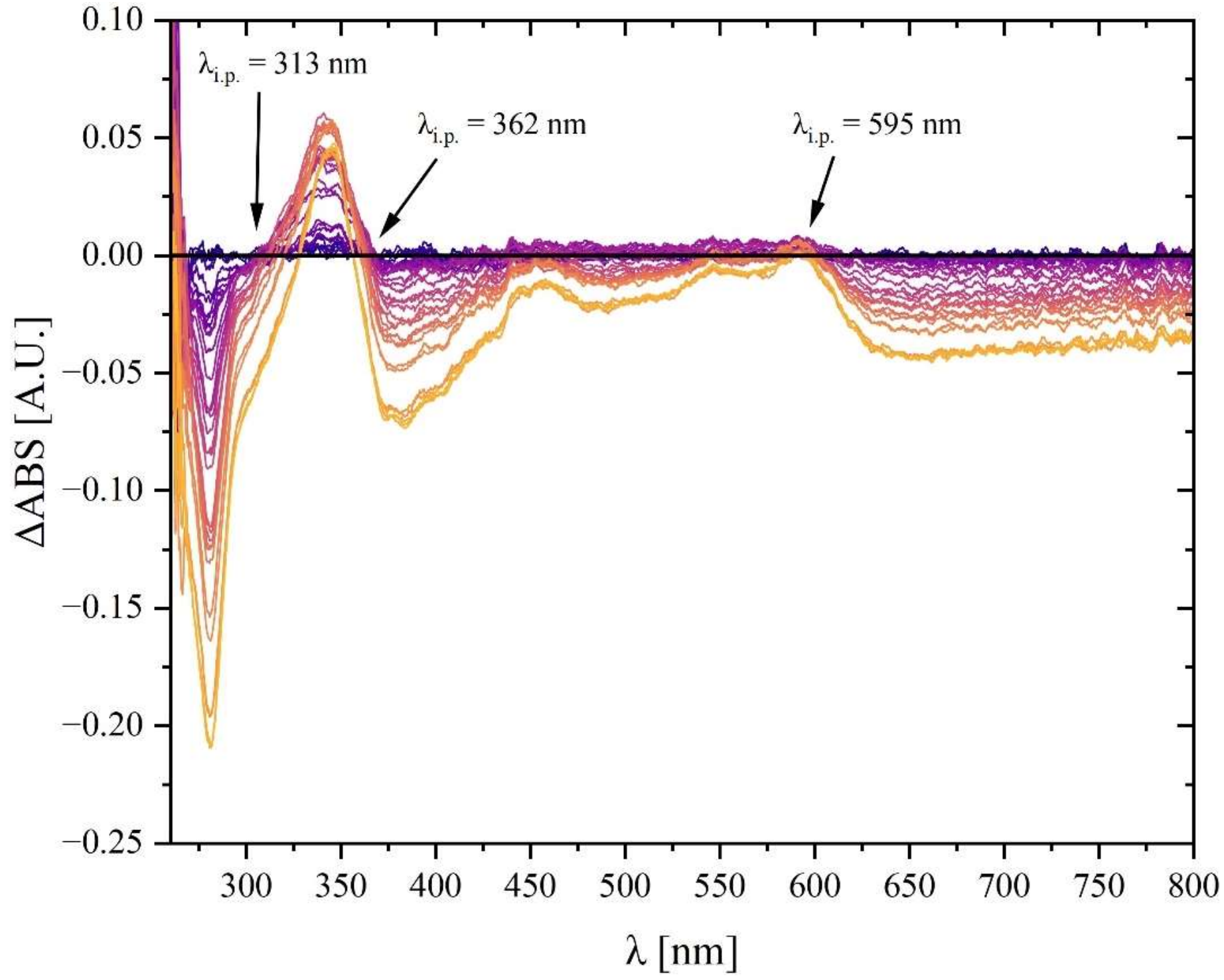

Figure S22. Difference UV-Vis reaction monitoring (absorbance at $t_0 = 0$) of the DDQ mediated fluorination of P_4 over 16 h.

3.19. In situ NMR Reaction Monitoring Experiments

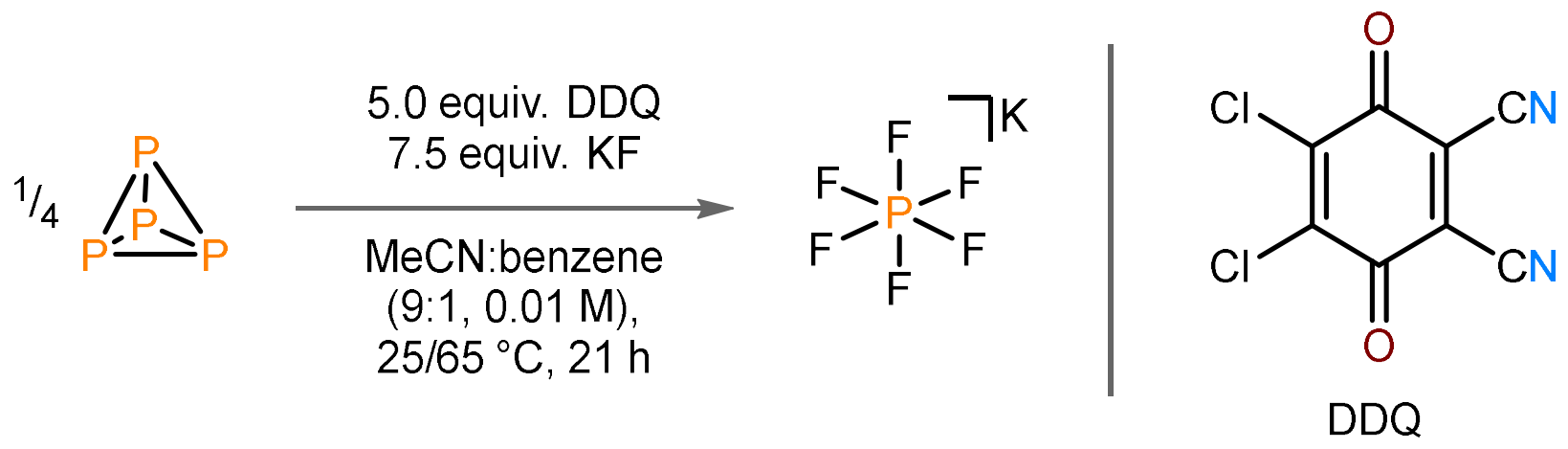

Scheme S15. Reaction conditions for the in-situ NMR monitoring of the DDQ-mediated fluorination of P_4.

According to the general procedure, a sample was prepared in a screw-cap NMR tube and measured on a Bruker Avance 400 NMR spectrometer equipped with a 5 mm PABBO BB/^{19}F–^{1}H probe head with Z-gradient. The specific experimental parameters are summarized in the table below. Integral traces obtained under different conditions are shown for the NMR monitoring experiments, with the relevant species marked. The resulting NMR spectra are displayed in Figure S68–Figure S72

Table S20. NMR Spectrometer settings for the reaction monitoring.

Nucleus	^{19}F	^{31}P
Experiment	^{19}F{^{1}H}	^{31}P{^{1}H}
Spectrometer Frequency	376.46 MHz	161.98 MHz
Temperature	25/65 °C	25/65 °C
Reciever Gain	208.1	208.1
Acquisition Time	1.2583 s	0.2490 s
Relaxation Delay	1.0000 s	2.0000 s
Pulse Width	16.5000 µs	15.5000 µs
Number of Scans	32	128

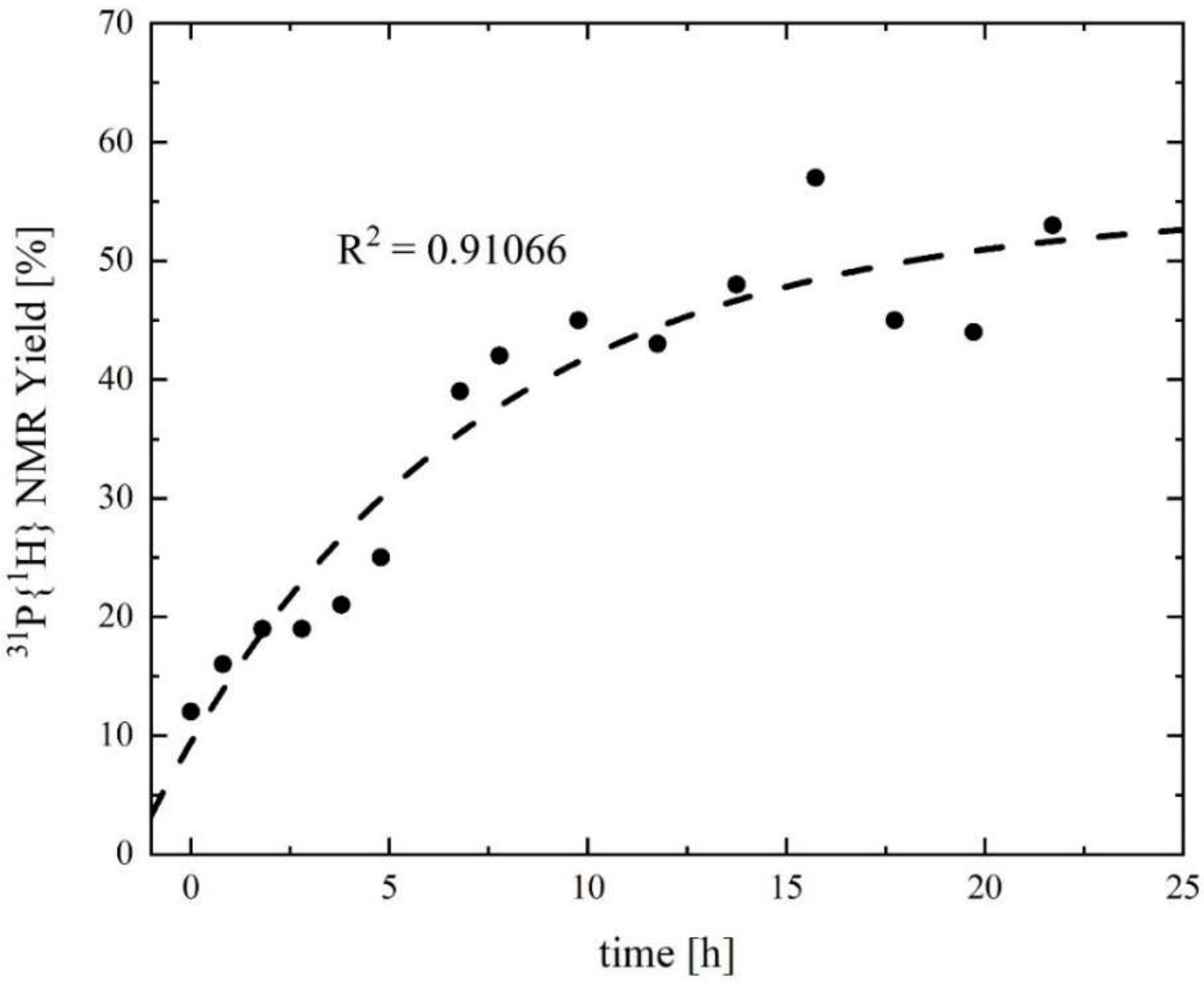

Figure S23. $^{31}P\{^{1}H\}$ NMR Yield of KPF_6 plotted against time. Data points fitted with first order rate law. For details see Figure S68.

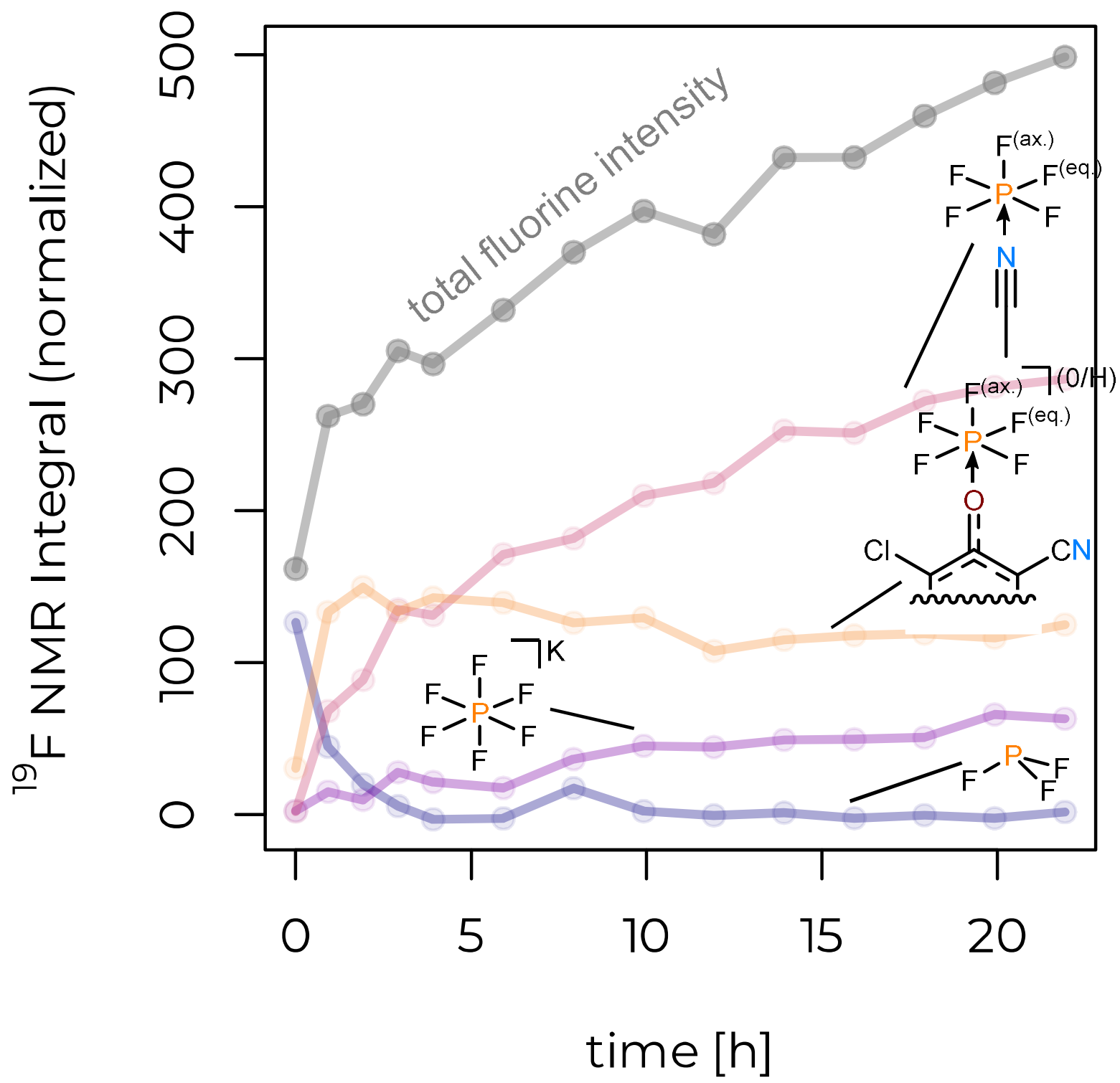

Figure S24. $^{19}F\{^{1}H\}$ NMR integrals of relevant species plotted over time, showing that the partially overlapping signals associated with the PF_5–LB species exhibit two independent curves evolving over time, indicating that at least two distinct species contribute to these signals. Total fluorine intensity increases as the reaction progresses, indicating a gradual transformation from insoluble KF[15] to soluble fluorophosphates. For details, see Figure S71.

3.20. Diffusion Ordered Spectroscopy (DOSY) Studies of K[PF $_5$·TEG]

The DOSY measurements were performed using the convection-suppressing DSTE (double stimulated echo) pulse sequence developed by Jerschow and Müller in a pseudo-2D mode.[20] No TMS was used because of possible reactions with the measured mixture. Therefore, the volume measurements are qualitative rather than quantitative. Referencing was done with the dynamic viscosity of acetonitrile as a solvent (0.334E-03 kg/(m·s)).[16]

The signal intensities of these groups in the DOSY spectra were classically analyzed as a function of the gradient strength by the software in the Bruker TopSpin 3.2 T_1/T_2 relaxation package by employing the Stejskal-Tanner equation (eq. 6).[17]

$$I = I_0 \cdot exp\left[-\gamma^2 G^2 \delta^2 \left(\Delta - \frac{\delta}{3}\right) D\right] \qquad \text{(eq. 6)}$$

where I is the signal with the gradient, I_0 is the signal intensity without diffusion weighting, γ is the gyromagnetic ratio, G is the strength of the gradient pulse, δ is the duration of the pulse, Δ is the time interval between the two pulses and D the diffusion coefficient.

estimated following the Stokes-Einstein equation (eq. 7), with D_i = self-diffusion coefficient k_B = Boltzmann constant, T = temperature, η = viscosity of the sample, c = correcting factor, F = shape factor:[18]

$$D_i = \frac{k_B T}{F c \pi \eta r_H} \qquad \text{(eq. 7)}$$

The shape factor F was set to 1 for a spherical shape. The semi-empirical modification by Chen (eq. 8) was used to calculate the correction factor c. The value for the solvent radius (r_{MeCN} = 2.12 Å) was taken from literature.[19–21]

$$c_{Chen} = \frac{6F}{1 + 0.695\left(\frac{r_{MeCN}}{r_H}\right)^{2.234}} \qquad \text{(eq. 8)}$$

The viscosity values for the solvent were taken from the literature.[16]

$$\eta\ [kg/sm] = 0.332 \cdot 10^3$$

After including all correction and calibration equations in the Stokes equation (eq. 9), the hydrodynamic radii r_H can be iteratively calculated. The corresponding volumes V_A were calculated with the assumption of a spherical shape.

$$D = \frac{kT\left(1 + 0.695\left(\frac{r_{MeCN}}{r_H}\right)^{2.234}\right)}{6\pi\eta r_H} \quad \text{(eq. 9)}$$

The experimental self-diffusion coefficients D_i, the viscosity corrected hydrodynamic radii r_H and the resulting volumes V_A of all samples are depicted in the following tables. The average D_i values were derived by using all baseline separated signals that were referring to the same species.

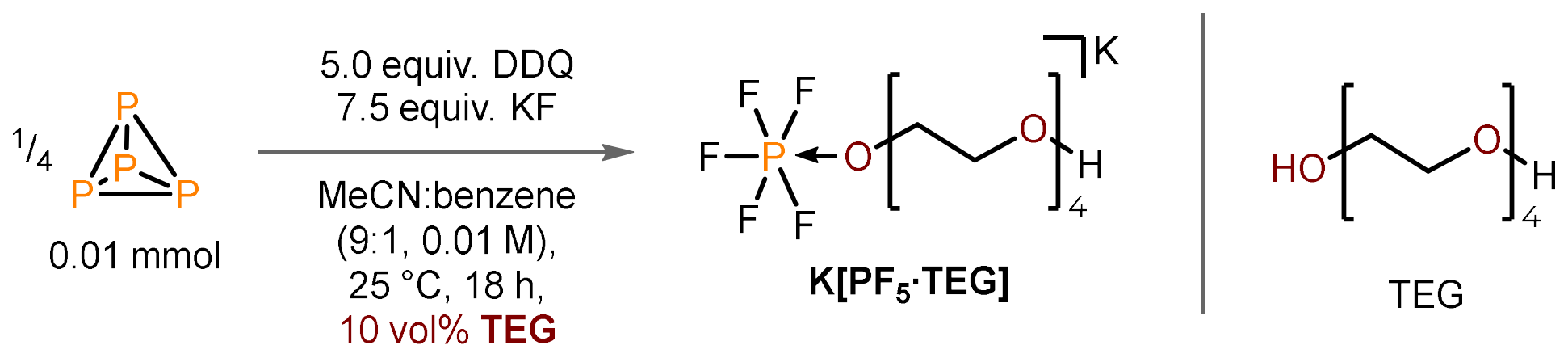

Scheme S16. Reaction conditions for DOSY Sample 1.

DOSY Sample 1 was prepared according to the general procedure, with the addition of tetraethylene glycol (10 vol%, 100 µL) to give a total volume of 1.0 mL and a final composition of 8:1:1 MeCN-d_6 / benzene / TEG (v/v). The sample was allowed to react for one hour at 25 °C and was then analyzed by ^{1}H and ^{19}F DOSY NMR. The diffusion coefficients, hydrodynamic volumes, and all additional DOSY-derived parameters for this sample are reported in tables S21 and S22. For the measurement a set of 4 dummy scans was used for every sample.

Table S21. Experimental self-diffusion coefficients D_i, viscosity corrected hydrodynamic radii r_H and resulting volumes V_A of TEG with errors calculated from the standard deviation of the diffusion coefficients (conditions: DOSY sample 1, vide supra). Acetonitrile was used as viscosity reference for the experimental self-diffusion coefficients D_i to allow for a qualitative comparison of hydrodynamic radii r_H and resulting volumes V_A. ^{1}H-DOSY with SW = 10 ppm, O1P = 4.75 ppm, gradient strength 10-95% linear (SMSQ10.100 pulse shape). ns: 8; d1 = 1 s; d20 = 0.04; p30 = 1750 us.

Solvent	Species (ppm)	Diffusion coefficient D_i ($m^2.s^{-1}$)	Standard deviation D_i ($m^2.s^{-1}$)	Hydro-dynamic radius r_H (Å)	Volume V_A (Å^3)	Error Volume V_A (Å^3)	R^2
Acetonitrile	3.35	1.01×10^{-9}	4.38×10^{-12}	6.831	1335	± 16	0.99

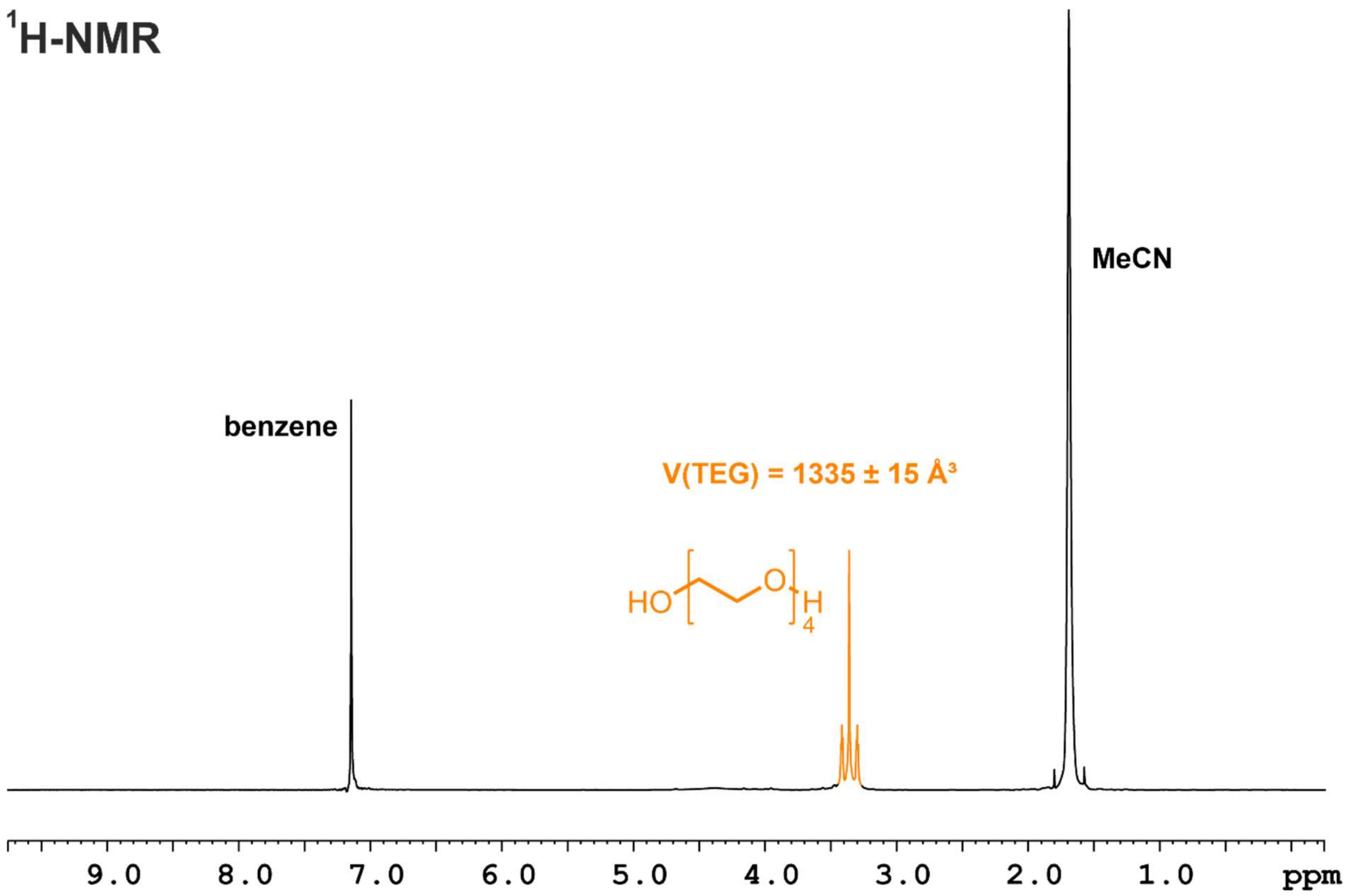

Figure S25. ^{1}H-NMR (600.4 MHz, MeCN-d_3, C_6H_6, TEG) spectrum with signals used for DOSY evaluation of TEG in the presence of benzene and MeCN as solvent at 298K. A qualitative volume of TEG of 1335 ± 15 Å^3 could be determined.

With DOSY, the hydrodynamic volume of predominantly (or completely) free TEG under the applied sample conditions (10 vol% TEG in MeCN) was determined to be 1335 ± 15 Å^3.

Table S22. Experimental self-diffusion coefficients D_i, viscosity corrected hydrodynamic radii r_H and resulting volumes V_A of the K[PF_5·TEG] adduct (conditions: DOSY sample 1, vide supra) with errors calculated from the standard deviation of the diffusion coefficients. Acetonitrile was used as viscosity reference for the experimental self-diffusion coefficients D_i to allow for a qualitative comparison of hydrodynamic radii r_H and resulting volumes V_A. ^{19}F-DOSY with SW = 10 ppm, O1P = -68.0 ppm, gradient strength 10-95% linear (SMSQ10.100 pulse shape). ns: 64; d1 = 1 s; d20 = 0.035; p30 = 1750 us.

Solvent	Proposed species (ppm)	Diffusion coefficient D_i ($m^2.s^{-1}$)	Standard deviation D_i ($m^2.s^{-1}$)	Hydro-dynamic radius r_H (Å)	Volume V_A (Å^3)	Error Volume V_A (Å^3)	R^2
Acetonitrile	-69.7<br>-71.0	$9.52x10^{-10}$	$6.61x10^{-12}$	7.178	1550	± 44	0.99

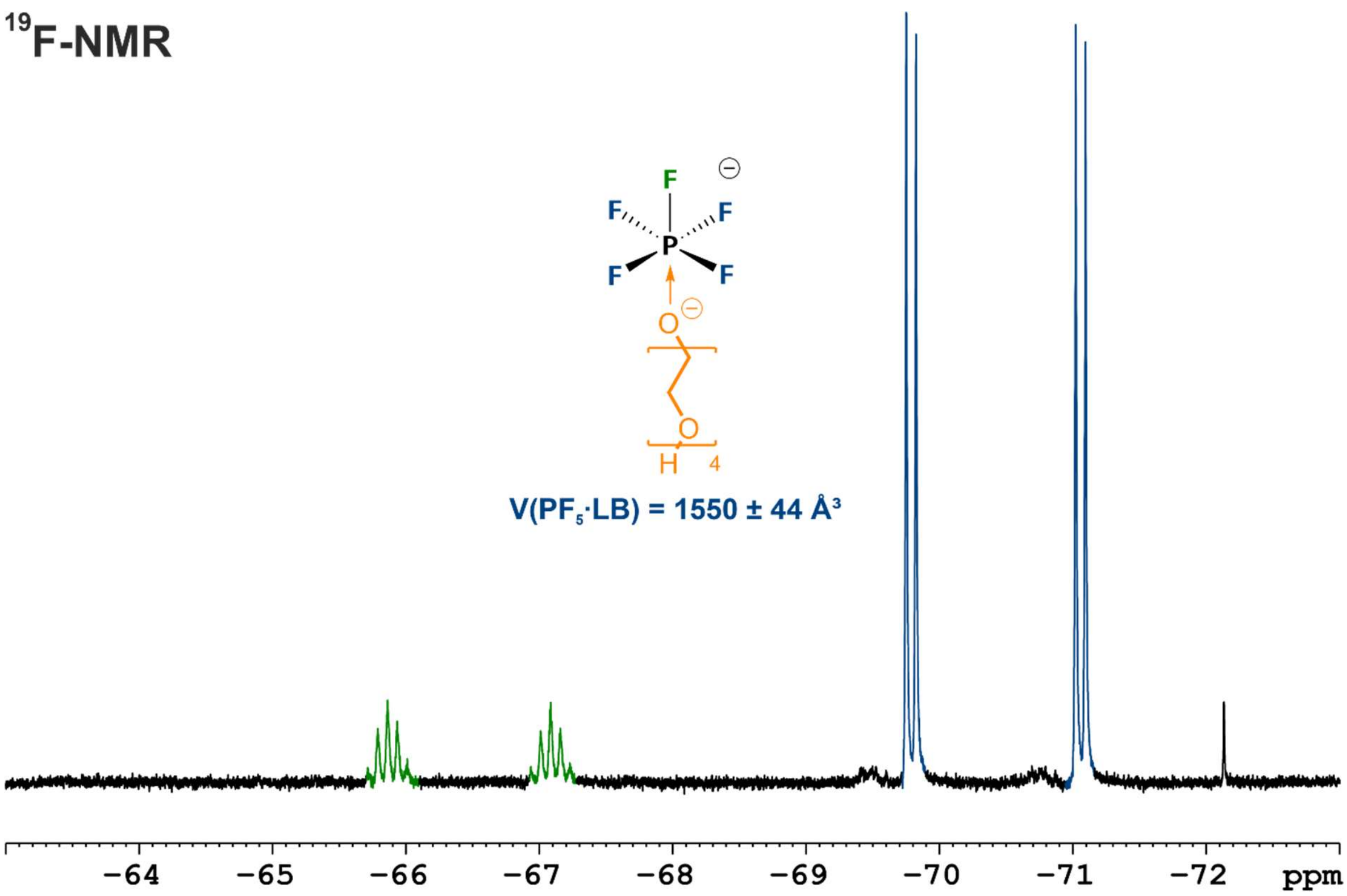

Figure S26. ^{19}F-NMR (564.5 MHz, MeCN-d_3, C_6H_6, TEG) spectrum with signals used for DOSY evaluation of the proposed PF_5·TEG Lewis base adduct in the presence of benzene and MeCN as solvent at 298K (conditions: DOSY sample 1, vide supra). A qualitative volume of TEG of 1550 Å^3 could be determined. These corresponding two blue signals were used for DOSY evaluation.

When examining the same sample, the hydrodynamic volume of the PF_5·LB was found to be 1550 ± 44 Å^3, which lies within the same range as that of free TEG but is approximately 200 Å^3 larger. This increase is consistent with the formation of a K[PF_5·TEG] complex.

DOSY Sample 2 was prepared by dissolving KPF_6 (5.0 mg, 0.027 mmol) in a 1.0 mL mixture of 8:1:1 MeCN-d_6:benzene:TEG (v:v); the sample was then analyzed by DOSY NMR. The diffusion coefficients, hydrodynamic volumes, and all additional DOSY-derived parameters for this sample are reported in Table S23.

Table S23. Experimental self-diffusion coefficients D_i, viscosity corrected hydrodynamic radii r_H and resulting volumes V_A for PF_6^- as a reference with errors calculated from the standard deviation of the diffusion coefficients. Acetonitrile was used as viscosity reference for the experimental self-diffusion coefficients D_i to allow for a qualitative comparison of hydrodynamic radii r_H and resulting volumes V_A. ^{19}F-DOSY with SW = 10 ppm, O1P = -78.1 ppm, gradient strength 10-95% linear (SMSQ10.100 pulse shape). ns: 32; d1 = 1 s; d20 = 0.035; p30 = 1500 us.

Solvent	Species (ppm)	Diffusion coefficient D_i ($m^2.s^{-1}$)	Standard deviation D_i ($m^2.s^{-1}$)	Hydro-dynamic radius r_H (Å)	Volume V_A (Å^3)	Error Volume V_A (Å^3)	R^2
Acetonitrile	-77.5<br>-78.7	2.02x10^{-9}	6.23x10^{-12}	3.843	237.9	± 10.0	0.99

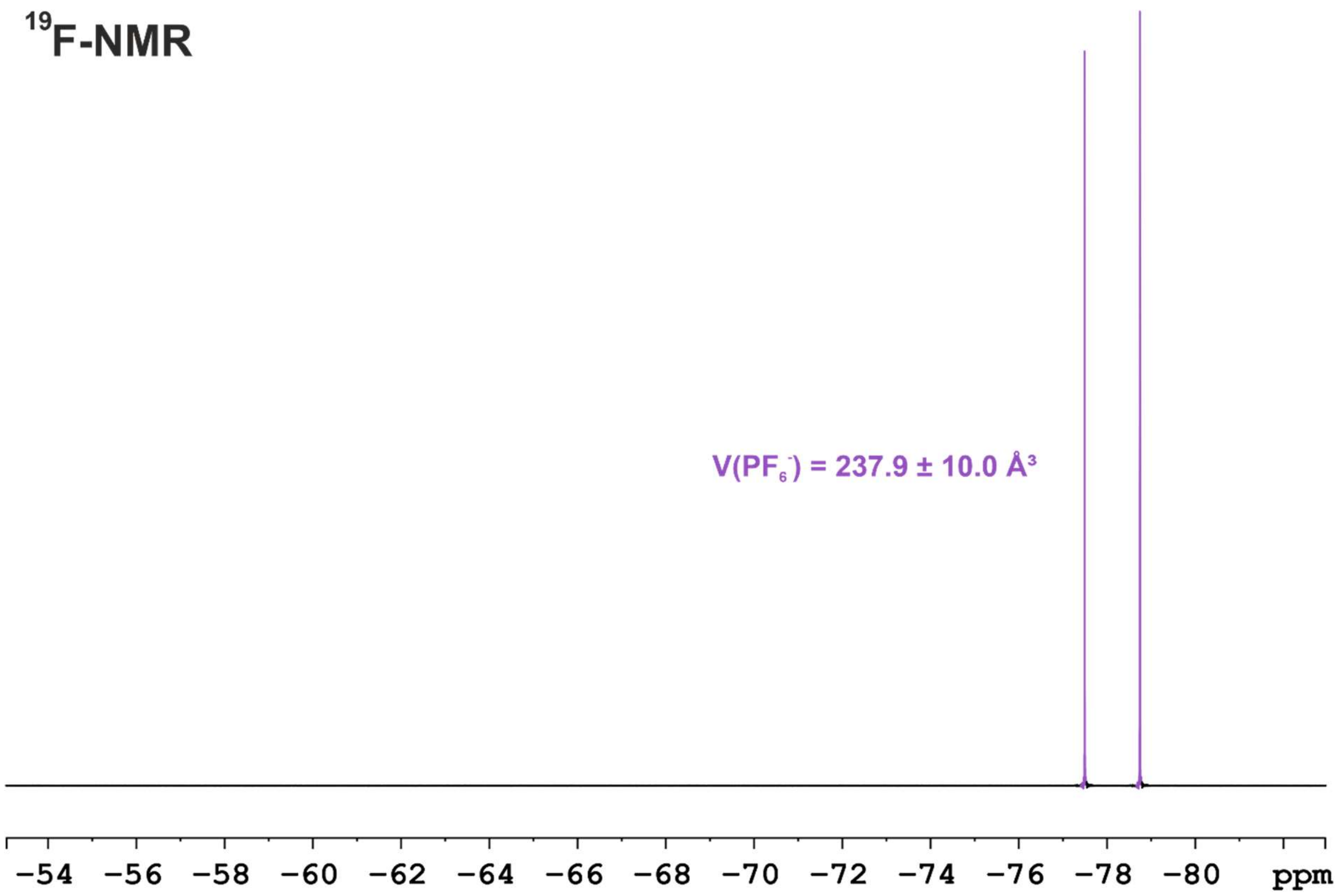

Figure S27. ^{19}F-NMR (564.5 MHz, MeCN-d_3, C_6H_6, TEG) spectrum with signals used for DOSY evaluation of PF_6^- with MeCN as solvent at 298K. A qualitative volume of PF_6^- of 238 ± 10 Å^3 could be determined. Thes corresponding two violet signals were used for DOSY evaluation.

The sum of the hydrodynamic volumes of KPF_6 and TEG closely matches the volume measured for K[PF_5·TEG], providing additional evidence for the presence of this adduct in solution.

4. NMR Spectra

4.1. Screening of Fluoride Sources

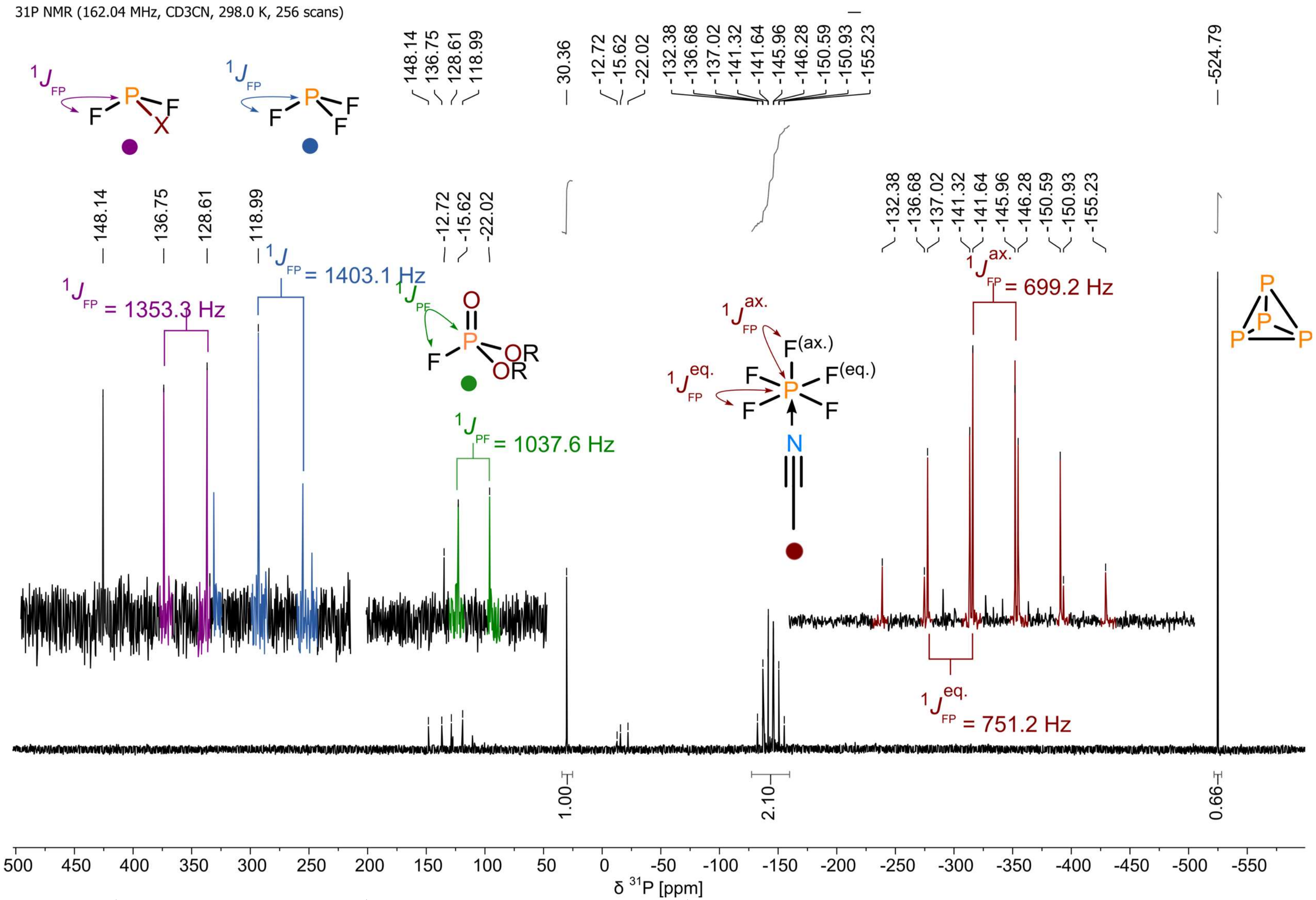

Figure S28. $^{31}P\{^1H\}$ NMR (162.04 MHz, MeCN-d_3, C_6D_6) corresponding to Table S5, entry 13 (conditions: 0.25 equiv. P_4, 5.0 equiv. DDQ, 7.5 equiv. LiF, 3.75 equiv. crypt[2.2.2] , MeCN-d_3:C_6D_6 (9:1, 0.01 M) 18 h, 65 °C, N_2). Signal assignments are based on chemical shifts, coupling constants, and coupling patterns, either by comparison with reported literature data or, where no direct precedent exists, by analogy to closely related species.[2,22]

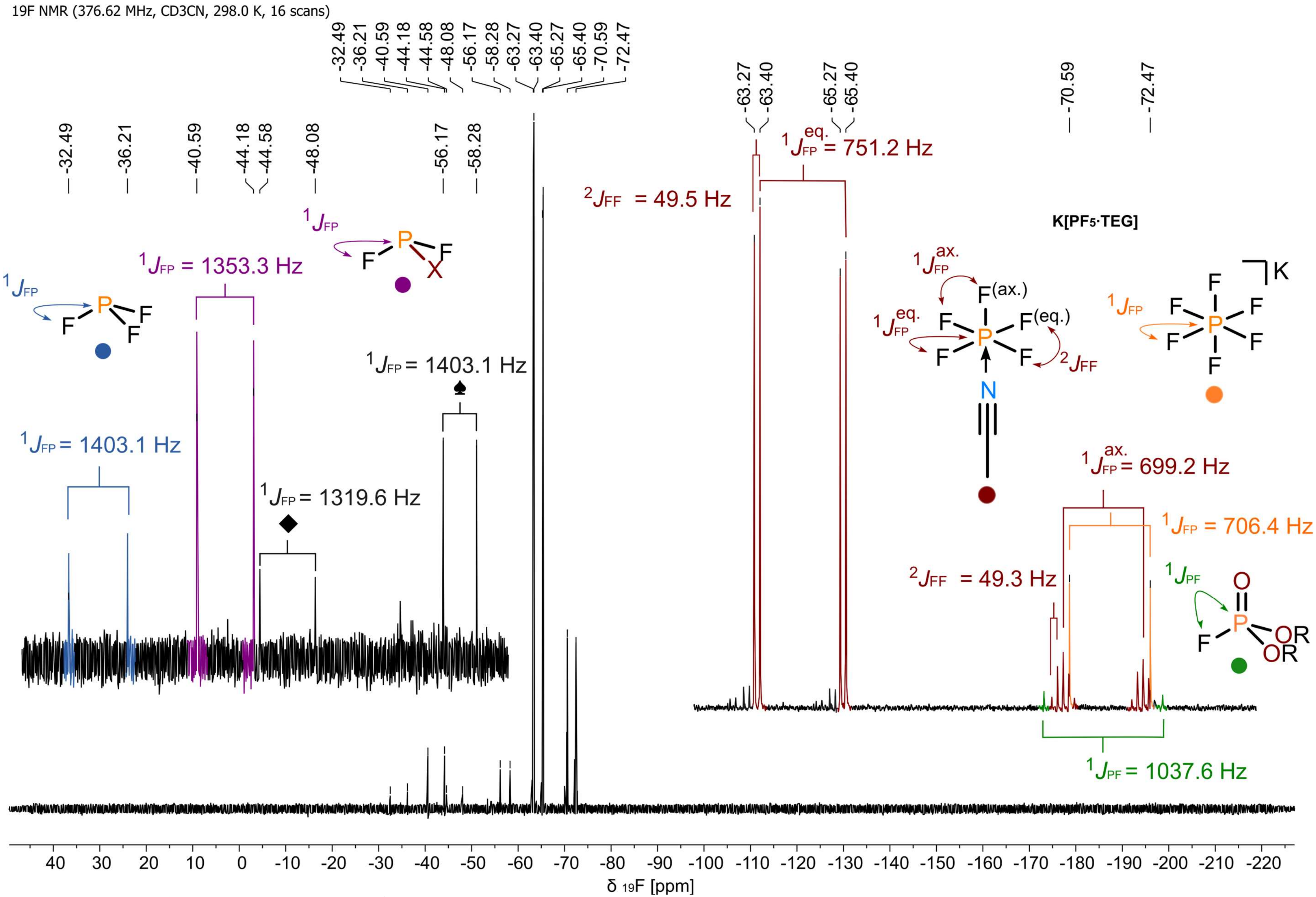

Figure S29. $^{19}F\{^1H\}$ NMR (376.46 MHz, MeCN-d_3, C_6D_6) corresponding to Table S5, entry 13 (conditions: 0.25 equiv. P_4, 5.0 equiv. DDQ, 7.5 equiv. LiF, 3.75 equiv. crypt[2.2.2], MeCN-d_3:C_6D_6 (9:1, 0.01 M) 18 h, 65 °C, N_2). Signal assignments are based on chemical shifts, coupling constants, and coupling patterns, either by comparison with reported literature data or, where no direct precedent exists, by analogy to closely related species. Signals marked (♠,♦) correspond to unidentified species.[2,22]

4.2. Screening of Oxidizing Agents

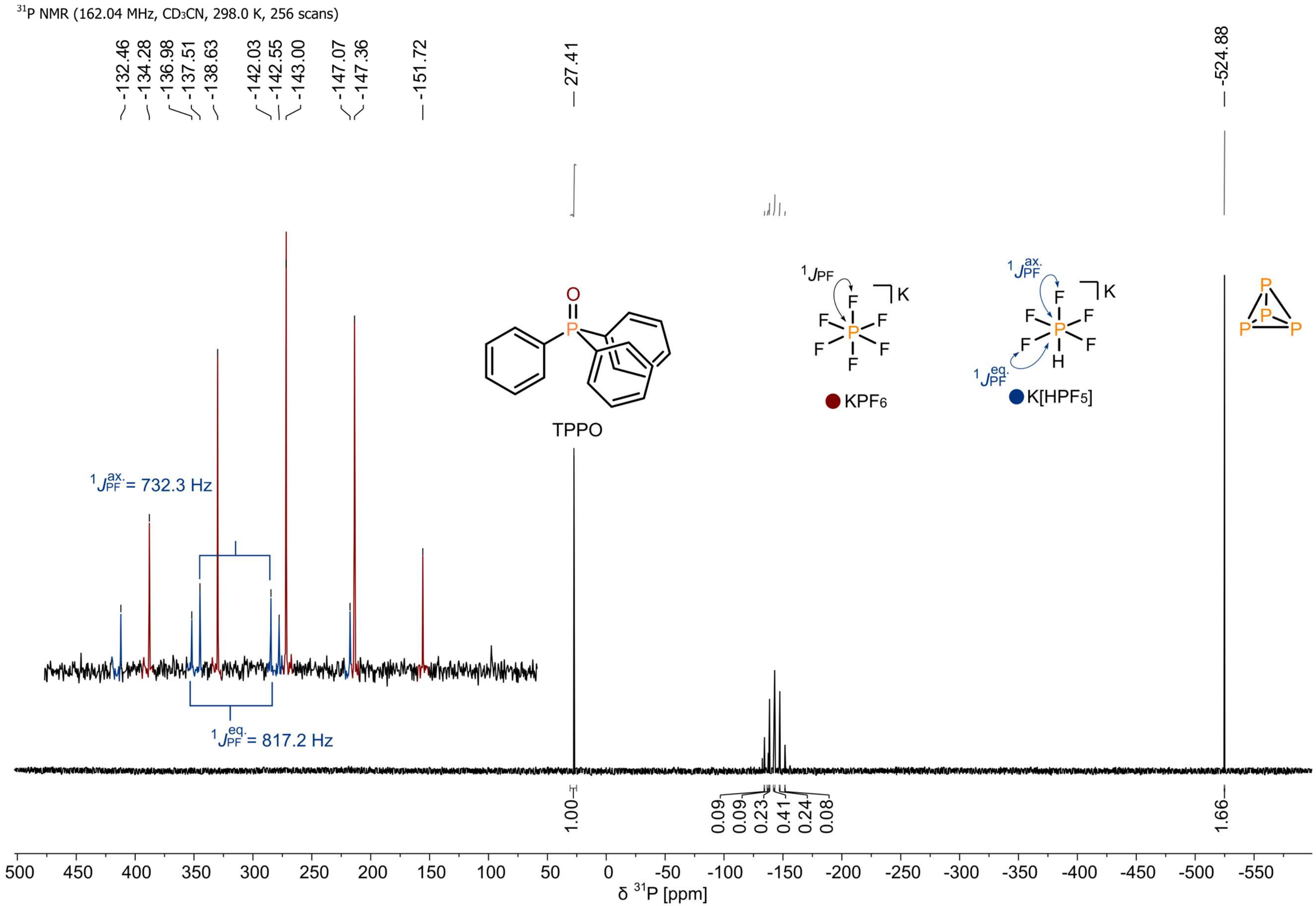

Figure S30. $^{31}P\{^1H\}$ NMR (162.04 MHz, MeCN-d_3, C_6D_6) corresponding to Table S11, entry 1 (conditions: 0.25 equiv. P_4, 5.0 equiv. magic blue, 7.5 equiv. KF, 7.5 equiv. crypt[2.2.2], MeCN-d_3:C_6D_6 (9:1, 0.01 M) 18 h, 65 °C, N_2). Signal assignments are based on chemical shifts, coupling constants, and coupling patterns, either by comparison with reported literature data or, where no direct precedent exists, by analogy to closely related species.[2,10]

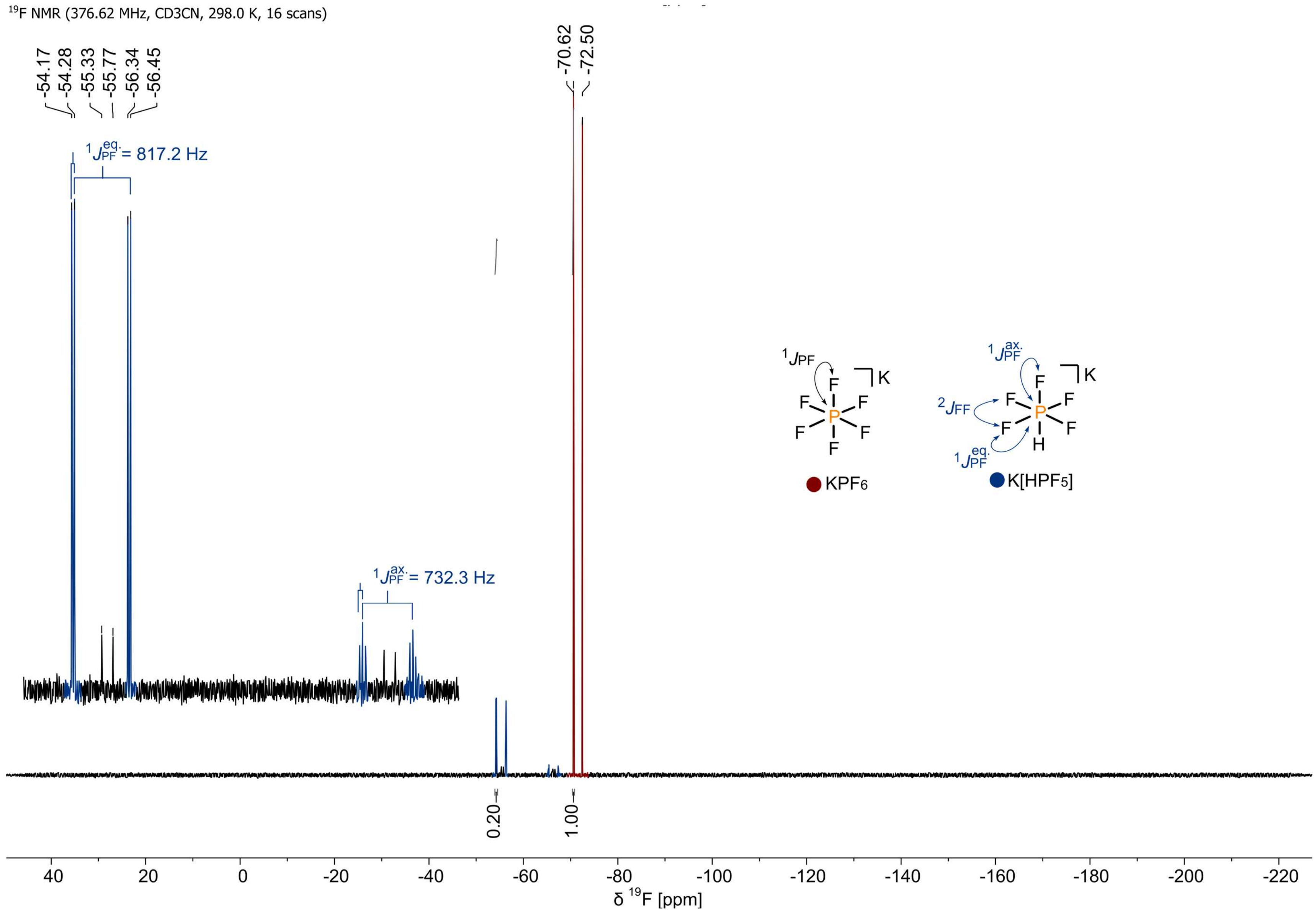

Figure S31. $^{19}F\{^1H\}$ NMR (376.46 MHz, MeCN-d_3, C_6D_6) corresponding to Table S5, entry 13 (conditions: 0.25 equiv. P_4, 5.0 equiv. DDQ, 7.5 equiv. KF, 3.75 equiv. crypt[2.2.2], MeCN-d_3:C_6D_6 (9:1, 0.01 M) 18 h, 65 °C, N_2). Signal assignments are based on chemical shifts, coupling constants, and coupling patterns, either by comparison with reported literature data or, where no direct precedent exists, by analogy to closely related species. [2,10]

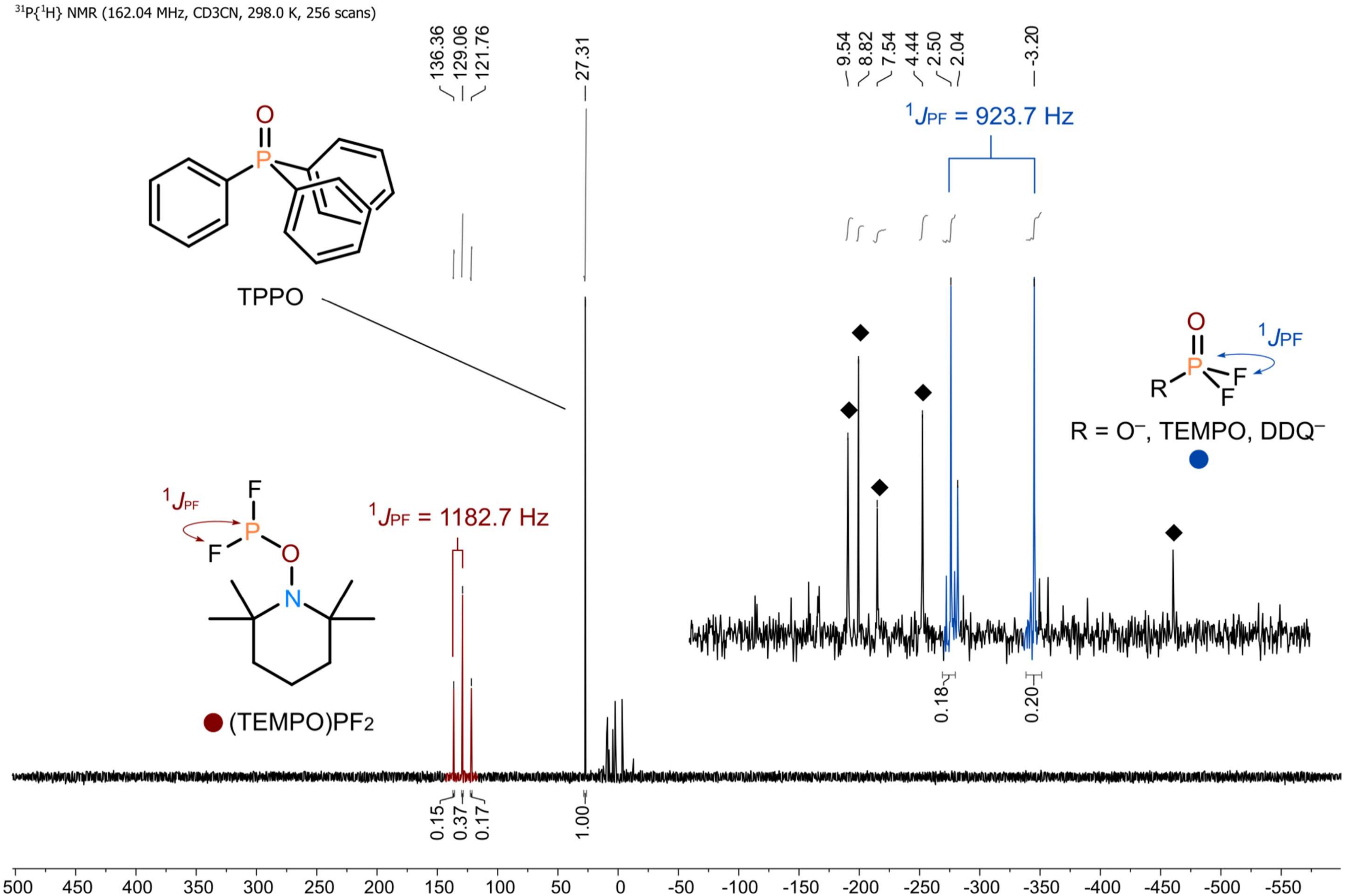

Figure S32. $^{31}P\{^1H\}$ NMR (162.04 MHz, MeCN-d_3, C_6D_6) corresponding to Table S11, entry 2 (conditions: 0.25 equiv. P_4, 5.0 equiv. TEMPO, 7.5 equiv. KF, 7.5 equiv. crypt[2.2.2], MeCN-d_3:C_6D_6 (9:1, 0.01 M) 18 h, 65 °C, N_2). Signal assignments are based on chemical shifts, coupling constants, and coupling patterns, either by comparison with reported literature data or, where no direct precedent exists, by analogy to closely related species. The formation of the proposed (TEMPO)PF_2 species is supported by its characteristic coupling pattern and is observed in 17% yield by $^{31}P\{^1H\}$ NMR integration (see Section S1.3). Signals marked (◆) correspond to unidentified species.[2]

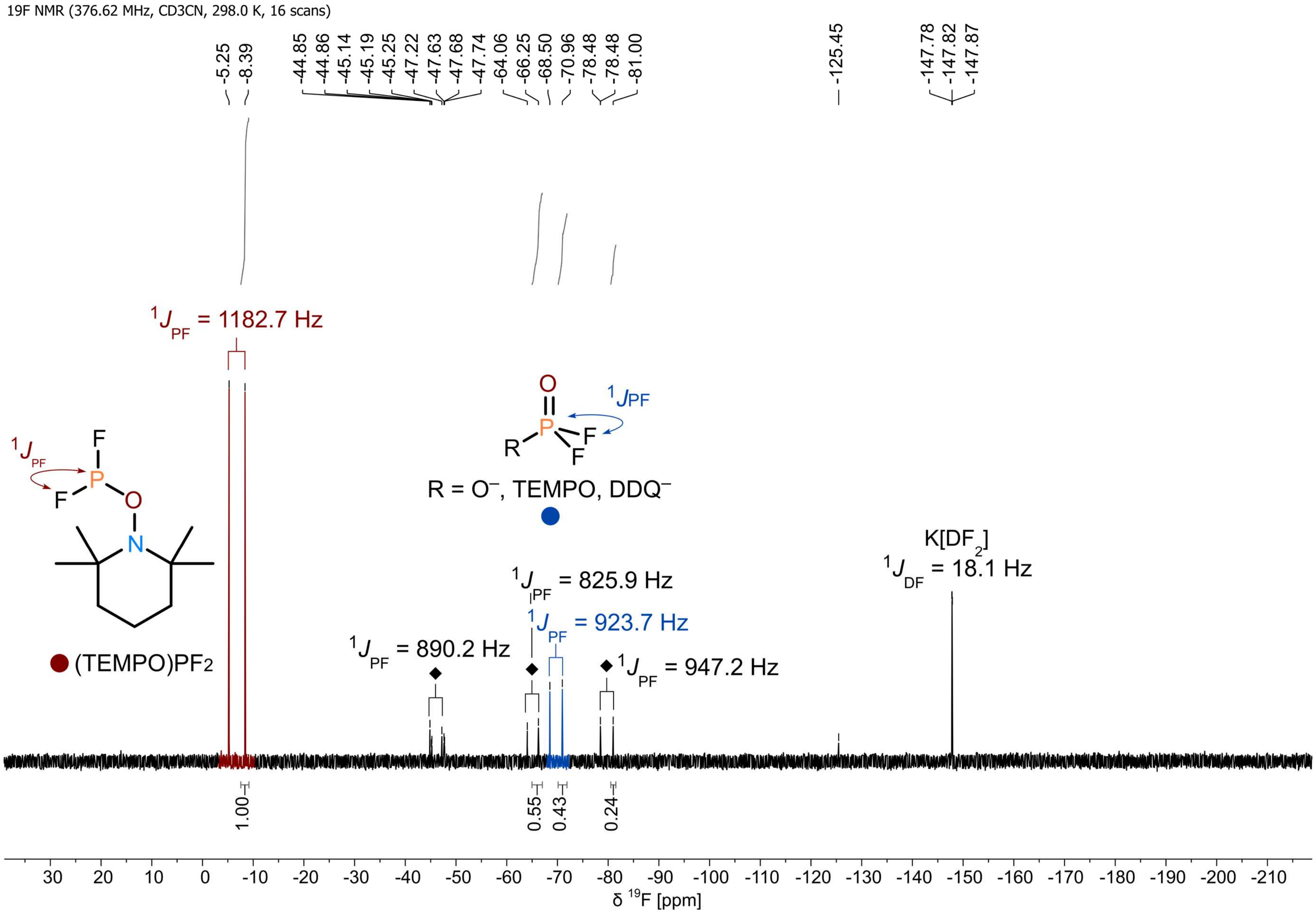

Figure S33. $^{19}F\{^1H\}$ NMR (376.46 MHz, MeCN-d_3, C_6D_6) corresponding to Table S11, entry 2 (conditions: 0.25 equiv. P_4, 5.0 equiv. TEMPO, 7.5 equiv. KF, 7 .5 equiv. crypt[2.2.2], MeCN-d_3:C_6D_6 (9:1, 0.01 M) 18 h, 65 °C, N_2). Signal assignments are based on chemical shifts, coupling constants, and coupling patterns, either by comparison with reported literature data or, where no direct precedent exists, by analogy to closely related species. The formation of the proposed (TEMPO)PF_2 species is supported by its characteristic coupling pattern and is observed in 17% yield by $^{31}P\{^1H\}$ NMR integration (see Section S1.3). Signals marked (◆) correspond to unidentified species.[2,23]

4.3. Sensitivity to Water and Oxidation

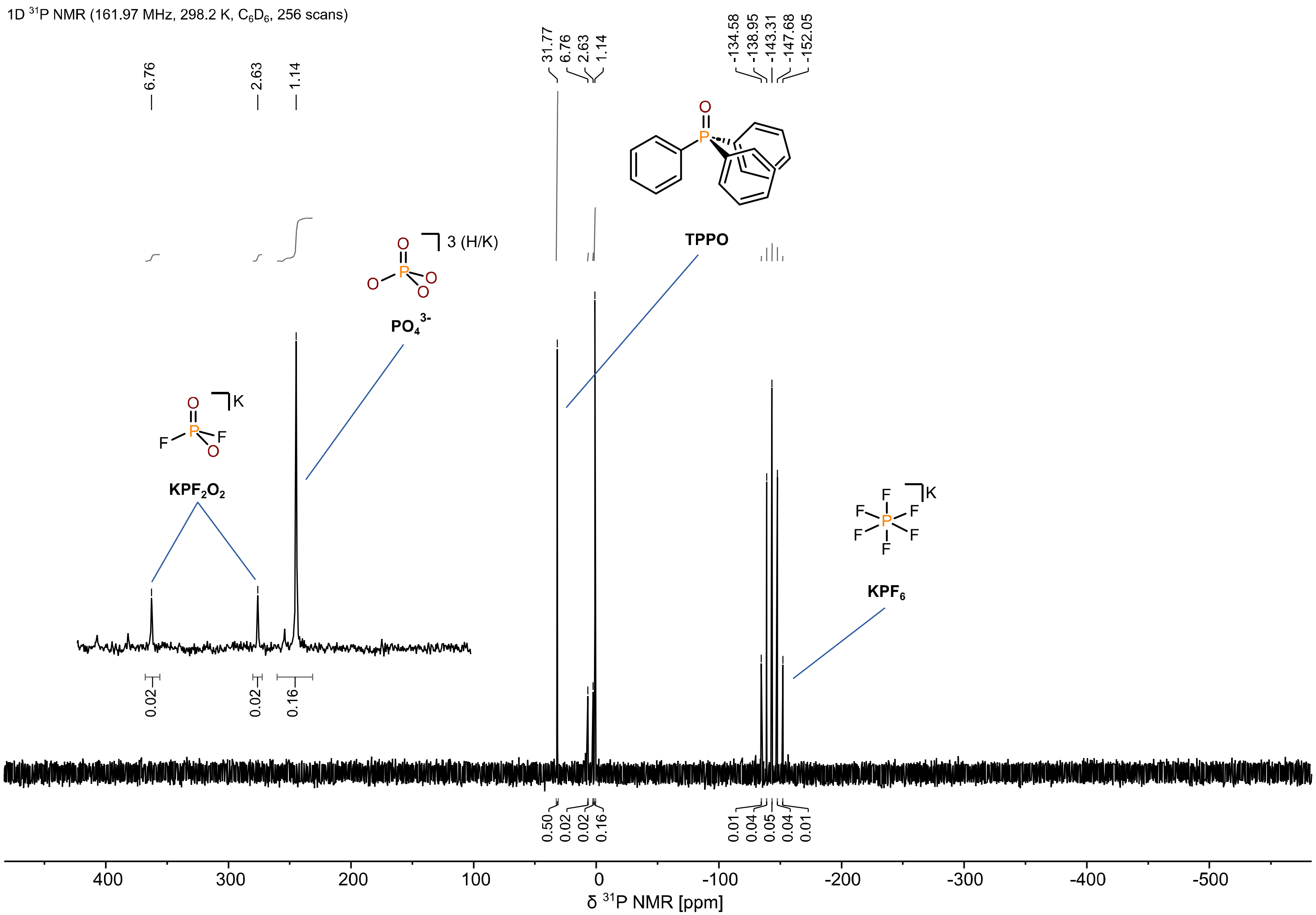

Figure S34. $^{31}P\{^1H\}$ NMR (161.97 MHz, MeCN, C_6D_6) corresponding to Table S12, entry 2 (conditions: 0.25 equiv. P_4, 5.0 equiv. DDQ, 7.5 equiv. KF, MeCN-d_3:C_6D_6 (9:1, 0.01 M), 18 h, 65 °C, air). Signal assignments are based on chemical shifts, coupling constants, and coupling patterns, either by comparison with reported literature data or, where no direct precedent exists, by analogy to closely related species.[2]

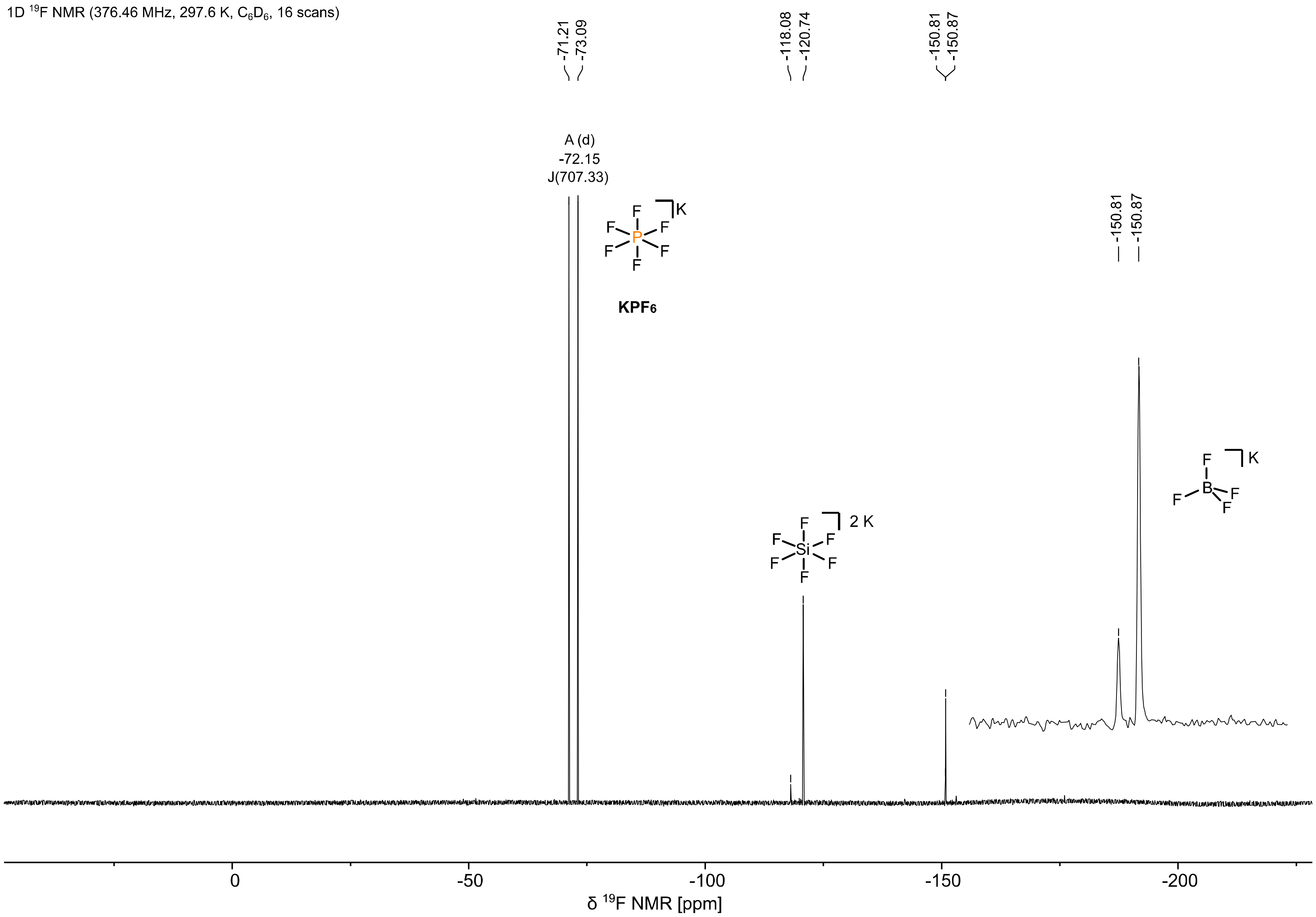

Figure S35. $^{19}F\{^{1}H\}$ NMR (376.46 MHz, MeCN, C_6D_6) corresponding to Table S12, entry 2 (conditions: 0.25 equiv. P_4, 5.0 equiv. DDQ, 7.5 equiv. KF, MeCN-d_3:C_6D_6 (9:1, 0.01 M), 18 h, 65 °C, air). Signal assignments are based on chemical shifts, coupling constants, and coupling patterns, either by comparison with reported literature data or, where no direct precedent exists, by analogy to closely related species.[2,9,24]

4.4. Reaction with Red Phosphorus

1D ^{31}P NMR (161.97 MHz, 298.1 K, C_6D_6, 256 scans)

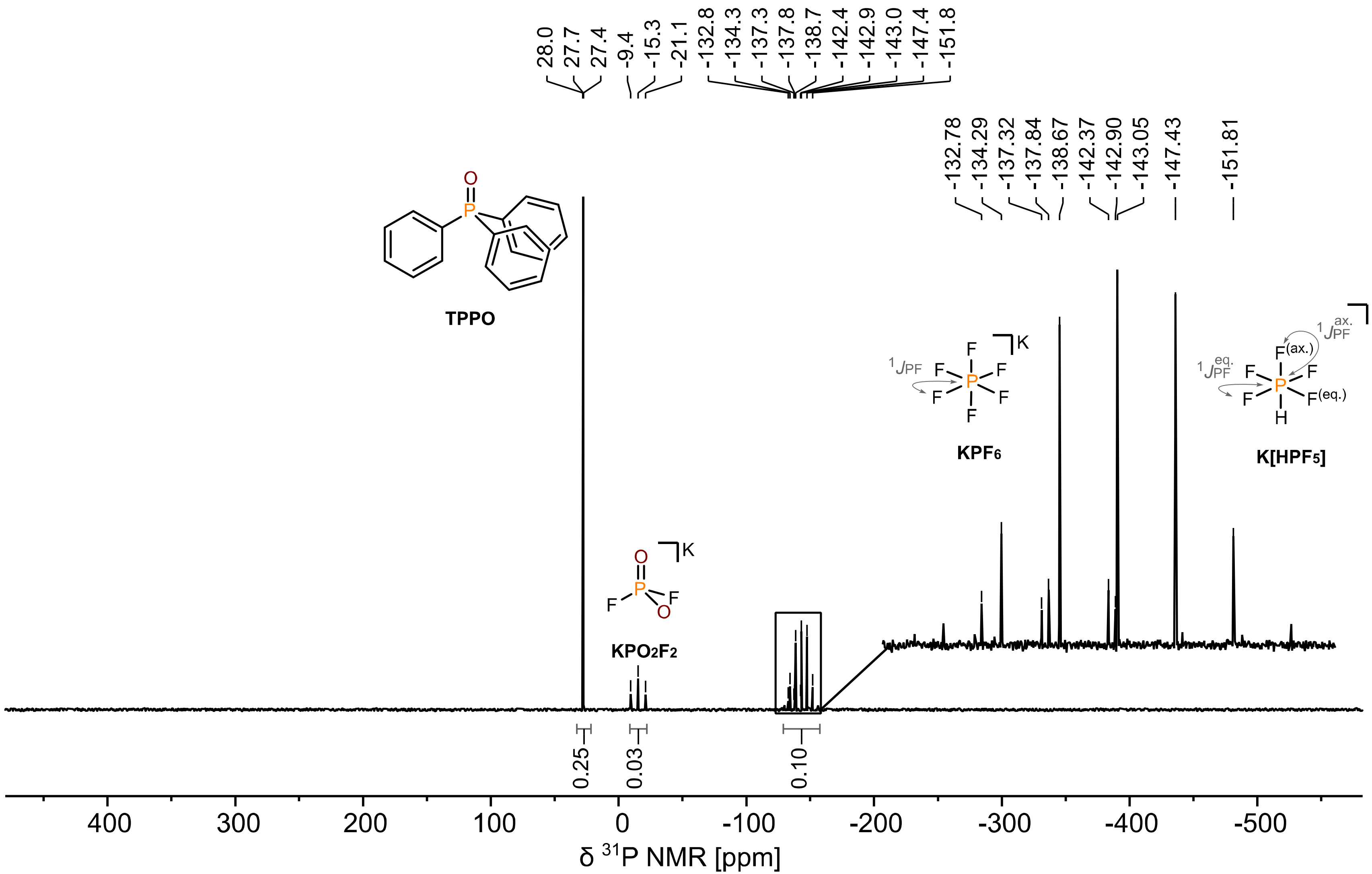

Figure S36. $^{31}P\{^1H\}$ NMR (161.97 MHz, DMF C_6D_6) corresponding to Table S14, entry 1 (conditions: 0.25 equiv. P_{red}, 5.0 equiv. DDQ, 7.5 equiv. KF, DMF (0.01 M), 18 h, 65 °C, N_2). Signal assignments are based on chemical shifts, coupling constants, and coupling patterns, either by comparison with reported literature data or, where no direct precedent exists, by analogy to closely related species.[2,10]

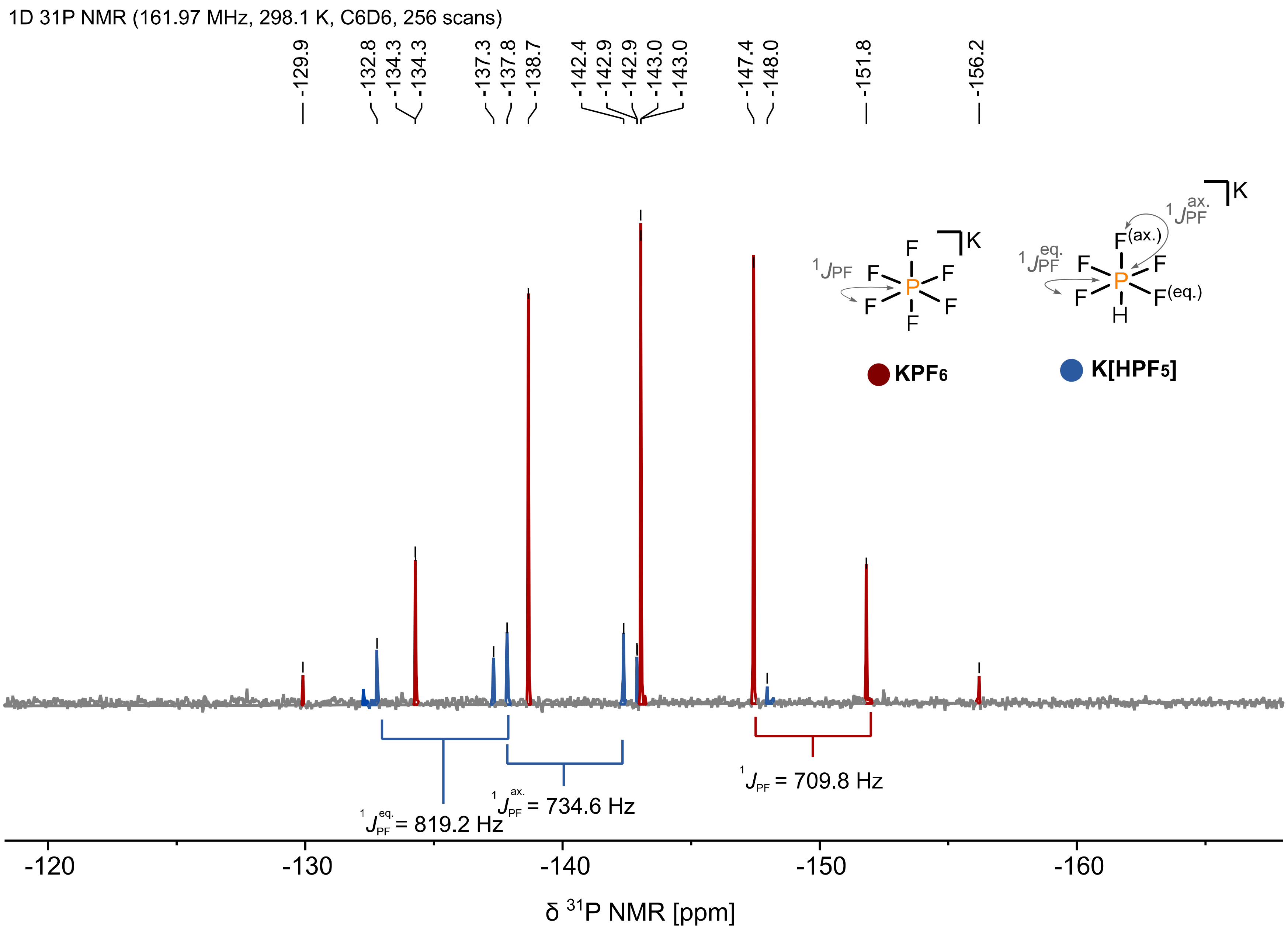

Figure S37. $^{31}P\{^{1}H\}$ NMR (161.97 MHz, DMF, C_6D_6) magnified section of **Figure S36**, showing the ^{31}P resonances and *J* coupling constants associated with KPF_6 (red) and $K[HPF_5]$ (blue).

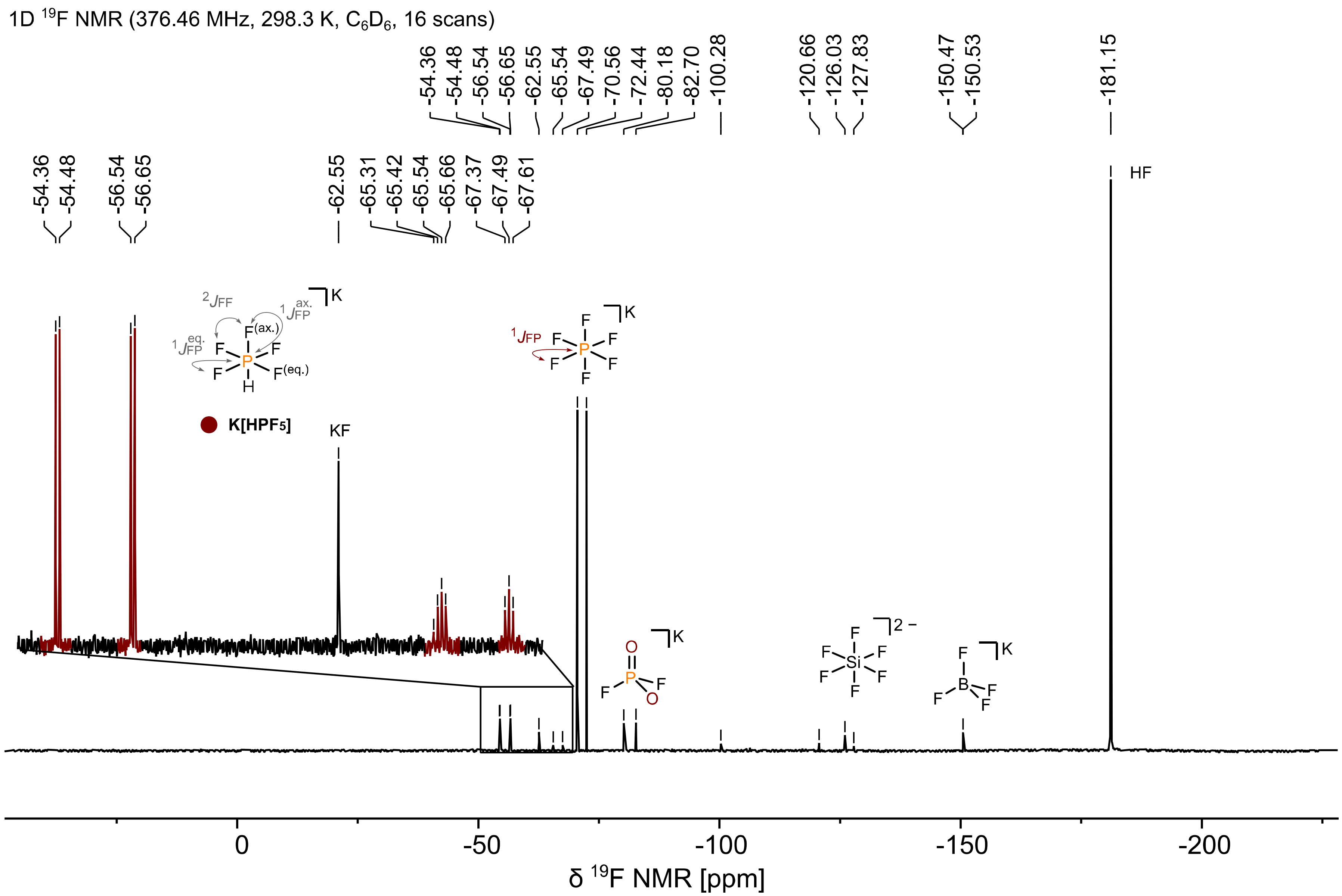

Figure S38. $^{19}F\{^{1}H\}$ NMR (376.46 MHz, DMF, C_6D_6) corresponding to Table S14, entry 1 (conditions: 0.25 equiv. P_{red}, 5.0 equiv. DDQ, 7.5 equiv. KF, DMF (0.01 M), 18 h, 65 °C, N_2). Signal assignments are based on chemical shifts, coupling constants, and coupling patterns, either by comparison with reported literature data or, where no direct precedent exists, by analogy to closely related species.[2,9,10,24,25]

4.5. Reaction Scale-Up

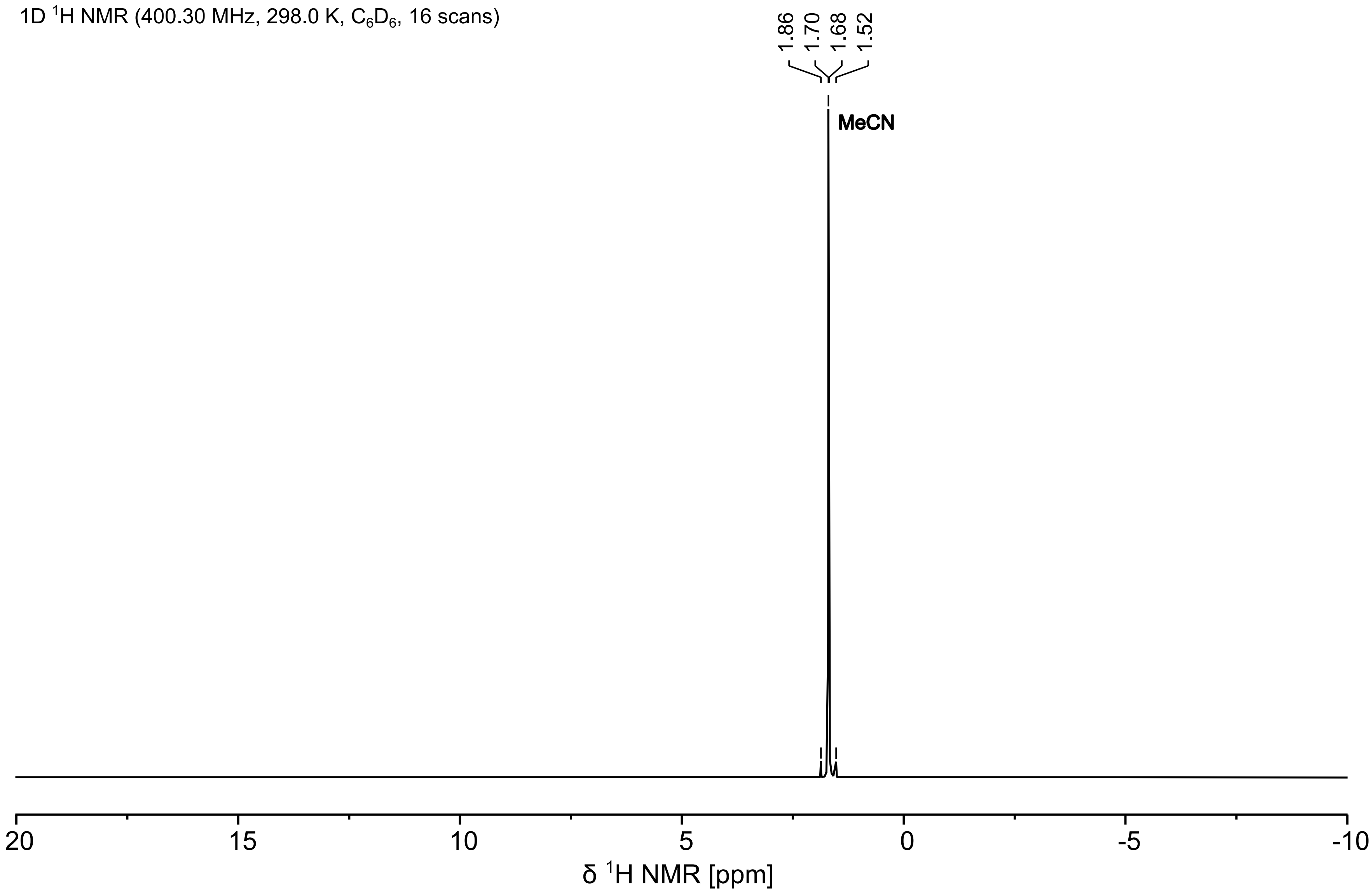

Figure S39. ^{1}H NMR (400.30 MHz, MeCN, C_6D_6) Large scale reaction using 0.1 mmol solid P_4 (see section 3.3; conditions: 0.25 equiv. P_4, 5.0 equiv. DDQ, 7.5 equiv. KF, MeCN (0.01 M), 18 h, 65 °C, N_2).

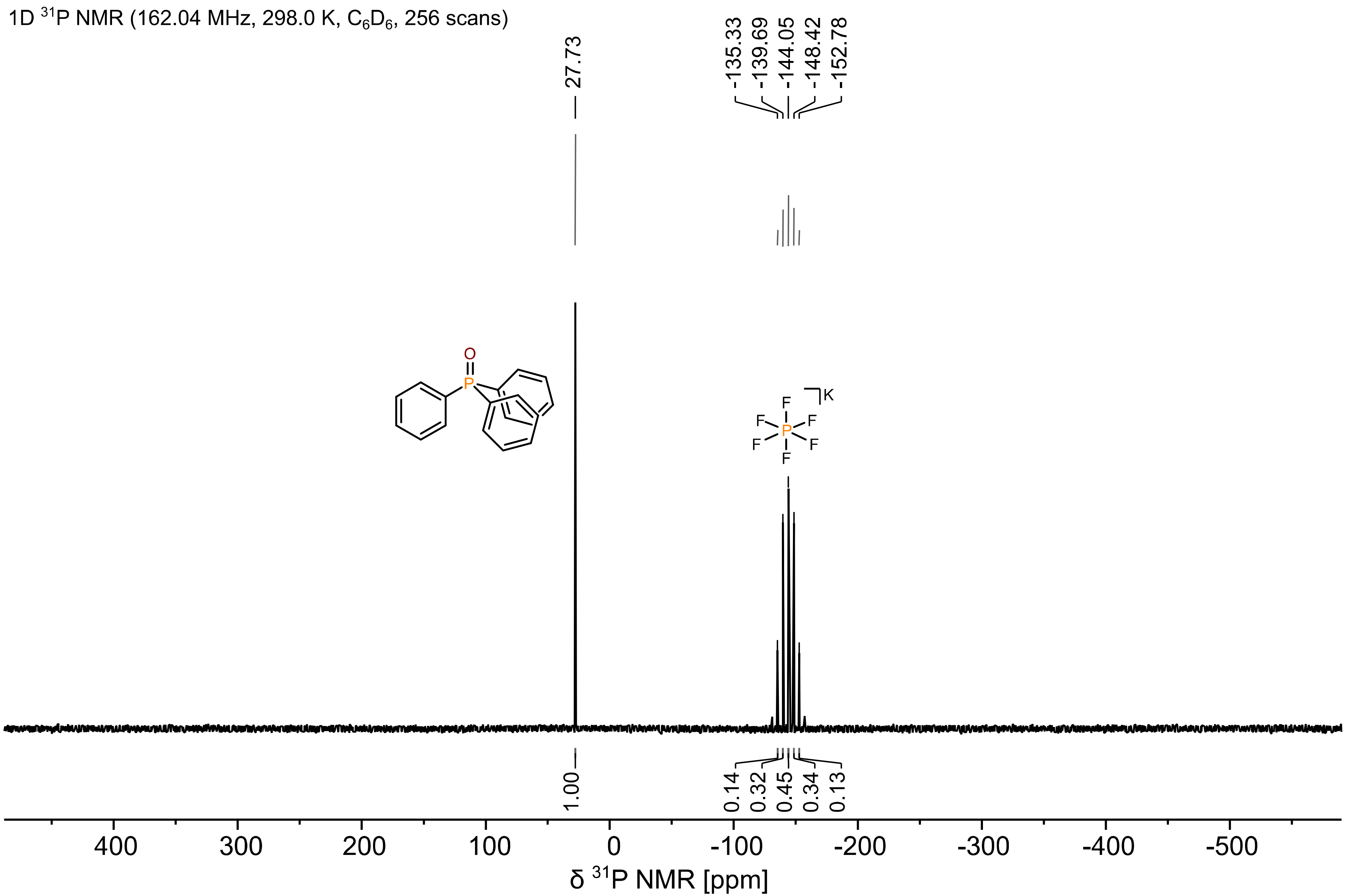

Figure S40. $^{31}P\{^1H\}$ NMR (161.97 MHz, MeCN, C_6D_6) Large scale reaction using 0.1 mmol solid P_4 with internal standard TPPO added, showing the formation of KPF_6 in 83% by NMR integration. (see section 3.3; conditions: 0.25 equiv. P_4, 5.0 equiv. DDQ, 7.5 equiv. KF, MeCN (0.01 M), 18 h, 65 °C, N_2).

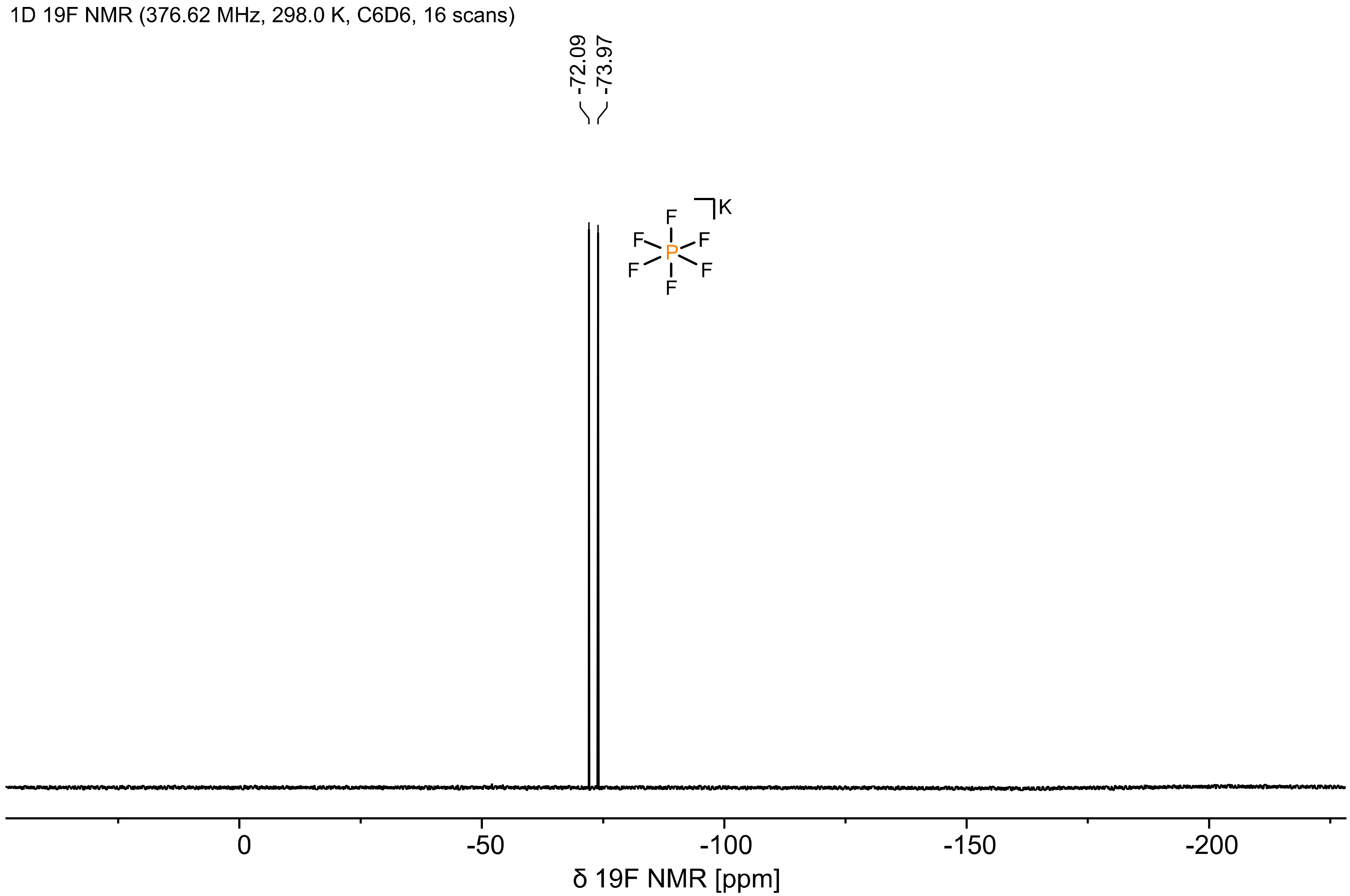

Figure S41. $^{19}F\{^{1}H\}$ NMR (376.46 MHz, MeCN, C_6D_6) Large scale reaction using 0.1 mmol solid P_4 (see section 3.3; conditions: 0.25 equiv. P_4, 5.0 equiv. DDQ, 7.5 equiv. KF, MeCN (0.01 M), 18 h, 65 °C, N_2).

4.6. Isolation of KPF_6

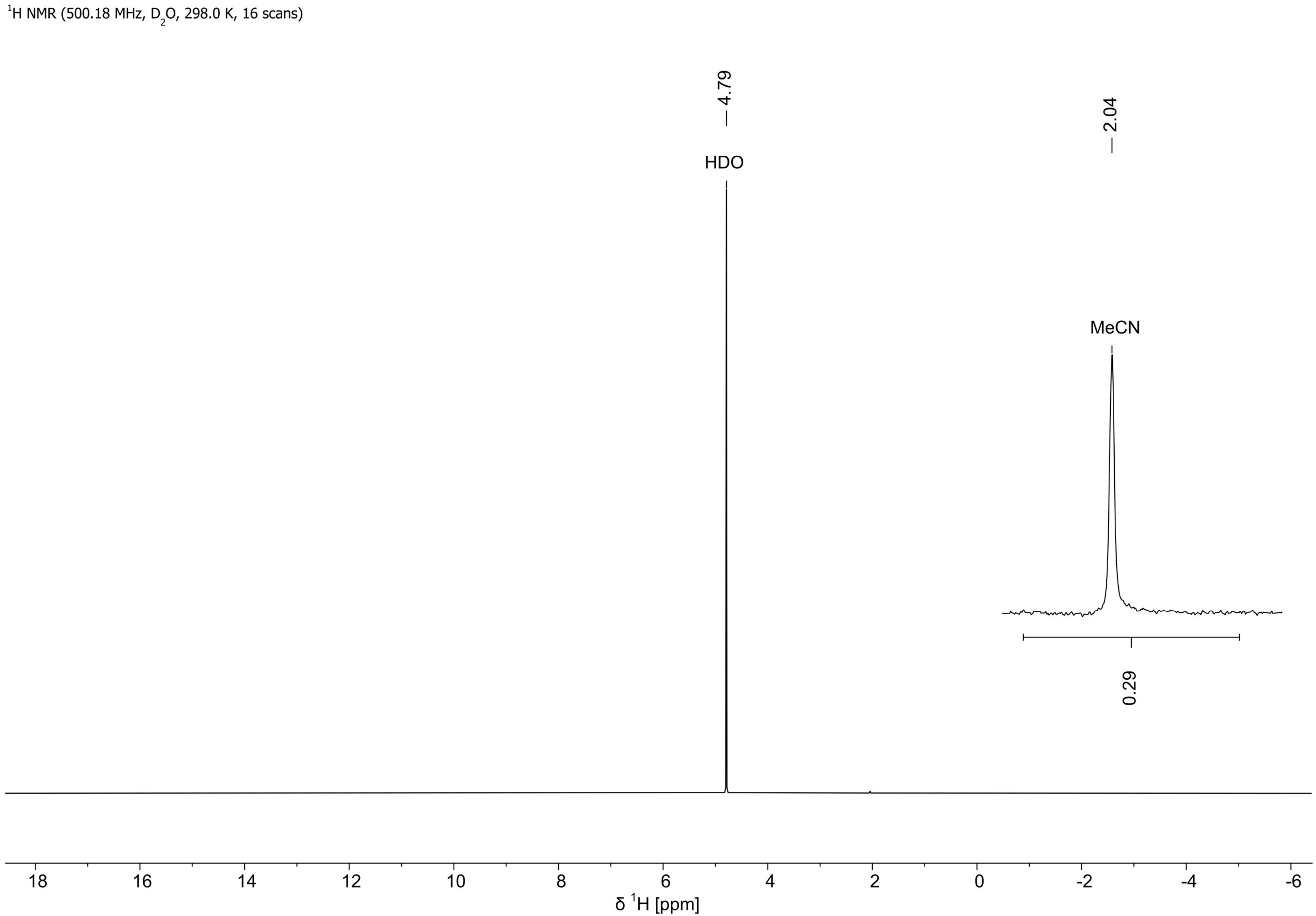

Figure S42. ^{1}H NMR (500.18 MHz, D_2O) of isolated KPF_6 (see sections 3.3 and 3.4; conditions: 0.25 equiv. P_4, 5.0 equiv. DDQ, 7.5 equiv. KF, MeCN (0.01 M), 18 h, 65 °C, N_2).

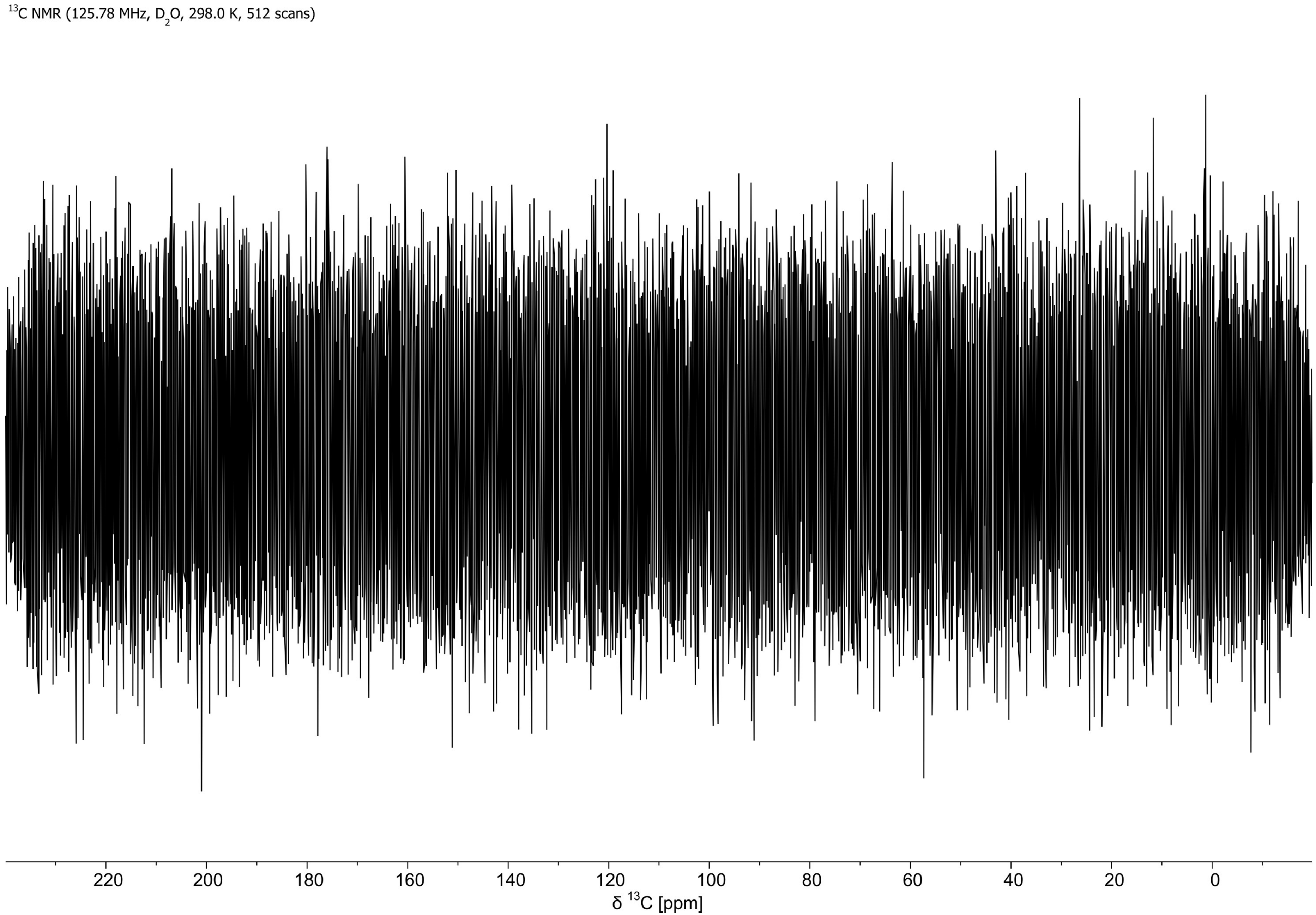

Figure S43. ^{13}C NMR (125.78 MHz, MeCN, C_6D_6) of isolated KPF_6 (see sections 3.3 and 3.4; conditions: 0.25 equiv. P_4, 5.0 equiv. DDQ, 7.5 equiv. KF, MeCN (0.01 M), 18 h, 65 °C, N_2).

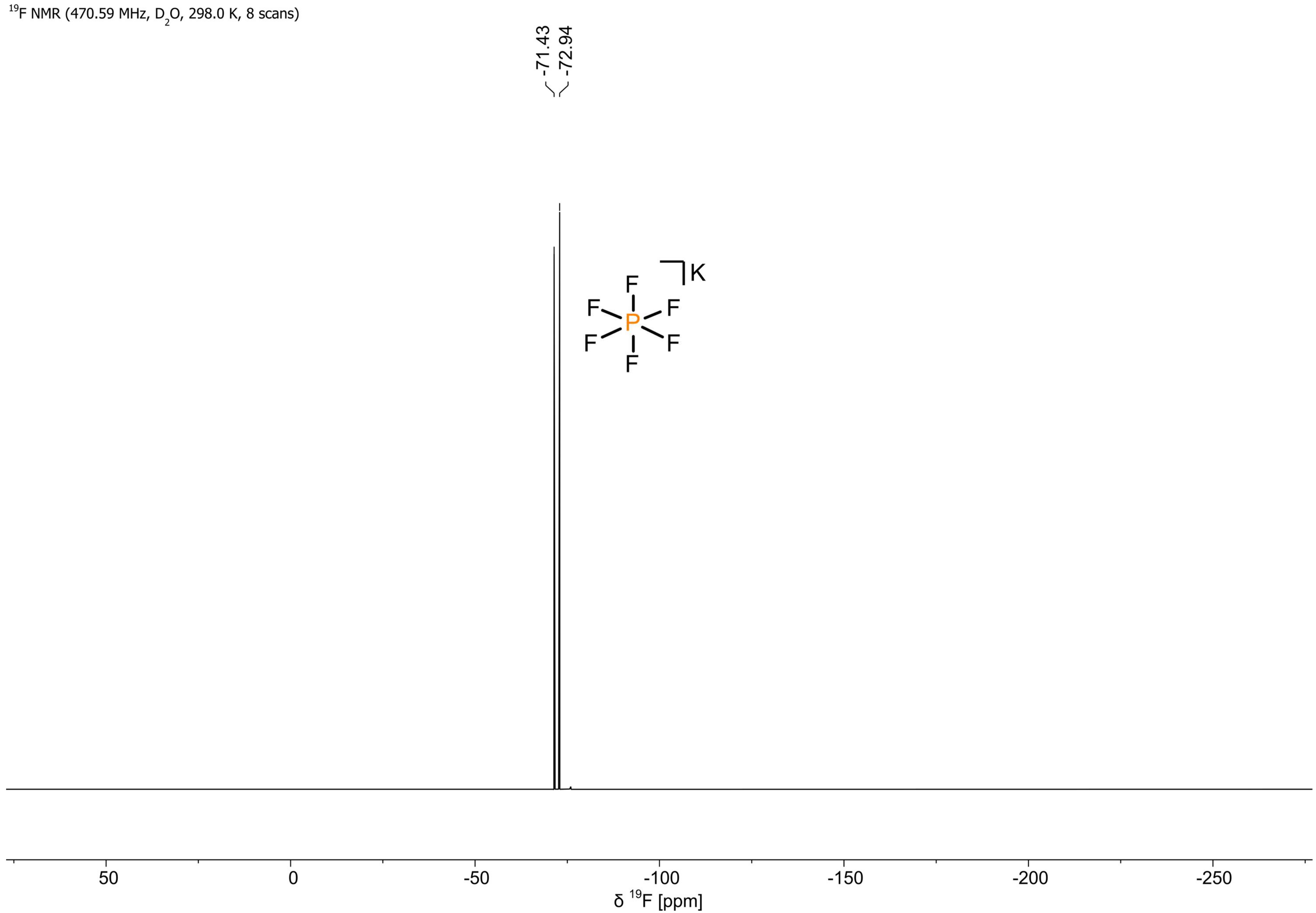

Figure S44. $^{19}F\{^1H\}$ NMR (470.59 MHz, D_2O) of isolated KPF_6 (see sections 3.3 and 3.4; conditions: 0.25 equiv. P_4, 5.0 equiv. DDQ, 7.5 equiv. KF, MeCN (0.01 M), 18 h, 65 °C, N_2).

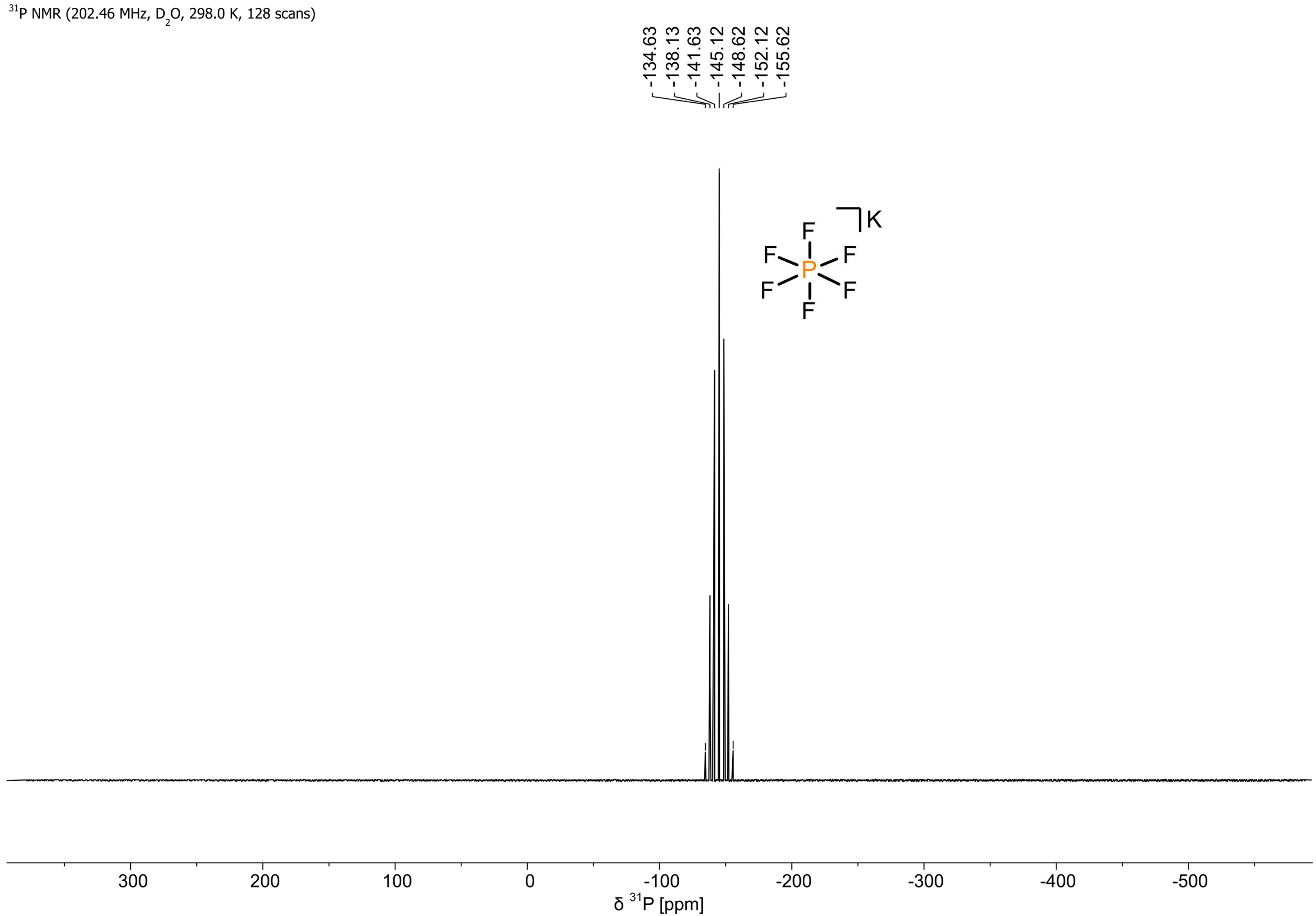

Figure S45. $^{31}P\{^{1}H\}$ NMR (202.46 MHz, D_2O) of isolated KPF_6 (see sections 3.3 and 3.4; conditions: 0.25 equiv. P_4, 5.0 equiv. DDQ, 7.5 equiv. KF, MeCN (0.01 M), 18 h, 65 °C, N_2).

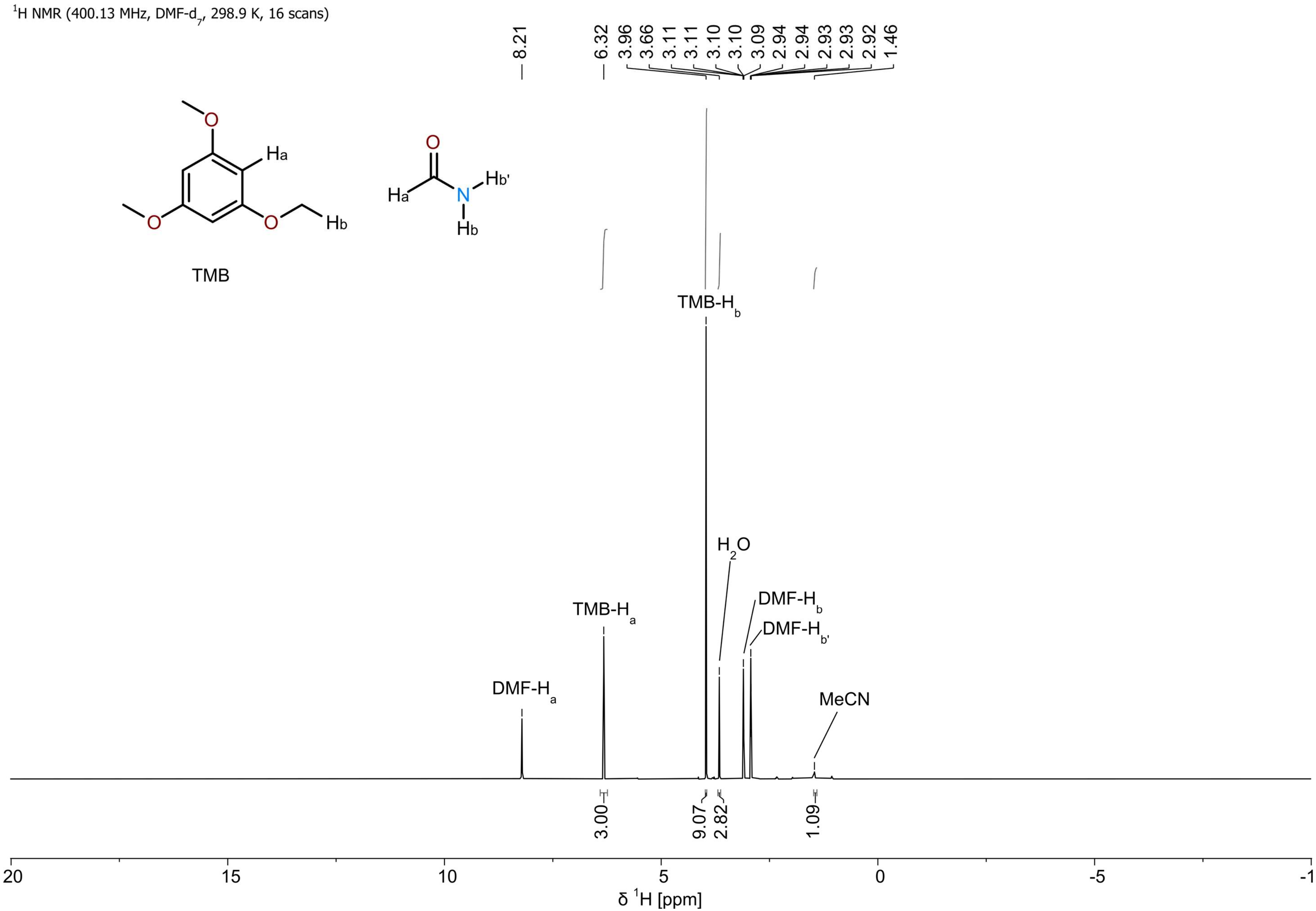

Figure S46. ^{1}H NMR (500.18 MHz, DMF-d_7) of isolated KPF_6 (see sections 3.3 and 3.4; conditions: 0.25 equiv. P_4, 5.0 equiv. DDQ, 7.5 equiv. KF, MeCN (0.01 M), 18 h, 65 °C, N_2) in DMF-d7 with TMB added as internal standard to quantify residual water and MeCN in the sample.

4.7. Effect of TEG as a Co-Solvent

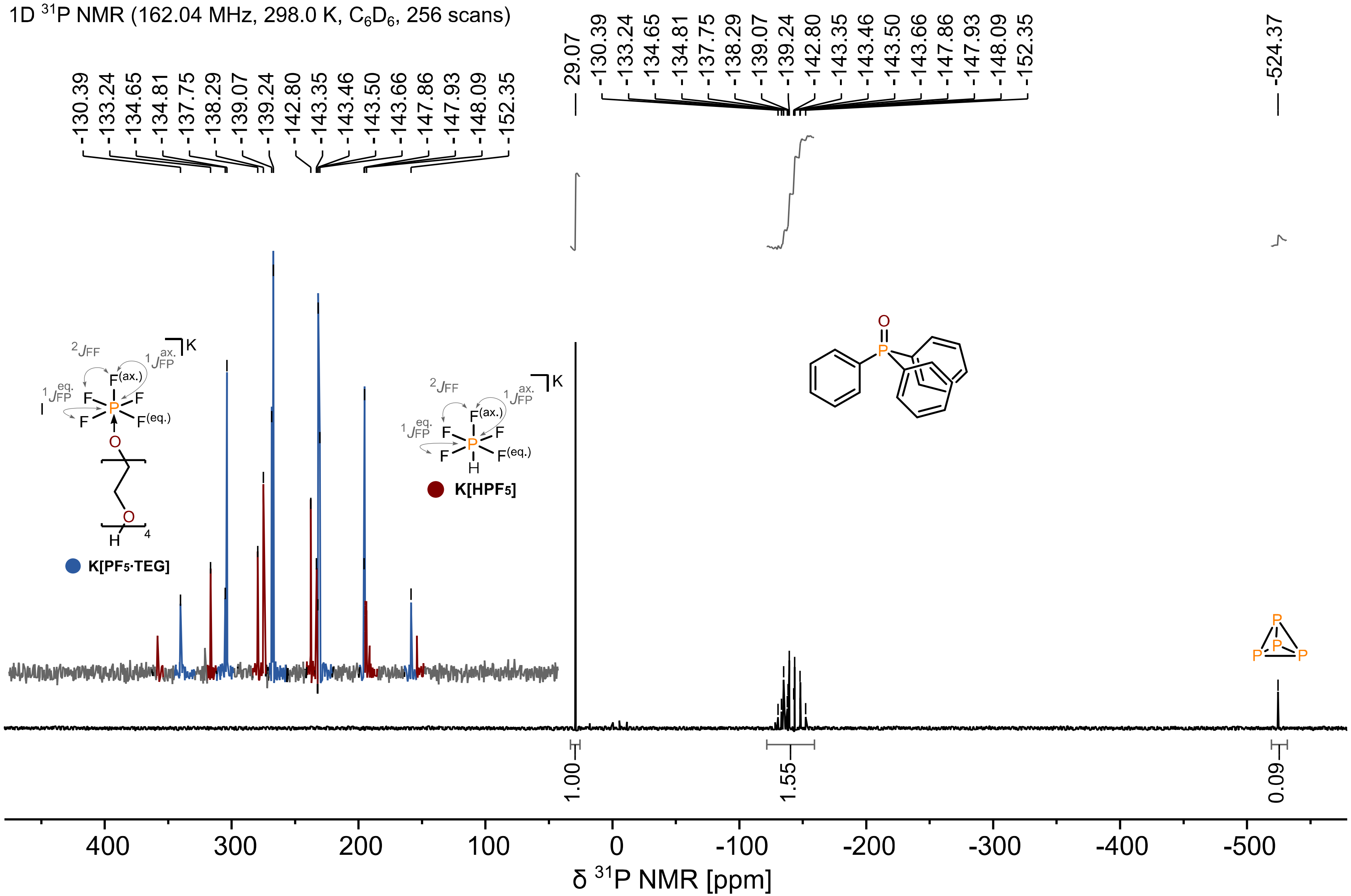

Figure S47. $^{31}P\{^{1}H\}$ NMR (161.97 MHz, MeCN, TEG C_6D_6) corresponding to Table S15, entry 1 (conditions: 0.25 equiv. P_4, 5.0 equiv. DDQ, 7.5 equiv. KF, MeCN:TEG:C_6H_6 (8:1:1, 0.01 M), 18 h, 25 °C, N_2) with internal standard TPPO (δ 29.1 ppm, s) added, showing the formation of K[PF_5·TEG] and K[HPF_5], with almost complete conversion of P_4. For details on chemical shifts and coupling pattern see main manuscript Figure 3.[2,10]

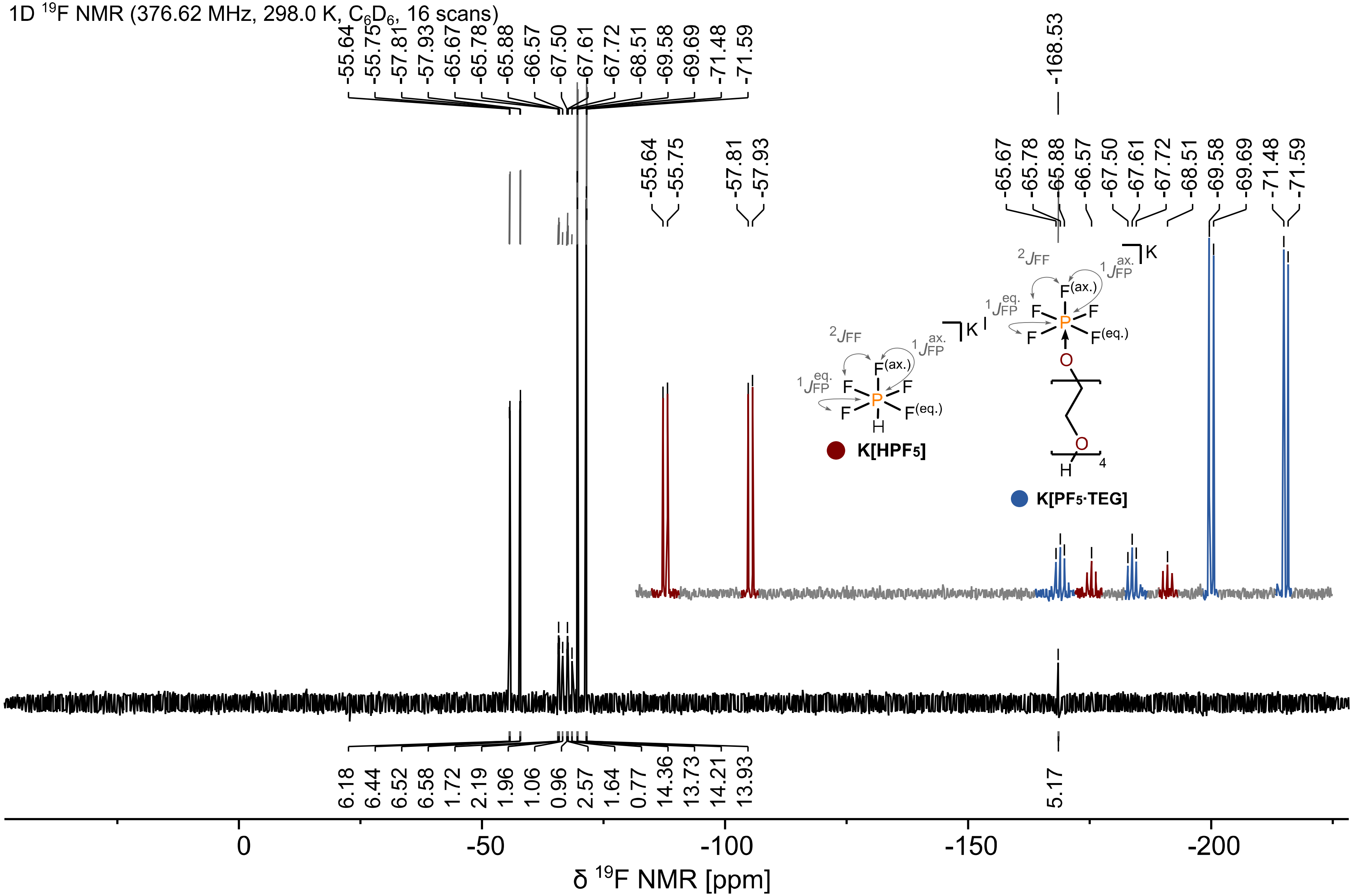

Figure S48. $^{19}F\{^{1}H\}$ NMR (376.46 MHz, MeCN, TEG, C_6D_6) corresponding to Table S15, entry 1 (conditions: 0.25 equiv. P_4, 5.0 equiv. DDQ, 7.5 equiv. KF, MeCN:TEG:C_6H_6 (8:1:1, 0.01 M), 18 h, 25 °C, N_2), showing the formation of K[PF_5·TEG] and K[HPF_5]. For details on chemical shifts and coupling pattern see main manuscript Figure 3. Integrals normalized to 100, to estimate K[PF_5·TEG]/K[HPF_5] ratio based on respective signals for equatorial fluorine atoms.[2,10]

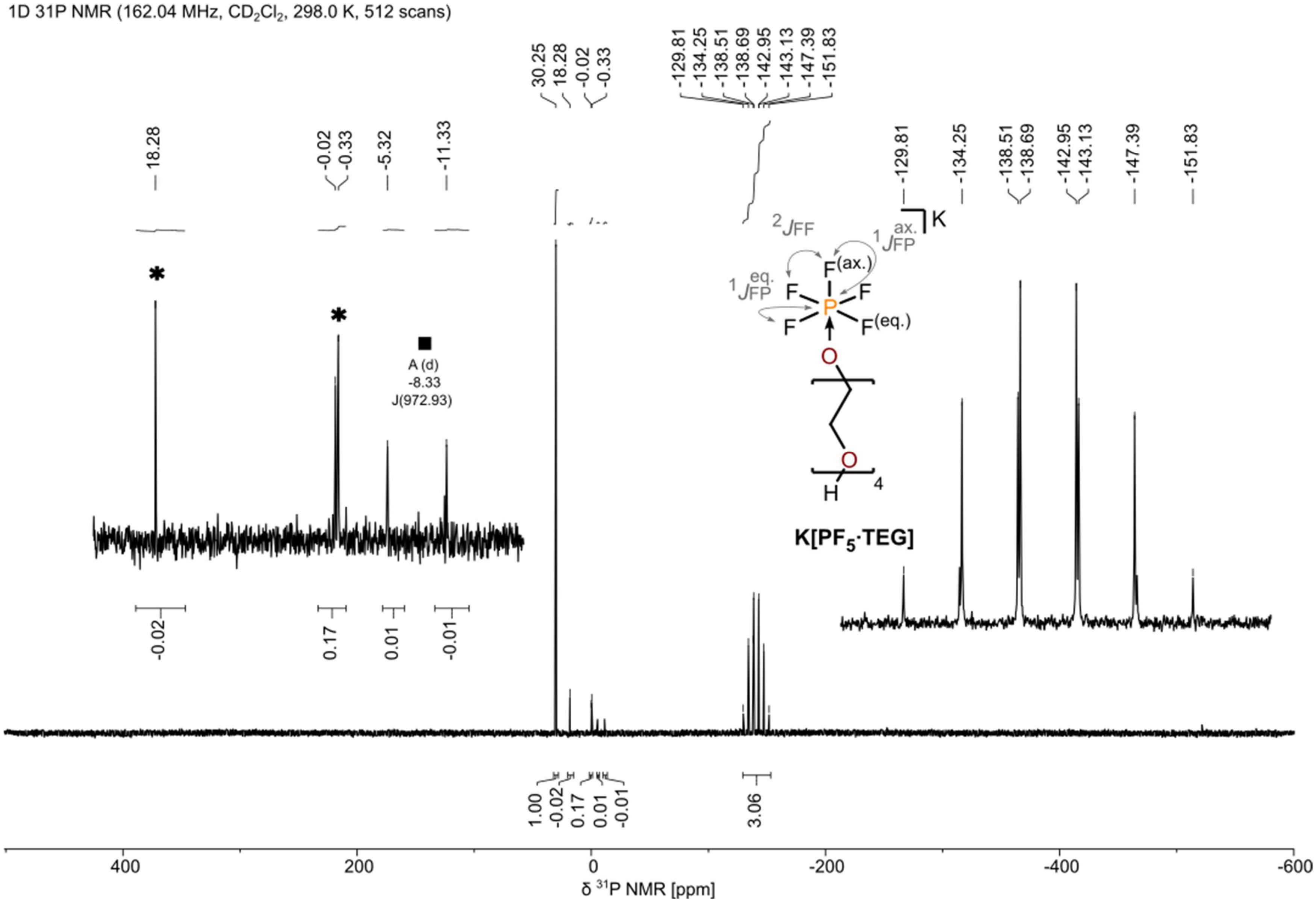

Figure S49. $^{31}P\{^{1}H\}$ NMR (161.97 MHz, CD_2Cl_2, TEG) corresponding to Table S15, entry 2 (conditions: 0.25 equiv. P_4, 5.0 equiv. DDQ, 7.5 equiv. KF, DCM-d_2:TEG:C_6H_6 (8:1:1, 0.01 M), 18 h, 25 °C, N_2), showing the selective formation of K[PF_5·TEG]. For further details on chemical shifts and coupling pattern see main manuscript Figure 3. (*) Unidentified species attributed to hydrolysis. (▪) Species attributed to solvolysis of the form $K_x[OP(O_x)(TEG)_{2-x}F]$ (x = 0, 1; δ – 8.3, d, 972.9 Hz), due to similar chemical shift and coupling constants of related species.[6]

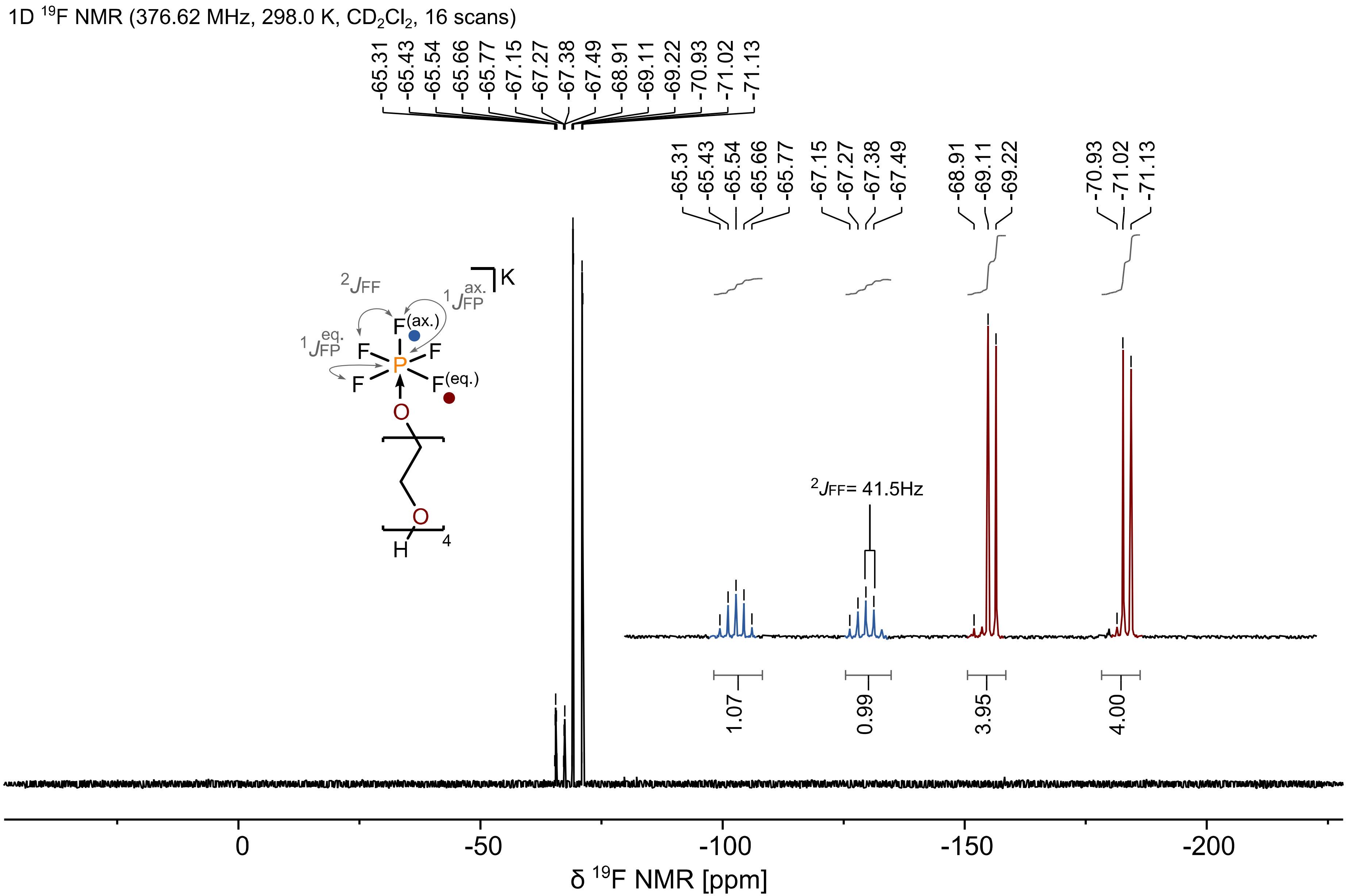

Figure S50. $^{19}F\{^1H\}$ NMR (376.46 MHz, CD_2Cl_2, TEG) corresponding to Table S15, entry 2 (conditions: 0.25 equiv. P_4, 5.0 equiv. DDQ, 7.5 equiv. KF, DCM-d_2:TEG:C_6H_6 (8:1:1, 0.01 M), 18 h, 25 °C, N_2), showing the selective formation of K[PF_5·TEG]. For further details on chemical shifts and coupling pattern see main manuscript Figure 3.

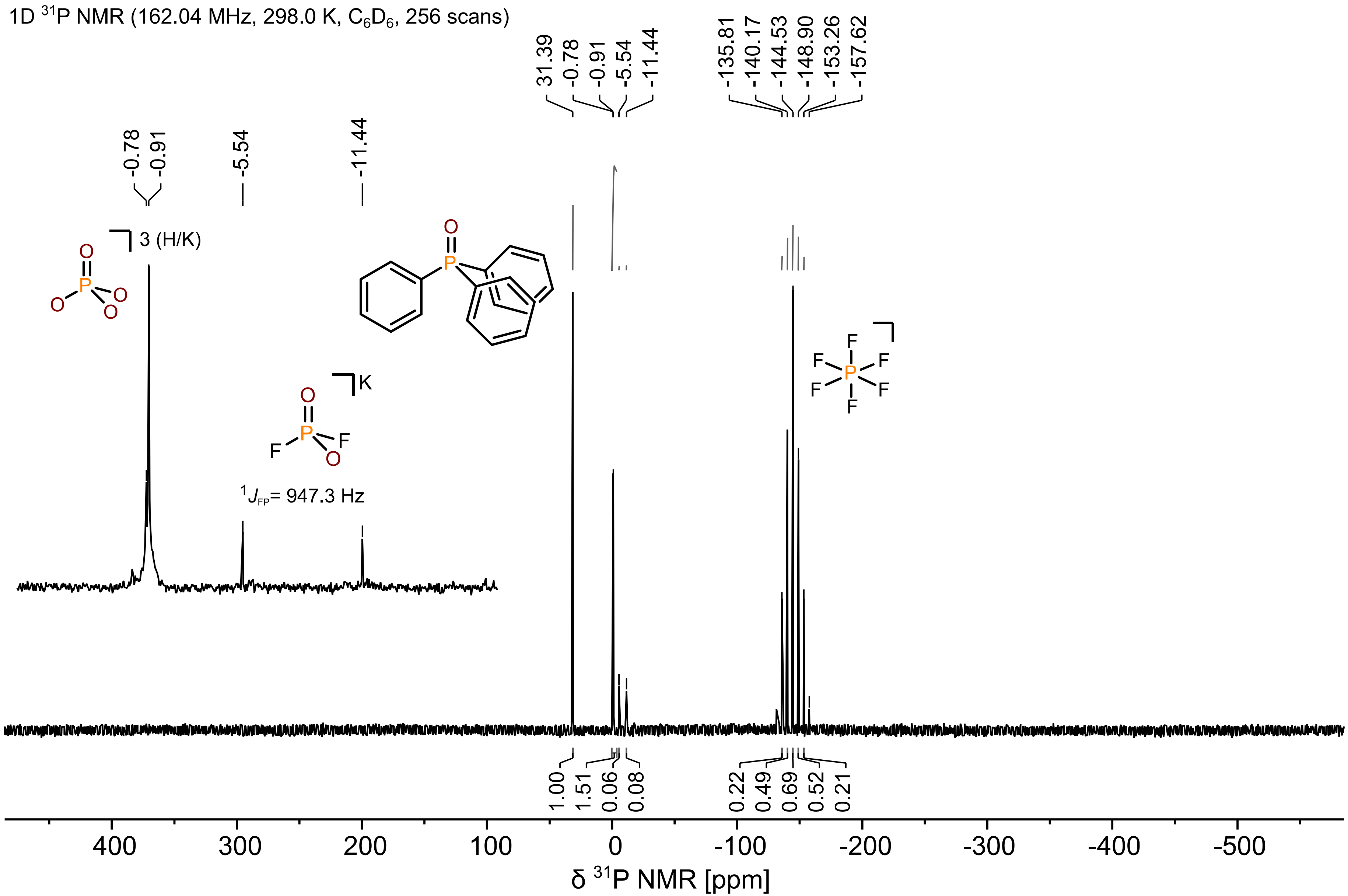

Figure S51. $^{31}P\{^{1}H\}$ NMR (161.97 MHz, MeCN, TEG, C_6D_6) corresponding to Table S16, entry 5 (conditions: 0.25 equiv. P_4, 5.0 equiv. DDQ, 7.5 equiv. KF, DCM-d_2:TEG:C_6H_6 (8:1:1, 0.01 M), 18 h, 65 °C, N_2), showing formation of KPF_6 alongside hydrolysis products PO_4^{3-} and KPO_2F_2.

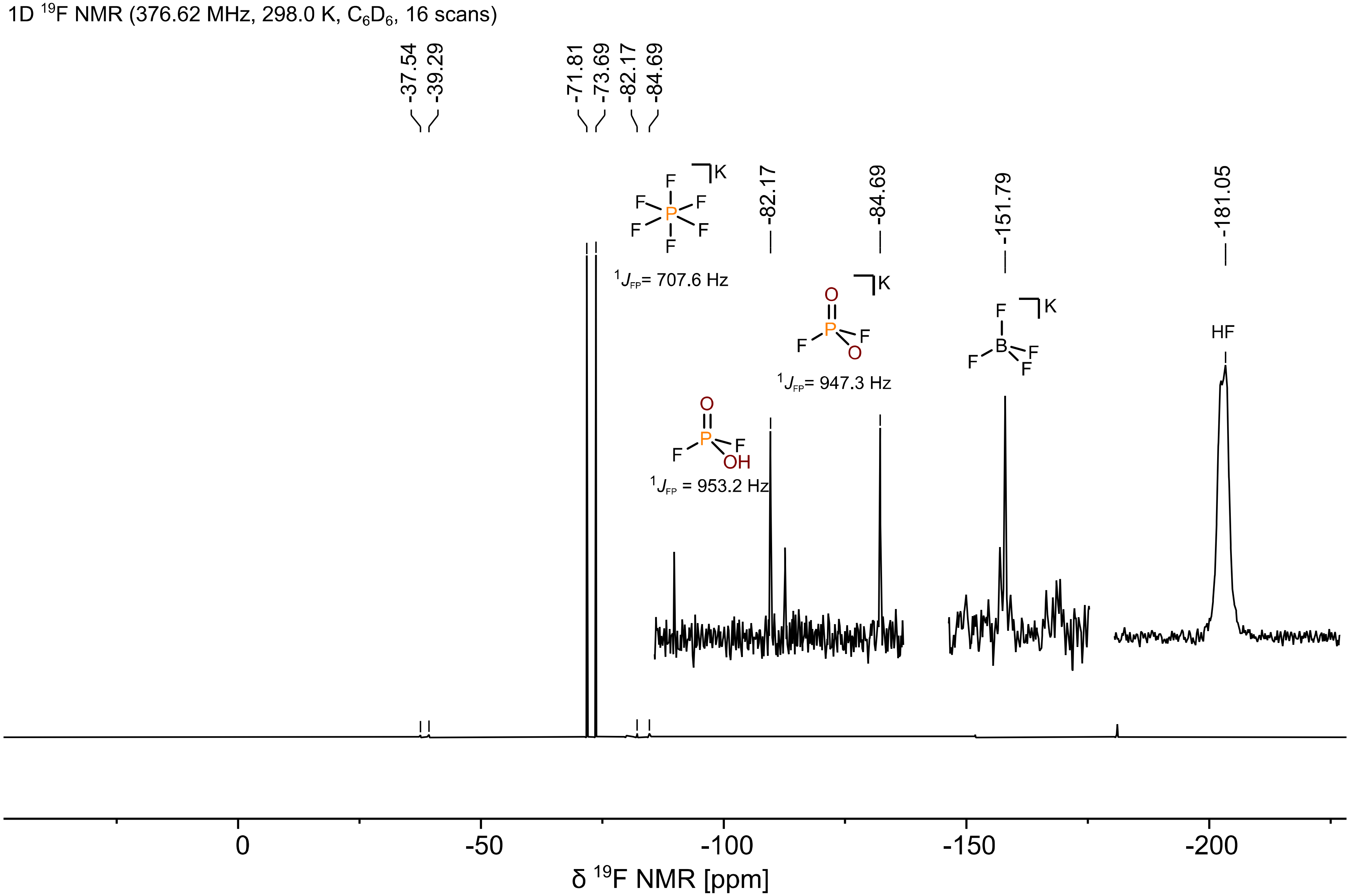

Figure S52. $^{19}F\{^{1}H\}$ NMR (376.46 MHz, MeCN, TEG, C_6D_6) corresponding to Table S16, entry 5 (conditions: 0.25 equiv. P_4, 5.0 equiv. DDQ, 7.5 equiv. KF, DCM-d_2:TEG:C_6H_6 (8:1:1, 0.01 M), 18 h, 65 °C, N_2), showing formation of KPF_6 alongside hydrolysis products PO_4^{3-} KPO_2F_2, HPO_2F_2 and HF.

4.8. Synthesis of $DDQH_2$

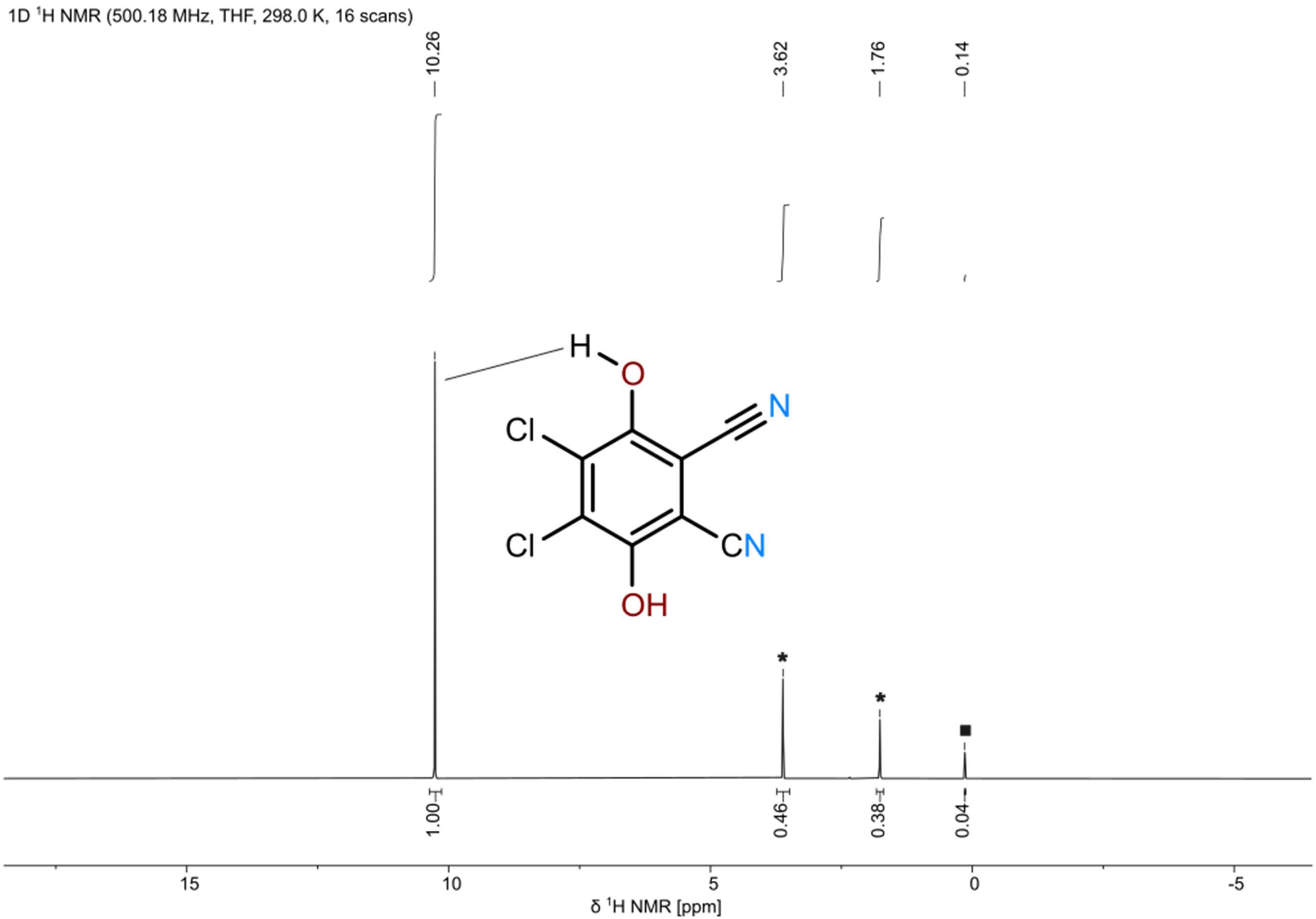

Figure S53. ¹H NMR (500.18 MHz, THF-d_8) of $DDQH_2$ (section S3.11). Residual solvent signals (*). Unidentified impurity (▪).

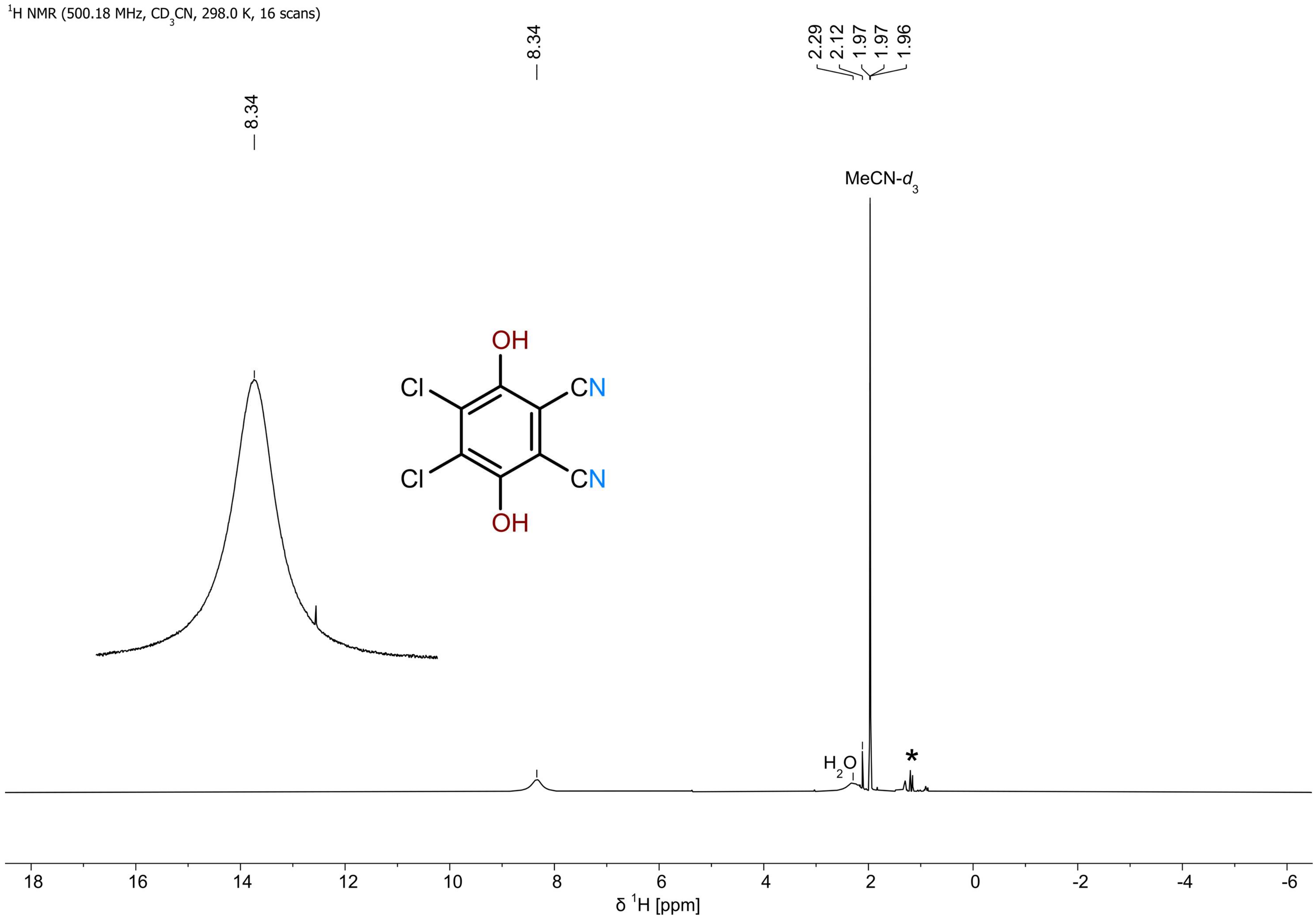

Figure S54. ^{1}H NMR (500.18 MHz, MeCN-d_3) of $DDQH_2$ (section S3.11). Unidentified impurities (*).

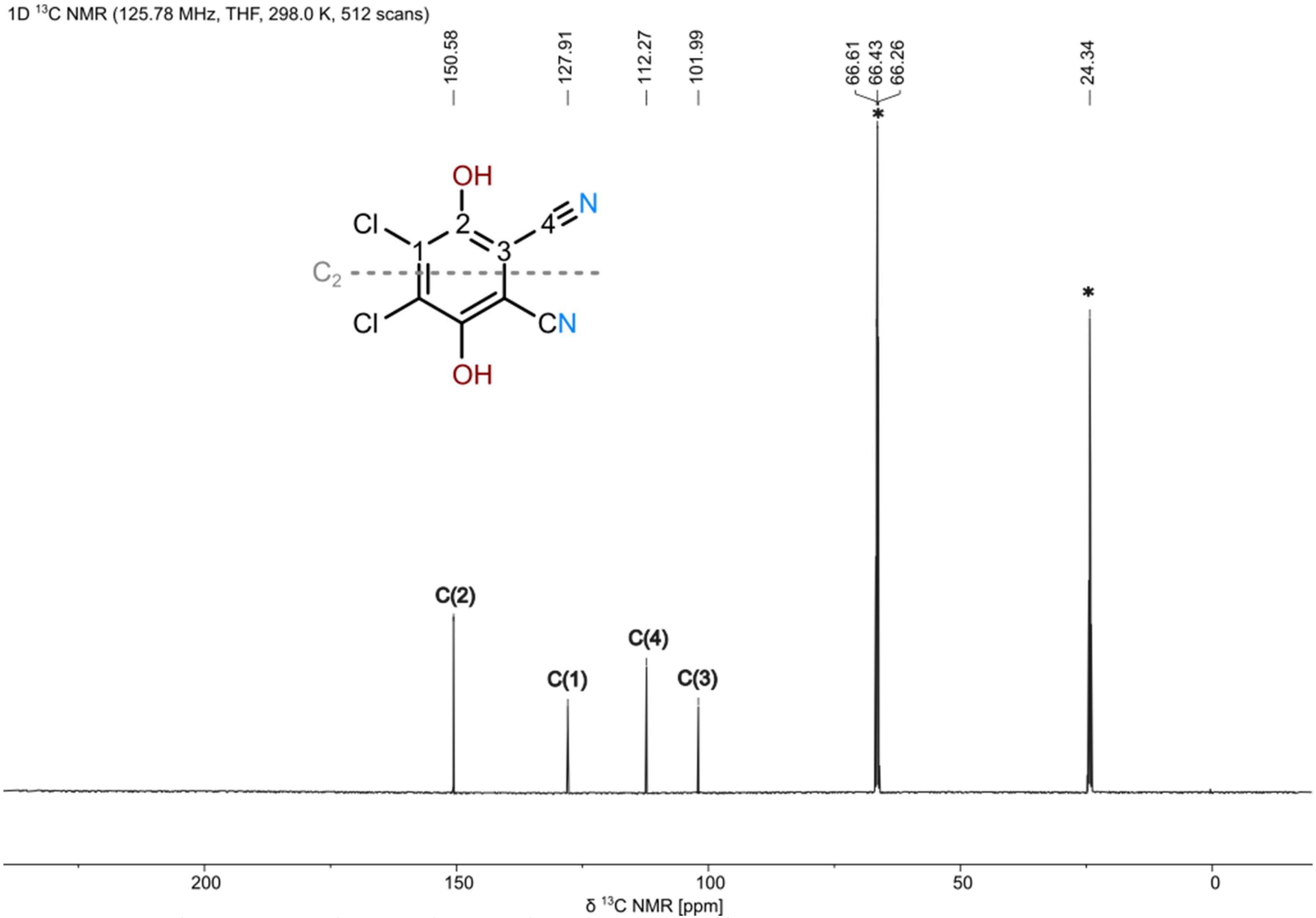

Figure S55. $^{13}C\{^{1}H\}$ NMR (125.87 MHz, THF-d_8) of $DDQH_2$ (section S3.10). Residual solvent signals (*).

4.9. Synthesis of P(DDQH)$_3$ from PCl$_3$ and DDQH$_2$

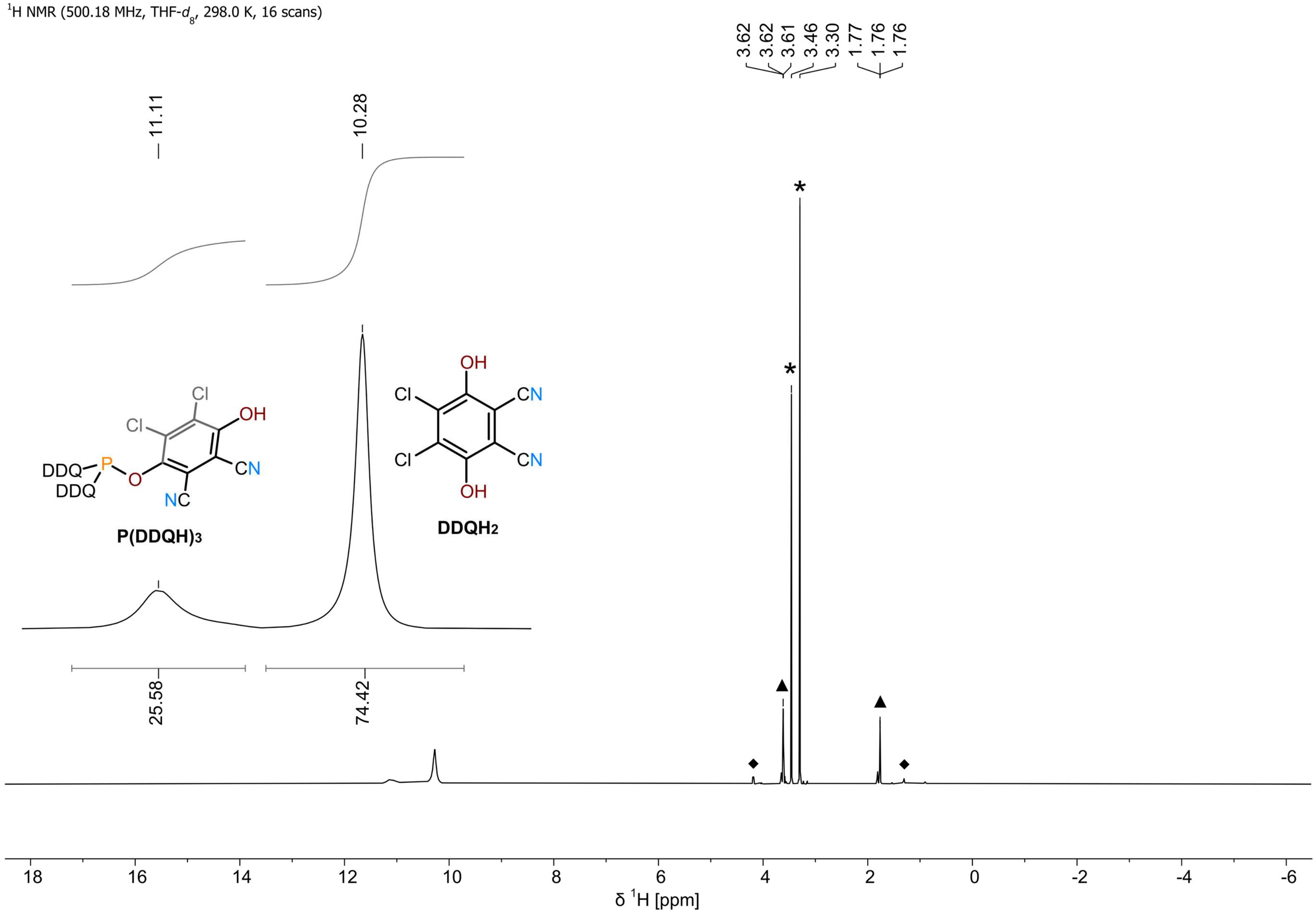

Figure S56. ^{1}H NMR (500.18 MHz, THF-d_8) of the reaction of PCl$_3$ with NaH and DDQH$_2$ (section S3.11; conditions: 1) 1.0 equiv. DDQH$_2$, 1.0 equiv. NaH, DME (100 mL), 20 °C, 19 h; 2) 0.33 equiv. PCl$_3$ (0.97 M in DCM), 20 °C, 18 h), showing OH-resonances of DDQH$_2$ (see Figure S53) and P(DDQH)$_3$. Residual solvent signals of THF-d_8 (▲). DME (*). Unidentified impurities (▪).

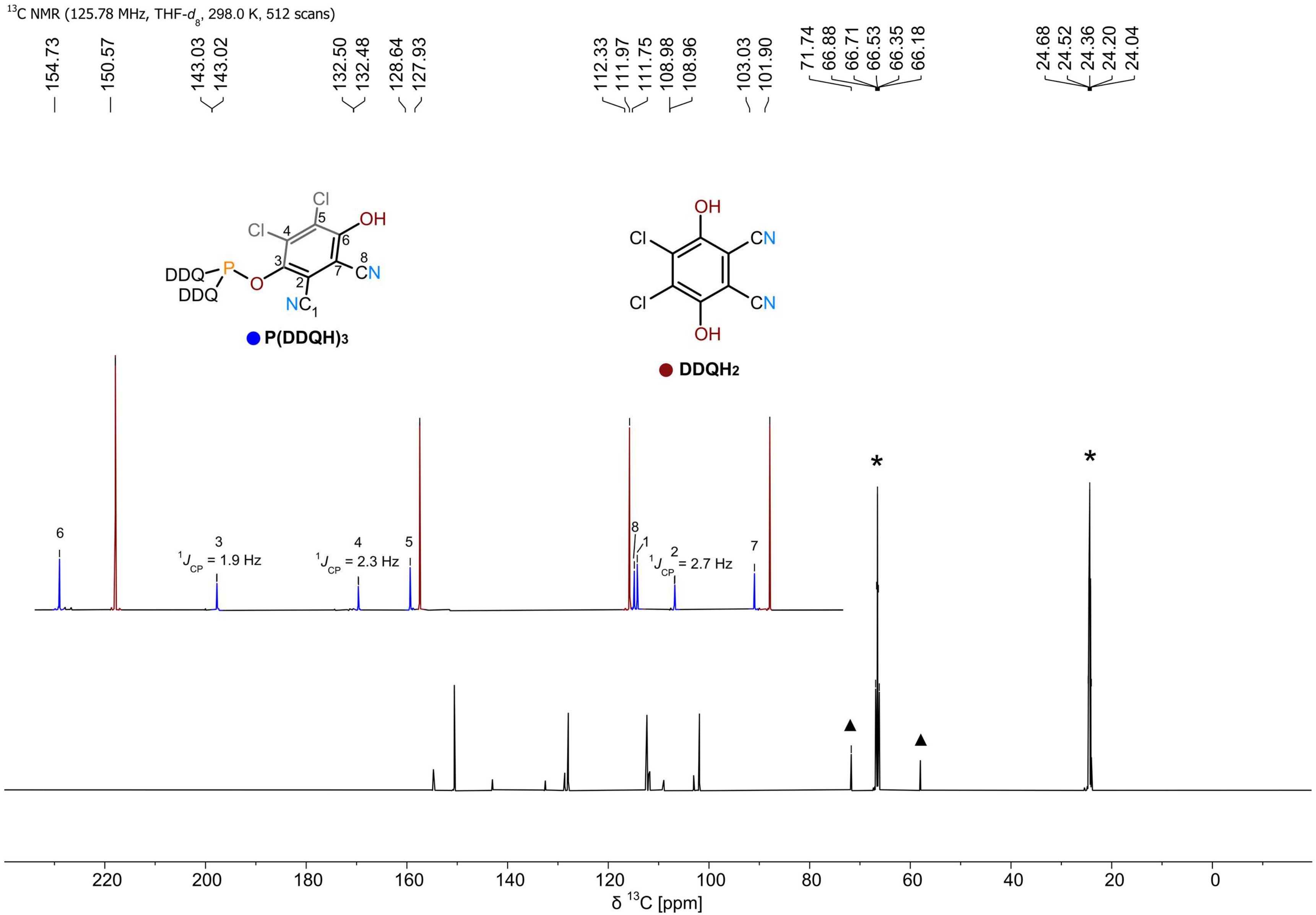

Figure S57. $^{13}C\{^{1}H\}$ NMR (125.78 MHz, THF-d_8) of the reaction of PCl_3 with NaH and $DDQH_2$ (section S3.11; conditions: 1) 1.0 equiv. $DDQH_2$, 1.0 equiv. NaH, DME (100 mL), 20 °C, 19 h; 2) 0.33 equiv. PCl_3 (0.97 M in DCM), 20 °C, 18 h), with carbon atoms and $^{1}J_{CP}$ coupling constants assigned for P(DDQH)3 (for assignment of $DDQH_2$ see Figure S55). Assignment of carbon atoms in P(DDQH)3 is derived from relative chemical shift with respect to the carbon resonances in $DDQH_2$ and the observation of $^{1}J_{CP}$ coupling. Residual solvent signals of THF-d_8 (*). DME (▲).

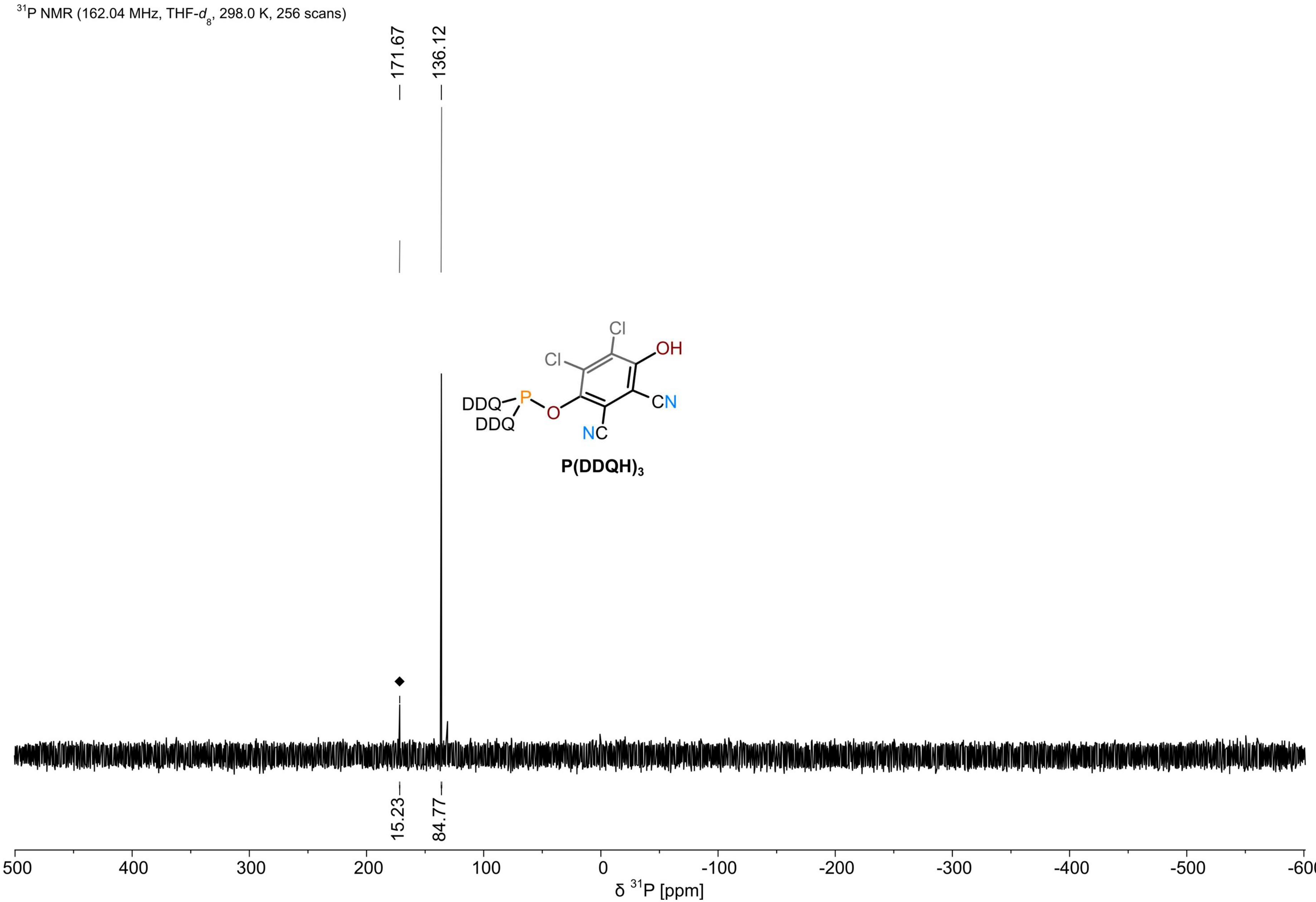

Figure S58 $^{31}P\{^1H\}$ NMR (162.04 MHz, THF-d_8) of the reaction of PCl_3 with NaH and $DDQH_2$ (section S3.11; conditions: 1) 1.0 equiv. $DDQH_2$, 1.0 equiv. NaH, DME (100 mL), 20 °C, 19 h; 2) 0.33 equiv. PCl_3 (0.97 M in DCM), 20 °C, 18 h). Impurity assigned as $Cl_2P(DDQH)$ or $ClP(DDQH)_2$ (♦) according to chemical shifts reported for similar chlorophosphites.[13,14]

4.10. Studies on the Formation of P(DDQH)₃

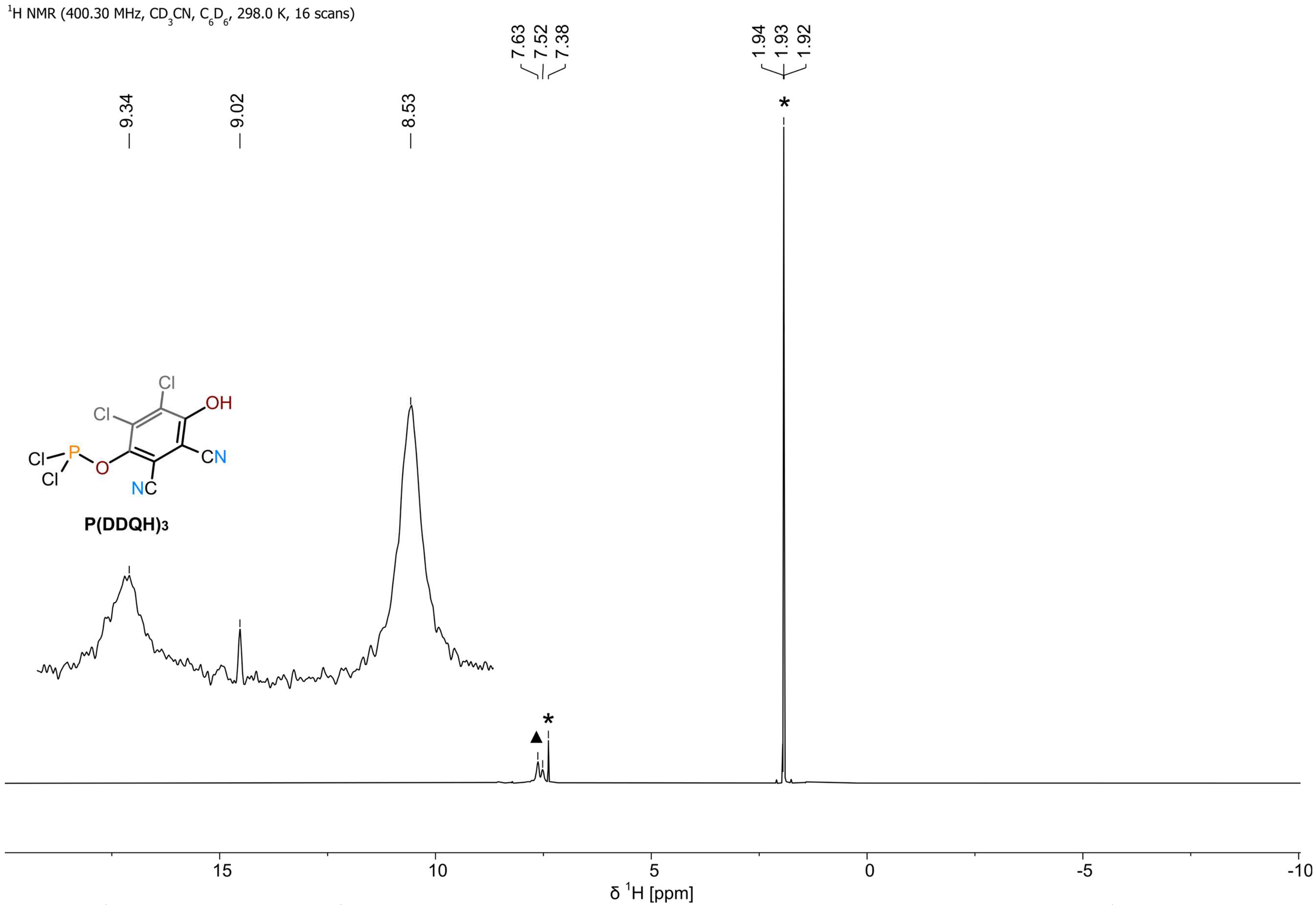

Figure S59. ^{1}H NMR (500.18 MHz, MeCN-d_3, C_6D_6) of the reaction between DDQ and P_4 in fully deuterated solvents with TPPO as internal standard. (conditions: 0.25 equiv. P_4, 5.0 equiv. DDQ, MeCN-d_3:C_6D_6 (9:1, 0.01 M), 18 h, 65 °C, N_2), showing two distinct broad resonances at 8.53 and 9.34 ppm attributed to the O–H resonances of P(DDQ)$_3$ and DDQH$_2$. Residual solvent signals (*). Aryl-protons of internal standard TPPO (▲).

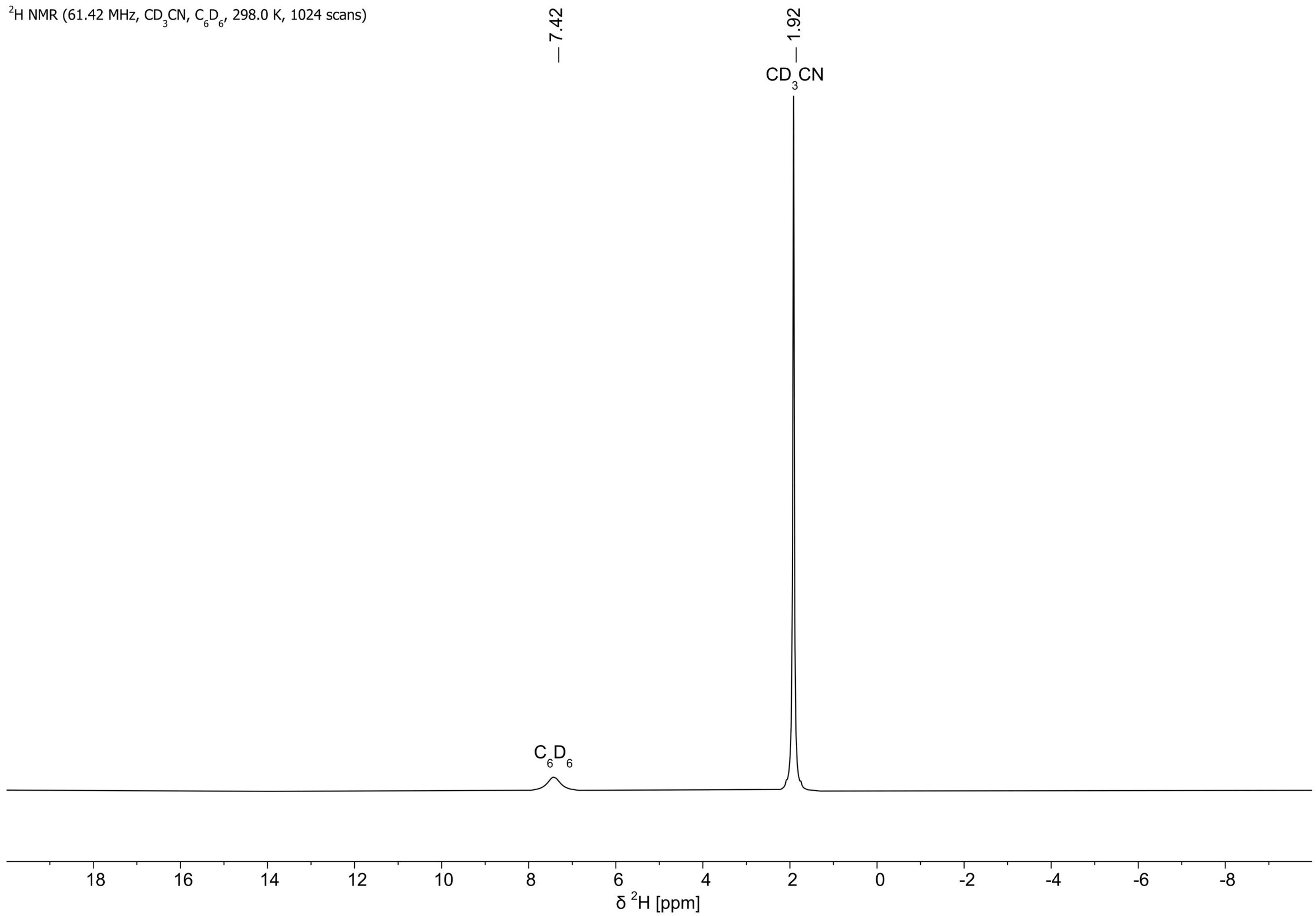

Figure S60. ^{2}H NMR (61.42 MHz, MeCN-*d*$_3$, C_6D_6) of the reaction between DDQ and P_4 in fully deuterated solvents with TPPO as internal standard. (conditions: 0.25 equiv. P_4, 5.0 equiv. DDQ, MeCN-*d*$_3$:C_6D_6 (9:1, 0.01 M), 18 h, 65 °C, N_2), showing no signals besides the resonances of the solvents MeCN-*d*$_3$ and C_6D_6 indicating that no deuterium incorporation into P(DDQH)$_3$ from the solvents took place.

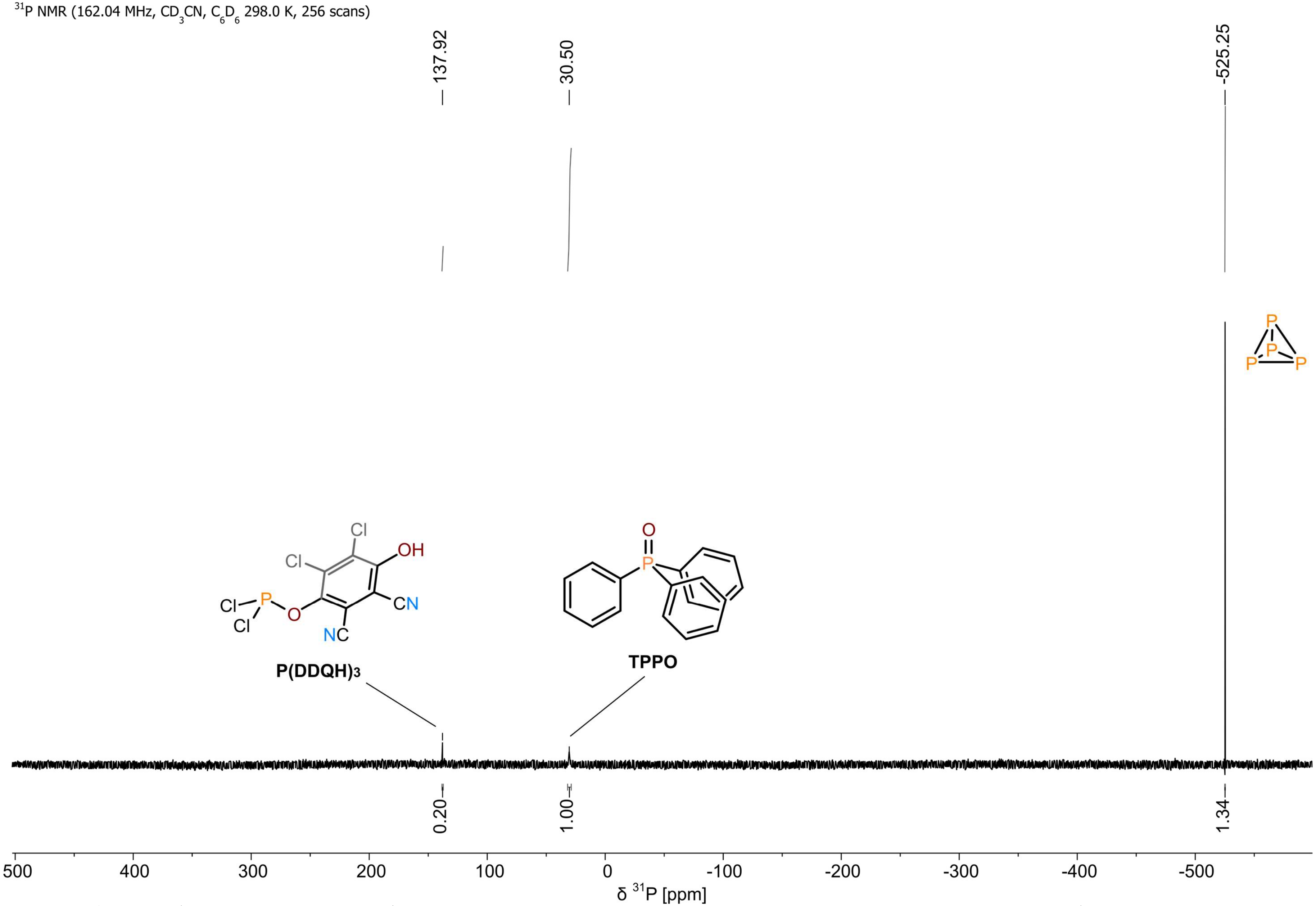

Figure S61. $^{31}P\{^{1}H\}$ NMR (162.04 MHz, MeCN-d_3, C_6D_6) of the reaction between DDQ and P_4 in fully deuterated solvents with TPPO as internal standard. (conditions: 0.25 equiv. P_4, 5.0 equiv. DDQ, MeCN-d_3:C_6D_6 (9:1, 0.01 M), 18 h, 65 °C, N_2).

4.11. Studies on Reactivity of P_4/DDQ Reaction Mixture with KF

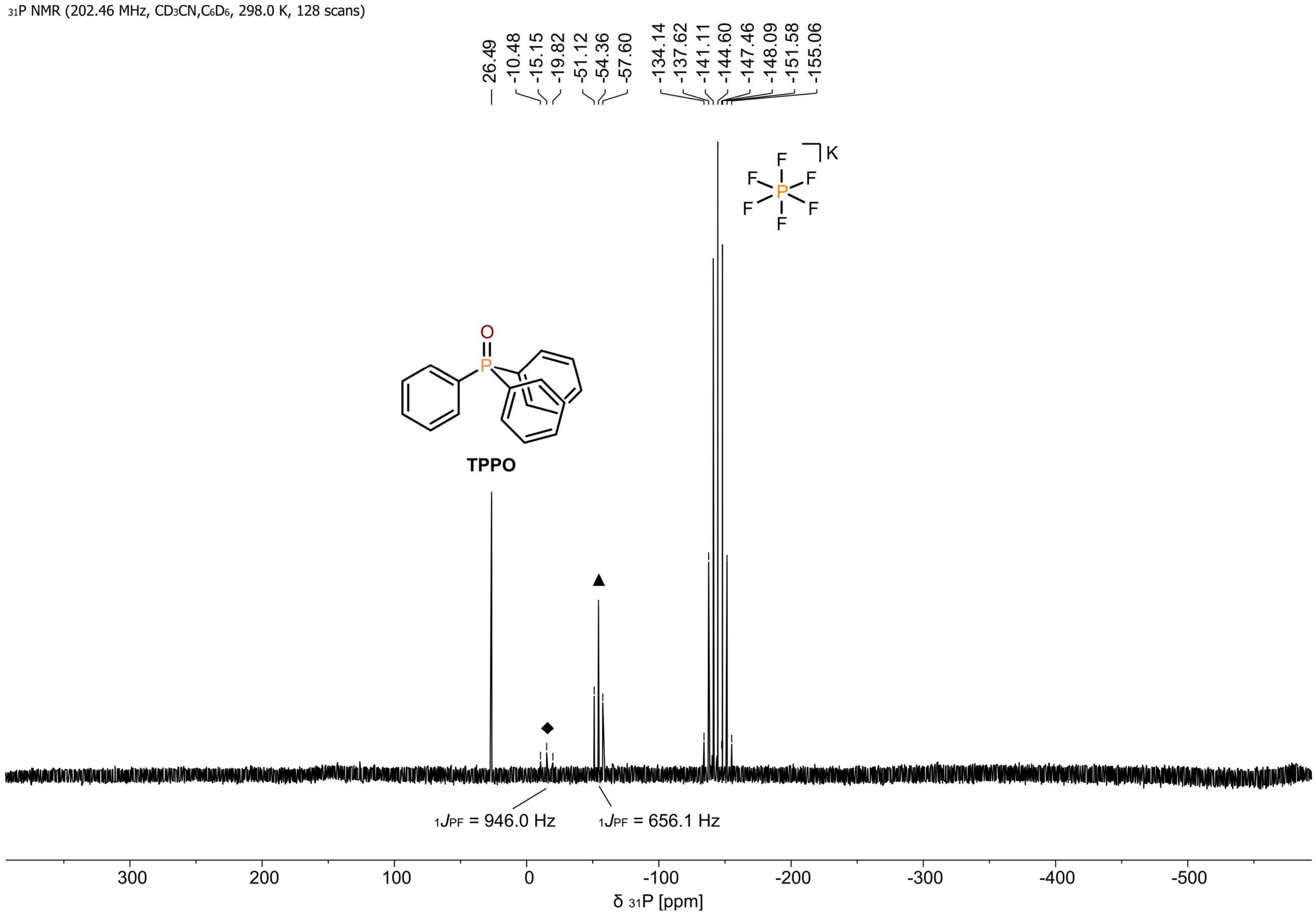

Figure S62. $^{31}P\{^1H\}$ NMR (162.04 MHz, MeCN-d_3, C_6D_6) of the reaction mixture shown in Figure S61 after addition of KF and further heating for 18 h. (conditions: initial reaction: 0.25 equiv. P_4, 5.0 equiv. DDQ, MeCN-d$_3$:C_6D_6 (9:1, 0.01 M), 18 h, 65 °C, N_2; subsequent addition: 7.5 equiv. KF, 18 h, 65 °C, N_2). The spectrum shows formation of KPF_6 from the initially NMR-silent phosphorus-containing species, alongside KPO_2F_2 (♦) and an unidentified "PF_2"-species (▲). Signal assignments are based on chemical shifts, coupling constants, and coupling patterns, either by comparison with reported literature data or, where no direct precedent exists, by analogy to closely related species.[2,10]

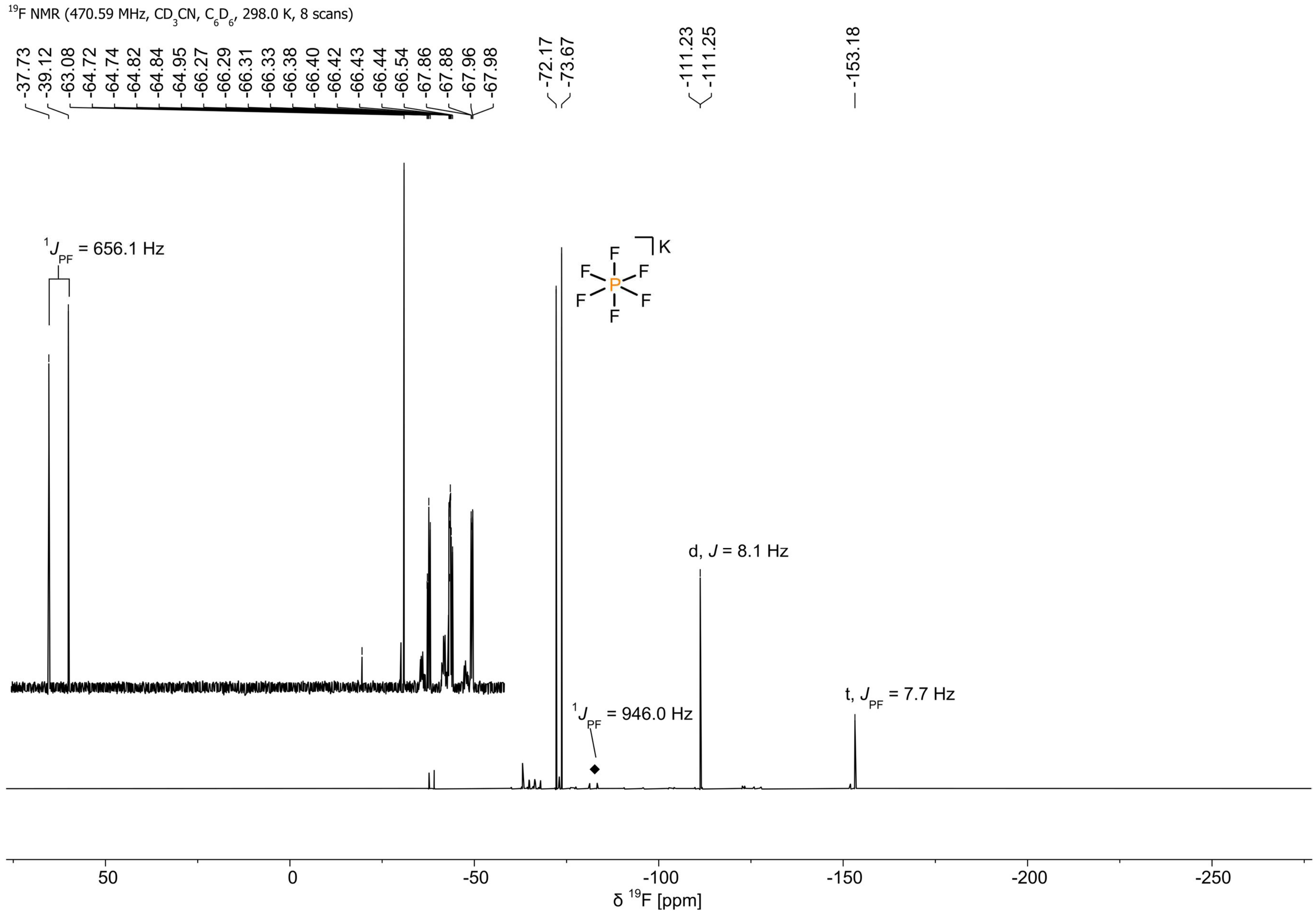

Figure S63. $^{19}F\{^{1}H\}$ NMR (376.46 MHz, MeCN, C_6D_6) of the reaction mixture shown in Figure S61 after addition of KF and further heating for 18 h. (conditions: initial reaction: 0.25 equiv. P_4, 5.0 equiv. DDQ, MeCN-d_3:C_6D_6 (9:1, 0.01 M), 18 h, 65 °C, N_2; subsequent addition: 7.5 equiv. KF, 18 h, 65 °C, N_2). The spectrum shows formation of KPF_6 from the initially NMR-silent phosphorus-containing species, alongside KPO_2F_2 (◆) and an unidentified "PF_2"-species (▲) and multiple unidentified fluorinated side products. Where relevant, resonances are annotated with multiplicities and coupling constants.

4.12. Reaction of K[DDQ˙] with P_4

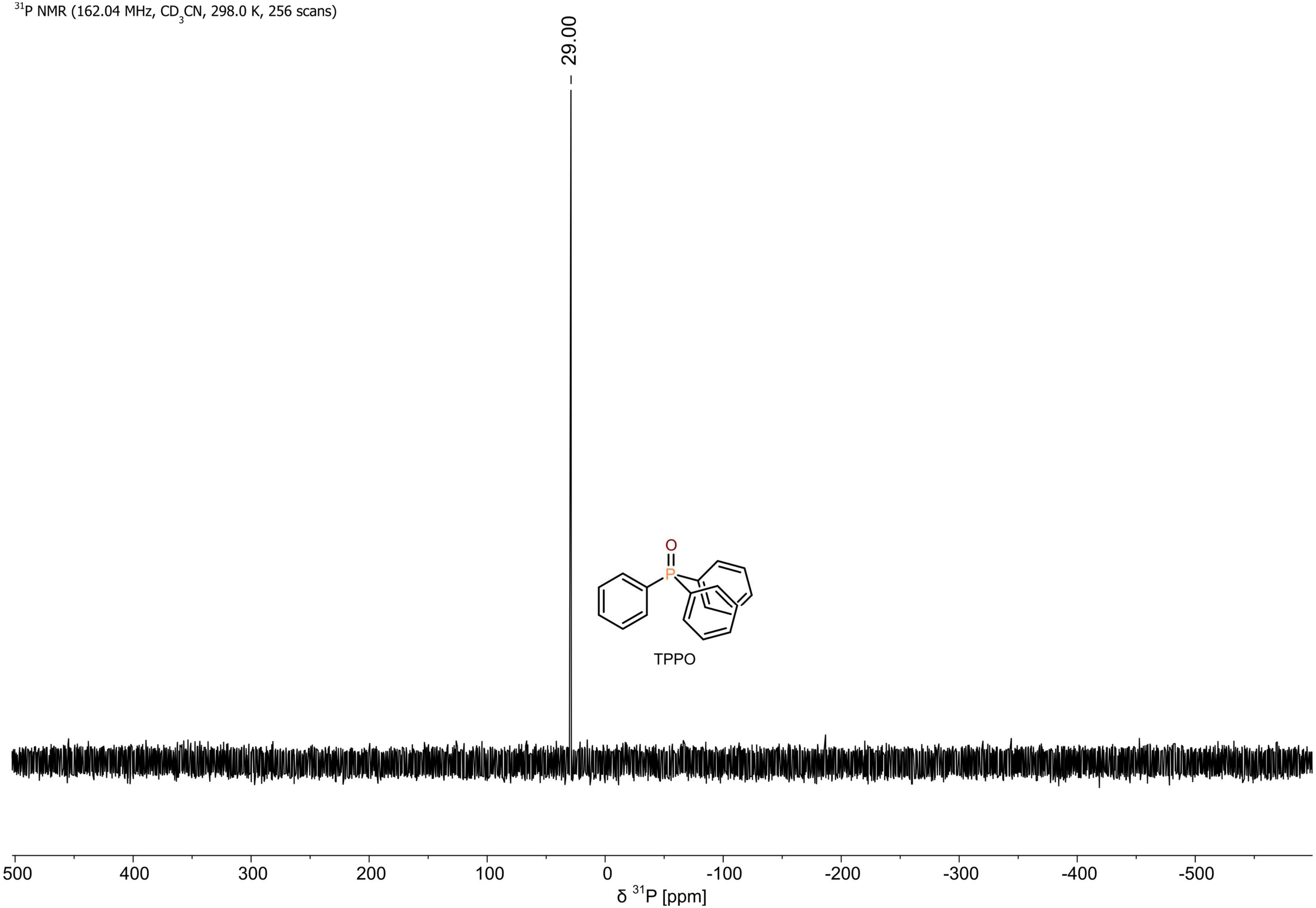

Figure S64. $^{31}P\{^1H\}$ NMR (161.97 MHz, MeCN-d_3, C_6D_6) corresponding to Table S18, entry 1 (conditions: 0.25 equiv. P_4, 5.0 equiv. K[DDQ˙], MeCN-d_3:C_6D_6 (9:1, 0.01 M), 18 h, 65 °C, N_2).

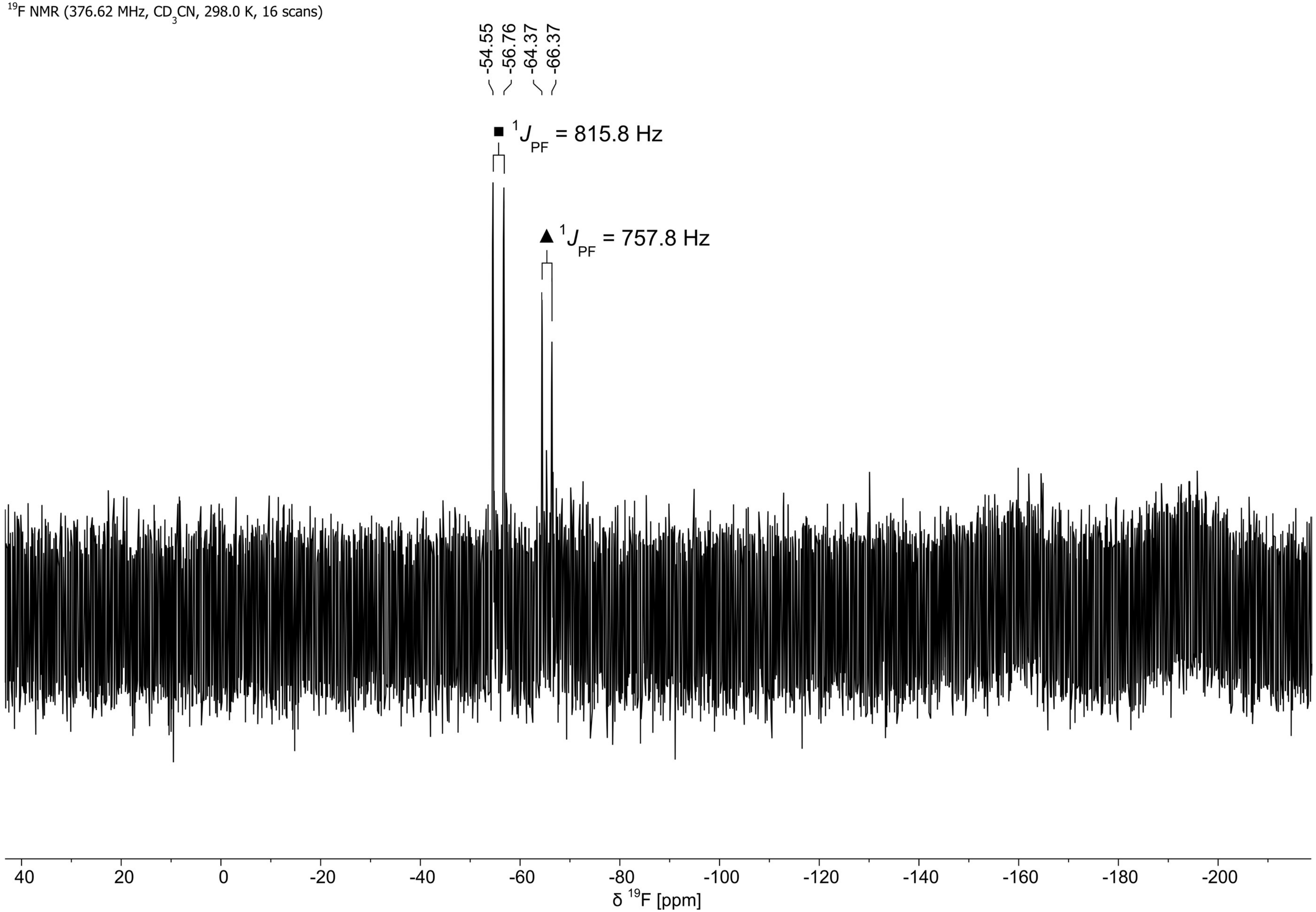

Figure S65. $^{19}F\{^{1}H\}$ NMR (376.46 MHz, MeCN, C_6D_6) corresponding to Table S18, entry 2 (conditions: 0.25 equiv. P_4, 5.0 equiv. K[DDQ·], 7.5 equiv. KF, MeCN-d_3:C_6D_6 (9:1, 0.01 M), 18 h, 65 °C, N_2), showing the formation of two unidentified fluorinated phosphorus species (▪,▲). The observed coupling constants are assigned as $^{1}J_{PF}$ couplings, consistent with values typically observed for direct P(V)–F bonds.[2]

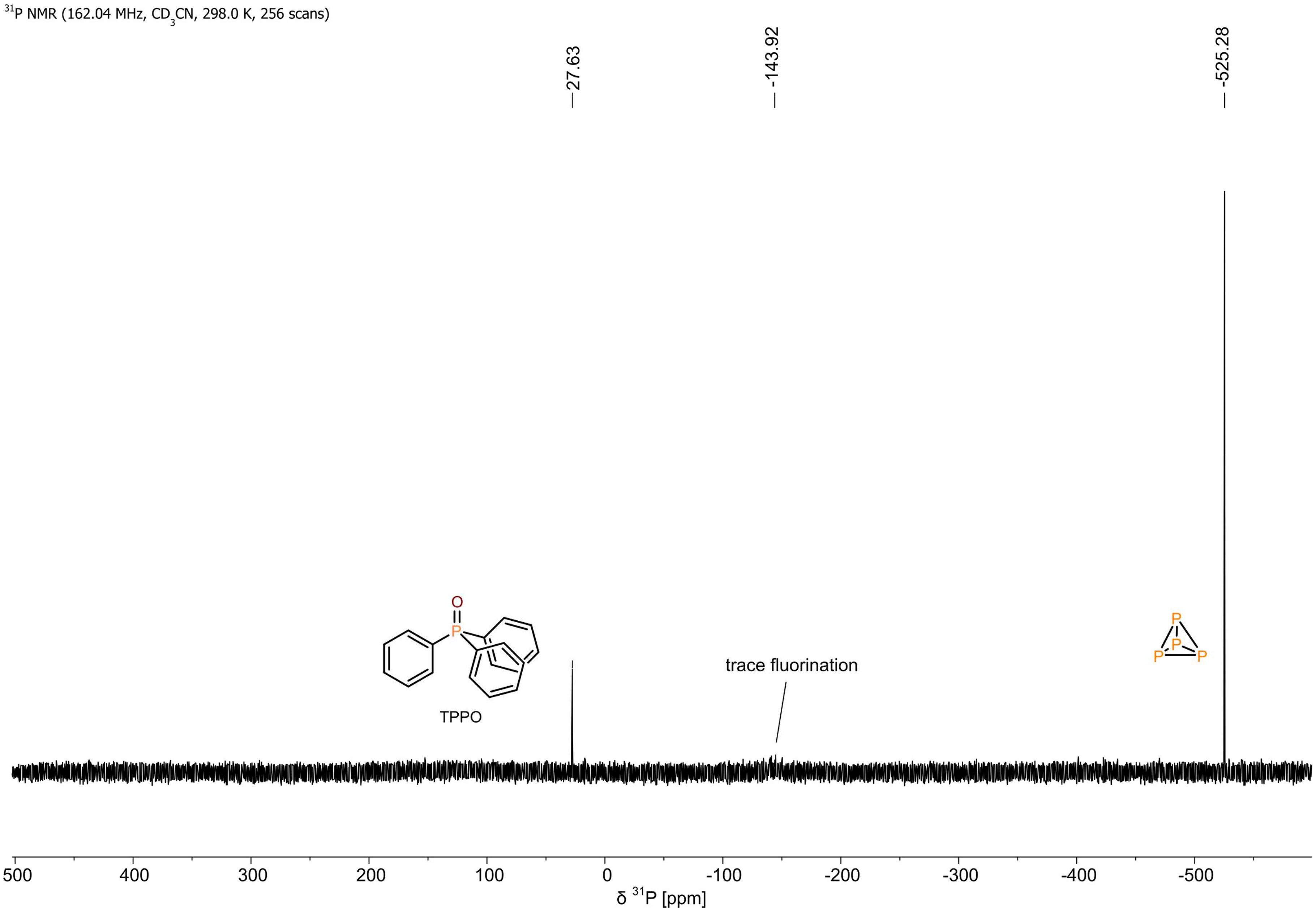

Figure S66. $^{31}P\{^1H\}$ NMR (161.97 MHz, MeCN-d_3, C_6D_6) corresponding to Table S18, entry 2 (conditions: 0.25 equiv. P_4, 5.0 equiv. K[DDQ$^{\cdot}$], 7.5 equiv. KF, MeCN-d_3:C_6D_6 (9:1, 0.01 M), 18 h, 65 °C, N_2).

4.13. Reaction of DDQ with KF

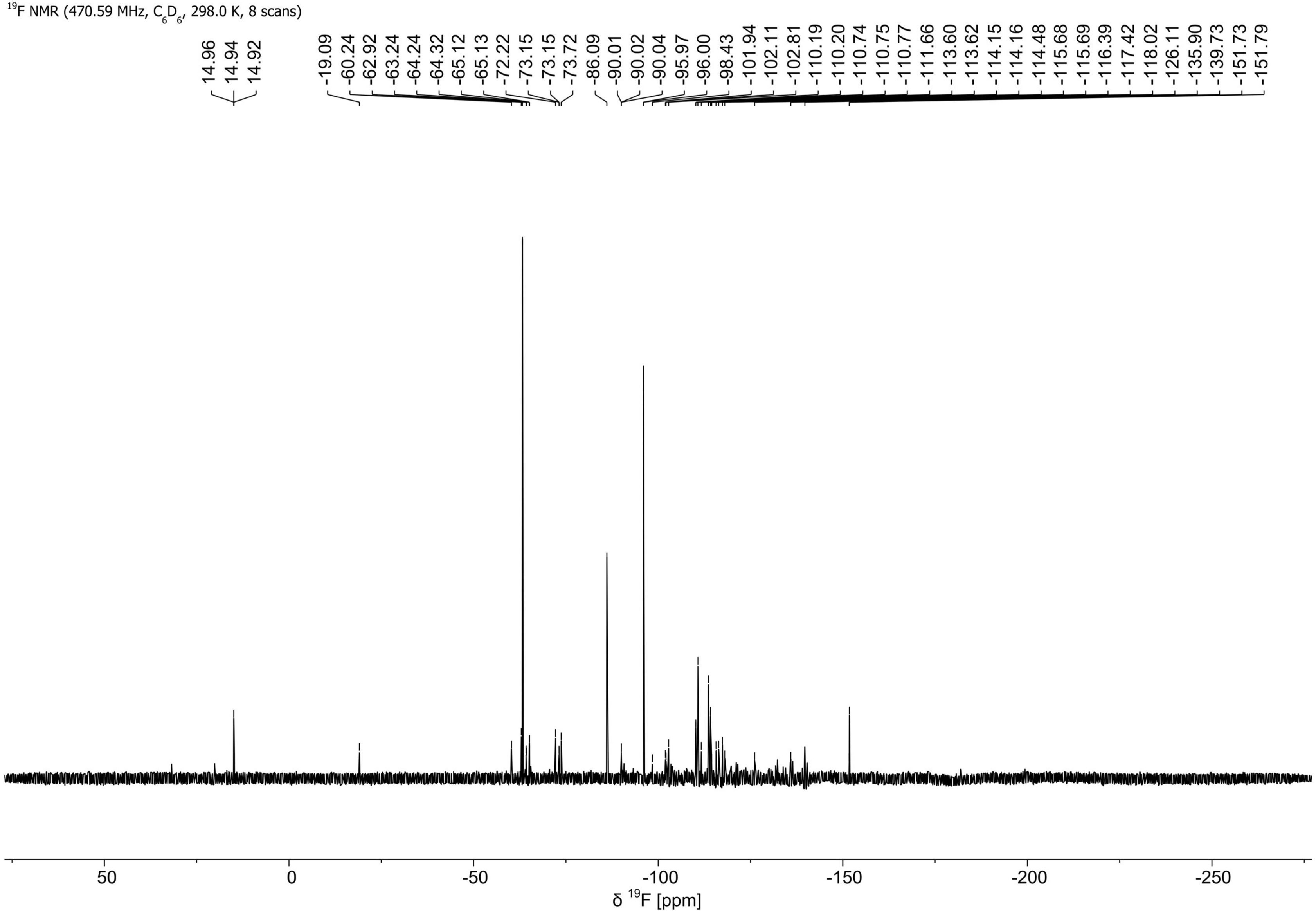

Figure S67. $^{19}F\{^{1}H\}$ NMR (376.62 MHz, MeCN, C_6D_6) of the reaction between DDQ and KF (conditions 1.0 equiv. DDQ, 1.5 equiv. KF, MeCN (0.2 M), 18 h, 65 °C).

4.14. In situ NMR Reaction Monitoring Experiments

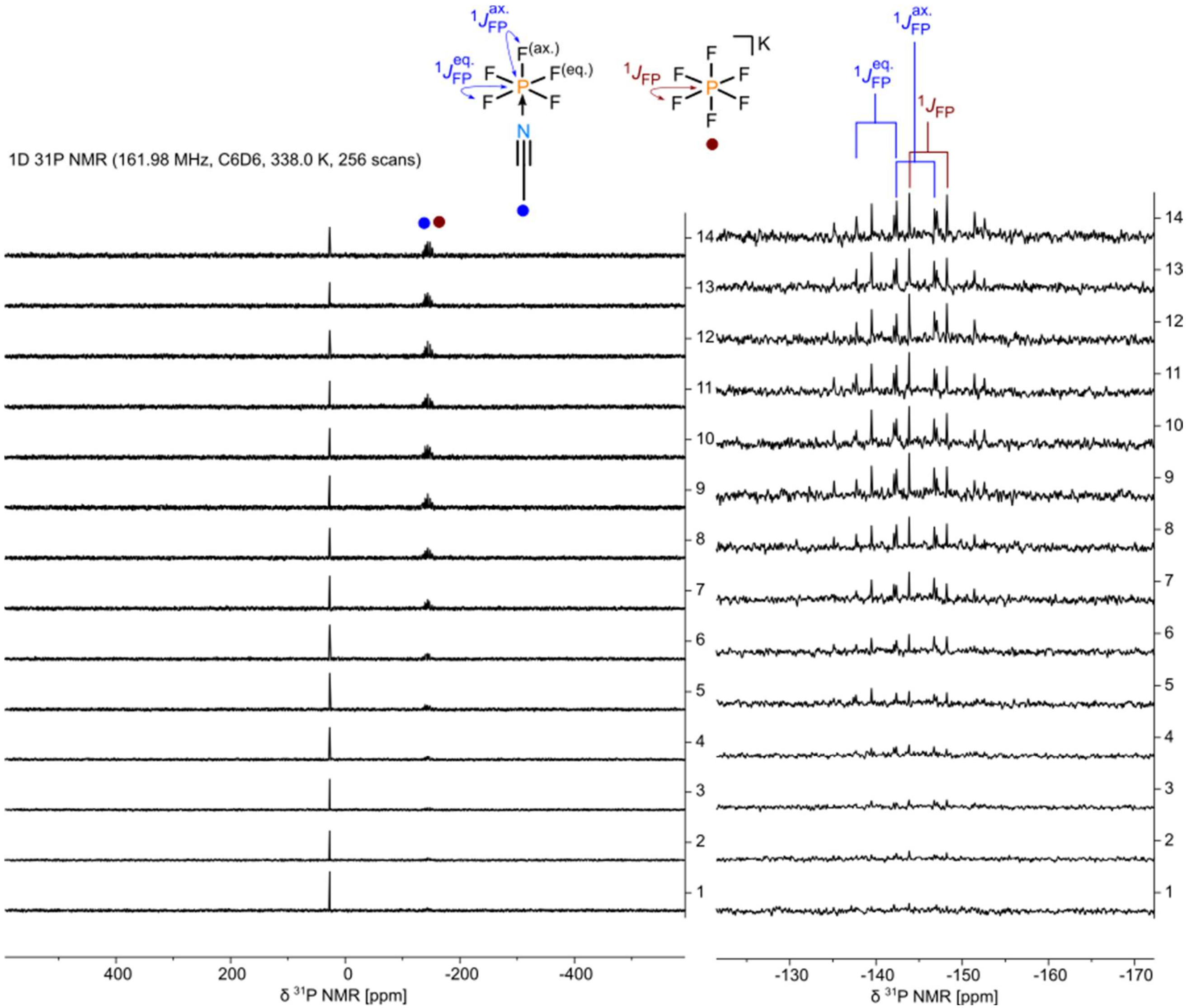

Figure S68. In situ $^{31}P\{^1H\}$ NMR (161.98 MHz, MeCN, C_6D_6) monitoring of the reaction at 65 °C using TPPO (δ 27.3 ppm, s) as an internal standard over 21 h (conditions: 0.25 equiv. P_4, 5.0 equiv. DDQ, 7.5 equiv. KF, MeCN:C_6H_6 (9:1, 0.01 M), 18 h, 65 °C, N_2; Δt = 1 h until t = 5 h, then Δt = 2 h). The inset highlights the formation of perfluorinated P(V) species, among them PF_5·MeCN (δ – 145.1 ppm, dp, $^1J_{PF(eq.)}$ = 755.3, $^1J_{PF(ax.)}$ = 710.8 Hz) KPF_6 (δ – 146.0 ppm, sept., ,$^1J_{PF}$ = 707.1 Hz). Due to low signal to noise ratio other P(V) species could not be assigned unambiguously.

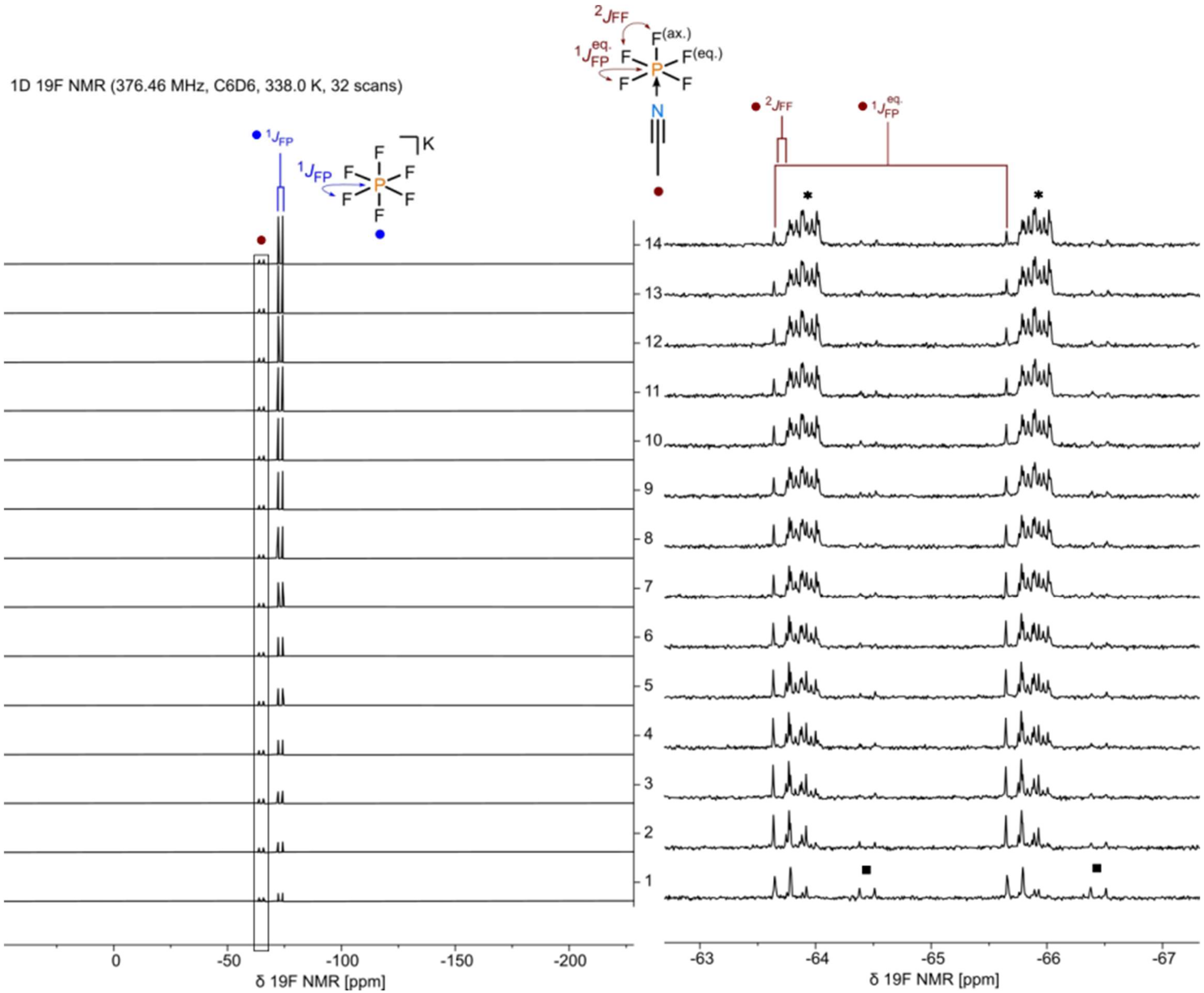

Figure S69. In situ $^{19}F\{^{1}H\}$ NMR (376.48 MHz, MeCN, C_6D_6) monitoring of the reaction at 65 °C over 21 h (conditions: 0.25 equiv. P_4, 5.0 equiv. DDQ, 7.5 equiv. KF, MeCN:C_6H_6 (9:1, 0.01 M), 18 h, 65 °C, N_2; Δt = 1 h until t = 5 h, then Δt = 2 h). The inset highlights the formation of perfluorinated P(V) species, among them KPF_6 and PF_5·MeCN (δ – 64.6 ppm, dd, $^{1}J_{FP}$ = 755.3, $^{2}J_{FF}$ = 51.0 Hz, $F^{(eq.)}$). Unidentified PF_5 Lewis-base adducts PF_5·LB (*) appear as overlapping signals and could not be further resolved. Minor unidentified PF_5·LB adduct (▪) which is quickly consumed (δ – 65.5 ppm, dd, $^{1}J_{FP}$ = 753.1, $^{2}J_{FF}$ = 49.5 Hz, $F^{(eq.)}$).

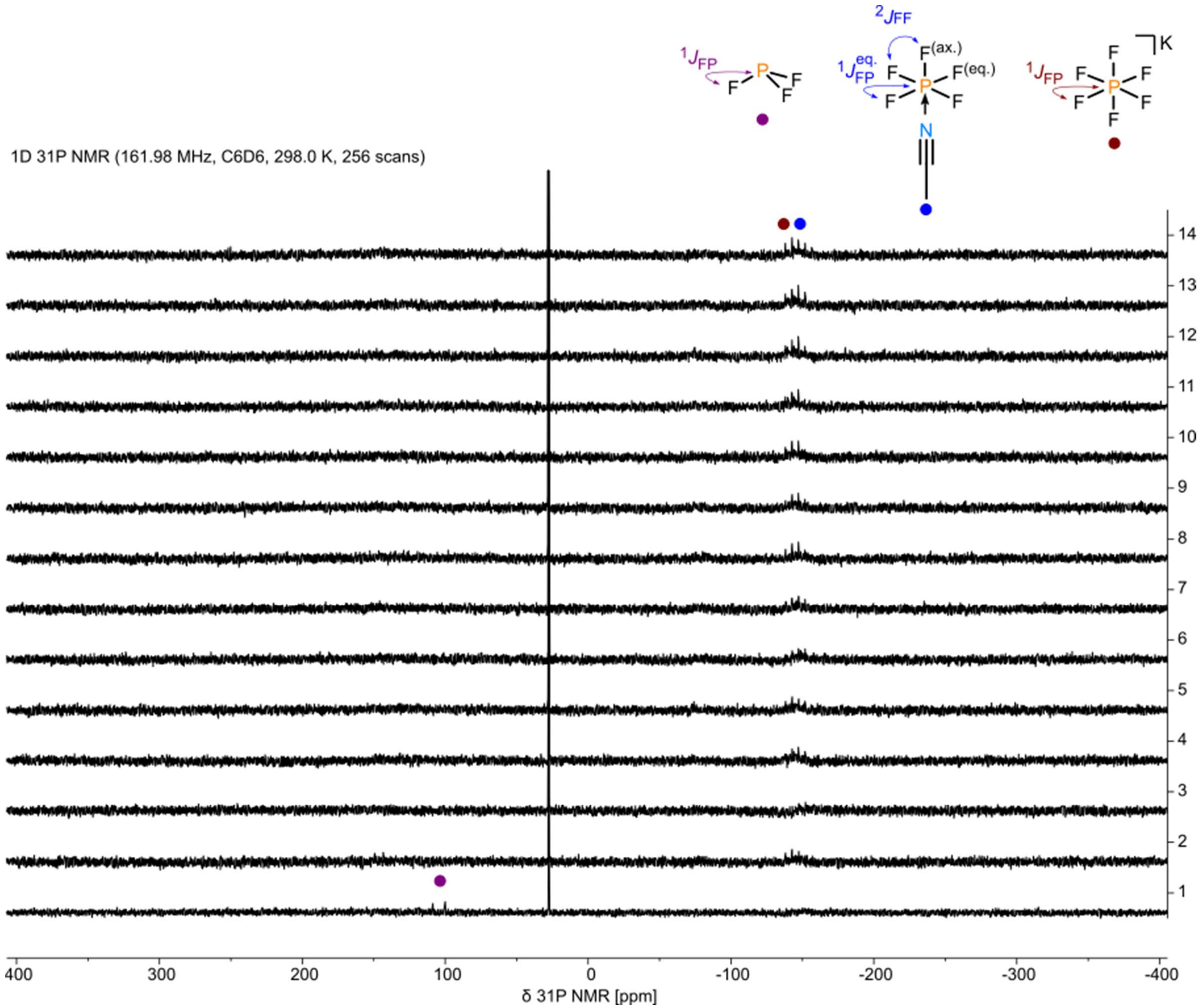

Figure S70. In situ $^{31}P\{^{1}H\}$ NMR (161.98 MHz, MeCN, C_6D_6) monitoring of the reaction at 25 °C using TPPO as an internal standard (δ 27.3 ppm, s) over 21 h (conditions: 0.25 equiv. P_4, 5.0 equiv. DDQ, 7.5 equiv. KF, MeCN:C_6H_6 (9:1, 0.01 M), 18 h, 25 °C, N_2; Δt = 1 h until t = 5 h, then Δt = 2 h), showing formation of traces of PF_3, PF_5·MeCN and KPF_6.

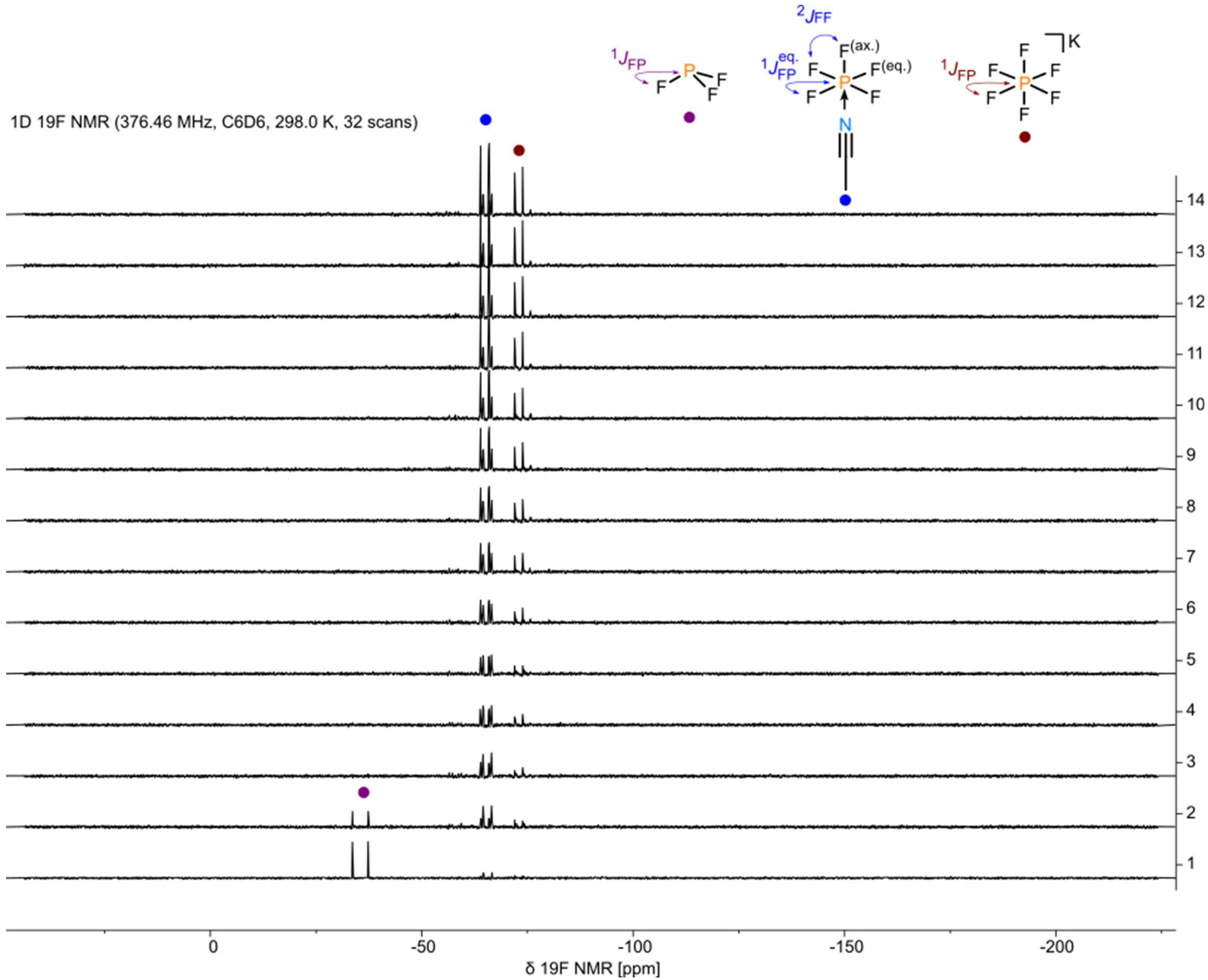

Figure S71. In situ ^{19}F{^{1}H} NMR (376.48 MHz, MeCN, C_6D_6) monitoring of the reaction at 25 ° over 21 h (conditions: 0.25 equiv. P_4, 5.0 equiv. DDQ, 7.5 equiv. KF, MeCN:C_6H_6 (9:1, 0.01 M), 18 h, 25 °C, N_2; Δt = 1 h until t = 5 h, then Δt = 2 h), showing formation of PF_3 (δ 34.5 ppm, d, $^1J_{FP}$ = 1400.9 Hz), PF_5·MeCN (δ – 64.6 ppm, dd, $^1J_{FP}$ = 755.3, $^2J_{FF}$ = 51.0 Hz, $F^{(eq.)}$) and KPF_6 (δ – 73.2 ppm, d, $^1J_{PF}$ = 707.0 Hz).

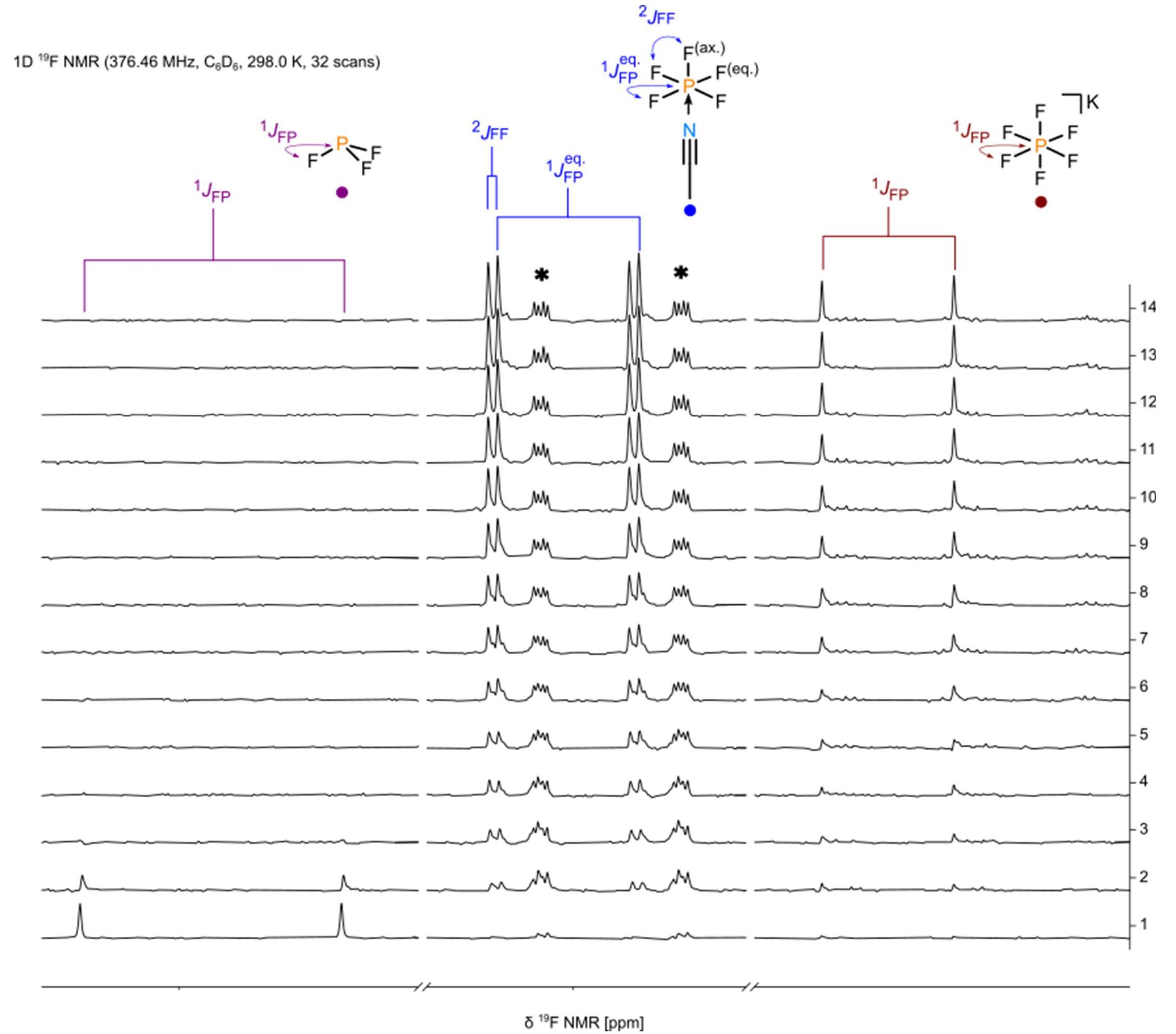

Figure S72. Section of the in situ $^{19}F\{^{1}H\}$ NMR (376.48 MHz, MeCN, C_6D_6) monitoring of the reaction at 25 ° over 21 h (conditions: 0.25 equiv. P_4, 5.0 equiv. DDQ, 7.5 equiv. KF, MeCN:C_6H_6 (9:1, 0.01 M), 18 h, 25 °C, N_2; Δt = 1 h until t = 5 h, then Δt = 2 h), showing formation of PF_3 (δ 34.5 ppm, d, $^{1}J_{FP}$ =1400.9 Hz), PF_5·MeCN (δ – 64.6 ppm, dd, $^{1}J_{FP}$ = 755.3, $^{2}J_{FF}$ = 51.0 Hz, $F^{(eq.)}$) and KPF_6 (δ – 73.2 ppm, d, $^{1}J_{PF}$ = 707.0 Hz). * Multiplet atrrtibuted to PF_5·DDQ adducts, where DDQ is either neutral, the protonated hydroquinone $DDQH^{-}$, or DDQ^{2-}.

5. ESI-MS

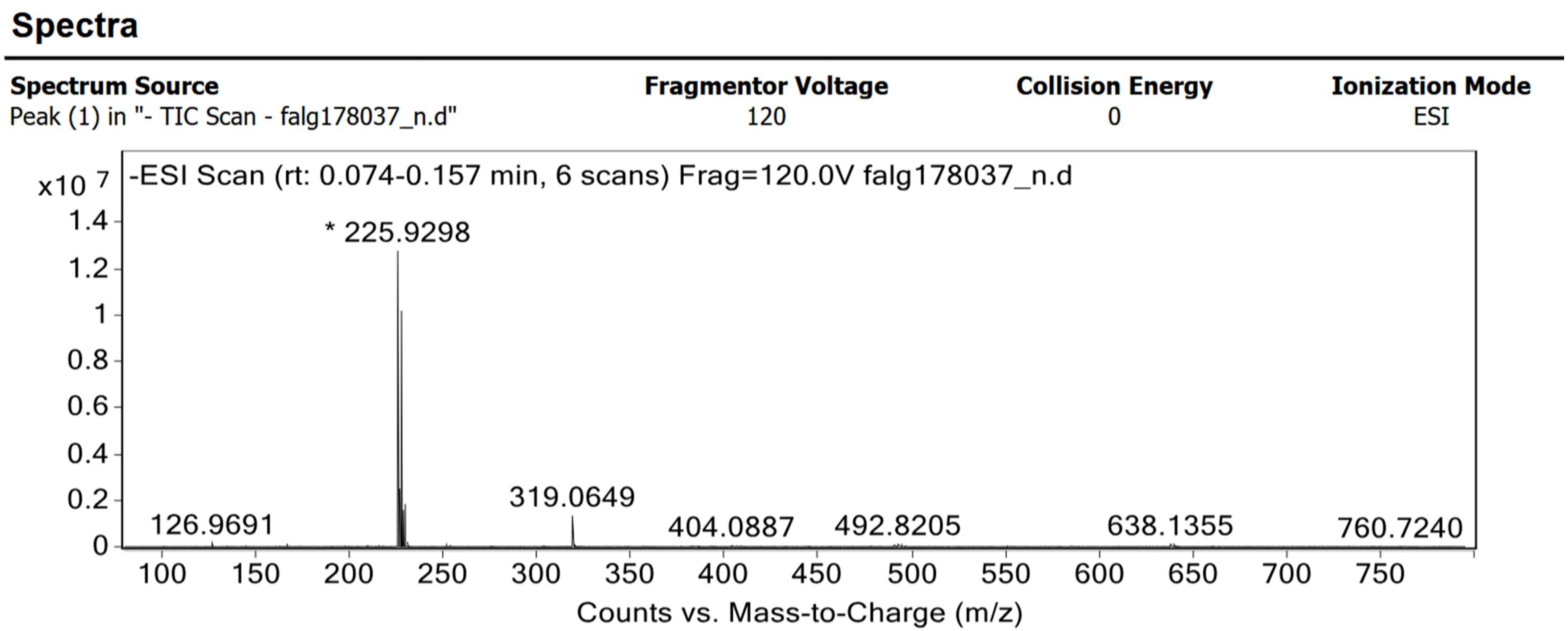

Figure S73. Off-line ESI-MS spectrum of crude reaction mixture under the conditions specified in Table S17, entry 1 (negative ion mode).

**Sample Name**	TEG	**Position**		**Instrument Name**	LC-QTOF	**User Name**	
**Inj Vol**	Unknown / Injection	**InjPosition**		**SampleType**	Sample	**IRM Calibration Status**	All Ions Missed
**Data Filename**	falg178037_n.d	**ACQ Method**	offline_neg_250915.m	**Comment**	Offline ESI (ACN)	**Acquired Time**	9/15/2025 4:13:35 PM (

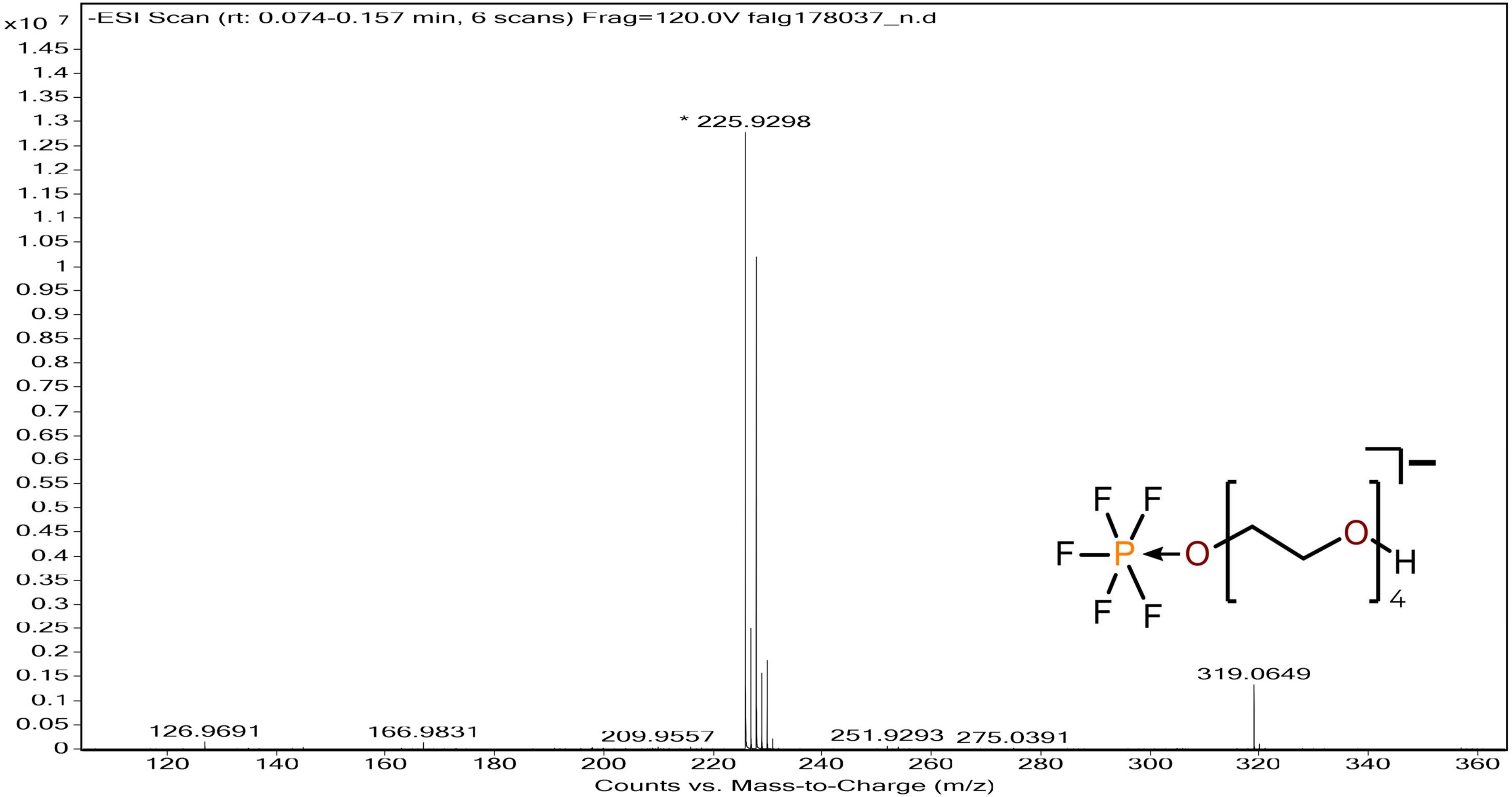

Figure S74. Section of the off-line ESI-MS spectrum of crude reaction mixture under the conditions specified in Table S17, entry 1, showing M^- peaks for $DDQ^{\cdot-}$ (calculated: 225.9136 m/z; found: 225.9298 m/z) and PF_5·TEG^- (calculated: 319.0733 m/z; found: 319.0619 m/z).

6. References

(1) Märsch, J.; Reiter, S.; Rittner, T.; Rodriguez-Lugo, R. E.; Whitfield, M.; Scott, D. J.; Kutta, R. J.; Nuernberger, P.; de Vivie-Riedle, R.; Wolf, R. Cobalt-Mediated Photochemical C−H Arylation of Pyrroles. Angew. Chem. Int. Ed. 2024, 63 (28), e202405780. https://doi.org/10.1002/anie.202405780.

(2) John, K.-P.; Schmutzler, R. Chemie Der Phosphorfluoride, XXXVII / Phosphorus-Fluorine Chemistry, XXXVII: 19F-Kernmagnetische Resonanzuntersuchungen an Phosphorpentafluorid-Basen-Komplexen / ^{19}F Nuclear Magnetic Resonance Investigation of Phosphorus Pentafluoride-Base Complexes. Z. Naturforsch. B. 1974, 29 (11–12), 730–733. https://doi.org/10.1515/znb-1974-11-1208.

(3) Budnikova, Yu. G.; Kargin, Yu. M.; Zaripov, I. M.; Romakhin, A. S.; Ignat'ev, Yu. A.; Nikitin, E. V.; Tomilov, A. P. Electrochemically Induced Processes of Formation of Phosphorus Acid Derivatives. 4. Synthesis of Trialkyl Phosphates from White Phosphorus. Russ. Chem. Bull. 1992, 41 (9), 1585–1588. https://doi.org/10.1007/BF00863577.

(4) Clark, R. J.; Belefant, H.; Williamson, S. M. Phosphorus Trifluoride. In Inorganic Syntheses; Angelici, R. J., Ed.; Inorg. Synth.; John Wiley & Sons, Inc: Hoboken, NJ, USA, 1990; pp 310–315. https://doi.org/10.1002/9780470132593.ch77.

(5) Clark, R. J.; Busch, M. A. Stereochemical Studies of Metal Carbonylphosphorus Trifluoride Complexes. Acc. Chem. Res. 1973, 6 (7), 246–252. https://doi.org/10.1021/ar50067a005.

(6) Campion, C. L.; Li, W.; Lucht, B. L. Thermal Decomposition of LiPF6-Based Electrolytes for Lithium-Ion Batteries. J. Electrochem. Soc. 2005, 152 (12), A2327. https://doi.org/10.1149/1.2083267.

(7) Bedford, R. B.; Betham, M.; Coles, S. J.; Horton, P. N.; López-Sáez, M.-J. The Influence of Steric Bulk on the Geometry of Triarylphosphite-Based Palladacycles and Their Tricyclohexylphosphine Adducts. Polyhedron 2006, 25 (4), 1003–1010. https://doi.org/10.1016/j.poly.2005.11.014.

(8) Marat, R. K.; Janzen, A. F. Fluorine Exchange between Four-, Five-, and Six-Coordinate Silicon Compounds. Can. J. Chem. 1977, 55 (22), 3845–3849. https://doi.org/10.1139/v77-543.

(9) Cresswell, A. J.; Davies, S. G.; Roberts, P. M.; Thomson, J. E. Beyond the Balz–Schiemann Reaction: The Utility of Tetrafluoroborates and Boron Trifluoride as Nucleophilic Fluoride Sources. Chem. Rev. 2015, 115 (2), 566–611. https://doi.org/10.1021/cr5001805.

(10) Riesel, L.; Kant, M. Die Reaktion der Zweikomponentensysteme $P(OR)_3 - x(NR_2)_x$ (x = 0–3)/CCl_4 und P_4/CCl_4 mit HF-Donatoren. Z. Anorg. Allg. Chem. 1985, 531 (12), 73–81. https://doi.org/10.1002/zaac.19855311211.

(11) Miller, J. S.; Krusic, P. J.; Dixon, D. A.; Reiff, W. Michael.; Zhang, J. H.; Anderson, E. C.; Epstein, A. J. Radical Ion Salts of 2,3-Dichloro-5,6-Dicyanobenzoquinone and Metallocenes. A Reexamination of Their Magnetic and Spectroscopic Properties. J. Am. Chem. Soc. 1986, 108 (15), 4459–4466. https://doi.org/10.1021/ja00275a036.

(12) Al-Raqa, S. Y. The Synthesis and Photophysical Properties of Novel, Symmetrical, Hexadecasubstituted Zn Phthalocyanines and Related Unsymmetrical Derivatives. Dyes Pigm. 2008, 77 (2), 259–265. https://doi.org/10.1016/j.dyepig.2007.05.002.

(13) Pinkus, A. G.; Waldrep, P. G.; Ma, S. Y. Structure of Products from Reactions of Phosphorus Pentachloride with Phenyl Salicylate and 2-hydroxybenzophenone; Related Compounds. P31 N.M.R. and Chemical Studies. J. Heterocycl. Chem. 1965, 2, (4), 357–365. https://doi.org/10.1002/jhet.5570020407.

(14) Slitikov, P. V.; Bogoyavlenskii, V. A.; Rasadkina, E. N.; Nifant'ev, E. E. Phosphorylation of 1,4-Bis(Hydroxymethyl)Benzene with Trivalent Phosphorus Derivatives. Russ. J. Gen. Chem. 2007, 77 (6), 1028–1033. https://doi.org/10.1134/S1070363207060126.

(15) Wynn, D. The Solubility of Alkali-Metal Fluorides in Non-Aqueous Solvents with and without Crown Ethers, as Determined by Flame Emission Spectrometry. Talanta 1984, 31 (11), 1036–1040. https://doi.org/10.1016/0039-9140(84)80244-1.

(16) Abraham, R.; Abdulkhadar, M.; Asokan, C. V. Ultrasonic Investigation of Molecular Interaction in Binary Mixtures of Nitriles with Methanol/Toluene. J. Chem. Thermodyn. 2000, 32 (1), 1–16. https://doi.org/10.1006/jcht.1999.0608.

(17) Stejskal, E. O.; Tanner, J. E. Spin Diffusion Measurements: Spin Echoes in the Presence of a Time-Dependent Field Gradient. J. Chem. Phys. 1965, 42 (1), 288–292. https://doi.org/10.1063/1.1695690.

(18) Macchioni, A.; Ciancaleoni, G.; Zuccaccia, C.; Zuccaccia, D. Determining Accurate Molecular Sizes in Solution through NMR Diffusion Spectroscopy. Chem. Soc. Rev. 2008, 37 (3), 479–489. https://doi.org/10.1039/B615067P.

(19) Valencia, D. P.; González, F. J. Estimation of Diffusion Coefficients by Using a Linear Correlation between the Diffusion Coefficient and Molecular Weight. J. Electroanal. Chem. 2012, 681, 121–126. https://doi.org/10.1016/j.jelechem.2012.06.013.

(20) Ben-Amotz, D.; Willis, K. G. Molecular Hard-Sphere Volume Increments. J. Phys. Chem. 1993, 97 (29), 7736–7742. https://doi.org/10.1021/j100131a051.

(21) Evans, R.; Dal Poggetto, G.; Nilsson, M.; Morris, G. A. Improving the Interpretation of Small Molecule Diffusion Coefficients. Anal. Chem. 2018, 90 (6), 3987–3994. https://doi.org/10.1021/acs.analchem.7b05032.

(22) Kolditz, L.; Beierlein, I. Zur Dissoziation von $PF_5{\cdot}CH_3CN$ Und $AsF_5{\cdot}CH_3CN$ in Acetonitril. Z. Chem. 1967, 7 (12), 468–469. https://doi.org/10.1002/zfch.19670071220.

(23) Christe, K. O.; Wilson, W. W.; Wilson, R. D.; Bau, R.; Feng, J. A. Syntheses, Properties, and Structures of Anhydrous Tetramethylammonium Fluoride and Its 1:1 Adduct with Trans-3-Amino-2-Butenenitrile. J. Am. Chem. Soc. 1990, 112 (21), 7619–7625. https://doi.org/10.1021/ja00177a025.

(24) Capó, N.; Barrios, L. A.; Cardona, J.; Ribas-Ariño, J.; Teat, S. J.; Roubeau, O.; Aromí, G. The Template Effect of a SiF_6^{2-} Guest Drives the Formation of a Heteroleptic Fe(II) Coordination Helicate. Chem. Commun. 2022, 58 (78), 10969–10972. https://doi.org/10.1039/D2CC04559A.

(25) Longuet, M.; Vitse, K.; Martin-Mingot, A.; Michelet, B.; Guégan, F.; Thibaudeau, S. Determination of the Hammett Acidity of HF/Base Reagents. J. Am. Chem. Soc. 2024. https://doi.org/10.1021/jacs.4c02344.
